# Palladium-catalyzed dearomative 1,4-difunctionalization of naphthalenes†
†Electronic supplementary information (ESI) available. CCDC 1982544-1982545. For ESI and crystallographic data in CIF or other electronic format see DOI: 10.1039/d0sc02816a


**DOI:** 10.1039/d0sc02816a

**Published:** 2020-06-10

**Authors:** Ping Yang, Chao Zheng, Yu-Han Nie, Shu-Li You

**Affiliations:** a State Key Laboratory of Organometallic Chemistry , Center for Excellence in Molecular Synthesis , Shanghai Institute of Organic Chemistry , University of Chinese Academy of Sciences , Chinese Academy of Sciences , 345 Lingling Lu , Shanghai 200032 , China . Email: slyou@sioc.ac.cn

## Abstract

A highly diastereoselective dearomatization of naphthalenes *via* a Pd-catalyzed 1,4-difunctionalization reaction is described. In the presence of a commercially available palladium precursor and ligand, intramolecular dearomative Heck-type insertion provides π-allylpalladium intermediates which are readily captured by a series of nucleophiles in excellent yields (up to 99%). This reaction features mild conditions, broad substrate scope, and useful transformations of the products.

## Abstract 2

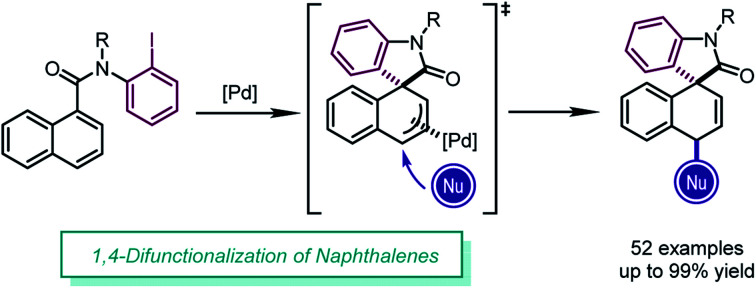
The dearomative 1,4-difunctionalization of naphthalenes is achieved by imitating the reactivity of simple conjugated dienes in aromatic systems, providing functionalized spirooxindoles in high yields (up to 99%) with exclusive diastereoselectivity.

## Introduction

**Scheme 1:**
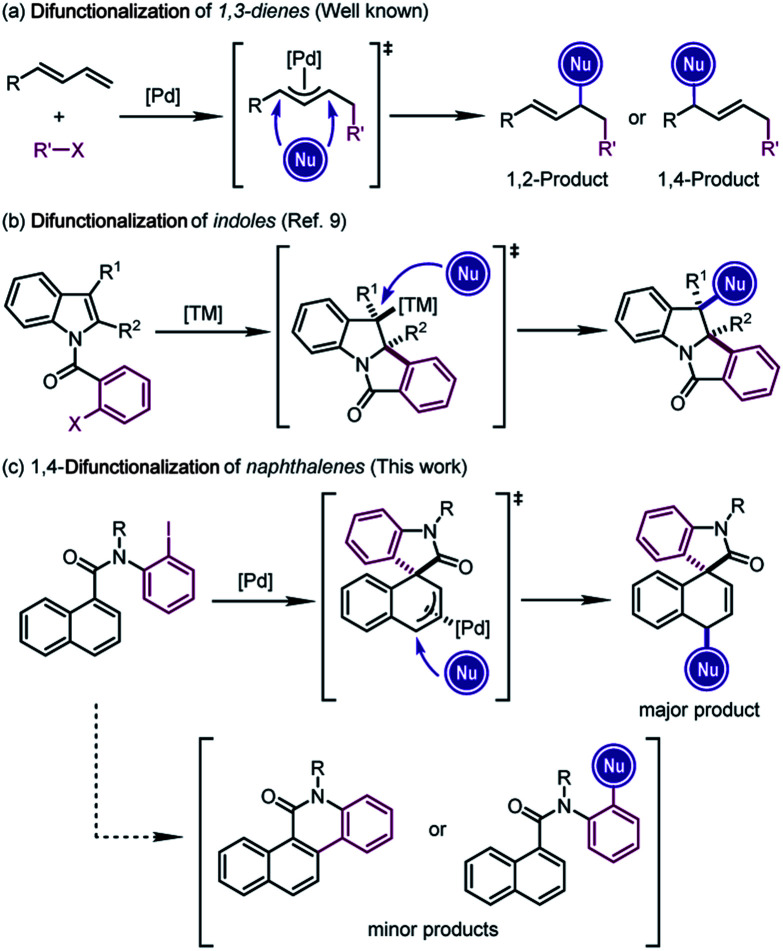
Difunctionalization of 1,3-dienes, indoles and naphthalenes.

The difunctionalization of alkenes is widely recognized as a powerful approach to generate significant molecular complexity from simple chemical feedstock.^1^ In particular, Pd-catalyzed difunctionalization of 1,3-dienes, which introduces two functional groups across the conjugated C=C double bonds, has witnessed significant progress in the past decade.^2^ Mechanistically, Pd(ii) complexes, usually generated from the oxidative addition of Pd(0) precursors, have been employed to achieve these reactions through Heck insertion^3^ to form a π-allylpalladium species. The subsequent regioselective nucleophilic attacks afford 1,2- or 1,4-addition-like products (Scheme 1a).2*d* However, the scope of these transformations was in general limited to structurally simple dienes^4^ and furans.^5^

In line with our continuous interest in catalytic dearomatization reactions,^6^ we envisioned that the formal “conjugated diene” structure of the phenyl ring might serve as the equivalent of 1,3-dienes. However, the dearomatization of electronically unbiased aromatic compounds such as naphthalenes and benzenes remained challenging,^7^ due to the generally higher aromatic stabilization energies of plain arenes compared with their heteroaromatic counterparts (36 kcal mol^–1^ for benzene and 22 kcal mol^–1^ for pyrrole).6*r* Therefore, the translation of Pd-catalyzed difunctionalization of 1,3-dienes to aromatic systems would open a new window for the dearomatization of non-activated arenes.

Inspired by the recent developments in Pd-catalyzed dearomative Heck reactions^8^ that are terminated by the nucleophilic attack on alkylpalladium intermediates (Scheme 1b),^9^ we realized that the π-allylpalladium intermediates formed from the Heck-type insertion into the naphthalene ring might also be captured using external nucleophiles, furnishing the dearomative 1,4-difunctionalization of naphthalenes (Scheme 1c).^10^ The successful execution of this reaction design relied on the judicious selection of the catalytic system which could overcome the thermodynamic disadvantage of the dearomatization process and at the same time avoid the competitive C–H activation or direct cross-coupling reactions. Herein we report our results from this study.

## Results and discussion

We began our investigation by studying the 1,4-difunctionalization of *N*-(2-iodophenyl)-*N*-methyl-1-naphthamide (**1a**) with dimethyl malonate (**2a**) (Table 1). Firstly, we tested different ligands in the presence of PdCl_2_ (10 mol%), NaH (2.0 equiv.), and Ag_3_PO_4_ (1.0 equiv.) in DMA at 100 °C. When BINAP (**L1**), BINAP(O) (**L2**), and Feringa phosphoramidite (*rac*-**L3**) were used as the ligand respectively, the dearomatized product **3a** was obtained as a single diastereomeric isomer in good yields (67–90%) (entries 1–3), whose structure and relative configuration were confirmed by X-ray crystallographic analysis. The relative configuration of **3a** revealed that the *in situ* formed π-allylpalladium intermediate was attacked by the nucleophile *via* an outer sphere mechanism. On the other hand, the reaction with the PHOX ligand (**L4**) was sluggish (entry 4). Subsequently, different solvents were examined by using *rac*-**L3** as the ligand (entries 5–8). DMA was found to be the optimal one among those tested. In particular, the desired reaction was prohibited significantly when ^*t*^BuOH was employed (entry 6). Next, the effects of various bases were examined (entries 9–12). This revealed that **3a** could be obtained in moderate yields by using K_2_CO_3_ or K_3_PO_4_, while the target product was not observed when 1,2,2,6,6-pentamethylpiperidine (PMP) or ^*t*^BuOK was employed. Surprisingly, the judicious choice of silver salts was quite critical to this reaction. Among those tested, Ag_3_PO_4_ provided the optimal reaction outcomes. Other commonly used silver salts including AgOTf, AgNTf_2_, and AgBF_4_ were ineffective (entries 13–15). Finally, the optimal yield of **3a** (95%) was obtained by using a pre-prepared sodium salt of **2a** with lower catalyst loading (5 mol%) (entry 16).

**Table 1:** Optimization of the reaction conditions^*a*^

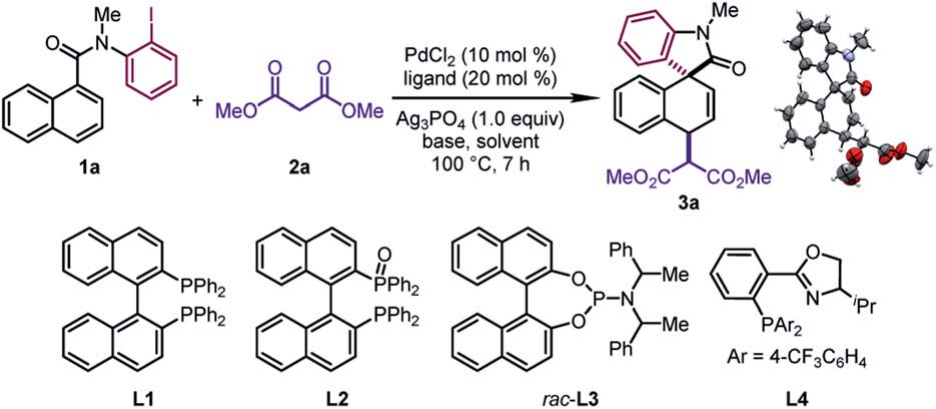					
Entry	Ligand	Base	Solvent	**1a** ^*b*^ (%)	**3a** ^*b*^ (%)
1^*c*^	**L1**	NaH	DMA	—	79
2^*c*^	**L2**	NaH	DMA	15	67
3	*rac*-**L3**^*d*^	NaH	DMA	—	90 (90^*e*^)
4^*c*^	**L4**	NaH	DMA	44	43
5	*rac*-**L3**^*d*^	NaH	Toluene	69	22
6	*rac*-**L3**^*d*^	NaH	^*t*^BuOH	Quant.	Trace
7	*rac*-**L3**^*d*^	NaH	DCE	63	36
8	*rac*-**L3**^*d*^	NaH	Dioxane	52	51
9	*rac*-**L3**^*d*^	K_2_CO_3_	DMA	43	46
10	*rac*-**L3**^*d*^	K_3_PO_4_	DMA	51	29
11	*rac*-**L3**^*d*^	PMP^*f*^	DMA	Quant.	Trace
12	*rac*-**L3**^*d*^	^*t*^BuOK	DMA	66	Trace
13^*g*^	*rac*-**L3**^*d*^	NaH	DMA	66	27
14^*h*^	*rac*-**L3**^*d*^	NaH	DMA	84	8
15^*i*^	*rac*-**L3**^*d*^	NaH	DMA	77	5
16^*j*^	*rac*-**L3**^*d*^	NaH	DMA	—	97 (95^*e*^)

^*a*^Reaction conditions: **1a** (0.2 mmol), **2a** (0.4 mmol), PdCl_2_ (0.02 mmol), ligand (0.04 mmol), base (0.4 mmol), and Ag_3_PO_4_ (0.2 mmol) in solvent (1.0 mL) at 100 °C.

^*b*^Yield determined by ^1^H NMR using CH_2_Br_2_ (0.2 mmol) as an internal standard.

^*c*^Ligand (0.02 mmol).

^*d*^ *rac*-**L3**: (*R*_a_,*R*,*R* + *S*_a_,*S*,*S*) : (*S*_a_,*R*,*R* + *R*_a_,*S*,*S*) = 1 : 4.

^*e*^Isolated yield.

^*f*^PMP: 1,2,2,6,6-pentamethylpiperidine.

^*g*^AgOTf as the silver salt.

^*h*^AgNTf_2_ as the silver salt.

^*i*^AgBF_4_ as the silver salt.

^*j*^Sodium salt of **2a** (pre-prepared by mixing NaH (0.4 mmol) and **2a** (0.4 mmol) in DMA at room temperature for 0.5 h), PdCl_2_ (0.01 mmol), and *rac*-**L3** (0.02 mmol).

With the optimal conditions in hand, we surveyed the generality of this novel 1,4-difunctionalization by allowing various naphthalene derivatives **1** to react with sodium salts of dialkyl malonates **2** (Table 2). When the methyl group on the nitrogen tether of **1** was changed to isopropyl or benzyl groups, the desired products **3b** and **3c** were obtained in good yields (82–86%). The substrates bearing an electron-donating group (Me and OMe) or halide (F, Cl, and Br) at the *para* position of the phenyl ring led to **3d–3h** in 75–98% yields. The good tolerance with halides would offer a handle on subsequent transformations. In contrast, when an electron-withdrawing group (CF_3_, CO_2_Me, and CN) was incorporated into the aryl iodide moiety, the corresponding products (**3i**, **3j**, **3l**) were formed in moderate yields (40–51%). Notably, an *ortho* substituent on the phenyl ring was well tolerated, affording the dearomatized product **3m** in 80% yield. Naphthalene derivatives bearing a substituent at the C6 position furnished the products **3n–3p** in reasonable yields (39–82%). It is worth noting that **3a** could be obtained in 75% yield by using *N*-(2-bromophenyl)-*N*-methyl-1-naphthamide as the substrate and BINAP as the ligand. In addition, when diethyl malonate or dibenzyl malonate was utilized as the nucleophile, the desired products **3q** and **3r** were obtained with good yields (81–87%). The dearomatized product **3s** bearing two all-carbon quaternary stereocenters could be afforded in 28% yield. The low yield probably resulted from the unfavorable steric hindrance in the second step.

**Table 2:** Substrate scope of naphthalenes and malonic diesters^*a*^

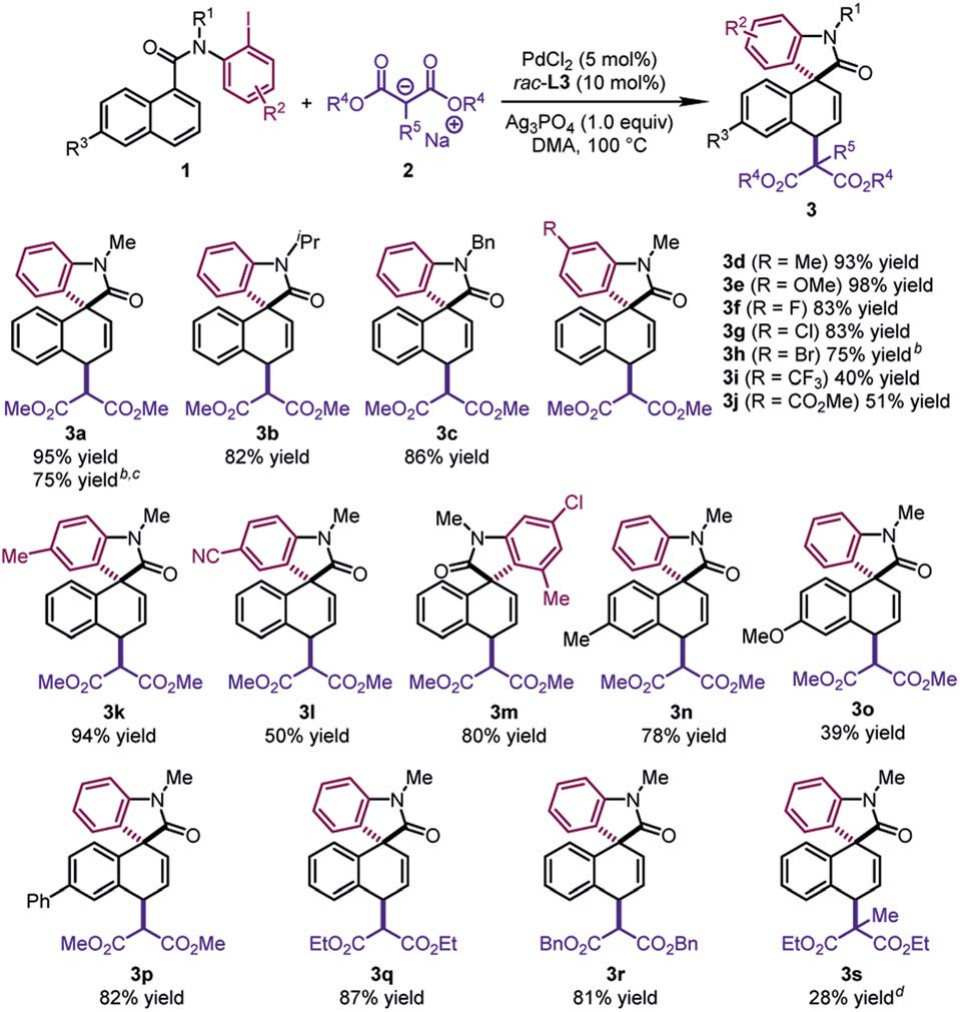

^*a*^ **1** (0.2 mmol), **2** (0.4 mmol, pre-prepared from malonic ester with NaH), PdCl_2_ (0.01 mmol), *rac*-**L3** (0.02 mmol), and Ag_3_PO_4_ (0.2 mmol) in DMA (1.0 mL) at 100 °C.

^*b*^Pre-synthesized PdCl_2_–BINAP complex (0.01 mmol) was used.

^*c*^ *N*-(2-Bromophenyl)-*N*-methyl-1-naphthamide was used at 120 °C.

^*d*^PdCl_2_ (0.02 mmol) and *rac*-**L3** (0.04 mmol) at 120 °C.

Next, different 1,3-diketones were explored under slightly modified conditions (Table 3). When acetylacetone and 3,5-heptanedione were employed as the coupling partners, the corresponding products **4a** and **4b** were delivered in good yields (67–69%). Notably, cyclic β-diketones were also tolerated, leading to **4c** and **4d** in moderate yields (42–55%).

**Table 3:** Substrate scope of 1,3-diketones^*a*^

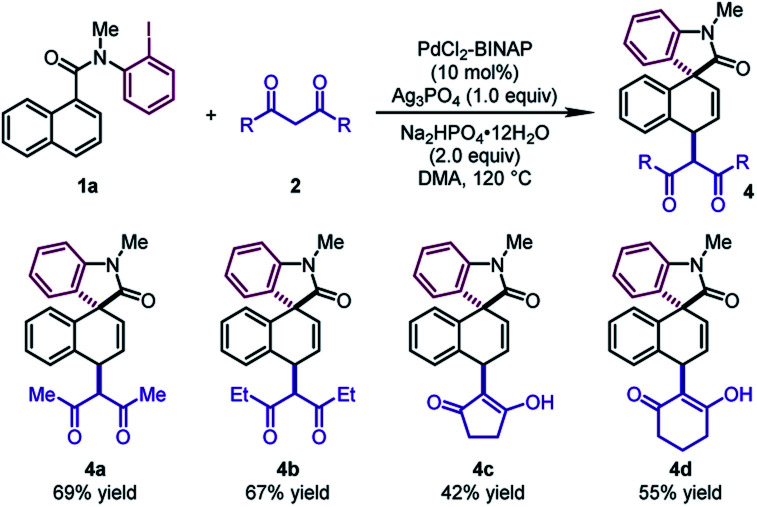

^*a*^ **1a** (0.2 mmol), **2** (0.4 mmol), pre-synthesized PdCl_2_–BINAP complex (0.02 mmol), Ag_3_PO_4_ (0.2 mmol), and Na_2_HPO_4_·12H_2_O (0.4 mmol) in DMA (1.0 mL) at 120 °C.

The reaction design was successfully applied to the sodium salts of β-ketoesters (Table 4). The reactions of diverse alkyl substituted β-ketoesters furnished the desired products in good to excellent yields (**5a–5f**, 56–99%). Aryl β-ketoesters also participated smoothly in the reaction, regardless of the electronic properties of the aryl group, delivering **5g–5i** in good yields (59–76%). Of particular note is that 2-thienyl and 2-furyl β-ketoesters could be converted to **5j** and **5k** in moderate to good yields (45–89%).

**Table 4:** Substrate scope of β-ketoesters^*a*^ ^,^^*b*^

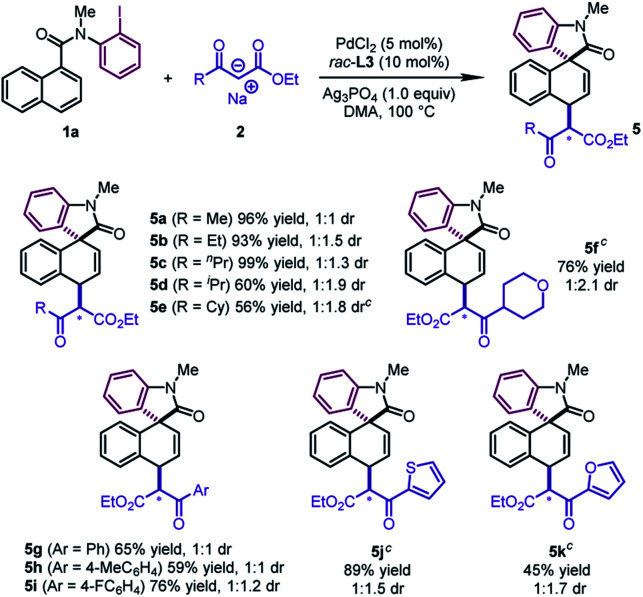

^*a*^ **1a** (0.2 mmol), **2** (0.4 mmol, pre-prepared from β-ketoester with NaH), PdCl_2_ (0.01 mmol), *rac*-**L3** (0.02 mmol), and Ag_3_PO_4_ (0.2 mmol) in DMA (1.0 mL) at 100 °C.

^*b*^The diastereomeric selectivity originates from the reversal of the relative configuration at the position denoted with an asterisk.

^*c*^PdCl_2_ (0.02 mmol), *rac*-**L3** (0.04 mmol).

**Scheme 2:**
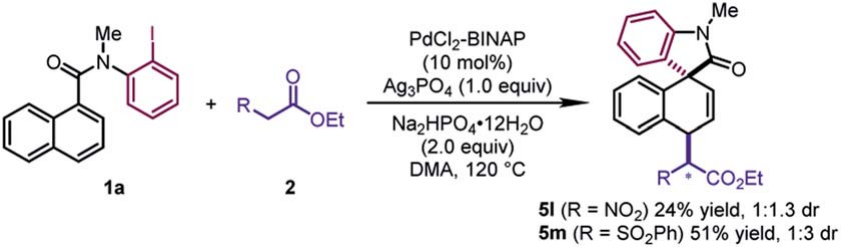
Substrate scope of esters.

In addition to β-ketoesters, the esters bearing an electron-withdrawing group (NO_2_ and SO_2_Ph) at the α-position were proved to be viable participants, leading to **5l** and **5m** in moderate yields (24–51%) (Scheme 2). Although obtained as a mixture of a pair of diastereoisomers with poor dr values, the products could undergo decarboxylation to deliver pure dearomatized compounds (*vide infra*).

To further demonstrate the generality of this reaction, we focused on developing the 1,4-difunctionalization with nitrogen-based nucleophiles (Table 5). Various benzyl amines were investigated as the nucleophiles. The corresponding products **6a** and **6b** could be obtained in excellent yields (97–99%). Moreover, furfurylamine and 2-thiophenemethylamine were also compatible, leading to dearomatized products **6c** and **6d** in good yields (76–98%). Cyclic secondary amines, such as pyrrolidine, piperidine and morpholine, reacted as the coupling partners to form the tertiary amines **6f–6h** in excellent yields (98–99%). Diethylamine and cyclohexylamine also participated in the reaction smoothly, leading to **6e** and **6i** in excellent yields (88–95%). It is noteworthy that various anilines regardless of electronic properties were also viable reaction partners. The desired products **6k–6m** could be delivered in high yields (80–99%). The structure of **6k** was determined by X-ray crystallographic analysis. Notably, the reactions of tryptamine and allylamine led to **6j** and **6n** in low yields. When (*R*)-1-phenylethylamine was used, **6o** was obtained in 96% yield with a 1 : 1 dr.

**Table 5:** Substrate scope of amines^*a*^

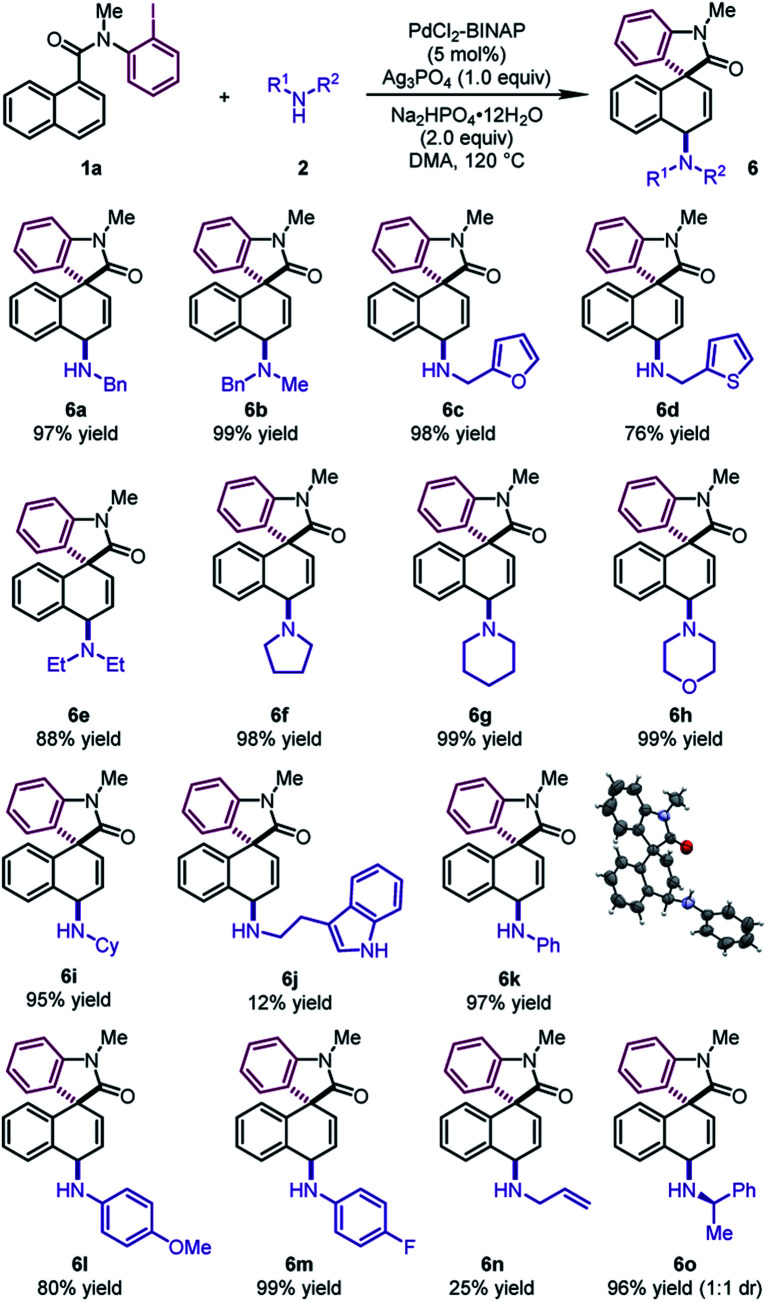

^*a*^ **1a** (0.2 mmol), **2** (0.4 mmol), pre-synthesized PdCl_2_–BINAP complex (0.01 mmol), Ag_3_PO_4_ (0.2 mmol), and Na_2_HPO_4_·12H_2_O (0.4 mmol) in DMA (1.0 mL) at 120 °C.

Preliminary investigations on the enantioselective variants of the dearomative 1,4-difunctionalization reactions of naphthalene derivatives with dimethyl malonate or aniline were performed. Unfortunately, the utilization of a series of chiral ligands did not afford satisfactory asymmetric induction (see the ESI† for details).

**Scheme 3:**
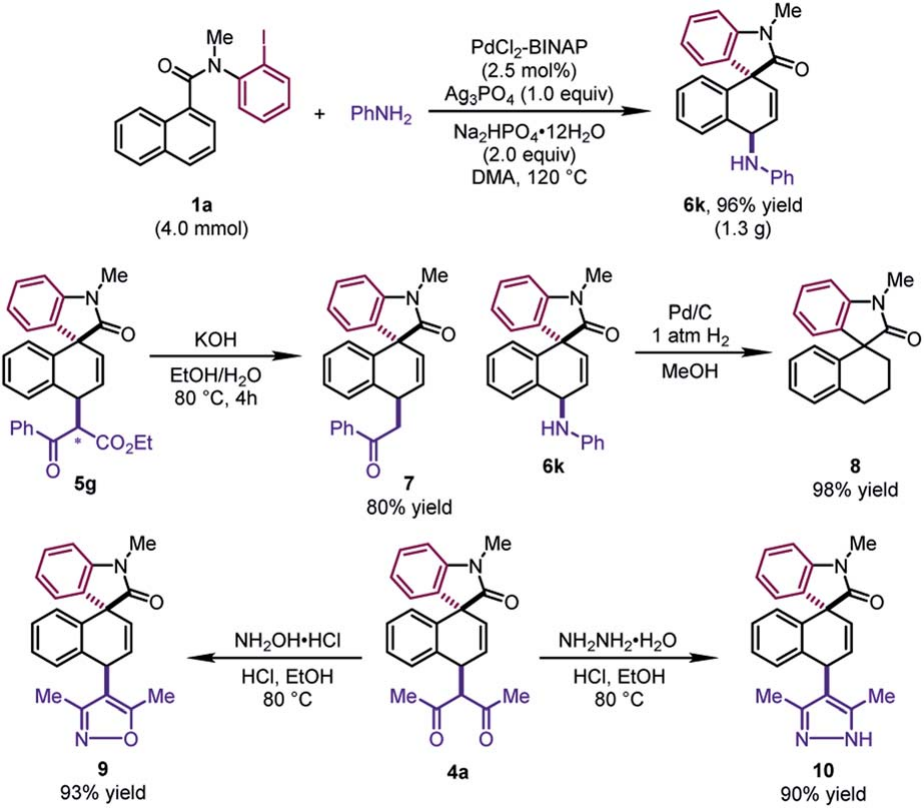
Gram-scale reaction and transformations of the products.

To test the practicality of this method, a gram-scale dearomative 1,4-difunctionalization reaction of **1a** (4.0 mmol) and aniline with a lower catalyst loading (2.5 mol%) was carried out (Scheme 3). The desired product **6k** could be afforded in 96% yield (1.3 g). Some synthetic transformations of the dearomatized products have been examined. The mixture of two diastereoisomers of **5g** (1 : 1 dr) could be decarboxylated to form **7** in 80% yield. The newly formed C–N bond of **6k** could be cleaved by the hydrogenolysis reaction with Pd/C, leading to **8** in excellent yield. In addition, the β-diketone compound **4a** could be condensed with hydroxylamine hydrochloride and hydrazine monohydrate, furnishing the corresponding isoxazole **9** and pyrazole **10** in good yields, respectively.

## Conclusions

In summary, we have developed a Pd-catalyzed dearomative 1,4-difunctionalization of naphthalene derivatives by mimicking the reactivity of simple conjugated dienes in more challenging electronically unbiased aromatic systems. Diverse nucleophiles were found to be compatible with the reaction conditions. Various functionalized spirooxindoles could be obtained efficiently in good to excellent yields (up to 99%) with exclusive diastereoselectivity. Further exploration of the application of this methodology is currently underway in our laboratory.

## Conflicts of interest

There are no conflicts to declare.

## Supplementary Material

Supplementary information

Crystal structure data
