# Supplementary material for: Palladium-catalyzed dearomative 1,4-difunctionalization of naphthalenes
Source: Chem Sci. 2020 Jun 10;11(26):6830–5. doi: 10.1039/d0sc02816a (PMC7504896; doi:10.1039/d0sc02816a)
Supplement: Supplementary file 1 [file SC-011-D0SC02816A-s001.pdf]

Electronic Supplementary Material (ESI) for Chemical Science.
This journal is © The Royal Society of Chemistry 2020

Supplimentary Information

Palladium-Catalyzed Dearomative 1,4-Difunctionalization of Naphthalenes

Ping Yang, Chao Zheng, Yu-Han Nie, and Shu-Li You*

State Key Laboratory of Organometallic Chemistry, Center for Excellence in Molecular Synthesis, Shanghai Institute of Organic Chemistry, University of Chinese Academy of Sciences, Chinese Academy of Sciences, 345 Lingling Lu, Shanghai 200032, China

E-mail: slyou@sioc.ac.cn

Table of Contents

General methods S2
General procedure for the preparation of substrates (**1a-1p**) S3-S14
General procedure for Pd-catalyzed dearomative reaction S15-S47
Preliminary asymmetric studies S48
Transformations of products S49-S52
X-Ray crystal data S53-S55
References S56
Copies of NMR spectra S57-S206

General methods

Unless stated otherwise, all reactions were carried out in flame-dried glassware under a dry argon atmosphere. All solvents were purified and dried according to standard methods prior to use. ^{1}H and ^{19}F NMR spectra were recorded at room temperature unless otherwise noted on a Bruker, Varian or Agilent instrument (400 MHz and 377 MHz, respectively) and internally referenced to tetramethylsilane signal or residual protio solvent signals, respectively. ^{13}C NMR spectra were recorded on a Bruker (101 MHz) or Varian instrument (101 MHz) and internally referenced to residual solvent signals. Data for ^{1}H NMR are recorded as follows: chemical shift (δ, ppm), multiplicity (s = singlet, d = doublet, t = triplet, m = multiplet or unresolved, br = broad singlet, coupling constant (s) in Hz, integration). Data for ^{13}C NMR and ^{19}F NMR are reported in terms of chemical shift (δ, ppm).

General procedure for the preparation of substrates

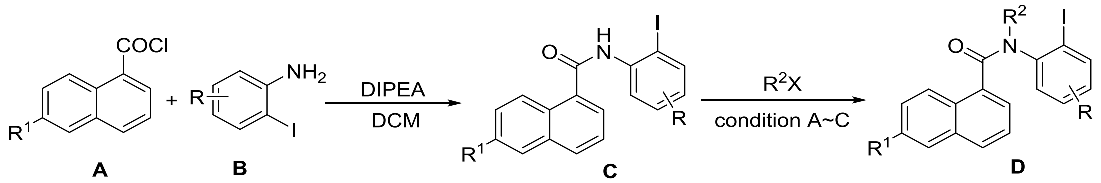

1-Naphthoyl chloride **A** (1.0 equiv) was dissolved in DCM (0.2 M), then DIPEA (1.3 equiv) and substituted 2-iodoaniline **B** (1.2 equiv) were added at room temperature. After completion (monitored by TLC), the reaction mixture was quenched with saturated aqueous $NaHCO_3$ and extracted with DCM (50 mL × 3). The combined DCM was dried over anhydrous Na_2SO_4 and filtrated. After the solvent was concentrated under reduced pressure, the crude product **C** was used without purification.

Conditions A:

To a solution of crude product **C** in THF (0.2 M), potassium tert-butoxide (1.2 equiv) was added carefully at room temperature. After 20 min, iodomethane (1.2 equiv) was added dropwise. Then the reaction mixture was stirred at room temperature. After completion (monitored by TLC), the reaction mixture was quenched with saturated aqueous $NaHCO_3$ and extracted with ethyl acetate (50 mL × 3). The combined ethyl acetate extract was dried over anhydrous Na_2SO_4 and filtrated. After the solvent was concentrated under reduced pressure, the crude product was purified by silica gel column chromatography (PE/EtOAc = 20:1) to afford the desired product (**1a**, **1d**-**1p**).

Conditions B:

Crude N-(2-iodophenyl)-1-naphthamide was dissolved in THF (0.4 M) in a sealed tube at 0 °C. Sodium hydride (1.5 equiv) was added carefully. After 20 min, 2-bromopropane (3.0 equiv) was added dropwise. Then the reaction mixture was stirred at 80 °C. After completion (monitored by TLC), the reaction mixture was quenched with H_2O and extracted with ethyl acetate (50 mL × 3). After the solvent

was concentrated under reduced pressure, the crude product was purified by silica gel column chromatography (PE/EtOAc = 20:1) to afford the desired product **1b**.

Conditions C:

To a solution of crude N-(2-iodophenyl)-1-naphthamide in DMF (0.4 M) at 0 °C, sodium hydride (1.3 equiv) was added carefully. After 30 min, benzyl bromide (1.1 equiv) was added dropwise. Then the reaction mixture was stirred at room temperature. After completion (monitored by TLC), the reaction mixture was quenched with H_2O and extracted with ethyl acetate (50 mL × 3). The combined ethyl acetate extract was washed with H_2O (50 mL × 4), dried over anhydrous Na_2SO_4 and filtrated. After the solvent was concentrated under reduced pressure, the crude product was purified by silica gel column chromatography (PE/EtOAc = 20:1) to afford the desired product **1c**.

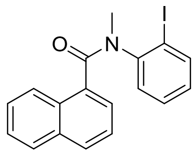

1a: white solid, 16.5 g, 85% yield (from **A** over 2 steps, 50 mmol scale), m.p. = 95-97 °C. Two sets of signals were observed due to the existence of rotamers. The peaks of the minor one are marked with asterisk. ^{1}H NMR (400 MHz, $CDCl_3$) δ 8.20 (d, *J* = 8.4 Hz, 1H), 7.96* (d, *J* = 7.6 Hz, 1H), 7.91-7.88* (m, 2H), 7.73-7.68 (m, 2H), 7.62 (d, *J* = 8.0 Hz, 1H), 7.58-7.51 (m, 1H), 7.49-7.43 (m, 2H), 7.17-7.13 (m, 1H), 7.10-7.06* (m, 1H), 6.93 (dd, *J* = 8.0, 1.6 Hz, 1H), 6.87-6.83 (m, 1H), 6.71-6.67 (m, 1H), 3.51 (s, 3H), 3.06* (s, 3H); ^{13}C NMR (101 MHz, $CDCl_3$) δ 170.5, 146.3, 145.5, 140.2, 140.0, 134.1, 133.9, 133.6, 133.4, 130.4, 130.0, 129.6, 129.5, 129.4, 129.2, 129.14, 129.08, 129.0, 128.5, 128.3, 127.1, 126.8, 126.6, 126.1, 125.7, 125.2, 124.4, 124.33, 124.26, 98.7, 39.9, 36.9. IR (film): v_{max} (cm^{-1}) = 3058, 1645, 1574, 1504, 1466, 1419, 1354, 1304, 1250, 1112, 1058, 1015, 977, 813, 796, 779, 747, 720, 657, 638, 587, 553, 511, 475, 446, 428. HRMS (ESI): Exact mass calcd. for $C_{18}H_{15}NOI$ ($[M+H]^+$): 388.01928. Found: 388.01886.

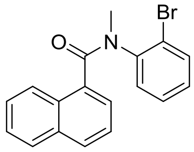

1a': white solid, 0.30 g, 40% yield (from **A** over 2 steps, 2.2 mmol scale), m.p. = 92-95 °C. Two sets of signals were observed due to the existence of rotamers. The peaks of the minor one are marked with asterisk. ^{1}H NMR (400 MHz, $CDCl_3$) δ 8.18 (d, *J* = 8.4 Hz, 1H), 7.91* (t, *J* = 7.6 Hz, 2H), 7.76-7.73 (m, 1H), 7.65 (d, *J* = 8.0 Hz, 1H), 7.57-7.53 (m, 1H), 7.48-7.44 (m, 2H), 7.38-7.37 (m, 1H), 7.19-7.15 (m, 1H), 6.95 (dd, *J* = 7.2, 1.6 Hz, 1H), 6.92-6.84 (m, 2H), 3.52 (s, 3H), 3.09* (s, 3H); ^{13}C NMR (101 MHz, $CDCl_3$) δ 170.7, 143.0, 134.0, 133.9, 133.7, 133.4, 130.4, 129.9, 129.63, 129.60, 129.5, 129.2, 129.1, 128.6, 128.3, 128.2, 127.3, 126.9, 126.6, 126.2, 125.7, 125.3, 124.44, 124.36, 124.3, 122.7, 39.8, 36.5. IR (film): ν_{max} (cm^{-1}) = 3064, 1643, 1575, 1470, 1420, 1353, 1305, 1247, 1116, 1060, 1022, 864, 795, 770, 724, 706, 660, 638, 587, 554, 511, 485, 454, 432. HRMS (ESI): Exact mass calcd. for $C_{18}H_{15}NOBr$ ($[M+H]^+$): 340.03315. Found: 340.03252.

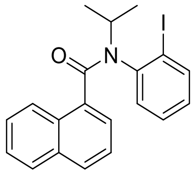

1b: white solid, 0.80 g, 19% yield (from **C** over 1 steps, 10 mmol scale), m.p. = 107-109 °C. Two sets of signals were observed due to the existence of rotamers. The peaks of the minor one are marked with asterisk. ^{1}H NMR (400 MHz, $CDCl_3$) δ 8.19 (d, *J* = 8.4 Hz, 1H), 8.01* (s, 2H), 7.91-7.88 (m, 1H), 7.70-7.63 (m, 2H), 7.60 (d, *J* = 8.4 Hz, 1H), 7.53 (d, *J* = 7.2 Hz, 1H), 7.43-7.40 (m, 1H), 7.15 (t, *J* = 7.6 Hz, 1H), 7.08 (d, *J* = 7.6 Hz, 1H), 6.91 (t, *J* = 7.6 Hz, 1H), 6.67 (t, *J* = 7.6 Hz, 1H), 4.79-4.73 (m, 1H), 4.24-4.21* (m, 1H), 1.68 (d, *J* = 6.4 Hz, 3H), 1.28 (d, *J* = 6.8 Hz, 3H), 1.08* (d, *J* = 6.4 Hz, 6H); ^{13}C NMR (101 MHz, $CDCl_3$) δ 170.4, 143.5, 140.2, 134.8, 133.3, 130.8, 130.3, 129.2, 129.1, 128.4, 128.3, 127.0, 126.5, 126.0, 125.8, 125.1, 124.2, 103.1, 51.4, 22.3, 19.7. IR (film): ν_{max} (cm^{-1}) = 2967, 1631, 1504, 1463, 1421, 1403,

1373, 1346, 1318, 1259, 1217, 1109, 1018, 981, 811, 791, 776, 759, 742, 718, 701, 661, 637, 581, 559, 453. HRMS (ESI): Exact mass calcd. for $C_{20}H_{19}NOI$ ([M+H]$^+$): 416.05058. Found: 416.05014.

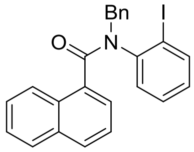

1c: white solid, 1.2 g, 26% yield (from **C** over 1 steps, 10 mmol scale), m.p. = 184-186 °C. Two sets of signals were observed due to the existence of rotamers. The peaks of the minor one are marked with asterisk. ^{1}H NMR (400 MHz, $CDCl_3$) δ 8.05 (d, *J* = 8.0 Hz, 1H), 7.96-7.91* (m, 4H), 7.73-7.69 (m, 2H), 7.62-7.56 (m, 1H), 7.51-7.24 (m, 8H), 7.15 (t, *J* = 7.6 Hz, 1H), 7.03-6.99* (m, 3H), 6.65-6.64 (m, 2H), 6.34 (d, *J* = 6.0 Hz, 1H), 6.08 (d, *J* = 14.0 Hz, 1H), 4.79* (br, 1H), 4.51* (d, *J* = 11.2 Hz, 1H), 4.29 (d, *J* = 14.0 Hz, 1H); ^{13}C NMR (101 MHz, $CDCl_3$) δ 170.2, 143.6, 140.0, 137.0, 133.8, 133.3, 131.5, 130.4, 129.8, 129.3, 129.2, 128.6, 128.3, 127.9, 126.8, 126.1, 125.4, 124.4, 123.9, 99.6, 51.8. IR (film): v_{max} (cm^{-1}) = 3060, 1645, 1576, 1503, 1467, 1440, 1403, 1374, 1323, 1292, 1252, 1203, 1152, 1081, 1007, 949, 801, 776, 752, 718, 702, 658, 634, 610, 582, 550, 496, 450, 434. HRMS (ESI): Exact mass calcd. for $C_{24}H_{19}NOI$ ([M+H]$^+$): 464.05058. Found: 464.04959.

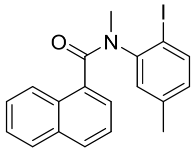

1d: white solid, 2.9 g, 72% yield (from **A** over 2 steps, 10 mmol scale), m.p. = 128-129 °C. Two sets of signals were observed due to the existence of rotamers. The peaks of the minor one are marked with asterisk. ^{1}H NMR (400 MHz, $CDCl_3$) δ 8.18 (d, *J* = 8.4 Hz, 1H), 7.92-7.89* (m, 2H), 7.83* (d, *J* = 8.0 Hz, 1H), 7.72 (d, *J* = 8.4 Hz, 1H), 7.64 (d, *J* = 8.0 Hz, 1H), 7.57-7.52 (m, 2H), 7.47-7.43 (m, 2H), 7.31* (s, 1H), 7.20-7.16 (m, 1H), 6.92* (dd, *J* = 8.0, 1.6 Hz, 1H), 6.80 (d, *J* = 1.6 Hz, 1H), 6.52 (dd, *J* = 8.0, 1.6 Hz, 1H), 3.49 (s, 3H), 3.06* (s, 3H), 2.40* (s, 3H), 1.85 (s, 3H); ^{13}C NMR (101 MHz, $CDCl_3$) δ 170.7, 146.0, 145.3, 140.4, 139.8, 139.6, 139.4, 134.3, 134.1,

133.7, 133.3, 130.8, 130.4, 130.22, 130.16, 129.8, 129.7, 129.5, 129.4, 128.5, 128.3, 127.2, 126.7, 126.6, 126.1, 125.8, 125.2, 124.4, 124.32, 124.30, 94.5, 40.0, 36.9, 21.1, 20.4. IR (film): v_{max} (cm^{-1}) = 1649, 1506, 1468, 1358, 1306, 1252, 1197, 1120, 1011, 858, 829, 797, 787, 775, 740, 718, 684, 645, 571, 494, 466, 432. HRMS (ESI): Exact mass calcd. for $C_{19}H_{17}NOI$ ($[M+H]^+$): 402.03493. Found: 402.03501.

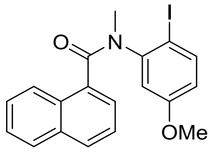

1e: white solid, 0.70 g, 42% yield (from **A** over 2 steps, 4 mmol scale), m.p. = 106-107 °C. Two sets of signals were observed due to the existence of rotamers. The peaks of the minor one are marked with asterisk. ^{1}H NMR (400 MHz, $CDCl_3$) δ 8.17 (d, *J* = 8.4 Hz, 1H), 7.93-7.90* (m, 2H), 7.83* (d, *J* = 8.8 Hz, 1H), 7.75 (d, *J* = 8.0 Hz, 1H), 7.66 (d, *J* = 8.4 Hz, 1H), 7.58-7.51 (m, 2H), 7.47-7.44 (m, 2H), 7.20 (dd, *J* = 8.0, 7.2 Hz, 1H), 7.04* (d, *J* = 2.8 Hz, 1H), 6.72* (dd, *J* = 8.8, 2.8 Hz, 1H), 6.47 (d, *J* = 2.8 Hz, 1H), 6.32 (dd, *J* = 8.8, 2.8 Hz, 1H), 3.86* (s, 3H), 3.51 (s, 3H), 3.20 (s, 3H), 3.07* (s, 3H); ^{13}C NMR (101 MHz, $CDCl_3$) δ 170.7, 161.3, 160.1, 147.0, 140.4, 140.0, 134.2, 133.3, 130.5, 129.6, 129.4, 128.6, 128.5, 127.2, 126.8, 126.6, 126.2, 125.6, 125.2, 124.6, 124.4, 124.1, 116.6, 115.2, 114.5, 86.7, 55.8, 55.2, 39.9, 36.8. IR (film): v_{max} (cm^{-1}) = 3002, 1650, 1568, 1506, 1461, 1440, 1401, 1359, 1320, 1253, 1230, 1183, 1108, 1038, 1007, 991, 919, 849, 832, 789, 774, 737, 711, 646, 599, 576, 500, 477, 430. HRMS (ESI): Exact mass calcd. for $C_{19}H_{17}NO_2I$ ($[M+H]^+$): 418.02985. Found: 418.02940.

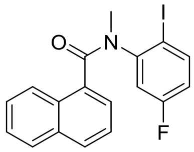

1f: white solid, 2.7 g, 67% yield (from **A** over 2 steps, 10 mmol scale), m.p. = 119-121 °C. Two sets of signals were observed due to the existence of rotamers. The peaks of the minor one are marked with asterisk. ^{1}H NMR (400 MHz, $CDCl_3$) δ 8.16 (d, *J* = 8.4 Hz, 1H), 7.95-7.91 (m, 1H), 7.76 (d, *J* = 8.4 Hz, 1H), 7.69 (d, *J* = 8.0 Hz,

1H), 7.65 (dd, J = 8.8, 6.0 Hz, 1H), 7.59-7.54 (m, 1H), 7.49* (d, J = 7.2 Hz, 2H), 7.46-7.43 (m, 1H), 7.22-7.18 (m, 1H), 6.93-6.88* (m, 1H), 6.72 (dd, J = 8.8, 2.8 Hz, 1H), 6.56-6.51 (m, 1H), 3.50 (s, 3H), 3.08* (s, 3H); ^{13}C NMR (101 MHz, $CDCl_3$) δ 170.4, 162.6 (d, J = 251.9 Hz), 147.7 (d, J = 9.4 Hz), 140.8 (d, J = 8.6 Hz), 133.7, 133.5, 130.4, 129.8, 128.6, 128.5, 127.3, 127.1, 126.7, 126.4, 125.6, 125.2, 124.44, 124.40, 117.0 (d, J = 22.7 Hz), 116.9 (d, J = 21.6 Hz), 92.2 (d, J = 3.8 Hz), 39.8, 36.9; ^{19}F NMR (377 MHz, $CDCl_3$) δ -111.4 (s), -111.7 (m). IR (film): ν_{max} (cm^{-1}) = 3057, 1647, 1573, 1505, 1462, 1405, 1351, 1303, 1246, 1195, 1106, 1018, 994, 865, 825, 810, 784, 743, 714, 643, 578, 514, 471, 432. HRMS (ESI): Exact mass calcd. for $C_{18}H_{14}NOFI$ ([M+H]$^+$): 406.00986. Found: 406.00938.

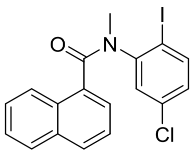

1g: white solid, 2.4 g, 57% yield (from **A** over 2 steps, 10 mmol scale), m.p. = 109-111 °C. Two sets of signals were observed due to the existence of rotamers. The peaks of the minor one are marked with asterisk. ^{1}H NMR (400 MHz, $CDCl_3$) δ 8.16 (d, J = 8.4 Hz, 1H), 7.95-7.89 (m, 1H), 7.76 (d, J = 8.4 Hz, 1H), 7.69 (d, J = 8.4 Hz, 1H), 7.63-7.56 (m, 2H), 7.50-7.48 (m, 1H), 7.45* (s, 1H), 7.43* (s, 1H), 7.23-7.19 (m, 1H), 7.12* (dd, J = 8.4, 2.4 Hz, 1H), 7.00 (d, J = 2.4 Hz, 1H), 6.73 (dd, J = 8.4, 2.4 Hz, 1H), 3.49 (s, 3H), 3.08* (s, 3H); ^{13}C NMR (101 MHz, $CDCl_3$) δ 170.4, 147.4, 140.9, 140.7, 135.7, 134.7, 133.7, 133.5, 133.4, 130.3, 130.0, 129.84, 129.77, 129.7, 129.5, 128.6, 128.4, 127.4, 127.0, 126.7, 126.4, 125.6, 125.2, 124.45, 124.40, 124.38, 96.3, 39.8, 36.9. IR (film): ν_{max} (cm^{-1}) = 3055, 1647, 1567, 1505, 1458, 1389, 1347, 1294, 1247, 1168, 1118, 1093, 1015, 984, 896, 873, 826, 803, 781, 736, 715, 676, 642, 576, 525, 470, 455, 441, 424. HRMS (ESI): Exact mass calcd. for $C_{18}H_{14}NOClI$ ([M+H]$^+$): 421.98031. Found: 421.97919.

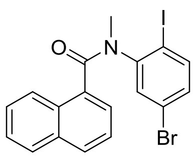

1h: white solid, 1.5 g, 32% yield (from **A** over 2 steps, 10 mmol scale), m.p. = 119-121 °C. Two sets of signals were observed due to the existence of rotamers. The peaks of the minor one are marked with asterisk. ^{1}H NMR (400 MHz, $CDCl_3$) δ 8.15 (d, *J* = 8.4 Hz, 1H), 7.93 (t, *J* = 8.4 Hz, 1H), 7.84* (d, *J* = 8.4 Hz, 1H), 7.76 (d, *J* = 8.4 Hz, 1H), 7.70 (d, *J* = 8.0 Hz, 1H), 7.63* (d, *J* = 2.0 Hz, 1H), 7.61-7.60* (m, 1H), 7.58-7.56 (m, 1H), 7.53 (d, *J* = 8.4 Hz, 1H), 7.50-7.47 (m, 1H), 7.45* (s, 1H), 7.43* (s, 1H), 7.23-7.19 (m, 1H), 7.15 (d, *J* = 2.0 Hz, 1H), 6.86 (dd, *J* = 8.4, 2.4 Hz, 1H), 3.49 (s, 3H), 3.08* (s, 3H); ^{13}C NMR (101 MHz, $CDCl_3$) δ 170.5, 147.5, 141.2, 141.0, 133.5, 133.4, 132.9, 132.6, 132.4, 132.3, 130.2, 129.8, 128.7, 128.5, 127.4, 127.0, 126.7, 126.4, 125.6, 125.2, 124.41, 124.39, 123.3, 122.3, 97.2, 39.9, 36.9. IR (film): ν_{max} (cm^{-1}) = 3070, 1653, 1562, 1506, 1456, 1382, 1350, 1293, 1263, 1117, 1081, 1009, 983, 911, 894, 875, 859, 826, 796, 773, 755, 727, 712, 643, 569, 510, 465, 437. HRMS (ESI): Exact mass calcd. for $C_{18}H_{14}NOBrI$ ($[M+H]^+$): 465.92980. Found: 465.92904.

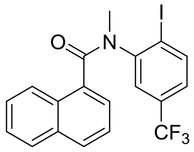

1i: white solid, 1.2 g, 75% yield (from **A** over 2 steps, 3.5 mmol scale), m.p. = 107-108 °C. Two sets of signals were observed due to the existence of rotamers. The peaks of the minor one are marked with asterisk. ^{1}H NMR (400 MHz, $CDCl_3$) δ 8.13 (d, *J* = 8.4 Hz, 1H), 7.96-7.91 (m, 1H), 7.80 (d, *J* = 8.4 Hz, 1H), 7.74-7.72 (m, 1H), 7.66 (d, *J* = 8.4 Hz, 1H), 7.60-7.55 (m, 1H), 7.48-7.42 (m, 1H), 7.36* (d, *J* = 7.2 Hz, 1H), 7.21 (s, 1H), 7.20 -7.16 (m, 1H), 6.93 (dd, *J* = 8.0, 1.2 Hz, 1H), 3.53 (s, 3H), 3.11* (s, 3H); ^{13}C NMR (101 MHz, $CDCl_3$) δ 170.6, 146.9, 146.4, 141.0, 140.8, 133.7, 133.5, 133.3, 131.4 (q, *J* = 33.6 Hz), 130.1, 129.9, 129.8, 128.7, 128.4, 127.4, 127.1, 126.8, 126.5, 126.2 (q, *J* = 3.4 Hz), 126.0, 125.5 (q, *J* = 3.7 Hz), 125.3, 125.2, 124.5,

124.3, 124.2, 103.4, 103.3, 39.8, 36.7; ^{19}F NMR (377 MHz, $CDCl_3$) δ -62.7 (s), -63.6 (s). IR (film): v_{max} (cm^{-1}) = 3067, 1656, 1594, 1469, 1406, 1330, 1285, 1258, 1174, 1115, 1078, 1013, 986, 915, 892, 860, 841, 798, 776, 744, 723, 708, 643, 578, 509, 475, 436. HRMS (ESI): Exact mass calcd. for $C_{19}H_{14}NOF_3I$ ($[M+H]^+$): 456.00667. Found: 456.00602.

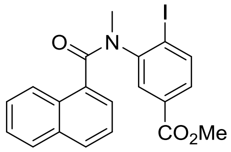

1j: white solid, 2.1 g, 47% yield (from **A** over 2 steps, 10 mmol scale), m.p. = 124-126 °C. Two sets of signals were observed due to the existence of rotamers. The peaks of the minor one are marked with asterisk. ^{1}H NMR (400 MHz, $CDCl_3$) δ 8.20 (d, *J* = 8.4 Hz, 1H), 8.12* (d, *J* = 2.0 Hz, 1H), 8.08* (d, *J* = 8.4 Hz, 1H), 7.92 (t, *J* = 8.4 Hz, 1H), 7.78-7.70 (m, 2H), 7.66-7.54 (m, 3H), 7.47-7.43 (m, 1H), 7.34 (dd, *J* = 8.0, 2.0 Hz, 1H), 7.19-7.15 (m, 1H), 3.96* (s, 3H), 3.68 (s, 3H), 3.52 (s, 3H), 3.10* (s, 3H); ^{13}C NMR (101 MHz, $CDCl_3$) δ 170.6, 166.0, 165.3, 146.6, 146.0, 140.5, 140.3, 133.8, 133.69, 133.65, 133.3, 132.3, 131.2, 130.2, 130.1, 130.0, 129.8, 129.7, 129.6, 128.6, 128.3, 127.3, 126.9, 126.7, 126.3, 125.8, 125.2, 124.4, 124.3, 105.3, 52.6, 52.3, 39.8, 36.8. IR (film): v_{max} (cm^{-1}) = 2948, 1722, 1644, 1584, 1560, 1506, 1466, 1431, 1392, 1335, 1306, 1275, 1241, 1114, 1097, 1015, 993, 964, 897, 842, 804, 775, 755, 669, 647, 593, 551, 509, 475, 427. HRMS (ESI): Exact mass calcd. for $C_{20}H_{17}NO_3I$ ($[M+H]^+$): 446.02476. Found: 446.02361.

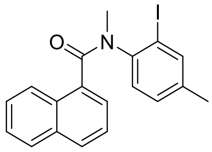

1k: white solid, 1.8 g, 45% yield (from **A** over 2 steps, 10 mmol scale), m.p. = 116-118 °C. Two sets of signals were observed due to the existence of rotamers. The peaks of the minor one are marked with asterisk. ^{1}H NMR (400 MHz, $CDCl_3$) δ 8.18 (d, *J* = 8.4 Hz, 1H), 7.92-7.88* (m, 2H), 7.81* (s, 1H), 7.73 (d, *J* = 8.0 Hz, 1H), 7.65

(d, J = 8.4 Hz, 1H), 7.58-7.52 (m, 2H), 7.47-7.43 (m, 2H), 7.36* (d, J = 7.6 Hz, 1H), 7.29* (dd, J = 8.0, 1.2 Hz, 1H), 7.20-7.16 (m, 1H), 6.82 (d, J = 8.0 Hz, 1H), 6.66 (dd, J = 8.0, 1.2 Hz, 1H), 3.49 (s, 3H), 3.06* (s, 3H), 2.36* (s, 3H), 2.07 (s, 3H); ^{13}C NMR (101 MHz, $CDCl_3$) δ 170.7, 143.7, 140.6, 140.4, 140.0, 139.4, 134.1, 133.7, 133.4, 130.8, 130.4, 129.9, 129.5, 129.3, 128.7, 128.53, 128.46, 128.3, 127.1, 126.8, 126.6, 126.1, 125.8, 125.2, 124.5, 124.34, 124.28, 98.5, 40.0, 37.1, 20.7, 20.4. IR (film): v_{max} (cm^{-1}) = 2919, 1638, 1484, 1415, 1351, 1300, 1257, 1122, 1057, 1034, 975, 901, 864, 826, 804, 775, 755, 664, 639, 613, 583, 561, 512, 492, 468, 445. HRMS (ESI): Exact mass calcd. for $C_{19}H_{17}NOI$ ($[M+H]^+$): 402.03493. Found: 402.03408.

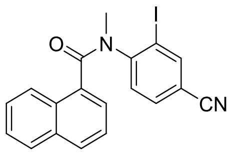

1l: white solid, 2.0 g, 49% yield (from **A** over 2 steps, 10 mmol scale), m.p. = 120-121 °C. Two sets of signals were observed due to the existence of rotamers. The peaks of the minor one are marked with asterisk. ^{1}H NMR (400 MHz, $CDCl_3$) δ 8.28* (s, 1H), 8.16 (d, J = 8.4 Hz, 1H), 8.02* (d, J = 1.2 Hz, 2H), 7.97-7.92* (m, 2H), 7.81-7.77 (m, 1H), 7.72 (d, J = 8.0 Hz, 1H), 7.58 (t, J = 7.6 Hz, 2H), 7.50 (t, J = 7.6 Hz, 1H), 7.37 (d, J = 7.2 Hz, 1H), 7.22-7.16 (m, 2H), 6.99 (d, J = 8.4 Hz, 1H), 3.51 (s, 3H), 3.10* (s, 3H); ^{13}C NMR (101 MHz, $CDCl_3$) δ 170.1, 150.8, 143.5, 133.6, 133.5, 133.2, 132.7, 130.3, 130.2, 130.1, 129.9, 129.7, 128.7, 128.6, 127.4, 127.3, 126.8, 126.6, 125.4, 125.2, 124.54, 124.47, 116.2, 113.0, 98.9, 39.6, 36.8. IR (film): v_{max} (cm^{-1}) = 3054, 2231, 1643, 1583, 1478, 1416, 1355, 1305, 1260, 1118, 1052, 1033, 980, 890, 865, 845, 806, 777, 735, 671, 648, 603, 585, 503, 470, 443. HRMS (ESI): Exact mass calcd. for $C_{19}H_{14}N_2OI$ ($[M+H]^+$): 413.01453. Found: 413.01337.

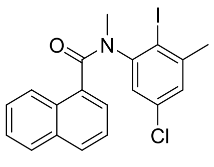

1m: white solid, 1.2 g, 75% yield (from **A** over 2 steps, 3.7 mmol scale), m.p. = 168-170 °C. Two sets of signals were observed due to the existence of rotamers. The peaks of the minor one are marked with asterisk. ^{1}H NMR (400 MHz, $CDCl_3$) δ 8.16 (d, *J* = 8.4 Hz, 1H), 7.91 (t, *J* = 8.0 Hz, 1H), 7.74 (d, *J* = 8.4 Hz, 1H), 7.66 (d, *J* = 8.4 Hz, 1H), 7.61-7.52 (m, 2H), 7.46 (t, *J* = 6.8 Hz, 1H), 7.31* (d, *J* = 2.0 Hz, 1H), 7.26* (d, *J* = 2.4 Hz, 1H), 7.20-7.16 (m, 1H), 6.88-6.84 (m, 1H), 3.48 (s, 3H), 3.06* (s, 3H), 2.54* (s, 3H), 2.33 (s, 3H); ^{13}C NMR (101 MHz, $CDCl_3$) δ 170.3, 147.6, 146.9, 145.6, 145.2, 135.0, 133.9, 133.8, 133.7, 133.6, 133.4, 130.3, 129.8, 129.7, 129.2, 128.62, 128.59, 128.4, 127.3, 126.9, 126.7, 126.6, 126.3, 126.2, 125.7, 125.2, 124.31, 124.28, 124.1, 103.8, 39.8, 37.0, 29.3, 29.2. IR (film): v_{max} (cm^{-1}) = 2915, 1639, 1555, 1505, 1429, 1406, 1362, 1306, 1264, 1170, 1150, 1112, 1085, 1057, 1020, 906, 868, 803, 775, 721, 647, 574, 558, 488, 467, 452, 415. HRMS (ESI): Exact mass calcd. for $C_{19}H_{16}NOClI$ ($[M+H]^+$): 435.99596. Found: 435.99541.

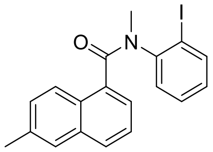

1n: white solid, 1.0 g, 50% yield (from **A** over 2 steps, 5.0 mmol scale), m.p. = 125-127 °C. Two sets of signals were observed due to the existence of rotamers. The peaks of the minor one are marked with asterisk. ^{1}H NMR (400 MHz, $CDCl_3$) δ 8.09 (d, *J* = 8.8 Hz, 1H), 7.98* (d, *J* = 7.6 Hz, 1H), 7.83* (d, *J* = 8.0 Hz, 1H), 7.72 (d, *J* = 8.0 Hz, 1H), 7.56 (d, *J* = 8.4 Hz, 1H), 7.50 (s, 1H), 7.44-7.34 (m, 2H), 7.12 (t, *J* = 7.6 Hz, 1H), 6.95-6.87 (m, 2H), 6.73 (t, *J* = 7.6 Hz, 1H), 3.51 (s, 3H), 3.08* (s, 3H), 2.54* (s, 3H), 2.49 (s, 3H); ^{13}C NMR (101 MHz, $CDCl_3$) δ 170.8, 146.4, 140.2, 140.1, 136.4, 135.9, 134.0, 133.7, 133.6, 130.0, 129.7, 129.5, 129.3, 129.1, 128.9, 128.8, 128.7, 127.5, 127.3, 125.6, 125.3, 124.5, 123.6, 123.5, 98.8, 39.9, 37.0, 21.8, 21.7. IR (film): v_{max} (cm^{-1}) = 2940, 1645, 1467, 1411, 1373, 1350, 1302, 1250, 1160, 1111, 1050, 1017, 981, 877, 823, 801, 772, 752, 722, 697, 647, 602, 558, 495, 457, 436. HRMS (ESI): Exact mass calcd. for $C_{19}H_{17}NOI$ ($[M+H]^+$): 402.03493. Found: 402.03517.

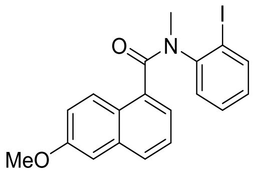

1o: white solid, 1.0 g, 48% yield (from **A** over 2 steps, 5.0 mmol scale), m.p. = 106-108 °C. Two sets of signals were observed due to the existence of rotamers. The peaks of the minor one are marked with asterisk. ^{1}H NMR (400 MHz, $CDCl_3$) δ 8.10 (d, *J* = 9.2 Hz, 1H), 7.96* (d, *J* = 7.6 Hz, 1H), 7.79* (d, *J* = 8.0 Hz, 1H), 7.70 (d, *J* = 7.6 Hz, 1H), 7.55-7.51 (m, 1H), 7.28 (d, *J* = 6.8 Hz, 1H), 7.23-7.20 (m, 1H), 7.18* (d, *J* = 2.4 Hz, 1H), 7.13-7.09 (m, 1H), 7.01 (d, *J* = 2.4 Hz, 1H), 6.94-6.87 (m, 2H), 6.73-6.69 (m, 1H), 3.91* (s, 3H), 3.86 (s, 3H), 3.49 (s, 3H), 3.07* (s, 3H); ^{13}C NMR (101 MHz, $CDCl_3$) δ 170.6, 158.0, 157.6, 146.3, 140.1, 140.0, 135.1, 134.7, 133.7, 130.0, 129.6, 129.3, 129.10, 129.06, 129.02, 128.3, 128.2, 127.3, 125.82, 125.76, 125.0, 122.2, 121.9, 119.9, 119.5, 106.4, 106.1, 98.7, 55.4, 55.3, 39.9, 36.9. IR (film): v_{max} (cm^{-1}) = 2931, 1628, 1511, 1467, 1430, 1376, 1356, 1302, 1267, 1249, 1217, 1170, 1112, 1059, 1021, 980, 937, 844, 820, 784, 772, 749, 723, 658, 618, 559, 509, 482, 456, 430. HRMS (ESI): Exact mass calcd. for $C_{19}H_{17}NO_2I$ ([M+H]$^+$): 418.02985. Found: 418.02983.

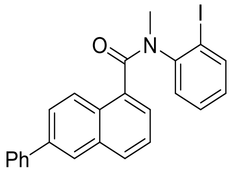

1p: white solid, 0.75 g, 32% yield (from **A** over 2 steps, 5.0 mmol scale), m.p. = 129-131 °C. Two sets of signals were observed due to the existence of rotamers. The peaks of the minor one are marked with asterisk. ^{1}H NMR (400 MHz, $CDCl_3$) δ 8.28 (d, *J* = 8.8 Hz, 1H), 8.09* (s, 1H), 7.97* (t, *J* = 7.6 Hz, 2H), 7.92 (s, 1H), 7.84-7.82 (m, 1H), 7.73-7.68 (m, 4H), 7.50-7.35 (m, 4H), 7.18 (t, *J* = 7.6 Hz, 1H), 7.10-7.08* (m, 1H), 6.98 (d, *J* = 7.6 Hz, 1H), 6.90 (t, *J* = 7.6 Hz, 1H), 6.72 (t, *J* = 7.6 Hz, 1H), 3.52 (s, 3H), 3.10* (s, 3H); ^{13}C NMR (101 MHz, $CDCl_3$) δ 170.5, 146.3, 140.7, 140.2,

140.1, 139.3, 138.7, 134.0, 133.7, 130.0, 129.8, 129.73, 129.66, 129.3, 129.20, 129.19, 129.02, 128.98, 127.7, 127.6, 127.5, 127.4, 126.9, 126.5, 126.4, 126.3, 126.1, 125.6, 124.9, 124.5, 124.3, 98.8, 40.0, 37.0. IR (film): v_{max} (cm^{-1}) = 2975, 1637, 1473, 1441, 1370, 1348, 1308, 1251, 1132, 1020, 981, 886, 833, 809, 795, 772, 758, 721, 695, 659, 636, 620, 558, 500, 449, 420. HRMS (ESI): Exact mass calcd. for $C_{24}H_{19}NOI$ ($[M+H]^+$): 464.05058. Found: 464.05028.

General procedure for Pd-catalyzed dearomative reaction of naphthalenes with malonate diesters (sodium dimethyl malonate as an example)

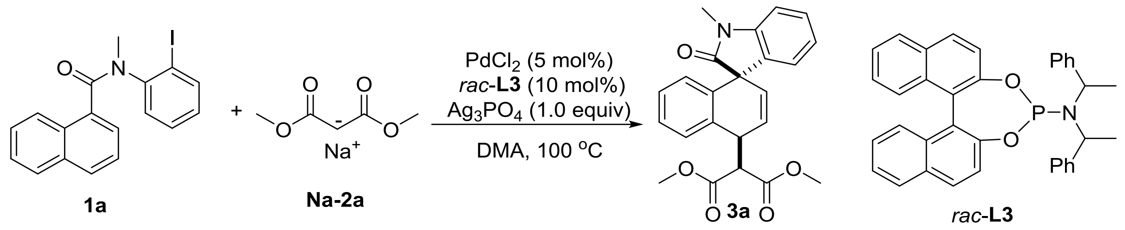

A flame-dried sealed tube was cooled to room temperature under argon. To this tube were added NaH (9.6 mg, 0.4 mmol), dimethyl malonate (52.8 mg, 0.4 mmol) and DMA (1.0 mL). Then the reaction mixture was stirred at room temperature for 0.5 h followed by the addition of **1a** (77.4 mg, 0.2 mmol), $PdCl_2$ (1.8 mg, 0.01 mmol), *rac*-**L3** (10.8 mg, 0.02 mmol) and Ag_3PO_4 (83.7 mg, 0.2 mmol). Then the reaction mixture was stirred at 100 °C. After completion (monitored by TLC), the reaction mixture was cooled to room temperature and diluted with ethyl acetate (3 mL). The mixture was filtered through celite, and the filtrate was concentrated under reduced pressure. The crude product was purified by silica gel column chromatography (PE/EtOAc = 20/1 – 10:1) to afford the desired product **3a**.

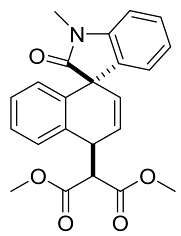

3a: white solid, 74.1 mg, 95% yield, m.p. = 150-152 °C. ^{1}H NMR (400 MHz, $CDCl_3$) δ 7.39-7.34 (m, 1H), 7.27-7.25 (m, 1H), 7.19-7.15 (m, 1H), 7.09-7.02 (m, 2H), 6.97-6.94 (m, 2H), 6.56-6.50 (m, 2H), 5.71 (d, *J* = 9.6 Hz, 1H), 4.51 (d, *J* = 10.4 Hz, 1H), 4.39 (dd, *J* = 10.4, 5.2 Hz, 1H), 3.77 (s, 3H), 3.61 (s, 3H), 3.25 (s, 3H); ^{13}C NMR (101 MHz, $CDCl_3$) δ 176.6, 169.5, 168.7, 144.1, 136.1, 135.6, 135.0, 130.9, 129.4, 128.9, 128.5, 127.5, 127.4, 127.3, 124.9, 123.6, 108.2, 62.5, 55.7, 52.6, 52.4, 40.4, 27.0. IR (film): v_{max} (cm^{-1}) = 2956, 1749, 1712, 1611, 1490, 1469, 1434, 1371, 1337, 1314, 1262, 1156, 1125, 1073, 1019, 978, 960, 904, 826, 777, 745, 725, 690, 665, 637,

561, 542, 493, 477, 446. HRMS (ESI): Exact mass calcd. for $C_{23}H_{21}NO_5Na$ ([M+Na]$^+$): 414.13119. Found: 414.13112.

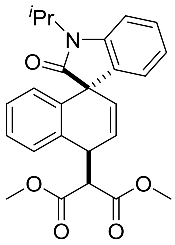

3b: white solid, 69.2 mg, 82% yield, m.p. = 160-162 °C. ^{1}H NMR (400 MHz, $CDCl_3$) δ 7.33-7.29 (m, 1H), 7.26-7.24 (m, 1H), 7.19-7.12 (m, 2H), 7.07-7.01 (m, 2H), 6.93 (dd, *J* = 7.2, 0.8 Hz, 1H), 6.52 (dd, *J* = 9.6, 4.8 Hz, 1H), 6.49 (dd, *J* = 8.0, 0.8 Hz, 1H), 5.69 (d, *J* = 10.0 Hz, 1H), 4.74-4.64 (m, 1H), 4.43 (d, *J* = 10.4 Hz, 1H), 4.38 (dd, *J* = 10.4, 4.8 Hz, 1H), 3.77 (s, 3H), 3.62 (s, 3H), 1.50 (dd, *J* = 7.2, 3.2 Hz, 6H); ^{13}C NMR (101 MHz, $CDCl_3$) δ 176.0, 169.6, 168.7, 142.6, 136.4, 135.7, 135.5, 130.5, 129.4, 128.46, 128.45, 127.4, 127.33, 127.29, 125.3, 122.9, 110.1, 62.5, 55.4, 52.7, 52.5, 44.1, 40.3, 19.5. IR (film): ν_{max} (cm^{-1}) = 2977, 1750, 1706, 1606, 1483, 1466, 1430, 1340, 1310, 1252, 1220, 1191, 1146, 1099, 1064, 963, 797, 774, 749, 720, 693, 572, 495, 451. HRMS (ESI): Exact mass calcd. for $C_{25}H_{25}NO_5Na$ ([M+Na]$^+$): 442.16249. Found: 442.16178.

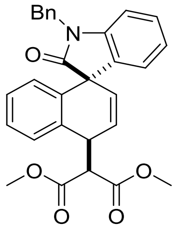

3c: white solid, 80.3 mg, 86% yield, m.p. = 148-149 °C. ^{1}H NMR (400 MHz, $CDCl_3$) δ 7.33-7.25 (m, 6H), 7.23-7.18 (m, 2H), 7.09-7.05 (m, 1H), 7.04-7.00 (m, 1H), 6.96-6.94 (m, 1H), 6.79 (d, *J* = 8.0 Hz, 1H), 6.60-6.54 (m, 2H), 5.76 (d, *J* = 10.0 Hz, 1H), 5.00 (AB, J_{AB} = 15.6 Hz, 1H), 4.88 (BA, J_{BA} = 15.6 Hz, 1H), 4.50 (d, *J* = 10.4 Hz, 1H), 4.42 (dd, *J* = 10.4, 4.8 Hz, 1H), 3.77 (s, 3H), 3.62 (s, 3H); ^{13}C NMR (101 MHz, $CDCl_3$) δ 176.6, 169.5, 168.7, 143.1, 136.2, 135.8, 135.6, 135.1, 131.0, 129.3, 128.9, 128.7, 128.6, 127.7, 127.51, 127.47, 127.38, 127.2, 125.0, 123.5, 109.4, 62.5, 55.6,

52.7, 52.5, 44.3, 40.3. IR (film): v_{max} (cm^{-1}) = 2950, 1745, 1714, 1607, 1484, 1462, 1436, 1318, 1260, 1195, 1172, 1149, 1114, 961, 934, 905, 815, 776, 759, 731, 693, 664, 627, 573, 551, 497, 477, 456. HRMS (ESI): Exact mass calcd. for $C_{29}H_{25}NO_5Na$ ($[M+Na]^+$): 490.16249. Found: 490.16151.

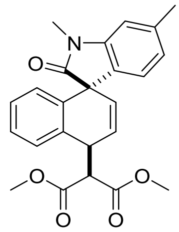

3d: white foam, 75.6 mg, 93% yield. ^1H NMR (400 MHz, $CDCl_3$) δ 7.26-7.24 (m, 1H), 7.18-7.14 (m, 1H), 7.06-7.02 (m, 1H), 6.89 (d, *J* = 7.6 Hz, 1H), 6.83 (d, *J* = 7.6 Hz, 1H), 6.77 (s, 1H), 6.54-6.50 (m, 2H), 5.69 (d, *J* = 10.0 Hz, 1H), 4.51 (d, *J* = 10.4 Hz, 1H), 4.38 (dd, *J* = 10.8, 5.2 Hz, 1H), 3.76 (s, 3H), 3.60 (s, 3H), 3.23 (s, 3H), 2.43 (s, 3H); ^{13}C NMR (101 MHz, $CDCl_3$) δ 176.8, 169.5, 168.7, 144.1, 139.0, 136.3, 135.6, 132.1, 130.7, 129.7, 128.4, 127.5, 127.3, 127.2, 124.6, 124.0, 109.1, 62.5, 55.5, 52.6, 52.4, 40.5, 26.9, 21.9. IR (film): v_{max} (cm^{-1}) = 2957, 1751, 1712, 1617, 1440, 1364, 1311, 1260, 1152, 1076, 965, 901, 830, 809, 775, 747, 724, 698, 682, 650, 622, 592, 556, 541, 499, 479, 444. HRMS (ESI): Exact mass calcd. for $C_{24}H_{23}NO_5Na$ ($[M+Na]^+$): 428.14684. Found: 428.14697.

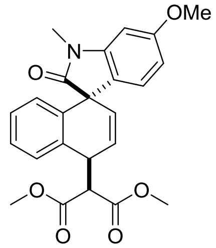

3e: white foam, 83.1 mg, 98% yield. ^1H NMR (400 MHz, $CDCl_3$) δ 7.25 (d, *J* = 7.2 Hz, 1H), 7.19-7.15 (m, 1H), 7.07-7.03 (m, 1H), 6.85 (d, *J* = 8.0 Hz, 1H), 6.59-6.49 (m, 4H), 5.70 (d, *J* = 9.6 Hz, 1H), 4.49 (d, *J* = 10.8 Hz, 1H), 4.37 (dd, *J* = 10.4, 5.2 Hz, 1H), 3.86 (s, 3H), 3.76 (s, 3H), 3.60 (s, 3H), 3.22 (s, 3H); ^{13}C NMR (101 MHz, $CDCl_3$) δ 177.1, 169.5, 168.7, 160.7, 145.3, 136.5, 135.6, 130.6, 129.9, 128.4, 127.5, 127.3, 127.2, 127.0, 125.5, 107.2, 96.2, 62.5, 55.6, 55.2, 52.6, 52.4, 40.4, 27.0. IR (film):

v_{max} (cm^{-1}) = 2931, 1747, 1712, 1622, 1503, 1436, 1366, 1319, 1258, 1148, 1079, 1032, 990, 965, 904, 857, 834, 775, 753, 722, 696, 651, 629, 582, 501, 476, 451, 420. HRMS (ESI): Exact mass calcd. for $C_{24}H_{23}NO_6Na$ ($[M+Na]^+$): 444.14176. Found: 444.14134.

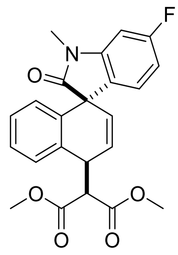

3f: white solid, 67.6 mg, 83% yield, m.p. = 148-150 °C. ^1H NMR (400 MHz, $CDCl_3$) δ 7.27-7.25 (m, 1H), 7.21-7.17 (m, 1H), 7.09-7.05 (m, 1H), 6.92-6.89 (m, 1H), 6.78-6.73 (m, 1H), 6.69 (dd, *J* = 8.8, 2.4 Hz, 1H), 6.56-6.49 (m, 2H), 5.68 (d, *J* = 10.0 Hz, 1H), 4.45 (d, *J* = 10.4 Hz, 1H), 4.38 (dd, *J* = 10.4, 5.2 Hz, 1H), 3.77 (s, 3H), 3.61 (s, 3H), 3.23 (s, 3H); ^{13}C NMR (101 MHz, $CDCl_3$) δ 176.9, 169.5, 168.7, 163.5 (d, *J* = 246.9 Hz), 145.6 (d, *J* = 11.5 Hz), 135.9, 135.7, 131.2, 130.39 (d, *J* = 3.1 Hz), 129.2, 128.7, 127.6, 127.5, 127.4, 126.1 (d, *J* = 9.7 Hz), 109.7 (d, *J* = 22.4 Hz), 97.1 (d, *J* = 27.8 Hz), 62.5, 55.2, 52.7, 52.5, 40.4, 27.2; ^{19}F NMR (377 MHz, $CDCl_3$) δ -111.2 (m). IR (film): v_{max} (cm^{-1}) = 3073, 1750, 1713, 1611, 1503, 1437, 1367, 1323, 1259, 1190, 1145, 1117, 1079, 974, 905, 867, 812, 768, 750, 719, 697, 652, 614, 542, 502, 479, 443. HRMS (ESI): Exact mass calcd. for $C_{23}H_{20}NO_5FNa$ ($[M+Na]^+$): 432.12177. Found: 432.12122.

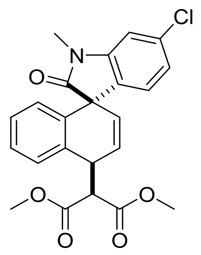

3g: white foam, 70.7 mg, 83% yield. ^1H NMR (400 MHz, $CDCl_3$) δ 7.28-7.26 (m, 1H), 7.21-7.17 (m, 1H), 7.08-7.04 (m, 2H), 6.95 (d, *J* = 2.0 Hz, 1H), 6.88 (d, *J* = 8.0 Hz, 1H), 6.55 (dd, *J* = 9.6, 4.8 Hz, 1H), 6.50 (d, *J* = 7.6 Hz, 1H), 5.67 (d, *J* = 10.0 Hz, 1H),

4.45 (d, *J* = 10.4 Hz, 1H), 4.38 (dd, *J* = 10.4, 5.2 Hz, 1H), 3.77 (s, 3H), 3.61 (s, 3H), 3.23 (s, 3H); ^{13}C NMR (101 MHz, $CDCl_3$) δ 176.6, 169.5, 168.7, 145.3, 135.7, 135.6, 134.7, 133.4, 131.4, 128.9, 128.7, 127.7, 127.4, 125.9, 123.5, 109.0, 62.5, 55.3, 52.7, 52.5, 40.4, 27.1. IR (film): v_{max} (cm^{-1}) = 2922, 1753, 1723, 1609, 1492, 1438, 1362, 1311, 1264, 1174, 1152, 1070, 981, 960, 902, 833, 811, 776, 742, 695, 678, 645, 621, 596, 556, 518, 499, 479, 449. HRMS (ESI): Exact mass calcd. for $C_{23}H_{20}NO_5NaCl$ ($[M+Na]^+$): 448.09222. Found: 448.09094.

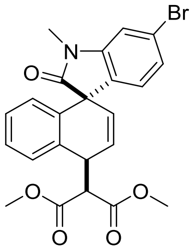

3h: white solid, 70.4 mg, 75% yield, m.p. = 180-182 °C. ^{1}H NMR (400 MHz, $CDCl_3$) δ 7.28-7.25 (m, 1H), 7.23-7.17 (m, 2H), 7.10 (d, *J* = 1.6 Hz, 1H), 7.09-7.05 (m, 1H), 6.83 (d, *J* = 7.6 Hz, 1H), 6.55 (dd, *J* = 9.6, 5.2 Hz, 1H), 6.51 (dd, *J* = 7.6, 0.8 Hz, 1H), 5.67 (d, *J* = 10.0 Hz, 1H), 4.44 (d, *J* = 10.4 Hz, 1H), 4.38 (dd, *J* = 10.4, 5.2 Hz, 1H), 3.77 (s, 3H), 3.61 (s, 3H), 3.23 (s, 3H); ^{13}C NMR (101 MHz, $CDCl_3$) δ 176.4, 169.5, 168.7, 145.5, 135.6, 135.5, 134.0, 131.4, 128.8, 128.7, 127.7, 127.5, 126.4, 126.3, 122.5, 111.8, 62.5, 55.4, 52.8, 52.5, 40.4, 27.2. IR (film): v_{max} (cm^{-1}) = 2957, 1753, 1722, 1606, 1489, 1436, 1360, 1310, 1263, 1152, 1076, 1058, 981, 957, 902, 834, 810, 776, 738, 695, 676, 643, 620, 588, 573, 555, 499, 478, 448. HRMS (ESI): Exact mass calcd. for $C_{23}H_{20}NO_5NaBr$ ($[M+Na]^+$): 492.04171. Found: 492.04185.

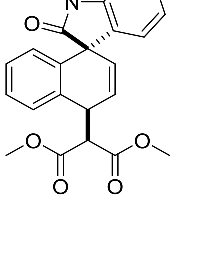

3i: white foam, 36.8 mg, 40% yield. ^{1}H NMR (400 MHz, $CDCl_3$) δ 7.37 (d, *J* = 7.6 Hz, 1H), 7.28 (d, *J* = 7.6 Hz, 1H), 7.23-7.19 (m, 1H), 7.16 (s, 1H), 7.09-7.05 (m, 2H), 6.58 (dd, *J* = 9.6, 4.8 Hz, 1H), 6.47-6.45 (m, 1H), 5.67 (d, *J* = 9.6 Hz, 1H), 4.45 (d, *J* = 10.4 Hz, 1H), 4.40 (dd, *J* = 10.4, 4.8 Hz, 1H), 3.77 (s, 3H), 3.62 (s, 3H), 3.29 (s, 3H); ^{13}C NMR (101 MHz, $CDCl_3$) δ 176.3, 169.4, 168.6, 144.8, 138.8, 135.6, 135.1, 131.7, 131.4 (q, *J* = 32.6 Hz), 128.8, 128.3, 127.8, 127.5, 127.4, 125.3, 124.0 (q, *J* = 273.4 Hz), 120.7 (q, *J* = 3.9 Hz), 105.1 (q, *J* = 3.7 Hz), 62.4, 55.5, 52.8, 52.6, 40.4, 27.2; ^{19}F NMR (377 MHz, $CDCl_3$) δ -62.4 (s). IR (film): v_{max} (cm^{-1}) = 2954, 1743, 1713, 1623, 1454, 1317, 1257, 1158, 1120, 1058, 966, 906, 867, 830, 772, 747, 720, 672, 644, 601, 555, 508, 477, 451. HRMS (ESI): Exact mass calcd. for $C_{24}H_{20}NO_5F_3Na$ ($[M+Na]^+$): 482.11858. Found: 482.11826.

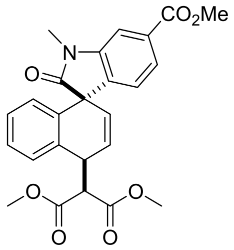

3j: white foam, 46.2 mg, 51% yield. ^{1}H NMR (400 MHz, $CDCl_3$) δ 7.81 (dd, *J* = 7.6, 1.2 Hz, 1H), 7.61 (s, 1H), 7.29-7.27 (m, 1H), 7.22-7.18 (m, 1H), 7.07-7.03 (m, 2H), 6.57 (dd, *J* = 9.6, 5.2 Hz, 1H), 6.46 (d, *J* = 7.2 Hz, 1H), 5.68 (d, *J* = 9.6 Hz, 1H), 4.46 (d, *J* = 10.4 Hz, 1H), 4.40 (dd, *J* = 10.4, 5.2 Hz, 1H), 3.96 (s, 3H), 3.77 (s, 3H), 3.62 (s, 3H), 3.30 (s, 3H); ^{13}C NMR (101 MHz, $CDCl_3$) δ 176.3, 169.5, 168.6, 166.7, 144.5, 140.0, 135.6, 135.3, 131.5, 131.1, 128.7, 128.4, 127.7, 127.5, 127.4, 125.4, 124.9, 109.0, 62.5, 55.7, 52.7, 52.5, 40.4, 27.2. IR (film): v_{max} (cm^{-1}) = 2952, 1713, 1618, 1450, 1329, 1296, 1249, 1193, 1151, 1078, 980, 813, 762, 735, 579, 512. HRMS (ESI): Exact mass calcd. for $C_{25}H_{23}NO_7Na$ ($[M+Na]^+$): 472.13667. Found: 472.13564.

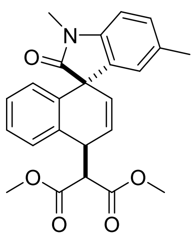

3k: white foam, 75.9 mg, 94% yield. ^{1}H NMR (400 MHz, $CDCl_3$) δ 7.27-7.25 (m, 1H), 7.19-7.15 (m, 2H), 7.07-7.03 (m, 1H), 6.83 (d, *J* = 7.6 Hz, 1H), 6.79 (s, 1H), 6.54-6.51 (m, 2H), 5.71 (d, *J* = 10.0 Hz, 1H), 4.50 (d, *J* = 10.4 Hz, 1H), 4.38 (dd, *J* = 10.4, 5.2 Hz, 1H), 3.76 (s, 3H), 3.61 (s, 3H), 3.23 (s, 3H), 2.28 (s, 3H); ^{13}C NMR (101 MHz, $CDCl_3$) δ 176.5, 169.5, 168.7, 141.7, 136.3, 135.6, 135.1, 133.2, 130.7, 129.6, 129.1, 128.4, 127.6, 127.33, 127.27, 125.6, 107.9, 62.5, 55.7, 52.6, 52.4, 40.4, 27.0, 21.1. IR (film): v_{max} (cm^{-1}) = 2919, 1708, 1602, 1499, 1433, 1328, 1257, 1193, 1148, 1074, 959, 807, 770, 720, 702, 647, 622, 555, 495, 473, 439. HRMS (ESI): Exact mass calcd. for $C_{24}H_{23}NO_5Na$ ([M+Na]$^+$): 428.14684. Found: 428.14759.

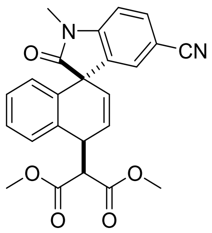

3l: white solid, 41.7 mg, 50% yield, m.p. = 186-188 °C. ^{1}H NMR (400 MHz, $CDCl_3$) δ 7.70 (dd, *J* = 8.0, 1.6 Hz, 1H), 7.30-7.28 (m, 1H), 7.25-7.21 (m, 2H), 7.11-7.07 (m, 1H), 7.03 (d, *J* = 8.0 Hz, 1H), 6.60 (dd, *J* = 9.6, 4.4 Hz, 1H), 6.44 (dd, *J* = 8.0, 0.8 Hz, 1H), 5.65 (d, *J* = 9.6 Hz, 1H), 4.42 (dd, *J* = 10.4, 4.8 Hz, 1H), 4.38 (d, *J* = 10.4 Hz, 1H), 3.77 (s, 3H), 3.63 (s, 3H), 3.29 (s, 3H); ^{13}C NMR (101 MHz, $CDCl_3$) δ 176.3, 169.3, 168.5, 147.9, 136.1, 135.6, 134.6, 134.2, 132.1, 128.9, 128.5, 128.1, 127.8, 127.7, 127.2, 118.9, 108.8, 106.8, 62.3, 55.2, 52.8, 52.6, 40.2, 27.3. IR (film): v_{max} (cm^{-1}) = 2949, 2218, 1720, 1613, 1495, 1436, 1368, 1325, 1261, 1195, 1149, 1119, 1100, 1071, 957, 828, 789, 771, 744, 706, 644, 622, 584, 506, 481, 447. HRMS (ESI): Exact mass calcd. for $C_{24}H_{20}N_2O_5Na$ ([M+Na]$^+$): 439.12644. Found: 439.12583.

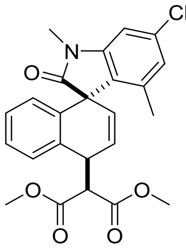

3m: white foam, 70.3 mg, 80% yield. ^{1}H NMR (400 MHz, $CDCl_3$) δ 7.28-7.26 (m, 1H), 7.22-7.17 (m, 1H), 7.08-7.04 (m, 1H), 6.85 (d, *J* = 1.2 Hz, 1H), 6.79 (d, *J* = 1.6 Hz, 1H), 6.57-6.54 (m, 2H), 5.67 (d, *J* = 9.6 Hz, 1H), 4.49 (d, *J* = 10.4 Hz, 1H), 4.39 (dd, *J* = 10.4, 5.2 Hz, 1H), 3.77 (s, 3H), 3.61 (s, 3H), 3.22 (s, 3H), 1.70 (s, 3H); ^{13}C NMR (101 MHz, $CDCl_3$) δ 176.4, 169.5, 168.7, 145.2, 137.0, 135.1, 134.5, 134.4, 131.3, 130.2, 128.9, 128.3, 127.7, 127.6, 126.6, 125.0, 106.7, 62.6, 55.2, 52.7, 52.5, 40.5, 27.2, 17.5. IR (film): v_{max} (cm^{-1}) = 2952, 1742, 1709, 1596, 1436, 1322, 1262, 1195, 1160, 1062, 963, 908, 862, 842, 770, 743, 721, 679, 656, 627, 598, 561, 537, 509, 469. HRMS (ESI): Exact mass calcd. for $C_{24}H_{22}NO_5NaCl$ ($[M+Na]^+$): 462.10787. Found: 462.10661.

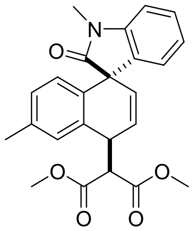

3n: white foam, 63.0 mg, 78% yield. ^{1}H NMR (400 MHz, $CDCl_3$) δ 7.38-7.34 (m, 1H), 7.09-7.06 (m, 2H), 6.97-6.93 (m, 2H), 6.87 (dd, *J* = 8.0, 1.2 Hz, 1H), 6.53 (dd, *J* = 10.0, 5.2 Hz, 1H), 6.39 (d, *J* = 8.0 Hz, 1H), 5.70 (d, *J* = 9.6 Hz, 1H), 4.48 (d, *J* = 10.4 Hz, 1H), 4.33 (dd, *J* = 10.4, 5.2 Hz, 1H), 3.77 (s, 3H), 3.62 (s, 3H), 3.24 (s, 3H), 2.27 (s, 3H); ^{13}C NMR (101 MHz, $CDCl_3$) δ 176.7, 169.6, 168.7, 144.1, 137.2, 135.4, 135.1, 133.1, 130.9, 129.5, 128.9, 128.8, 128.1, 127.3, 124.9, 123.5, 108.2, 62.5, 55.4, 52.6, 52.4, 40.4, 27.0, 21.1. IR (film): v_{max} (cm^{-1}) = 2954, 1747, 1729, 1707, 1610, 1492, 1468, 1435, 1370, 1332, 1312, 1259, 1182, 1153, 1125, 1095, 1073, 963, 899, 847, 828, 748, 730, 688, 655, 633, 542, 515, 474, 434. HRMS (ESI): Exact mass calcd. for $C_{24}H_{23}NO_5Na$ ($[M+Na]^+$): 428.14684. Found: 428.14598.

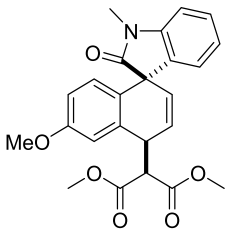

3o: white solid (purified by preparative TLC), 33.0 mg, 39% yield, m.p. = 167-169 °C. ^{1}H NMR (400 MHz, $CDCl_3$) δ 7.36 (t, *J* = 7.6 Hz, 1H), 7.07 (t, *J* = 7.2 Hz, 1H), 6.97-6.92 (m, 2H), 6.82 (s, 1H), 6.61 (d, *J* = 6.8 Hz, 1H), 6.50 (dd, *J* = 9.6, 5.2 Hz, 1H), 6.41 (d, *J* = 8.4 Hz, 1H), 5.71 (d, *J* = 9.6 Hz, 1H), 4.51 (d, *J* = 10.4 Hz, 1H), 4.35 (dd, *J* = 10.4, 5.2 Hz, 1H), 3.77 (s, 3H), 3.74 (s, 3H), 3.64 (s, 3H), 3.24 (s, 3H); ^{13}C NMR (101 MHz, $CDCl_3$) δ 176.9, 169.6, 168.7, 158.8, 144.1, 137.0, 135.1, 130.7, 129.8, 128.8, 128.6, 128.2, 124.9, 123.5, 113.8, 113.0, 108.2, 62.6, 55.4, 55.2, 52.7, 52.6, 40.7, 27.0. IR (film): v_{max} (cm^{-1}) = 2952, 1745, 1708, 1607, 1492, 1468, 1370, 1330, 1258, 1155, 1124, 1093, 1074, 1045, 961, 902, 867, 829, 749, 731, 686, 636, 570, 541, 471, 427. HRMS (ESI): Exact mass calcd. for $C_{24}H_{23}NO_6Na$ ($[M+Na]^+$): 444.14176. Found: 444.14120.

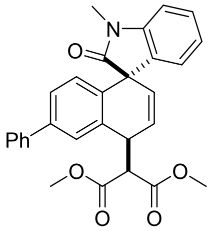

3p: white foam (purified by preparative TLC), 76.6 mg, 82% yield. ^{1}H NMR (400 MHz, $CDCl_3$) δ 7.54-7.52 (m, 3H), 7.42-7.36 (m, 3H), 7.33-7.28 (m, 2H), 7.09 (t, *J* = 7.2 Hz, 1H), 7.00 (d, *J* = 7.2 Hz, 1H), 6.96 (d, *J* = 7.6 Hz, 1H), 6.59-6.56 (m, 2H), 5.74 (d, *J* = 10.0 Hz, 1H), 4.57 (d, *J* = 10.8 Hz, 1H), 4.46 (dd, *J* = 10.4, 5.2 Hz, 1H), 3.78 (s, 3H), 3.55 (s, 3H), 3.27 (s, 3H); ^{13}C NMR (101 MHz, $CDCl_3$) δ 176.5, 169.6, 168.7, 144.1, 140.2, 136.1, 135.1, 134.9, 131.0, 129.5, 128.94, 128.91, 128.0, 127.5, 127.1, 126.9, 125.9, 125.0, 123.6, 108.3, 62.6, 55.6, 52.7, 52.6, 40.6, 27.0. IR (film): v_{max} (cm^{-1}) = 2951, 1744, 1702, 1608, 1470, 1433, 1366, 1321, 1257, 1193, 1152,

1128, 1076, 1019, 963, 899, 825, 759, 728, 700, 632, 541, 513, 484, 460, 430. HRMS (ESI): Exact mass calcd. for $C_{29}H_{25}NO_5Na$ ([M+Na]$^+$): 490.16249. Found: 490.16171.

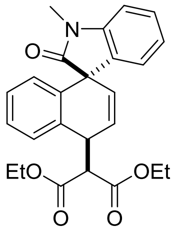

3q: white foam, 73.3 mg, 87% yield. ^{1}H NMR (400 MHz, $CDCl_3$) δ 7.35 (t, *J* = 7.6 Hz, 1H), 7.31 (d, *J* = 7.6 Hz, 1H), 7.16 (t, *J* = 7.2 Hz, 1H), 7.08-7.02 (m, 2H), 6.96-6.93 (m, 2H), 6.55 (dd, *J* = 10.0, 4.0 Hz, 1H), 6.50 (d, *J* = 8.0 Hz, 1H), 5.70 (d, *J* = 10.0 Hz, 1H), 4.43-4.37 (m, 2H), 4.31-4.03 (m, 4H), 3.25 (s, 3H), 1.26 (t, *J* = 7.2 Hz, 3H), 1.13 (t, *J* = 7.2 Hz, 3H); ^{13}C NMR (101 MHz, $CDCl_3$) δ 176.5, 169.1, 168.3, 144.1, 136.1, 135.8, 135.2, 131.1, 129.2, 128.80, 128.76, 127.5, 127.3, 127.1, 124.9, 123.5, 108.2, 62.8, 61.5, 61.4, 55.6, 40.2, 26.9, 14.1, 14.0. IR (film): v_{max} (cm^{-1}) = 2980, 1729, 1709, 1609, 1490, 1465, 1348, 1298, 1228, 1180, 1127, 1094, 1076, 1036, 991, 939, 847, 789, 753, 724, 688, 664, 632, 540, 491, 474, 446. HRMS (ESI): Exact mass calcd. for $C_{25}H_{25}NO_5Na$ ([M+Na]$^+$): 442.16249. Found: 442.16243.

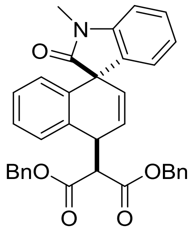

3r: colorless oil, 88.1 mg, 81% yield. ^{1}H NMR (400 MHz, $CDCl_3$) δ 7.37-7.33 (m, 1H), 7.31-7.27 (m, 5H), 7.26-7.20 (m, 4H), 7.17-7.14 (m, 2H), 7.07-6.97 (m, 3H), 6.95-6.92 (m, 2H), 6.52-6.48 (m, 2H), 5.68 (d, *J* = 10.0 Hz, 1H), 5.26 (AB, J_{AB} = 12.4 Hz, 1H), 5.16-5.12 (m, 2H), 4.98 (BA, J_{BA} = 12.4 Hz, 1H), 4.57 (d, *J* = 10.0 Hz, 1H), 4.42 (dd, *J* = 10.0, 5.2 Hz, 1H), 3.23 (s, 3H); ^{13}C NMR (101 MHz, $CDCl_3$) δ 176.5, 168.7, 168.0, 144.2, 136.1, 135.7, 135.6, 135.5, 135.2, 130.8, 129.3, 128.9, 128.7, 128.5, 128.4, 128.24, 128.21, 128.1, 127.53, 127.48, 127.3, 125.0, 123.5, 108.2, 67.2, 67.0, 62.7, 55.6, 40.2, 27.0. IR (film): v_{max} (cm^{-1}) = 2932, 1712, 1610, 1491, 1469,

1368, 1343, 1253, 1153, 1074, 960, 907, 726, 694, 647, 541, 488. HRMS (ESI): Exact mass calcd. for $C_{35}H_{29}NO_5Na$ ($[M+Na]^+$): 566.19379. Found: 566.19338.

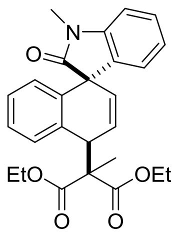

3s: white foam, 24.4 mg, 28% yield. ^{1}H NMR (400 MHz, $CDCl_3$) δ 7.31-7.27 (m, 1H), 7.26-7.25 (m, 1H), 7.13-7.09 (m, 1H), 7.04-6.96 (m, 2H), 6.91 (d, *J* = 8.0 Hz, 1H), 6.78 (dd, *J* = 7.6, 0.8 Hz, 1H), 6.52 (dd, *J* = 7.6, 1.2 Hz, 1H), 6.18 (dd, *J* = 10.0, 4.0 Hz, 1H), 5.60 (dd, *J* = 10.0, 1.2 Hz, 1H), 4.88 (d, *J* = 3.6 Hz, 1H), 4.30-4.23 (m, 4H), 3.30 (s, 3H), 1.40 (s, 3H), 1.29 (q, *J* = 7.2 Hz, 6H); ^{13}C NMR (101 MHz, $CDCl_3$) δ 177.5, 172.0, 171.2, 143.2, 136.7, 135.6, 133.8, 128.6, 128.4, 128.1, 127.8, 127.6, 127.5, 127.3, 124.8, 123.5, 108.1, 61.8, 61.7, 59.6, 54.7, 42.5, 26.8, 15.6, 14.2, 14.1. IR (film): v_{max} (cm^{-1}) = 2922, 1725, 1607, 1489, 1469, 1367, 1341, 1297, 1239, 1103, 1019, 960, 860, 749, 690, 540, 476. HRMS (ESI): Exact mass calcd. for $C_{26}H_{27}NO_5Na$ ($[M+Na]^+$): 456.17814. Found: 456.17784.

General procedure for Pd-catalyzed dearomative reaction of naphthalenes with 1,3-diketones (acetylacetone as an example)

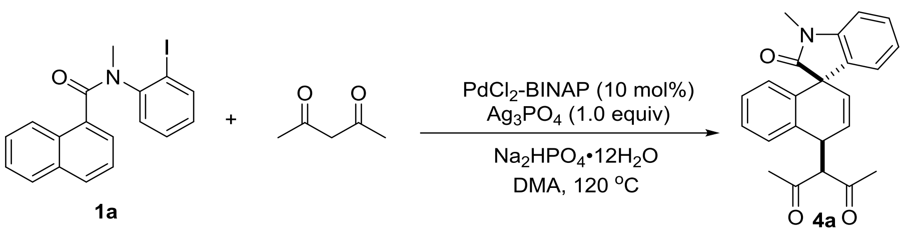

A flame-dried sealed tube was cooled to room temperature under argon. To this tube were added **1a** (0.2 mmol), pre-synthesized $PdCl_2$-BINAP (16.0 mg, 0.02 mmol), $Na_2HPO_4·12H_2O$ (143.3 mg, 0.40 mmol), Ag_3PO_4 (83.7 mg, 0.2 mmol), acetylacetone (40.0 mg, 0.40 mmol) and DMA (1.0 mL). Then the reaction mixture was stirred at 120 °C. After completion (monitored by TLC), the reaction mixture was cooled to room temperature and diluted with ethyl acetate (3 mL). The mixture was filtered through celite, and the filtrate was concentrated under reduced pressure. The crude product was purified by silica gel column chromatography (PE/EtOAc = 10/1) to afford the desired product **4a**.

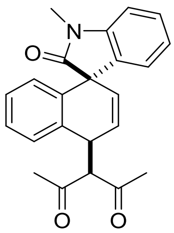

4a: white solid (purified by preparative TLC), 49.7 mg, 69% yield, m.p. = 133-135 °C. ^{1}H NMR (400 MHz, $CDCl_3$) δ 7.40-7.36 (m, 1H), 7.17-7.13 (m, 2H), 7.11-7.03 (m, 2H), 6.98-6.95 (m, 2H), 6.52 (d, *J* = 7.6 Hz, 1H), 6.39 (dd, *J* = 9.6, 5.2 Hz, 1H), 5.65 (d, *J* = 9.6 Hz, 1H), 4.96 (d, *J* = 9.6 Hz, 1H), 4.44 (dd, *J* = 9.6, 5.2 Hz, 1H), 3.28 (s, 3H), 2.24 (s, 3H), 1.95 (s, 3H); ^{13}C NMR (101 MHz, $CDCl_3$) δ 205.1, 202.4, 177.1, 144.0, 136.2, 136.1, 135.2, 131.7, 129.0, 128.9, 128.6, 127.8, 127.7, 127.1, 125.0, 123.8, 108.3, 76.9, 55.7, 40.5, 33.4, 31.6, 27.0. IR (film): v_{max} (cm^{-1}) = 2919, 1713, 1610, 1489, 1469, 1418, 1343, 1275, 1239, 1147, 1125, 1090, 1067, 1019, 960, 813,

787, 773, 747, 716, 691, 642, 592, 541, 523, 491, 443. HRMS (ESI): Exact mass calcd. for $C_{23}H_{21}NO_3Na$ ($[M+Na]^+$): 382.14136. Found: 382.14155.

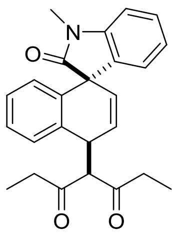

4b: white foam, 51.6 mg, 67% yield. ^{1}H NMR (400 MHz, $CDCl_3$) δ 7.40-7.36 (m, 1H), 7.16-7.06 (m, 3H), 7.05-7.01 (m, 1H), 6.98-6.94 (m, 2H), 6.51 (d, *J* = 7.6 Hz, 1H), 6.36 (dd, *J* = 9.6, 5.6 Hz, 1H), 5.62 (d, *J* = 10.0 Hz, 1H), 4.95 (d, *J* = 10.0 Hz, 1H), 4.47 (dd, *J* = 10.0, 5.2 Hz, 1H), 3.29 (s, 3H), 2.61-2.35 (m, 3H), 1.97-1.87 (m, 1H), 1.07 (t, *J* = 7.2 Hz, 3H), 0.84 (t, *J* = 7.2 Hz, 3H); ^{13}C NMR (101 MHz, $CDCl_3$) δ 207.1, 204.7, 177.1, 143.9, 136.2, 136.0, 135.2, 131.9, 128.9, 128.8, 128.3, 127.6, 127.5, 127.0, 124.9, 123.7, 108.3, 75.5, 55.7, 40.9, 39.8, 37.8, 27.0, 7.6, 7.2. IR (film): ν_{max} (cm^{-1}) = 2922, 1703, 1612, 1492, 1471, 1346, 1242, 1127, 1099, 1021, 982, 789, 766, 746, 714, 689, 541, 490, 443. HRMS (ESI): Exact mass calcd. for $C_{25}H_{25}NO_3Na$ ($[M+Na]^+$): 410.17266. Found: 410.17299.

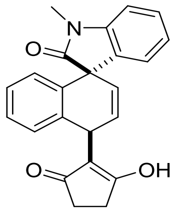

4c: white foam, 30.3 mg, 42% yield. ^{1}H NMR (400 MHz, $CDCl_3$) δ 11.11 (s, 1H), 7.43-7.39 (m, 1H), 7.25-7.23 (m, 1H), 7.19 (d, *J* = 7.2 Hz, 1H), 7.17-7.15 (m, 2H), 7.05-6.98 (m, 2H), 6.40 (d, *J* = 8.0 Hz, 1H), 6.11 (dd, *J* = 10.0, 4.4 Hz, 1H), 5.57 (dd, *J* = 10.0, 2.0 Hz, 1H), 5.00 (d, *J* = 2.4 Hz, 1H), 3.31 (s, 3H), 2.57-2.51 (m, 4H); ^{13}C NMR (101 MHz, $CDCl_3$) δ 205.6, 185.7, 180.6, 143.8, 136.6, 135.4, 132.3, 130.4, 129.6, 129.1, 128.5, 127.2, 126.4, 125.3, 124.6, 124.2, 118.6, 109.0, 54.9, 32.64, 32.60, 28.1, 27.2. IR (film): ν_{max} (cm^{-1}) = 2927, 1682, 1602, 1469, 1371, 1317, 1250, 1216, 1129, 1096, 1059, 998, 952, 812, 763, 742, 691, 655, 543, 494, 471, 454.

HRMS (ESI): Exact mass calcd. for $C_{23}H_{19}NO_3Na$ ([M+Na]$^+$): 380.12571. Found: 380.12554.

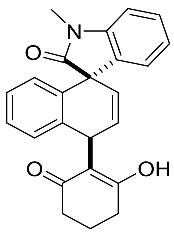

4d: white foam (purified by preparative TLC), 41.0 mg, 55% yield. ^1H NMR (400 MHz, $CDCl_3$) δ 9.26 (s, 1H), 7.42-7.36 (m, 1H), 7.18 (d, *J* = 4.0 Hz, 2H), 7.13 (d, *J* = 4.4 Hz, 2H), 7.02-6.99 (m, 2H), 6.42 (d, *J* = 8.0 Hz, 1H), 6.10 (dd, *J* = 10.0, 4.0 Hz, 1H), 5.59-5.57 (m, 1H), 5.54 (dd, *J* = 10.0, 2.4 Hz, 1H), 3.28 (s, 3H), 2.55-2.46 (m, 4H), 2.03-1.96 (m, 2H); ^{13}C NMR (101 MHz, $CDCl_3$) δ 198.5, 180.3, 174.5, 144.1, 137.0, 135.4, 132.6, 131.3, 129.1, 129.0, 128.4, 127.2, 126.6, 125.2, 124.2, 123.9, 117.3, 108.8, 54.9, 36.9, 32.5, 30.7, 27.0, 20.7. IR (film): v_{max} (cm^{-1}) = 2922, 1695, 1639, 1594, 1469, 1424, 1391, 1367, 1240, 1174, 1127, 1084, 958, 922, 790, 747, 691, 587, 540, 478, 447. HRMS (ESI): Exact mass calcd. for $C_{24}H_{21}NO_3Na$ ([M+Na]$^+$): 394.14136. Found: 394.14119.

General procedure for Pd-catalyzed dearomative reaction of naphthalenes with *β*-ketoesters (ethyl acetoacetate as an example)

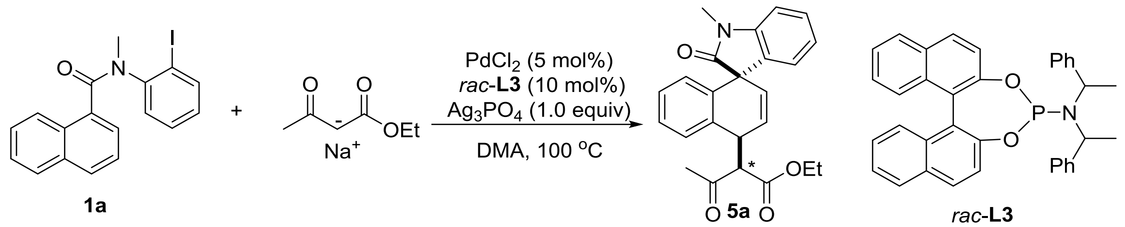

To this tube were added NaH (9.6 mg, 0.4 mmol), ethyl acetoacetate (52.1 mg, 0.4 mmol) and DMA (1.0 mL). Then the reaction mixture was stirred at room temperature for 0.5 h followed by the addition of **1a** (77.4 mg, 0.2 mmol), $PdCl_2$ (1.8 mg, 0.01 mmol), *rac*-**L3** (10.8 mg, 0.02 mmol) and Ag_3PO_4 (83.7 mg, 0.2 mmol). Then the reaction mixture was stirred at 100 °C. After completion (monitored by TLC), the reaction mixture was cooled to room temperature and diluted with ethyl acetate (3 mL). The mixture was filtered through celite, and the filtrate was concentrated under reduced pressure. The crude product was purified by silica gel column chromatography (PE/EtOAc = 20/1 – 10:1) to afford the desired product **5a**.

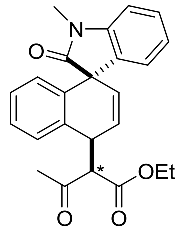

5a: white foam (purified by preparative TLC), 75.1 mg, 96% yield. Two sets of signals were observed due to the existence of diastereoisomers. ^{1}H NMR (400 MHz, $CDCl_3$) 7.39-7.35 (m, 1.5H), 7.18-7.14 (m, 1.5H), 7.11-7.02 (m, 2H), 6.97-6.94 (m, 2H), 6.58-6.49 (m, 1.5H), 6.41 (dd, J = 9.6, 5.2 Hz, 0.5H), 5.69-5.66 (m, 1H), 4.72 (d, J = 10.4 Hz, 0.5H), 4.65 (d, J = 10.0 Hz, 0.5H), 4.45 (dd, J = 10.0, 5.2 Hz, 0.5H), 4.39 (dd, J = 10.4, 5.2 Hz, 0.5H), 4.28-4.07 (m, 2H), 3.27 (s, 1.5H), 3.26 (s, 1.5H), 2.31 (s, 1.5H), 1.91 (s, 1.5H), 1.25 (t, J = 7.2 Hz, 1.5H), 1.17 (t, J = 7.2 Hz, 1.5H); ^{13}C NMR (101 MHz, $CDCl_3$) δ 205.4, 203.0, 177.0, 176.7, 168.8, 168.4, 144.0, 136.6, 136.3, 135.8, 135.4, 135.2, 135.1, 132.1, 130.7, 129.4, 128.88, 128.86, 128.82, 128.7, 128.6,

127.6, 127.5, 127.08, 127.06, 124.9, 123.7, 123.6, 108.3, 108.2, 69.8, 68.5, 61.45, 61.37, 55.8, 55.7, 40.4, 39.7, 33.2, 31.7, 26.94, 26.93, 14.12, 14.10. IR (film): v_{max} (cm^{-1}) = 2979, 1740, 1706, 1610, 1490, 1469, 1344, 1299, 1244, 1180, 1144, 1073, 1020, 960, 856, 748, 691, 540, 491, 445. HRMS (ESI): Exact mass calcd. for $C_{24}H_{23}NO_4Na$ ($[M+Na]^+$): 412.15193. Found: 412.15120.

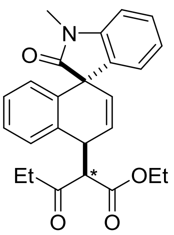

5b: white foam (purified by preparative TLC), 75.1 mg, 93% yield. Two sets of signals were observed due to the existence of diastereoisomers. ^{1}H NMR (400 MHz, $CDCl_3$) δ 7.42-7.37 (m, 1H), 7.20-7.04 (m, 4H), 6.99-6.96 (m, 2H), 6.60 (dd, *J* = 9.6, 5.2 Hz, 0.63H), 6.54-6.50 (m, 1H), 6.37 (dd, *J* = 9.6, 5.2 Hz, 0.37H), 5.70-5.66 (m, 1H), 4.71-4.64 (m, 1H), 4.50 (dd, *J* = 10.0, 5.2 Hz, 0.38H), 4.41 (dd, *J* = 10.8, 5.2 Hz, 0.58H), 4.28-4.08 (m, 2H), 3.30 (s, 1.73H), 3.29 (s, 1.13H), 2.73-2.60 (m, 0.76H), 2.50-2.40 (m, 0.63H), 1.92-1.82 (m, 0.59H), 1.27 (t, *J* = 7.2 Hz, 2H), 1.19 (t, *J* = 7.2 Hz, 1H), 1.09 (t, *J* = 7.2 Hz, 1H), 0.84 (t, *J* = 7.2 Hz, 2H); ^{13}C NMR (101 MHz, $CDCl_3$) δ 207.9, 205.7, 177.1, 176.8, 168.9, 168.7, 144.0, 136.9, 136.3, 135.9, 135.5, 135.3, 135.2, 132.4, 130.8, 129.4, 128.9, 128.7, 128.6, 127.61, 127.57, 127.54, 127.46, 127.12, 127.08, 125.0, 123.73, 123.66, 108.3, 108.2, 69.1, 67.9, 61.5, 61.4, 55.8, 55.7, 40.6, 39.9, 39.6, 38.1, 27.0, 14.2, 7.5, 7.1. IR (film): v_{max} (cm^{-1}) = 2935, 1738, 1707, 1610, 1490, 1469, 1367, 1344, 1298, 1232, 1175, 1125, 1094, 959, 788, 749, 690, 541, 491, 445. HRMS (ESI): Exact mass calcd. for $C_{25}H_{25}NO_4Na$ ($[M+Na]^+$): 426.16758. Found: 426.16690.

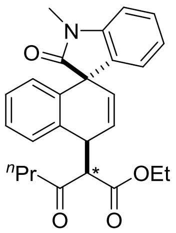

5c: white foam, 82.4 mg, 99% yield. Two sets of signals were observed due to the existence of diastereoisomers. ^{1}H NMR (400 MHz, $CDCl_3$) δ 7.39-7.35 (m, 1.4H), 7.17-7.13 (m, 1.7H), 7.10-7.01 (m, 2H), 6.95 (d, *J* = 6.0 Hz, 2H), 6.57 (dd, *J* = 9.6, 5.2 Hz, 0.55H), 6.52-6.48 (m, 1H), 6.36 (dd, *J* = 9.6, 5.2 Hz, 0.42H), 5.67-5.64 (m, 1H), 4.68-4.62 (m, 1H), 4.47 (dd, *J* = 10.0, 5.2 Hz, 0.43H), 4.40 (dd, *J* = 10.4, 5.2 Hz, 0.57H), 4.28-4.05 (m, 2H), 3.28 (s, 1.55H), 3.27 (s, 1.33H), 2.65-2.53 (m, 0.85H), 2.45-2.37 (m, 0.62H), 1.87-1.79 (m, 0.63H), 1.68-1.59 (m, 0.89H), 1.50-1.30 (m, 1.32H), 1.25 (t, *J* = 7.2 Hz, 1.79H), 1.17 (t, *J* = 7.2 Hz, 1.3H), 0.90 (t, *J* = 7.2 Hz, 1.35H), 0.74 (t, *J* = 7.2 Hz, 1.75H); ^{13}C NMR (101 MHz, $CDCl_3$) δ 207.1, 205.0, 177.0, 176.8, 168.8, 168.5, 144.1, 144.0, 136.8, 136.3, 135.9, 135.6, 135.3, 135.2, 132.3, 130.9, 129.5, 128.89, 128.87, 128.85, 128.70, 128.67, 127.6, 127.52, 127.51, 127.48, 127.1, 125.0, 123.7, 123.6, 108.3, 108.2, 69.4, 68.1, 61.4, 61.3, 55.8, 55.7, 48.0, 46.4, 40.5, 39.9, 27.03, 27.01, 16.8, 16.4, 14.192, 14.186, 13.6, 13.5. IR (film): v_{max} (cm^{-1}) = 2962, 2932, 2875, 1740, 1707, 1610, 1489, 1469, 1419, 1366, 1343, 1300, 1241, 1173, 1123, 1092, 1073, 1023, 989, 960, 748, 716, 690, 541, 491, 474, 445. HRMS (ESI): Exact mass calcd. for $C_{26}H_{27}NO_4Na$ ([M+Na]$^+$): 440.18323. Found: 440.18355.

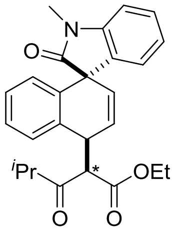

5d: white solid (purified by preparative TLC), 50.0 mg, 60% yield, m.p. = 114-116 °C. Two sets of signals were observed due to the existence of diastereoisomers. ^{1}H NMR (400 MHz, $CDCl_3$) δ 7.39-7.33 (m, 1.43H), 7.17-7.00 (m, 3.86H), 6.96-6.93 (m, 2H), 6.58 (dd, *J* = 9.6, 5.2 Hz, 0.65H), 6.51-6.47 (m, 1H), 6.30 (dd, *J* = 9.6, 5.2 Hz, 0.35H),

5.68-5.62 (m, 1H), 4.84 (d, *J* = 10.8 Hz, 0.61H), 4.78 (d, *J* = 10.4 Hz, 0.35H), 4.48 (dd, *J* = 10.4, 5.6 Hz, 0.39H), 4.42 (dd, *J* = 10.8, 5.6 Hz, 0.59H), 4.29-4.03 (m, 2H), 3.28 (s, 1.74H), 3.26 (s, 1.08H), 2.88-2.77 (m, 0.38H), 2.22-2.12 (m, 0.61H), 1.24 (t, *J* = 7.2 Hz, 2H), 1.17-1.11 (m, 3H), 1.06 (d, *J* = 7.2 Hz, 2H), 0.66 (d, *J* = 6.8 Hz, 2H); ^{13}C NMR (101 MHz, $CDCl_3$) δ 210.3, 208.5, 177.0, 176.7, 168.8, 168.6, 144.11, 144.05, 136.8, 136.3, 136.0, 135.8, 135.31, 135.29, 132.4, 131.0, 129.7, 128.87, 128.85, 128.84, 128.72, 128.69, 127.6, 127.51, 127.49, 127.4, 127.09, 127.06, 125.0, 123.7, 123.6, 108.2, 108.1, 68.0, 66.9, 61.4, 61.3, 55.9, 55.7, 43.5, 42.4, 40.8, 40.4, 27.1, 27.0, 17.9, 17.74, 17.70, 16.2, 14.2. IR (film): ν_{max} (cm^{-1}) = 2969, 2922, 1736, 1702, 1610, 1488, 1468, 1367, 1344, 1299, 1271, 1230, 1158, 1117, 1092, 1057, 1003, 961, 750, 725, 690, 666, 636, 541, 491, 474, 444. HRMS (ESI): Exact mass calcd. for $C_{26}H_{27}NO_4Na$ ([M+Na]$^+$): 440.18323. Found: 440.18328.

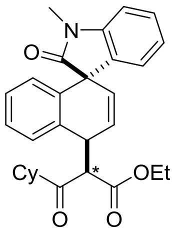

5e: yellow oil (purified by preparative TLC), 51.2 mg, 56% yield. Two sets of signals were observed due to the existence of diastereoisomers. ^{1}H NMR (400 MHz, $CDCl_3$) δ 7.39-7.31 (m, 1H), 7.16-7.01 (m, 4H), 6.96-6.93 (m, 2H), 6.56 (dd, *J* = 9.6, 5.2 Hz, 0.65H), 6.51-6.48 (m, 1H), 6.32 (dd, *J* = 9.6, 5.2 Hz, 0.37H), 5.67-5.62 (m, 1H), 4.83 (d, *J* = 10.8 Hz, 0.65H), 4.76 (d, *J* = 10.4 Hz, 0.36H), 4.48-4.40 (m, 1H), 4.28-4.04 (m, 2H), 3.28-3.26 (m, 3H), 1.98-1.84 (m, 2H), 1.78-1.62 (m, 2H), 1.56-1.49 (m, 1H), 1.33-0.88 (m, 9H); ^{13}C NMR (101 MHz, $CDCl_3$) δ 209.6, 207.7, 176.8, 176.6, 168.8, 168.6, 144.11, 144.07, 136.7, 136.2, 136.1, 135.9, 135.2, 132.3, 131.2, 129.6, 128.83, 128.80, 128.7, 128.6, 127.5, 127.43, 127.40, 127.36, 127.0, 124.9, 123.6, 123.5, 108.2, 108.1, 68.1, 66.9, 61.3, 61.2, 55.8, 55.6, 52.9, 51.8, 40.7, 40.4, 28.1, 28.0, 27.8, 27.02, 26.98, 26.4, 25.9, 25.8, 25.6, 25.1, 14.19, 14.15. IR (film): ν_{max} (cm^{-1}) = 2928, 2853, 1738, 1706, 1610, 1469, 1448, 1367, 1343, 1244, 1165, 1125, 1093, 1072, 960, 748,

690, 541, 493, 474, 444. HRMS (ESI): Exact mass calcd. for $C_{29}H_{31}NO_4Na$ ($[M+Na]^+$): 480.21453. Found: 480.21385.

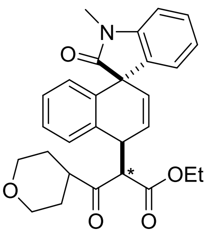

5f: white foam (purified by preparative TLC), 69.9 mg, 76% yield. Two sets of signals were observed due to the existence of diastereoisomers. ^{1}H NMR (400 MHz, $CDCl_3$) δ 7.40-7.34 (m, 1H), 7.18-7.02 (m, 4H), 6.98-6.93 (m, 2H), 6.59 (dd, *J* = 9.6, 5.6 Hz, 0.68H), 6.53-6.49 (m, 1H), 6.31 (dd, *J* = 9.6, 5.2 Hz, 0.31H), 5.69-5.64 (m, 1H), 4.86 (d, *J* = 10.8 Hz, 0.63H), 4.80 (d, *J* = 10.0 Hz, 0.30H), 4.51-4.40 (m, 1H), 4.27-3.73 (m, 4H), 3.45-3.39 (m, 1H), 3.31-3.26 (m, 4H), 3.12-3.06 (m, 0.71H), 2.81-2.74 (m, 0.34H), 2.09-1.52 (m, 4H), 1.24 (t, *J* = 7.2 Hz, 2H), 1.24 (t, *J* = 7.2 Hz, 1H); ^{13}C NMR (101 MHz, $CDCl_3$) δ 207.9, 205.9, 176.9, 176.6, 168.5, 168.4, 144.01, 143.97, 136.6, 136.4, 136.0, 135.7, 135.10, 135.06, 132.3, 130.8, 129.6, 129.0, 128.89, 128.86, 128.82, 128.6, 127.7, 127.5, 127.4, 127.12, 127.08, 124.9, 123.7, 123.6, 108.23, 108.16, 67.5, 67.32, 67.28, 67.23, 66.9, 66.5, 61.4, 61.3, 55.8, 55.7, 49.6, 48.7, 40.7, 40.3, 28.2, 27.8, 27.7, 27.4, 27.01, 26.97, 26.0, 14.1. IR (film): v_{max} (cm^{-1}) = 2951, 2844, 1739, 1704, 1610, 1489, 1469, 1367, 1343, 1297, 1238, 1165, 1113, 1091, 1019, 960, 869, 826, 749, 690, 561, 541, 492, 474, 444. HRMS (ESI): Exact mass calcd. for $C_{28}H_{29}NO_5Na$ ($[M+Na]^+$): 482.19379. Found: 482.19481.

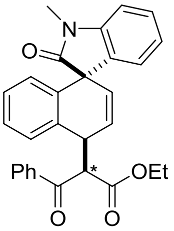

5g: white foam, 59.1 mg, 65% yield. Two sets of signals were observed due to the existence of diastereoisomers. ^{1}H NMR (400 MHz, $CDCl_3$) δ 8.21-8.19 (m, 1H), 7.94-7.92 (m, 1H), 7.58-7.54 (m, 0.5H), 7.49-7.46 (m, 1H), 7.41-7.34 (m, 2H),

7.31-7.27 (m, 1H), 7.20-7.16 (m, 0.5H), 7.12-7.04 (m, 2H), 6.97-6.92 (m, 2H), 6.86-6.82 (m, 1H), 6.67 (dd, *J* = 9.6, 5.2 Hz, 0.5H), 6.54-6.42 (m, 1.5H), 5.73 (d, *J* = 9.6 Hz, 0.5H), 5.60-5.56 (m, 1H), 5.49 (d, *J* = 10.8 Hz, 0.5H), 4.69-4.61 (m, 1H), 4.25-3.95 (m, 2H), 3.31 (s, 1.5H), 3.27 (s, 1.5H), 1.17 (t, *J* = 7.2 Hz, 1.5H), 1.02 (t, *J* = 7.2 Hz, 1.5H); ^{13}C NMR (101 MHz, $CDCl_3$) δ 196.9, 194.3, 177.1, 176.8, 168.8, 168.6, 144.1, 144.0, 137.2, 136.62, 136.58, 136.3, 135.9, 135.7, 135.32, 135.30, 133.7, 133.2, 131.9, 131.6, 129.6, 129.43, 129.35, 129.1, 129.0, 128.82, 128.78, 128.4, 127.5, 127.4, 127.2, 127.1, 126.9, 125.0, 123.63, 123.59, 108.21, 108.16, 66.0, 63.7, 61.4, 55.8, 41.0, 40.7, 27.1, 27.0, 14.1, 14.0. IR (film): v_{max} (cm^{-1}) = 2922, 1707, 1610, 1489, 1469, 1448, 1366, 1343, 1297, 1254, 1201, 1156, 1125, 1094, 1074, 1021, 1001, 960, 784, 749, 689, 613, 540, 492, 474, 445. HRMS (ESI): Exact mass calcd. for $C_{29}H_{25}NO_4Na$ ([M+Na]$^+$): 474.16758. Found: 474.16693.

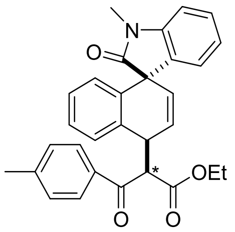

5h: white foam, 54.6 mg, 59% yield. Two sets of signals were observed due to the existence of diastereoisomers. ^{1}H NMR (400 MHz, $CDCl_3$) δ 8.13-8.11 (m, 1H), 7.86-7.84 (m, 1H), 7.40-7.33 (m, 2H), 7.28 (s, 0.5H), 7.20-7.16 (m, 0.5H), 7.13-7.05 (m, 3H), 6.96-6.92 (m, 2H), 6.85-6.83 (m, 1H), 6.67 (dd, *J* = 9.6, 5.6 Hz, 0.5H), 6.52 (d, *J* = 7.6 Hz, 0.5H), 6.48-6.42 (m, 1H), 5.72 (d, *J* = 9.6 Hz, 0.5H), 5.58-5.52 (m, 1H), 5.45 (d, *J* = 10.8 Hz, 0.5H), 4.69-4.61 (m, 1H), 4.22-4.09 (m, 1H), 4.07-3.97 (m, 1H), 3.31 (s, 1.5H), 3.26 (s, 1.5H), 2.39 (s, 1.5H), 2.29 (s, 1.5H), 1.16 (t, *J* = 7.2 Hz, 1.5H), 1.03 (t, *J* = 7.2 Hz, 1.5H); ^{13}C NMR (101 MHz, $CDCl_3$) δ 196.4, 193.8, 177.1, 176.8, 168.9, 168.7, 144.6, 144.11, 144.06, 144.0, 136.7, 136.3, 135.81, 135.78, 135.4, 134.8, 134.1, 131.9, 131.6, 129.7, 129.6, 129.5, 129.2, 128.9, 128.8, 127.5, 127.43, 127.38, 127.2, 127.0, 126.8, 125.0, 123.62, 123.57, 108.2, 108.1, 65.7, 63.6, 61.4, 55.8, 40.9, 40.7, 27.1, 27.0, 21.8, 21.7, 14.1, 14.0. IR (film): v_{max} (cm^{-1}) = 2922, 1708, 1671, 1606, 1469, 1366, 1343, 1296, 1256, 1156, 1124, 1094, 1074, 1021, 959, 832,

749, 692, 590, 540, 474, 445. HRMS (ESI): Exact mass calcd. for $C_{30}H_{27}NO_4Na$ ($[M+Na]^+$): 488.18323. Found: 488.18308.

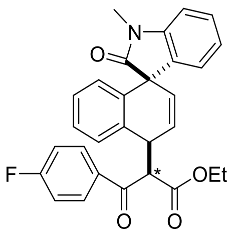

5i: white foam, 71.6 mg, 76% yield. Two sets of signals were observed due to the existence of diastereoisomers. ^1H NMR (400 MHz, $CDCl_3$) δ 8.27-8.24 (m, 1H), 7.99-7.96 (m, 1H), 7.40-7.34 (m, 1.54H), 7.21-7.12 (m, 1.65H), 7.09-7.05 (m, 2H), 6.98-6.91 (m, 3H), 6.87-6.80 (m, 1H), 6.68 (dd, *J* = 9.6, 5.2 Hz, 0.57H), 6.54-6.52 (m, 0.45H), 6.47-6.43 (m, 1H), 5.73 (d, *J* = 9.6 Hz, 0.55H), 5.59 (d, *J* = 10.0 Hz, 0.43H), 5.56 (d, *J* = 10.8 Hz, 0.44H), 5.45 (d, *J* = 10.8 Hz, 0.55H), 4.68-4.58 (m, 1H), 4.26-3.96 (m, 2H), 3.32 (s, 1.67H), 3.27 (s, 1.32H), 1.18 (t, *J* = 7.2 Hz, 1.69H), 1.04 (t, *J* = 7.2 Hz, 1.37H); ^{13}C NMR (101 MHz, $CDCl_3$) δ 195.5, 192.8, 177.2, 176.8, 168.6, 168.5, 166.2 (d, *J* = 256.5 Hz), 165.9 (d, *J* = 256.3 Hz), 144.01, 143.97, 136.5, 136.3, 135.9, 135.5, 135.2, 133.7 (d, *J* = 2.7 Hz), 133.1 (d, *J* = 2.8 Hz), 132.3, 132.2, 132.1, 131.9, 131.4, 129.6, 129.1, 128.9, 128.85, 128.80, 127.6, 127.49, 127.47, 127.2, 127.1, 126.9, 125.0, 123.7, 123.6, 115.9 (d, *J* = 21.9 Hz), 115.5 (d, *J* = 21.9 Hz), 108.3, 108.2, 65.9, 63.6, 61.51, 61.48, 55.9, 41.1, 40.7, 27.1, 27.0, 14.1, 14.0; ^{19}F NMR (377 MHz, $CDCl_3$) δ -104.4 (m), -105.0 (m). IR (film): v_{max} (cm^{-1}) = 2979, 1737, 1706, 1676, 1595, 1489, 1469, 1412, 1366, 1344, 1298, 1255, 1235, 1200, 1156, 1125, 1094, 1074, 1020, 960, 845, 748, 690, 666, 635, 589, 540, 495, 475, 445. HRMS (ESI): Exact mass calcd. for $C_{29}H_{24}NO_4FNa$ ($[M+Na]^+$): 492.15816. Found: 492.15763.

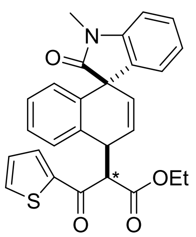

5j: colorless oil (purified by preparative TLC), 81.9 mg, 89% yield. Two sets of signals were observed due to the existence of diastereoisomers. ^{1}H NMR (400 MHz, $CDCl_3$) δ 8.15 (d, *J* = 4.0 Hz, 0.39H), 7.74 (d, *J* = 3.6 Hz, 0.56H), 7.68 (d, *J* = 4.8 Hz, 0.36H), 7.52 (d, *J* = 4.8 Hz, 0.53H), 7.42-7.34 (m, 1H), 7.20-7.04 (m, 3H), 6.97-6.93 (m, 2H), 6.88-6.82 (m, 2H), 6.67 (dd, *J* = 9.6, 5.2 Hz, 0.59H), 6.52 (d, *J* = 8.0 Hz, 0.38H), 6.46-6.41 (m, 1H), 5.72 (d, *J* = 9.6 Hz, 0.55H), 5.60 (d, *J* = 9.6 Hz, 0.38H), 5.39 (d, *J* = 10.4 Hz, 0.39H), 5.28 (d, *J* = 10.8 Hz, 0.57H), 4.65 (dd, *J* = 10.8, 5.2 Hz, 0.39H), 4.56 (dd, *J* = 10.8, 5.2 Hz, 0.57H), 4.27-4.03 (m, 2H), 3.32 (s, 1.68H), 3.26 (s, 1.2H), 1.21 (t, *J* = 7.2 Hz, 1.76H), 1.08 (t, *J* = 7.2 Hz, 1.18H); ^{13}C NMR (101 MHz, $CDCl_3$) δ 189.2, 187.0, 177.2, 176.8, 168.6, 168.5, 145.2, 144.3, 144.1, 144.0, 136.5, 136.3, 135.7, 135.5, 135.42, 135.38, 135.31, 135.26, 135.21, 131.9, 131.2, 129.6, 129.1, 128.89, 128.87, 128.85, 128.7, 128.6, 127.6, 127.5, 127.4, 127.1, 126.9, 124.99, 124.98, 123.7, 123.6, 108.3, 108.2, 66.7, 65.0, 61.55, 61.53, 55.89, 55.87, 41.2, 40.7, 27.12, 27.05, 14.2, 14.1. IR (film): v_{max} (cm^{-1}) = 2979, 1735, 1704, 1650, 1610, 1489, 1469, 1412, 1345, 1301, 1259, 1201, 1155, 1124, 1071, 960, 854, 819, 746, 692, 664, 631, 562, 540, 492, 445. HRMS (ESI): Exact mass calcd. for $C_{27}H_{23}NO_4NaS$ ($[M+Na]^+$): 480.12400. Found: 480.12359.

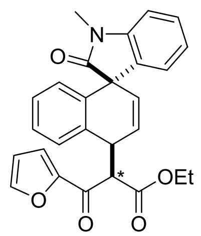

5k: white foam (purified by preparative TLC), 40.1 mg, 45% yield. Two sets of signals were observed due to the existence of diastereoisomers. ^{1}H NMR (400 MHz, $CDCl_3$) δ 7.66-7.65 (m, 0.72H), 7.51-7.50 (m, 0.67H), 7.41-7.34 (m, 1.45H), 7.30 (d, *J* = 3.6 Hz, 0.64H), 7.18 (t, *J* = 7.6 Hz, 0.48H), 7.10-7.04 (m, 2H), 6.98-6.92 (m, 2H), 6.90-6.85 (m, 1H), 6.66 (dd, *J* = 9.6, 5.2 Hz, 0.65H), 6.55-6.50 (m, 0.79H), 6.45-6.40 (m, 1H), 6.27 (dd, *J* = 3.6, 1.6 Hz, 0.6H), 5.71 (d, *J* = 10.0 Hz, 0.6H), 5.61 (d, *J* = 9.6 Hz, 0.36H), 5.19 (d, *J* = 10.8 Hz, 0.36H), 5.06 (d, *J* = 10.8 Hz, 0.61H), 4.65 (dd, *J* = 10.8, 5.2 Hz, 0.36H), 4.53 (dd, *J* = 10.8, 5.2 Hz, 0.60H), 4.28-4.04 (m, 2H), 3.32 (s,

1.76H), 3.27 (s, 1.06H), 1.22 (t, *J* = 7.2 Hz, 1.91H), 1.10 (t, *J* = 7.2 Hz, 1.18H); ^{13}C NMR (101 MHz, $CDCl_3$) δ 183.9, 182.1, 177.2, 176.8, 168.5, 168.4, 152.7, 152.0, 148.2, 148.0, 144.0, 143.9, 136.4, 136.2, 135.6, 135.5, 135.25, 135.23, 132.0, 131.0, 129.5, 129.12, 129.07, 128.89, 128.86, 127.53, 127.47, 127.18, 127.14, 126.9, 125.00, 124.98, 123.8, 123.7, 122.2, 121.9, 112.7, 112.6, 108.3, 108.2, 66.0, 64.5, 61.6, 55.87, 55.82, 41.0, 40.4, 27.1, 27.0, 14.2, 14.1. IR (film): v_{max} (cm^{-1}) = 3017, 1735, 1705, 1664, 1611, 1562, 1489, 1464, 1393, 1367, 1345, 1263, 1218, 1160, 1125, 1075, 1019, 960, 883, 745, 691, 665, 592, 540, 491, 474, 445. HRMS (ESI): Exact mass calcd. for $C_{27}H_{23}NO_5Na$ ($[M+Na]^+$): 464.14684. Found: 464.14699.

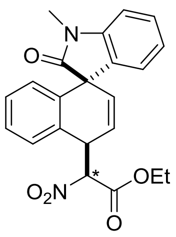

5l: white foam, 18.8 mg, 24% yield. Two sets of signals were observed due to the existence of diastereoisomers. ^{1}H NMR (400 MHz, $CDCl_3$) δ 7.42-7.38 (m, 1H), 7.31 (d, *J* = 7.6 Hz, 0.45H), 7.23-7.17 (m, 1.75H), 7.13-7.10 (m, 2H), 7.00-6.96 (m, 2H), 6.57-6.50 (m, 1.59H), 6.40 (dd, *J* = 9.6, 5.2 Hz, 0.43H), 6.26-6.20 (m, 1H), 5.90-5.87 (m, 1H), 4.70-4.59 (m, 1H), 4.37-4.10 (m, 2H), 3.26-3.25 (m, 3H), 1.32 (t, *J* = 7.2 Hz, 2H), 1.15 (t, *J* = 7.2 Hz, 1H); ^{13}C NMR (101 MHz, $CDCl_3$) δ 176.3, 176.1, 164.7, 163.6, 144.2, 136.9, 136.3, 134.5, 134.3, 132.9, 132.4, 131.9, 129.3, 129.24, 129.22, 128.40, 128.38, 128.35, 128.2, 128.1, 127.9, 127.80, 127.78, 127.5, 125.02, 125.00, 123.84, 123.81, 108.5, 96.12, 96.05, 63.1, 62.9, 56.2, 56.0, 42.1, 41.7, 27.2, 27.1, 14.0, 13.9. IR (film): v_{max} (cm^{-1}) = 2922, 1744, 1708, 1611, 1556, 1490, 1469, 1345, 1241, 1188, 1125, 1074, 1018, 961, 852, 748, 720, 690, 662, 632, 541, 492, 445. HRMS (ESI): Exact mass calcd. for $C_{22}H_{20}N_2O_5Na$ ($[M+Na]^+$): 415.12644. Found: 415.12575.

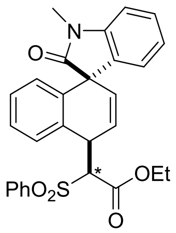

5m: white solid, 50.0 mg, 51% yield, m.p. = 145-147 °C. Two sets of signals were observed due to the existence of diastereoisomers. ^{1}H NMR (400 MHz, $CDCl_3$) δ 8.02-7.94 (m, 2H), 7.71 (d, *J* = 7.6 Hz, 0.24H), 7.63-7.59 (m, 1H), 7.54-7.48 (m, 2H), 7.39-7.34 (m, 1H), 7.24-7.22 (m, 0.71H), 7.15-7.01 (m, 3H), 6.95-6.90 (m, 2H), 6.81 (dd, *J* = 9.6, 5.6 Hz, 0.78H), 6.52-6.46 (m, 1H), 6.33 (dd, *J* = 9.6, 4.8 Hz, 0.24H), 5.76-5.72 (m, 1H), 5.46 (d, *J* = 10.8 Hz, 0.75H), 5.15 (d, *J* = 7.2 Hz, 0.22H), 4.79-4.78 (m, 0.24H), 4.62 (dd, *J* = 10.8, 5.6 Hz, 0.8H), 3.97 (q, *J* = 7.2 Hz, 0.46H), 3.80-3.72 (m, 1.6H), 3.24 (s, 3H), 0.94 (t, *J* = 7.2 Hz, 0.68H), 0.80 (t, *J* = 7.2 Hz, 2.33H); ^{13}C NMR (101 MHz, $CDCl_3$) δ 176.3, 176.1, 166.5, 165.2, 144.1, 144.0, 139.1, 138.8, 137.1, 135.9, 135.0, 134.7, 134.4, 134.1, 134.0, 133.9, 130.6, 130.3, 130.2, 129.9, 129.4, 129.2, 129.02, 128.98, 128.90, 128.87, 128.5, 127.7, 127.55, 127.50, 127.2, 124.92, 124.88, 123.6, 123.5, 108.3, 108.2, 80.4, 77.9, 62.2, 61.8, 55.8, 55.4, 39.8, 38.7, 27.1, 27.0, 13.74, 13.67. IR (film): v_{max} (cm^{-1}) = 2924, 1739, 1708, 1610, 1489, 1469, 1366, 1326, 1289, 1262, 1141, 1080, 1031, 986, 961, 832, 749, 714, 688, 633, 591, 555, 538, 493, 445. HRMS (ESI): Exact mass calcd. for $C_{28}H_{26}NO_5S$ ($[M+H]^+$): 488.15262. Found: 488.15190.

General procedure for Pd-catalyzed dearomative reaction of naphthalenes with amines ($BnNH_2$ as an example)

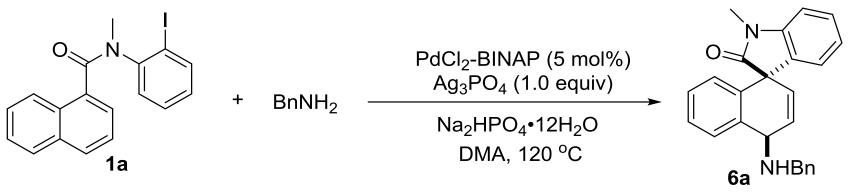

A flame-dried sealed tube was cooled to room temperature under argon. To this tube were added **1a** (77.4 mg, 0.2 mmol), pre-synthesized $PdCl_2$-BINAP (8.0 mg, 0.01 mmol), $Na_2HPO_4{\cdot}12H_2O$ (143.3 mg, 0.40 mmol), Ag_3PO_4 (83.7 mg, 0.2 mmol), $BnNH_2$ (42.9 mg, 0.40 mmol) and DMA (1.0 mL). Then the reaction mixture was stirred at 120 °C. After completion (monitored by TLC), the reaction mixture was cooled to room temperature and diluted with ethyl acetate (3 mL). The mixture was filtered through celite, and the filtrate was concentrated under reduced pressure. The crude product was purified by silica gel column chromatography (PE/EtOAc = 10/1) to afford the desired product **6a**.

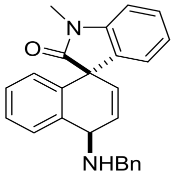

6a: white foam, 71.0 mg, 97% yield. ^{1}H NMR (400 MHz, $CDCl_3$) δ 7.47-7.43 (m, 3H), 7.38-7.31 (m, 3H), 7.28-7.22 (m, 2H), 7.10-7.06 (m, 2H), 7.00 (d, *J* = 6.8 Hz, 1H), 6.95 (d, *J* = 8.0 Hz, 1H), 6.61 (dd, *J* = 9.6, 5.2 Hz, 1H), 6.54 (d, *J* = 7.6 Hz, 1H), 5.75 (d, *J* = 9.6 Hz, 1H), 4.39 (d, *J* = 4.8 Hz, 1H), 4.04-3.91 (m, 2H), 3.24 (s, 3H), 2.64 (s, 1H); ^{13}C NMR (101 MHz, $CDCl_3$) δ 176.9, 144.2, 140.7, 137.4, 136.3, 134.6, 132.5, 129.5, 128.9, 128.6, 128.43, 128.41, 127.4, 127.2, 126.9, 126.8, 124.9, 123.6, 108.4, 55.6, 53.9, 50.5, 27.0. IR (film): v_{max} (cm^{-1}) = 2927, 1705, 1609, 1489, 1468, 1343, 1258, 1124, 1072, 1021, 962, 837, 743, 692, 636, 576, 540, 483, 446. HRMS (ESI): Exact mass calcd. for $C_{25}H_{23}N_2O$ ($[M+H]^+$): 367.18049. Found: 367.18002.

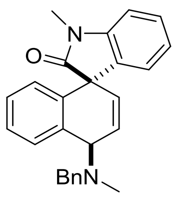

6b: yellow oil, 75.0 mg, 99% yield. ^{1}H NMR (400 MHz, $CDCl_3$) δ 7.79 (d, *J* = 7.6 Hz, 1H), 7.42-7.41 (m, 2H), 7.32 (t, *J* = 7.2 Hz, 2H), 7.28-7.21 (m, 3H), 7.05 (t, *J* = 7.2 Hz, 1H), 6.97 (t, *J* = 7.6 Hz, 1H), 6.91 (d, *J* = 7.6 Hz, 1H), 6.86 (d, *J* = 7.2 Hz, 1H), 6.53 (d, *J* = 7.6 Hz, 1H), 6.39 (dd, *J* = 10.4, 3.2 Hz, 1H), 5.75 (d, *J* = 10.4 Hz, 1H), 4.57 (s, 1H), 3.90 (AB, J_{AB} = 13.2 Hz, 1H), 3.65 (BA, J_{BA} = 13.2 Hz, 1H), 3.28 (s, 3H), 2.36 (s, 3H); ^{13}C NMR (101 MHz, $CDCl_3$) δ 177.8, 143.6, 140.4, 136.6, 135.7, 135.1, 129.4, 128.9, 128.5, 128.3, 127.9, 127.6, 127.5, 126.9, 126.5, 125.2, 124.6, 123.3, 108.2, 58.03, 57.96, 54.4, 38.2, 26.8. IR (film): v_{max} (cm^{-1}) = 3025, 2927, 1712, 1607, 1489, 1468, 1450, 1366, 1339, 1256, 1191, 1156, 1124, 1075, 1019, 991, 970, 875, 777, 733, 695, 632, 540, 482, 446. HRMS (ESI): Exact mass calcd. for $C_{26}H_{25}N_2O$ ($[M+H]^+$): 381.19614. Found: 381.19603.

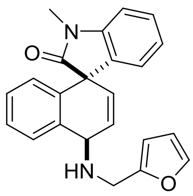

6c: yellow oil, 70.0 mg, 98% yield. ^{1}H NMR (400 MHz, $CDCl_3$) δ 7.43 (d, *J* = 7.6 Hz, 1H), 7.38-7.35 (m, 2H), 7.26 (d, *J* = 8.4 Hz, 1H), 7.08-7.07 (m, 2H), 7.00 (d, *J* = 7.2 Hz, 1H), 6.95 (d, *J* = 7.6 Hz, 1H), 6.59-6.53 (m, 2H), 6.31-6.27 (m, 2H), 5.75 (d, *J* = 9.6 Hz, 1H), 4.42 (d, *J* = 4.4 Hz, 1H), 3.99 (AB, J_{AB} = 14.4 Hz, 1H), 3.90 (BA, J_{BA} = 14.0 Hz, 1H), 3.24 (s, 3H), 3.00 (s, 1H); ^{13}C NMR (101 MHz, $CDCl_3$) δ 176.8, 154.3, 144.1, 141.7, 137.1, 136.3, 134.5, 132.2, 129.4, 128.8, 127.4, 127.2, 127.0, 124.9, 123.6, 110.2, 108.3, 106.9, 55.6, 54.0, 43.3, 27.0. IR (film): v_{max} (cm^{-1}) = 3287, 2886, 1705, 1610, 1489, 1468, 1365, 1343, 1300, 1256, 1218, 1188, 1146, 1124, 1093, 1071, 1009, 961, 917, 862, 836, 742, 690, 664, 636, 599, 563, 540, 478, 446. HRMS (ESI): Exact mass calcd. for $C_{23}H_{21}N_2O_2$ ($[M+H]^+$): 357.15975. Found: 357.16049.

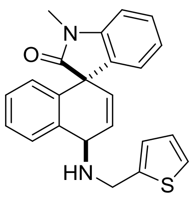

6d: yellow oil, 56.6 mg, 76% yield. ^{1}H NMR (400 MHz, $CDCl_3$) δ 7.44 (d, J = 7.6 Hz, 1H), 7.36 (t, J = 7.6 Hz, 1H), 7.28 (t, J = 7.2 Hz, 1H), 7.24-7.22 (m, 1H), 7.10-7.05 (m, 2H), 7.01-6.99 (m, 2H), 6.97-6.94 (m, 2H), 6.60-6.52 (m, 2H), 5.75 (d, J = 9.6 Hz, 1H), 4.42 (d, J = 4.8 Hz, 1H), 4.21 (AB, J_{AB} = 14.0 Hz, 1H), 4.11 (BA, J_{BA} = 14.0 Hz, 1H), 3.23 (s, 3H), 2.66 (s, 1H); ^{13}C NMR (101 MHz, $CDCl_3$) δ 176.8, 145.3, 144.1, 137.4, 136.2, 134.6, 132.3, 129.4, 128.8, 128.7, 127.4, 127.2, 126.9, 126.6, 124.9, 124.6, 124.3, 123.6, 108.3, 55.6, 53.2, 45.3, 27.0. IR (film): v_{max} (cm^{-1}) = 3285, 3011, 1704, 1610, 1489, 1468, 1365, 1344, 1299, 1257, 1216, 1159, 1125, 1093, 1072, 1020, 993, 961, 836, 743, 692, 665, 636, 563, 540, 472, 446. HRMS (ESI): Exact mass calcd. for $C_{23}H_{21}N_2OS$ ($[M+H]^+$): 373.13691. Found: 373.13723.

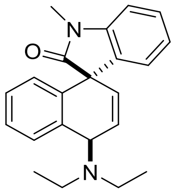

6e: white foam, 58.4 mg, 88% yield. ^{1}H NMR (400 MHz, $CDCl_3$) δ 7.76 (d, J = 7.6 Hz, 1H), 7.28 (t, J = 7.2 Hz, 1H), 7.24-7.21 (m, 1H), 7.05-6.97 (m, 2H), 6.92 (d, J = 8.0 Hz, 1H), 6.89 (d, J = 7.2 Hz, 1H), 6.53 (d, J = 7.6 Hz, 1H), 6.30 (d, J = 10.0 Hz, 1H), 5.66 (d, J = 10.0 Hz, 1H), 4.62 (s, 1H), 3.30 (s, 3H), 2.84-2.79 (m, 2H), 2.59-2.54 (m, 2H), 1.10 (t, J = 6.8 Hz, 6H); ^{13}C NMR (101 MHz, $CDCl_3$) δ 178.1, 143.5, 137.6, 136.0, 135.0, 129.4, 128.4, 127.4, 127.3, 126.7, 126.5, 126.4, 124.6, 123.3, 108.2, 55.1, 54.4, 44.4, 26.7, 14.9. IR (film): v_{max} (cm^{-1}) = 2967, 2800, 1710, 1606, 1488, 1470, 1367, 1342, 1297, 1255, 1193, 1121, 1078, 1048, 983, 950, 769, 741, 692, 607, 541, 479, 448. HRMS (ESI): Exact mass calcd. for $C_{22}H_{25}N_2O$ ($[M+H]^+$): 333.19614. Found: 333.19641.

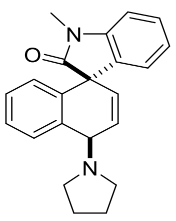

6f: white foam, 65.0 mg, 98% yield. ^{1}H NMR (400 MHz, $CDCl_3$) δ 7.71 (d, *J* = 8.0 Hz, 1H), 7.29 (t, *J* = 7.6 Hz, 1H), 7.25-7.21 (m, 1H), 7.03 (t, *J* = 7.6 Hz, 1H), 6.98 (t, *J* = 7.6 Hz, 1H), 6.92 (d, *J* = 8.0 Hz, 1H), 6.88 (d, *J* = 7.2 Hz, 1H), 6.54 (d, *J* = 7.6 Hz, 1H), 6.33 (dd, *J* = 10.0, 3.6 Hz, 1H), 5.74 (d, *J* = 10.0 Hz, 1H), 4.80 (s, 1H), 3.29 (s, 3H), 2.94-2.92 (m, 2H), 2.72-2.69 (m, 2H), 1.79-1.75 (m, 4H); ^{13}C NMR (101 MHz, $CDCl_3$) δ 177.7, 143.5, 136.5, 135.8, 134.7, 129.5, 128.5, 128.1, 127.5, 127.3, 126.6, 125.6, 124.6, 123.3, 108.2, 55.2, 54.5, 48.1, 26.7, 24.1. IR (film): v_{max} (cm^{-1}) = 2961, 2801, 1709, 1605, 1488, 1468, 1366, 1341, 1299, 1253, 1194, 1124, 1073, 1020, 989, 949, 930, 857, 772, 741, 688, 636, 543, 481, 449. HRMS (ESI): Exact mass calcd. for $C_{22}H_{23}N_2O$ ($[M+H]^+$): 331.18049. Found: 331.18115.

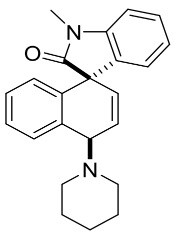

6g: yellow oil, 68.0 mg, 99% yield. ^{1}H NMR (400 MHz, $CDCl_3$) δ 7.75 (d, *J* = 7.6 Hz, 1H), 7.31-7.21 (m, 2H), 7.04 (t, *J* = 7.6 Hz, 1H), 6.98 (t, *J* = 7.6 Hz, 1H), 6.92 (d, *J* = 7.6 Hz, 1H), 6.88 (d, *J* = 7.2 Hz, 1H), 6.53 (d, *J* = 7.6 Hz, 1H), 6.34 (dd, *J* = 10.0, 2.8 Hz, 1H), 5.68 (d, *J* = 10.0 Hz, 1H), 4.46 (s, 1H), 3.29 (s, 3H), 2.67 (s, 4H), 1.62-1.54 (m, 4H), 1.44-1.43 (m, 2H); ^{13}C NMR (101 MHz, $CDCl_3$) δ 177.9, 143.5, 136.4, 135.9, 135.1, 129.3, 128.4, 127.4, 127.3, 127.1, 126.4, 124.6, 123.3, 108.2, 60.5, 54.4, 50.3, 26.9, 26.7, 24.9. IR (film): v_{max} (cm^{-1}) = 2929, 2848, 2801, 1713, 1607, 1488, 1468, 1367, 1339, 1300, 1254, 1204, 1153, 1120, 1077, 1036, 1019, 988, 950, 863, 773, 739, 692, 634, 541, 481, 446. HRMS (ESI): Exact mass calcd. for $C_{23}H_{25}N_2O$ ($[M+H]^+$): 345.19614. Found: 345.19566.

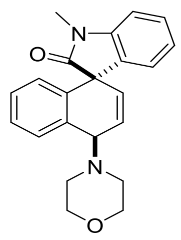

6h: white foam, 68.5 mg, 99% yield. ^{1}H NMR (400 MHz, $CDCl_3$) δ 7.72 (d, *J* = 7.6 Hz, 1H), 7.30 (t, *J* = 7.6 Hz, 1H), 7.25 (t, *J* = 7.6 Hz, 1H), 7.06 (t, *J* = 7.6 Hz, 1H), 7.00 (t, *J* = 7.6 Hz, 1H), 6.94 (d, *J* = 8.0 Hz, 1H), 6.89 (d, *J* = 7.2 Hz, 1H), 6.53 (d, *J* = 7.6 Hz, 1H), 6.31 (dd, *J* = 10.0, 2.8 Hz, 1H), 5.72 (d, *J* = 10.0 Hz, 1H), 4.48 (s, 1H), 3.77-3.68 (m, 4H), 3.29 (s, 3H), 2.75 (s, 4H); ^{13}C NMR (101 MHz, $CDCl_3$) δ 177.6, 143.5, 135.5, 135.4, 135.3, 129.4, 128.6, 128.0, 127.6, 127.5, 126.5, 125.5, 124.6, 123.4, 108.3, 67.9, 60.0, 54.3, 49.4, 26.7. IR (film): v_{max} (cm^{-1}) = 2950, 2884, 2845, 1721, 1608, 1488, 1467, 1367, 1333, 1293, 1250, 1107, 1075, 1004, 986, 950, 928, 865, 851, 775, 742, 711, 689, 641, 584, 541, 487, 468, 445. HRMS (ESI): Exact mass calcd. for $C_{22}H_{23}N_2O_2$ ($[M+H]^+$): 347.17540. Found: 347.17422.

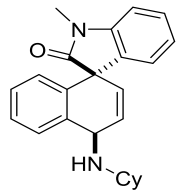

6i: white solid, 68.1 mg, 95% yield, m.p. = 148-150 °C. ^{1}H NMR (400 MHz, $CDCl_3$) δ 7.39-7.35 (m, 2H), 7.27-7.24 (m, 1H), 7.10-7.00 (m, 3H), 6.94 (d, *J* = 7.6 Hz, 1H), 6.61 (dd, *J* = 9.6, 5.2 Hz, 1H), 6.51 (d, *J* = 7.6 Hz, 1H), 5.73 (d, *J* = 9.6 Hz, 1H), 4.49 (d, *J* = 4.0 Hz, 1H), 3.23 (s, 3H), 2.78 (s, 1H), 2.06 (d, *J* = 9.2 Hz, 1H), 1.85-1.65 (m, 4H), 1.33-1.26 (m, 6H); ^{13}C NMR (101 MHz, $CDCl_3$) δ 176.8, 144.2, 138.1, 136.2, 134.6, 133.0, 129.5, 128.8, 128.4, 127.3, 126.9, 124.9, 123.5, 108.3, 55.7, 53.0, 50.9, 33.8, 33.2, 27.0, 26.3, 25.1, 25.0. IR (film): v_{max} (cm^{-1}) = 3283, 2922, 2849, 1700, 1611, 1491, 1468, 1365, 1344, 1258, 1097, 1075, 959, 888, 863, 837, 795, 777, 748, 723, 689, 635, 573, 542, 480, 448, 427. HRMS (ESI): Exact mass calcd. for $C_{24}H_{27}N_2O$ ($[M+H]^+$): 359.21179. Found: 359.21194.

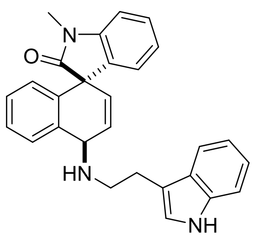

6j: white foam (purified by preparative TLC), 9.8 mg, 12% yield. ^{1}H NMR (400 MHz, $CDCl_3$) δ 8.05 (s, 1H), 7.62 (d, *J* = 7.6 Hz, 1H), 7.40-7.34 (m, 3H), 7.24-7.15 (m, 2H), 7.10-7.05 (m, 4H), 7.01 (d, *J* = 7.2 Hz, 1H), 6.97 (d, *J* = 8.0 Hz, 1H), 6.61 (dd, *J* = 9.6, 4.8 Hz, 1H), 6.54 (d, *J* = 7.6 Hz, 1H), 5.78 (d, *J* = 9.6 Hz, 1H), 4.51 (d, *J* = 3.2 Hz, 1H), 3.25 (s, 3H), 3.13-3.11 (m, 3H), 3.04-2.97 (m, 1H); ^{13}C NMR (101 MHz, $CDCl_3$) δ 176.9, 144.1, 136.4, 136.3, 134.5, 132.0, 129.7, 129.0, 127.7, 127.6, 127.4, 127.0, 125.0, 123.8, 122.1, 121.9, 119.3, 119.2, 114.2, 111.1, 108.5, 55.8, 55.0, 47.2, 27.1, 26.2. IR (film): v_{max} (cm^{-1}) = 2917, 2852, 1700, 1610, 1489, 1467, 1365, 1344, 1300, 1259, 1125, 1092, 961, 907, 739, 690, 637, 541, 475, 447, 425. HRMS (ESI): Exact mass calcd. for $C_{28}H_{26}N_3O$ ($[M+H]^+$): 420.20704. Found: 420.20734.

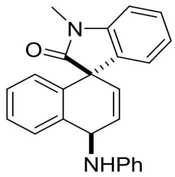

6k: white solid, 68.4 mg, 97% yield, m.p. = 204-205 °C. ^{1}H NMR (400 MHz, $CDCl_3$) δ 7.51 (d, *J* = 7.6 Hz, 1H), 7.37 (t, *J* = 7.2 Hz, 1H), 7.22-7.18 (m, 3H), 7.12-7.06 (m, 3H), 6.95 (d, *J* = 8.0 Hz, 1H), 6.88 (d, *J* = 7.6 Hz, 2H), 6.73 (t, *J* = 7.2 Hz, 1H), 6.59 (dd, *J* = 9.6, 4.8 Hz, 1H), 6.53 (d, *J* = 8.0 Hz, 1H), 5.74 (d, *J* = 9.6 Hz, 1H), 5.35 (s, 1H), 4.94 (s, 1H), 3.24 (s, 3H); ^{13}C NMR (101 MHz, $CDCl_3$) δ 176.9, 147.7, 144.2, 137.4, 136.0, 134.1, 131.2, 129.4, 129.1, 129.0, 128.8, 128.0, 127.5, 126.4, 124.9, 123.7, 118.0, 114.5, 108.5, 55.5, 50.7, 27.1. IR (film): v_{max} (cm^{-1}) = 3336, 3045, 1705, 1598, 1500, 1365, 1342, 1297, 1260, 1173, 1122, 1094, 1057, 983, 955, 870, 742, 724, 690, 615, 541, 498, 470, 447. HRMS (ESI): Exact mass calcd. for $C_{24}H_{21}N_2O$ ($[M+H]^+$): 353.16484. Found: 353.16383.

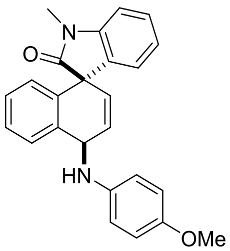

6l: white foam, 61.0 mg, 80% yield. ^{1}H NMR (400 MHz, $CDCl_3$) δ 7.43 (d, *J* = 7.6 Hz, 1H), 7.39-7.35 (m, 1H), 7.18 (t, *J* = 7.2 Hz, 1H), 7.10 (t, *J* = 7.2 Hz, 1H), 7.06-7.03 (m, 2H), 6.95 (d, *J* = 7.6 Hz, 1H), 6.87-6.84 (m, 2H), 6.81-7.79 (m, 2H), 6.60 (dd, *J* = 9.6, 4.8 Hz, 1H), 6.52 (d, *J* = 7.6 Hz, 1H), 5.74 (d, *J* = 9.6 Hz, 1H), 5.20 (d, *J* = 4.8 Hz, 1H), 4.64 (s, 1H), 3.75 (s, 3H), 3.25 (s, 3H); ^{13}C NMR (101 MHz, $CDCl_3$) δ 176.9, 152.7, 144.2, 141.8, 137.5, 136.0, 134.2, 131.7, 129.2, 129.0, 128.8, 127.9, 127.4, 126.5, 124.9, 123.7, 116.9, 114.9, 108.5, 55.8, 55.6, 52.5, 27.1. IR (film): v_{max} (cm^{-1}) = 3300, 2929, 1701, 1610, 1506, 1467, 1365, 1344, 1229, 1178, 1124, 1093, 1035, 984, 960, 821, 746, 689, 637, 585, 540, 512, 488, 447. HRMS (ESI): Exact mass calcd. for $C_{25}H_{23}N_2O_2$ ($[M+H]^+$): 383.17540. Found: 383.17450.

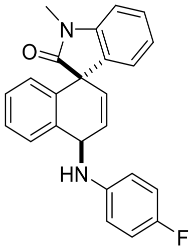

6m: white foam, 73.3 mg, 99% yield. ^{1}H NMR (400 MHz, $CDCl_3$) δ 7.44-7.40 (m, 1H), 7.40-7.36 (m, 1H), 7.20 (t, *J* = 7.6 Hz, 1H), 7.11 (t, *J* = 7.6 Hz, 1H), 7.08-7.05 (m, 2H), 6.96 (d, *J* = 7.6 Hz, 1H), 6.93-6.88 (m, 2H), 6.84-6.81 (m, 2H), 6.59 (dd, *J* = 9.6, 4.8 Hz, 1H), 6.53 (d, *J* = 7.6 Hz, 1H), 5.77 (d, *J* = 9.6 Hz, 1H), 5.22 (d, *J* = 4.4 Hz, 1H), 4.85 (s, 1H), 3.25 (s, 3H); ^{13}C NMR (101 MHz, $CDCl_3$) δ 176.9, 156.3 (d, *J* = 236.3 Hz),144.2, 144.1 (d, *J* = 1.8 Hz), 137.3, 136.1, 134.1, 131.4, 129.3, 129.05, 129.03, 128.0, 127.5, 126.5, 124.9, 123.7, 116.2 (d, *J* = 7.5 Hz), 115.8 (d, *J* = 22.3 Hz), 108.5, 55.7, 52.1, 27.1; ^{19}F NMR (377 MHz, $CDCl_3$) δ -126.9 (s). IR (film): v_{max} (cm^{-1}) = 3299, 3055, 1693, 1607, 1502, 1466, 1364, 1345, 1300, 1260, 1213, 1123, 1099, 1073, 1052, 963, 877, 820, 768, 749, 688, 640, 580, 541, 523, 499, 486, 468,

447, 413. HRMS (ESI): Exact mass calcd. for $C_{24}H_{20}N_2OF$ ([M+H]$^+$): 371.15542. Found: 371.15539.

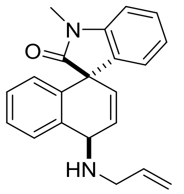

6n: white foam (purified by preparative TLC), 15.6 mg, 25% yield. ^{1}H NMR (400 MHz, $CDCl_3$) δ 7.43 (d, *J* = 7.6 Hz, 1H), 7.38 (t, *J* = 7.6 Hz, 1H), 7.29 (d, *J* = 7.2 Hz, 1H), 7.12-7.06 (m, 2H), 7.02 (d, *J* = 7.6 Hz, 1H), 6.96 (d, *J* = 7.6 Hz, 1H), 6.61 (dd, *J* = 9.6, 5.2 Hz, 1H), 6.53 (d, *J* = 7.6 Hz, 1H), 6.08-5.98 (m, 1H), 5.77 (d, *J* = 9.6 Hz, 1H), 5.29 (d, *J* = 17.2 Hz, 1H), 5.15 (d, *J* = 10.4 Hz, 1H), 4.41 (d, *J* = 5.2 Hz, 1H), 3.50-3.39 (m, 2H), 3.25 (s, 3H), 2.90 (s, 1H); ^{13}C NMR (101 MHz, $CDCl_3$) δ 176.9, 144.2, 137.1, 137.0, 136.4, 134.5, 132.4, 129.6, 129.1, 128.9, 127.5, 127.3, 127.0, 125.0, 123.7, 116.4, 108.4, 55.7, 54.1, 49.3, 27.1. IR (film): v_{max} (cm^{-1}) = 3290, 2925, 1706, 1609, 1489, 1468, 1417, 1366, 1342, 1300, 1257, 1124, 1093, 1072, 1020, 993, 961, 917, 858, 835, 745, 690, 636, 564, 540, 477, 446. HRMS (ESI): Exact mass calcd. for $C_{21}H_{21}N_2O$ ([M+H]$^+$): 317.16484. Found: 317.16568.

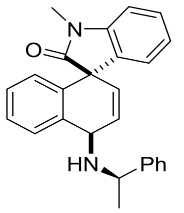

6o: white foam, 72.7 mg, 96% yield. Two sets of signals were observed due to the existence of diastereoisomers. ^{1}H NMR (400 MHz, $CDCl_3$) δ 7.60-7.58 (m, 2H), 7.44-7.22 (m, 5H), 7.14-6.92 (m, 5H), 6.67 (dd, *J* = 9.6, 4.8 Hz, 0.5H), 6.55-6.50 (m, 1H), 6.45 (d, *J* = 7.6 Hz, 0.5H), 5.72 (dd, *J* = 9.6, 6.4 Hz, 1H), 4.29 (q, *J* = 6.4 Hz, 0.5H), 4.14-4.10 (m, 0.5H), 4.07-4.04 (m, 1H), 3.23 (s, 3H), 2.60 (s, 1H), 1.39 (d, *J* = 6.4 Hz, 1.5H), 1.32 (d, *J* = 6.4 Hz, 1.5H); ^{13}C NMR (101 MHz, $CDCl_3$) δ 176.9, 176.8, 146.2, 146.1, 144.24, 144.19, 138.8, 137.9, 136.7, 135.6, 134.6, 134.5, 133.5, 132.4,

129.9, 129.1, 128.82, 128.77, 128.67, 128.6, 128.30, 128.26, 127.8, 127.33, 127.30, 127.26, 127.1, 126.88, 126.82, 126.79, 126.2, 125.0, 124.9, 123.6, 123.5, 108.30, 108.26, 55.9, 55.5, 55.0, 54.6, 51.8, 51.5, 27.03, 27.01, 26.2, 25.8. IR (film): v_{max} (cm^{-1}) = 3294, 2961, 1706, 1610, 1489, 1469, 1450, 1365, 1342, 1301, 1257, 1124, 1102, 1072, 1021, 968, 832, 746, 701, 637, 606, 539, 477, 445. HRMS (ESI): Exact mass calcd. for $C_{26}H_{25}N_2O$ ($[M+H]^+$): 381.19614. Found: 381.19607.

Preliminary asymmetric studies

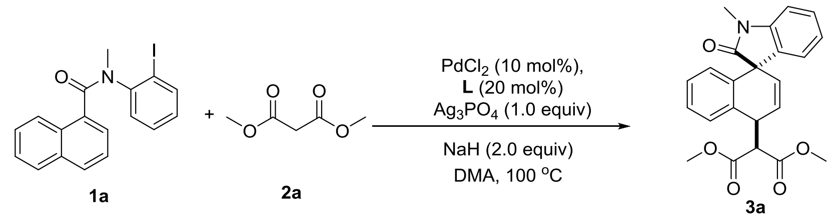

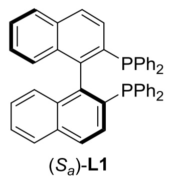

(S_a)-**L1**

3a: 79% NMR yield
rac

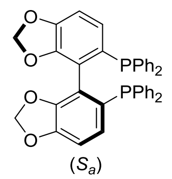

(S_a)

3a: 67% NMR yield
rac

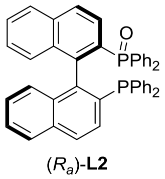

(R_a)-**L2**

3a: 67% NMR yield
5% ee

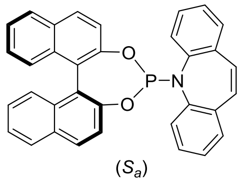

(S_a)

3a: 84% NMR yield
13% ee

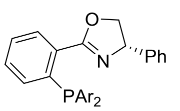

Ar = 4-$CF_3C_6H_4$
(S)-**L4**

3a: 43% NMR yield
1% ee

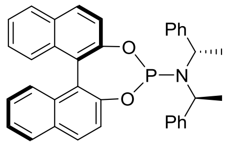

(R_a, S, S)-**L3**

3a: 87% NMR yield
rac

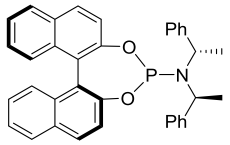

(S_a, S, S)-**L3**

3a: 70% NMR yield
15% ee

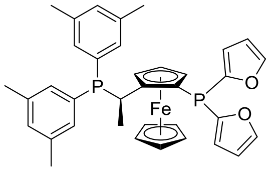

(R_p, R)

3a: 46% NMR yield
9% ee

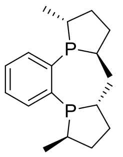

(R, R, R, R)

3a: 13% NMR yield
10% ee

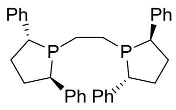

(R, R, R, R)

3a: 62% NMR yield
6% ee

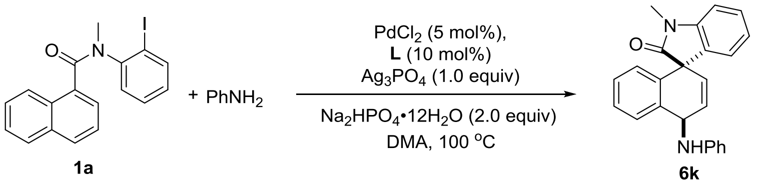

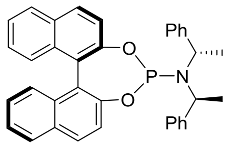

(R_a, S, S)-**L3**

6k: 84% NMR yield
3% ee

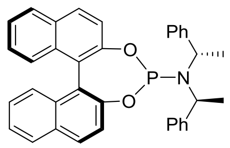

(S_a, S, S)-**L3**

6k: 14% NMR yield
not detect ee

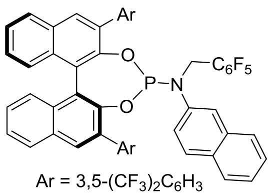

Ar = 3,5-$(CF_3)_2C_6H_3$
(S_a)

6k: 8% NMR yield
30% ee

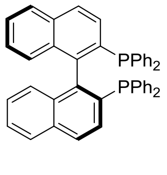

(S_a)-**L1**

6k: 100% NMR yield
3% ee

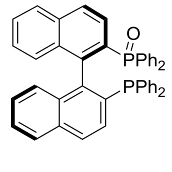

(R_a)-**L2**

6k: 100% NMR yield
rac

Transformations of products

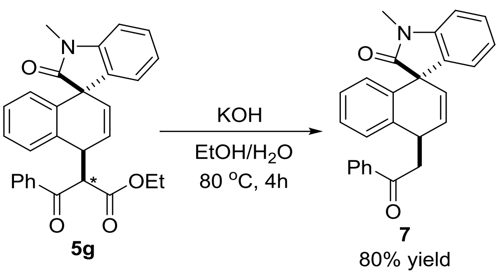

To a sealed tube were added **5g** (36.1 mg, 0.08 mmol), KOH (18 mg, 0.32 mmol, 4.0 equiv), H_2O (0.8 mL) and ethanol (0.8 mL). Then the reaction mixture was stirred at 80 °C for 4 h. After completion (monitored by TLC), the mixture was quenched with sat. NH_4Cl solution (10 mL) and extracted with EtOAc (3 x 10 mL). The combined organic layers were dried with anhydrous Na_2SO_4. The solvent was removed and the crude product was purified by flash chromatography on silica gel (PE/EtOAc = 20/1) to afford **7**.

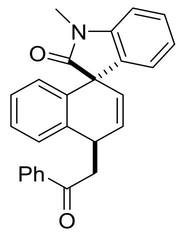

7: white foam, 24.2 mg, 80% yield. ^{1}H NMR (400 MHz, $CDCl_3$) δ 8.04 (d, *J* = 7.6 Hz, 2H), 7.53 (t, *J* = 7.2 Hz, 1H), 7.44 (t, *J* = 7.6 Hz, 2H), 7.38-7.34 (m, 2H), 7.21 (t, *J* = 7.6 Hz, 1H), 7.09 (t, *J* = 7.6 Hz, 1H), 7.05-7.00 (m, 2H), 6.95 (d, *J* = 7.6 Hz, 1H), 6.54-6.49 (m, 2H), 5.57 (d, *J* = 9.6 Hz, 1H), 4.40-4.36 (m, 1H), 3.91 (dd, *J* = 17.6, 9.2 Hz, 1H), 3.59 (dd, *J* = 17.6, 4.8 Hz, 1H), 3.26 (s, 3H); ^{13}C NMR (101 MHz, $CDCl_3$) δ 199.5, 177.4, 144.1, 139.3, 137.2, 135.6, 135.1, 133.2, 132.9, 128.8, 128.7, 128.52, 128.47, 128.0, 127.1, 126.7, 126.3, 125.1, 123.6, 108.2, 55.4, 51.0, 36.3, 26.9. IR (film): v_{max} (cm^{-1}) = 2930, 1711, 1684, 1607, 1489, 1467, 1343, 1263, 1206, 1154, 1128, 1092, 1072, 1023, 1001, 965, 756, 725, 692, 654, 628, 540, 489, 441. HRMS (ESI): Exact mass calcd. for $C_{26}H_{21}NO_2Na$ ([M+Na]$^+$): 402.14645. Found: 402.14677.

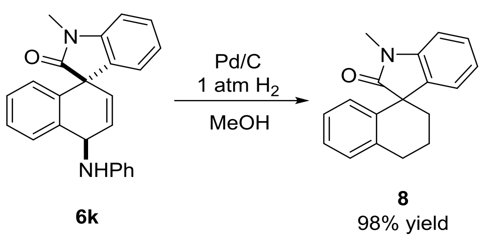

A flame-dried Schlenk tube was cooled down to room temperature under argon. To this tube were added **6k** (70.5 mg, 0.2 mmol), MeOH (2.0 mL) and Pd/C (5%, 30 mg). Then the reaction mixture was subjected to hydrogen (1 atm). The mixture was stirred at 30 °C for 24 h. After completion, the mixture was filtered and the filtrate was concentrated under reduced pressure. The crude product was purified by silica gel column chromatography (PE/EtOAc = 20/1) to afford **8**.

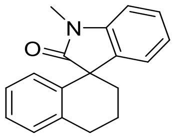

8: white solid, 52.0 mg, 98% yield, m.p. = 124-126 °C. ^{1}H NMR (400 MHz, $CDCl_3$) δ 7.28 (t, J = 7.6 Hz, 1H), 7.16 (d, J = 7.2 Hz, 1H), 7.11 (t, J = 7.2 Hz, 1H), 7.05 (d, J = 7.2 Hz, 1H), 7.01-6.90 (m, 3H), 6.47 (d, J = 7.6 Hz, 1H), 3.28 (s, 3H), 3.04-2.92 (m, 2H), 2.36-2.34 (m, 1H), 2.22-2.16 (m, 1H), 2.03-1.93 (m, 2H); ^{13}C NMR (101 MHz, $CDCl_3$) δ 180.5, 143.2, 137.9, 137.4, 135.2, 129.7, 128.0, 127.9, 127.1, 126.4, 124.1, 122.9, 108.1, 52.3, 34.1, 29.3, 26.6, 18.8. IR (film): v_{max} (cm^{-1}) = 2931, 1710, 1606, 1488, 1466, 1370, 1341, 1301, 1252, 1154, 1130, 1093, 1063, 1022, 965, 771, 753, 689, 624, 588, 539, 494, 477, 439. HRMS (ESI): Exact mass calcd. for $C_{18}H_{18}NO$ ([M+H]$^{+}$): 264.13829. Found: 264.13825.

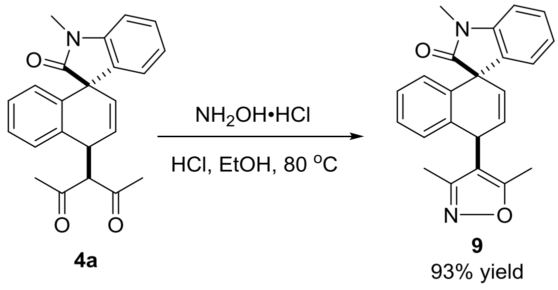

A solution of **4a** (35.9 mg, 0.1 mmol), hydroxylamine hydrochloride (13.9 mg, 0.2 mmol, 2.0 equiv) and con. HCl (20 μL) in ethanol (1 mL) was heated to 80 °C for 7 h. After completion (monitored by TLC), the solvent of reaction mixture was removed under reduced pressure. The crude product was purified by silica gel column chromatography (PE/EtOAc = 20/1) to afford **9**.[1]

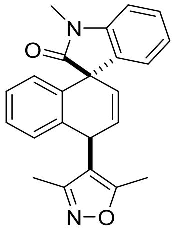

9: white solid, 33.1 mg, 93% yield, m.p. = 170-172 °C. ^{1}H NMR (400 MHz, $CDCl_3$) δ 7.34-7.30 (m, 1H), 7.17-7.13 (m, 1H), 7.08-7.04 (m, 1H), 7.02-7.01 (m, 2H), 6.97-6.95 (m, 2H), 6.65-6.63 (m, 1H), 6.01 (dd, *J* = 10.0, 3.2 Hz, 1H), 5.60 (dd, *J* = 10.0, 2.4 Hz, 1H), 4.78 (t, *J* = 2.8 Hz, 1H), 3.33 (s, 3H), 2.35 (s, 3H), 2.19 (s, 3H); ^{13}C NMR (101 MHz, $CDCl_3$) δ 178.3, 166.2, 160.0, 143.4, 135.9, 135.1, 133.8, 129.0, 128.7, 128.6, 127.9, 127.7, 127.3, 124.7, 124.5, 123.5, 115.8, 108.5, 54.3, 33.5, 26.8, 11.3, 10.7. IR (film): v_{max} (cm^{-1}) = 2925, 1716, 1639, 1605, 1489, 1469, 1370, 1342, 1256, 1202, 1129, 1078, 1016, 952, 888, 817, 774, 750, 690, 631, 541, 475, 449. HRMS (ESI): Exact mass calcd. for $C_{23}H_{20}N_2O_2Na$ ($[M+Na]^+$): 379.14170. Found: 379.14225.

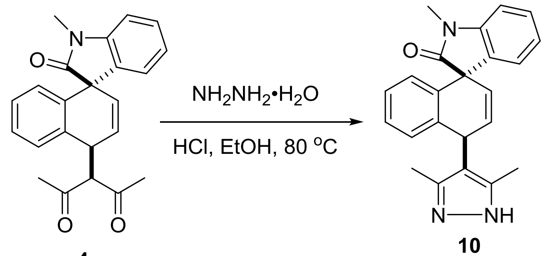

4a **10** 90% yield

A solution of **4a** (35.9 mg, 0.1 mmol), hydrazine monohydrate (10.0 mg, 0.2 mmol, 2.0 equiv) and con. HCl (20 μL) in ethanol (1 mL) was heated to 80 °C for 7 h. After completion (monitored by TLC), the solvent of reaction mixture was removed under

reduced pressure. The crude product was purified by silica gel column chromatography (DCM/MeOH= 20/1) to afford **10**.[1]

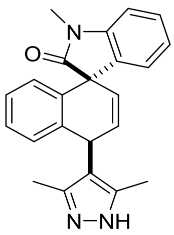

10: white solid, 32.0 mg, 90% yield, m.p. = 129-131 °C. ^{1}H NMR (400 MHz, $CDCl_3$) δ 9.12 (s, 1H), 7.30 (t, *J* = 7.2 Hz, 1H), 7.13-7.10 (m, 1H), 7.05-7.00 (m, 4H), 6.94 (d, *J* = 7.6 Hz, 1H), 6.62 (d, *J* = 7.6 Hz, 1H), 6.05 (dd, *J* = 10.0, 2.4 Hz, 1H), 5.56-5.53 (m, 1H), 4.85 (s, 1H), 3.34 (s, 3H), 2.23 (s, 6H); ^{13}C NMR (101 MHz, $CDCl_3$) δ 178.7, 143.3, 136.8, 136.4, 133.6, 130.1, 129.3, 128.5, 127.7, 127.2, 127.0, 124.8, 123.5, 123.1, 118.0, 108.4, 54.4, 34.3, 26.8, 11.3. IR (film): ν_{max} (cm^{-1}) = 2921, 1712, 1607, 1488, 1468, 1368, 1342, 1299, 1253, 1126, 1080, 953, 909, 826, 728, 691, 541, 478, 447. HRMS (ESI): Exact mass calcd. for $C_{23}H_{22}N_3O$ ($[M+H]^+$): 356.17574. Found: 356.17563.

X-Ray crystal data

X-Ray crystal data of **3a** (CCDC 1982544)

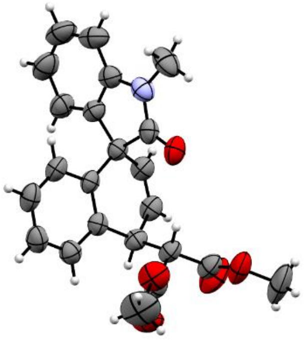

Crystal data and structure refinement for d8v19785.

Identification code	d8v19785	
Empirical formula	C23 H21 N O5	
Formula weight	391.41	
Temperature	293(2) K	
Wavelength	0.71073 Å	
Crystal system	Monoclinic	
Space group	P 21/c	
Unit cell dimensions	a = 16.6707(14) Å	α= 90°.
	b = 9.3051(9) Å	β= 107.808(3)°.
	c = 13.8903(13) Å	γ = 90°.
Volume	2051.5(3) $Å^3$	
Z	4	
Density (calculated)	1.267 Mg/m^3	
Absorption coefficient	0.090 mm^{-1}	
F(000)	824	
Crystal size	0.190 x 0.150 x 0.100 mm^3	
Theta range for data collection	2.676 to 26.000°.	
Index ranges	-20<=h<=19, -11<=k<=11, -17<=l<=16	
Reflections collected	17265	
Independent reflections	4015 [R(int) = 0.0509]	
Completeness to theta = 25.242°	99.5 %	
Absorption correction	Semi-empirical from equivalents	
Max. and min. transmission	0.7456 and 0.5563	
Refinement method	Full-matrix least-squares on F^2	
Data / restraints / parameters	4015 / 0 / 266	

Goodness-of-fit on F^2	1.051
Final R indices [I>2sigma(I)]	R1 = 0.0502, wR2 = 0.1150
R indices (all data)	R1 = 0.0854, wR2 = 0.1388
Extinction coefficient	0.025(3)
Largest diff. peak and hole	0.157 and -0.145 e.Å^{-3}

X-Ray crystal data of **6k** (CCDC 1982545)

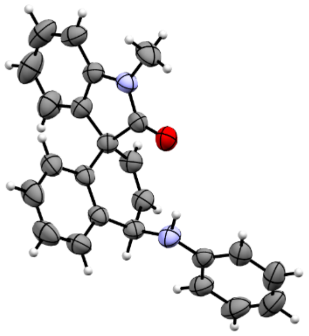

Crystal data and structure refinement for mo_d8v191162_0m.

Identification code	mo_d8v191162_0m	
Empirical formula	C24 H20 N2 O	
Formula weight	352.42	
Temperature	293(2) K	
Wavelength	0.71073 Å	
Crystal system	Monoclinic	
Space group	P 21/n	
Unit cell dimensions	a = 12.8388(4) Å	α= 90°.
	b = 11.4187(4) Å	β= 113.4690(10)°.
	c = 13.7487(4) Å	γ = 90°.
Volume	1848.85(10) Å^3	
Z	4	
Density (calculated)	1.266 Mg/m^3	
Absorption coefficient	0.078 mm^{-1}	
F(000)	744	
Crystal size	0.200 x 0.160 x 0.130 mm^3	
Theta range for data collection	2.406 to 25.990°.	
Index ranges	-15<=h<=14, -14<=k<=14, -16<=l<=16	
Reflections collected	26778	
Independent reflections	3596 [R(int) = 0.0307]	

Completeness to theta = 25.242°	99.4 %
Absorption correction	Semi-empirical from equivalents
Max. and min. transmission	0.7456 and 0.6708
Refinement method	Full-matrix least-squares on F^2
Data / restraints / parameters	3596 / 0 / 250
Goodness-of-fit on F^2	1.015
Final R indices [I>2sigma(I)]	R1 = 0.0415, wR2 = 0.1110
R indices (all data)	R1 = 0.0531, wR2 = 0.1220
Extinction coefficient	0.044(6)
Largest diff. peak and hole	0.182 and -0.145 e.$Å^{-3}$

References

[1] Tang, S.-B.; Zhang, X.; Tu, H.-F.; You, S.-L. *J. Am. Chem. Soc.* **2018**, *140*, 7737.

Copies of NMR spectra

1a

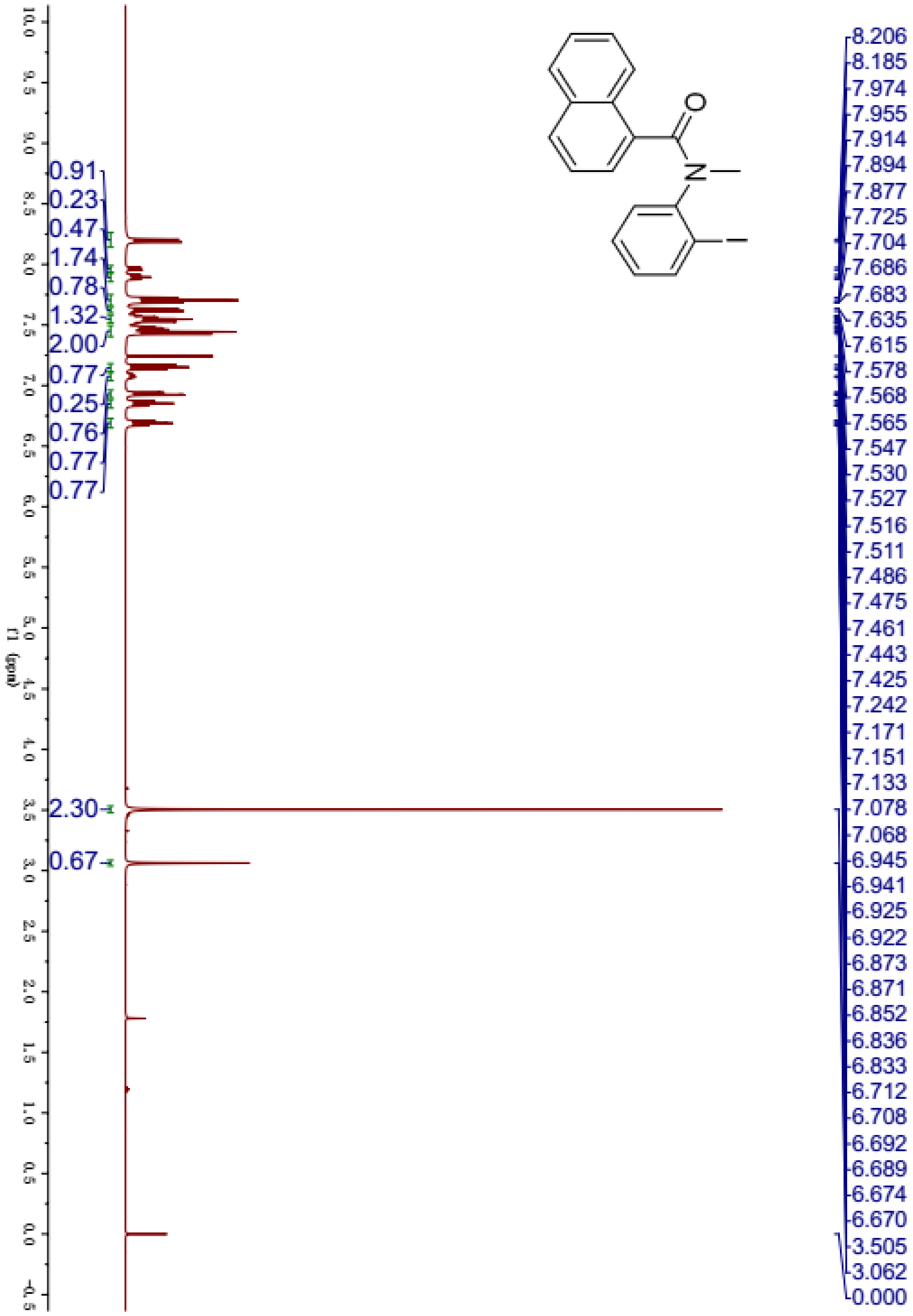

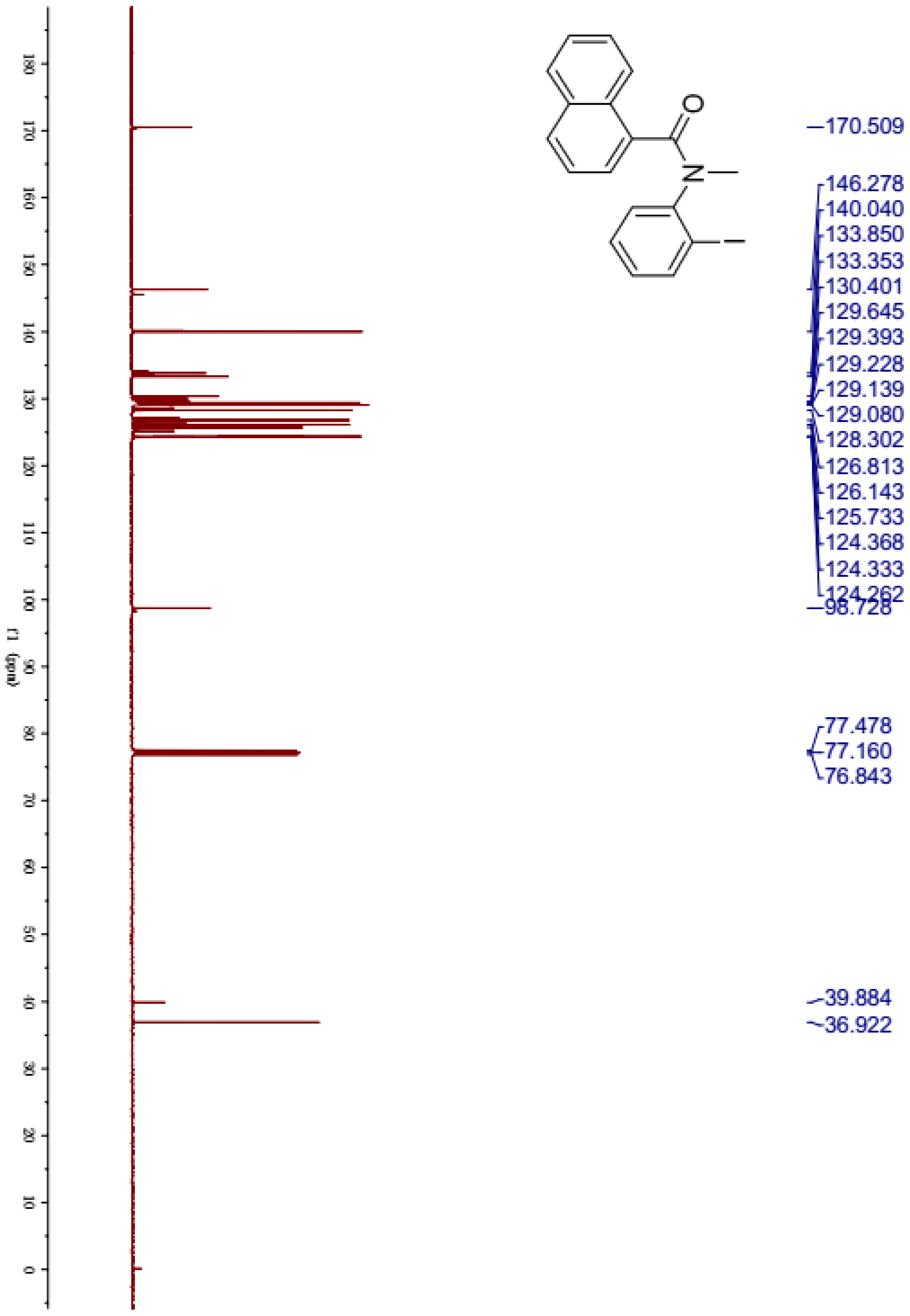
170.509
146.278
140.040
133.850
133.353
130.401
129.645
129.393
129.228
129.139
129.080
128.302
126.813
126.143
125.733
124.368
124.333
124.262
98.728
77.478
77.160
76.843
39.884
36.922
f1 (ppm)

1a’

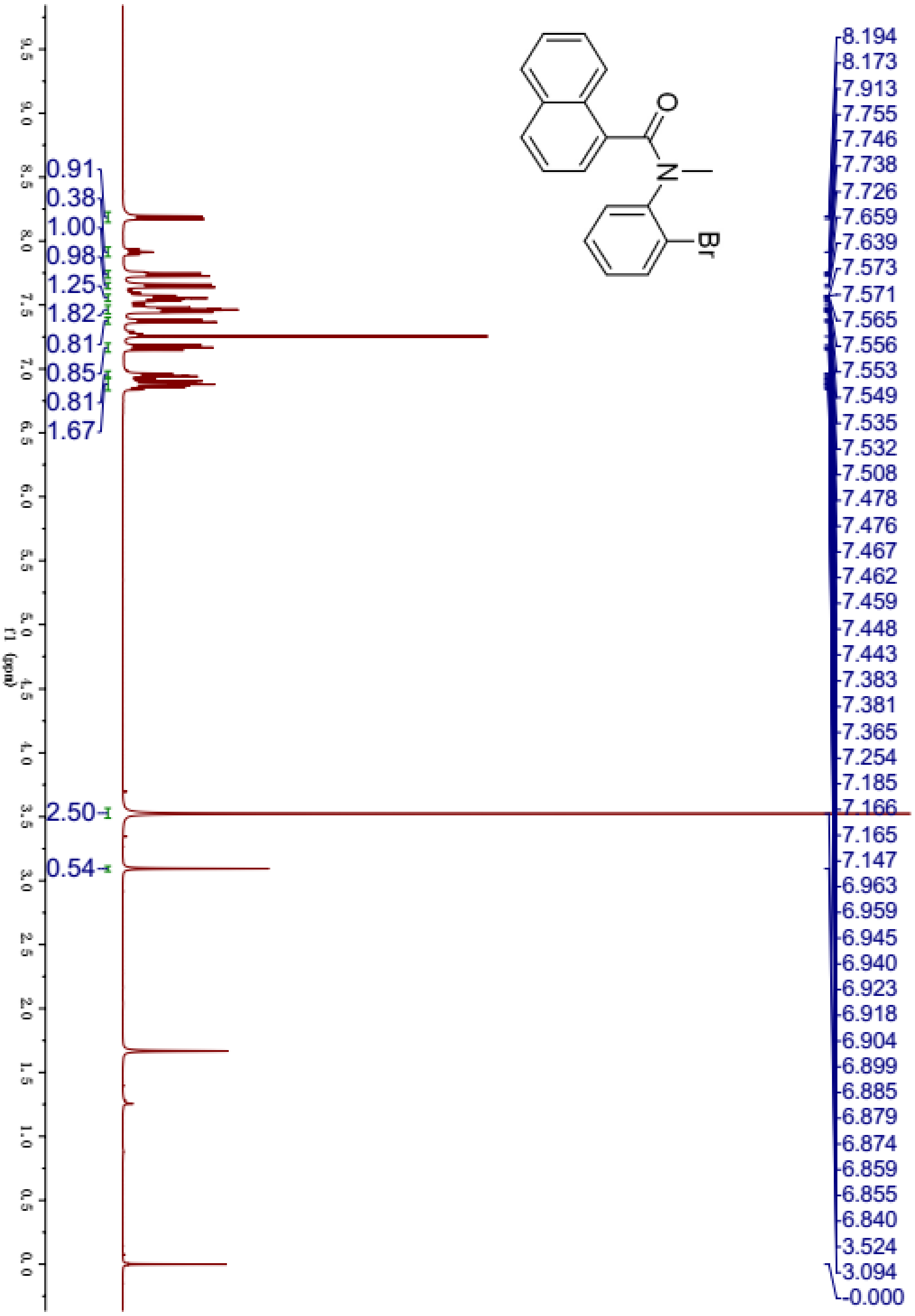
0.91
0.38
1.00
0.98
1.25
1.82
0.81
0.85
0.81
1.67
2.50
0.54
O
N
Br
8.194
8.173
7.913
7.755
7.746
7.738
7.726
7.659
7.639
7.573
7.571
7.565
7.556
7.553
7.549
7.535
7.532
7.508
7.478
7.476
7.467
7.462
7.459
7.448
7.443
7.383
7.381
7.365
7.254
7.185
7.166
7.165
7.147
6.963
6.959
6.945
6.940
6.923
6.918
6.904
6.899
6.885
6.879
6.874
6.859
6.855
6.840
3.524
3.094
-0.000

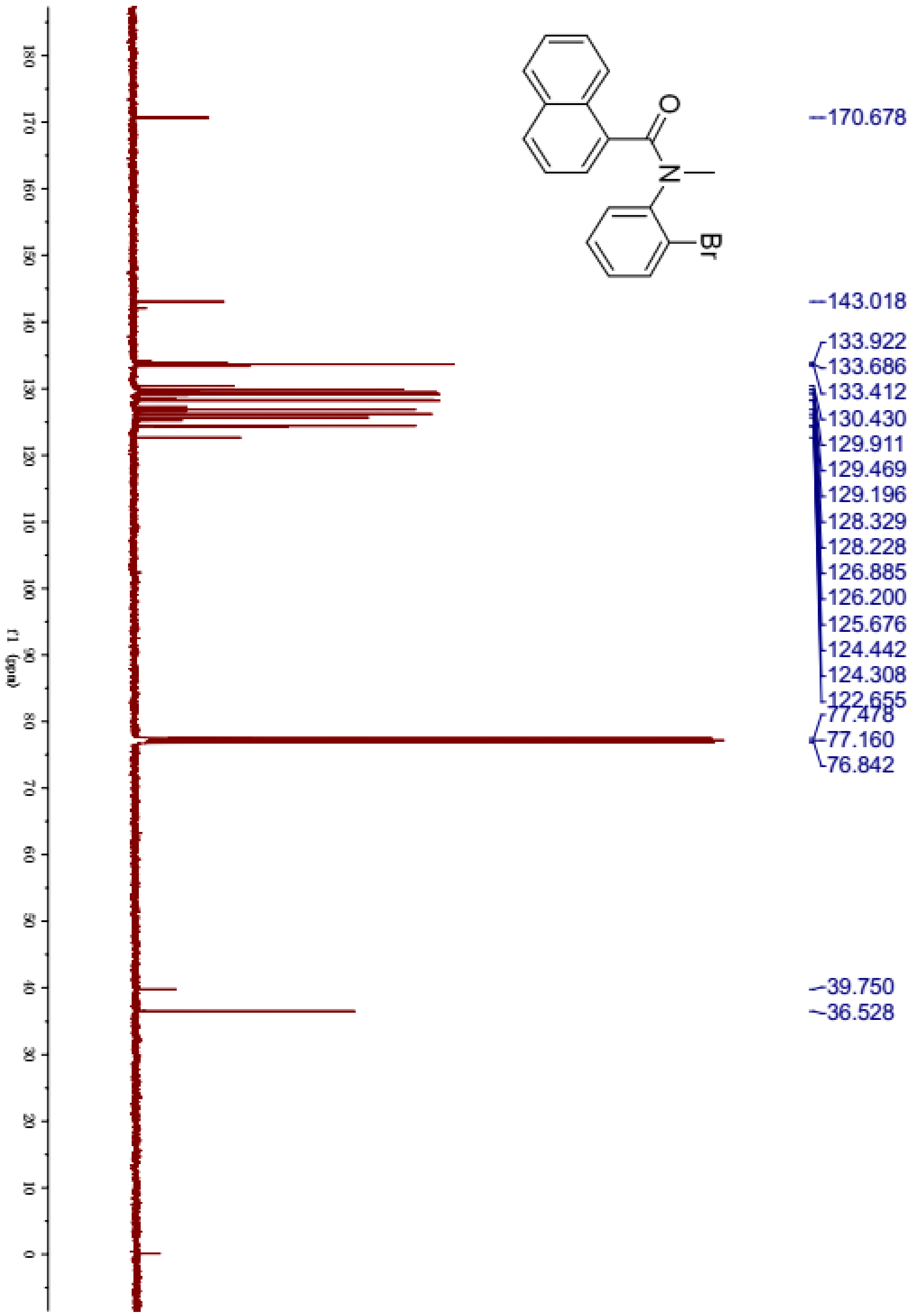
O
N
Br
170.678
143.018
133.922
133.686
133.412
130.430
129.911
129.469
129.196
128.329
128.228
126.885
126.200
125.676
124.442
124.308
122.655
77.478
77.160
76.842
39.750
36.528

1b

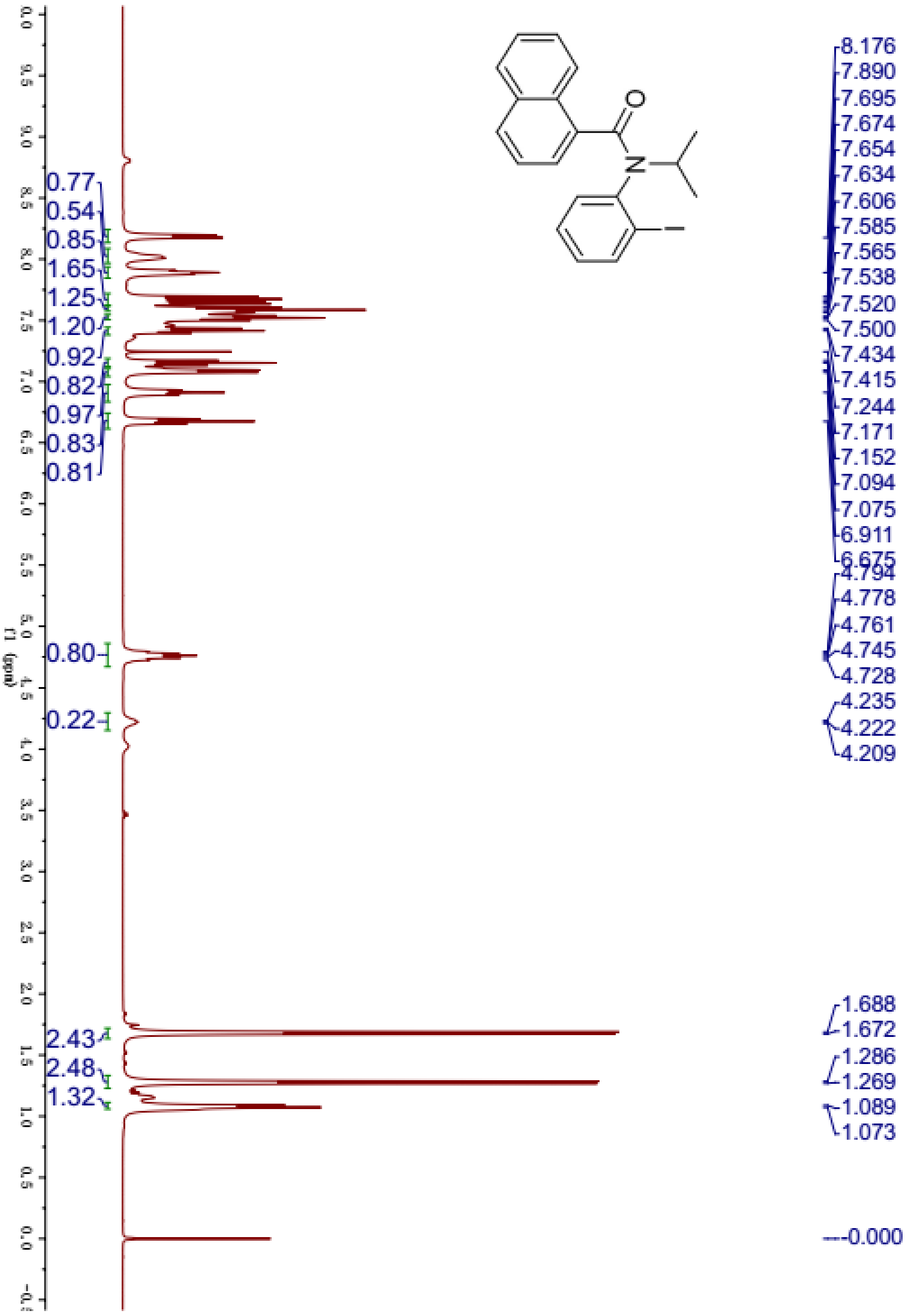
8.176
7.890
7.695
7.674
7.654
7.634
7.606
7.585
7.565
7.538
7.520
7.500
7.434
7.415
7.244
7.171
7.152
7.094
7.075
6.911
6.675
4.794
4.778
4.761
4.745
4.728
4.235
4.222
4.209
1.688
1.672
1.286
1.269
1.089
1.073
-0.000
0.77
0.54
0.85
1.65
1.25
1.20
0.92
0.82
0.97
0.83
0.81
0.80
0.22
2.43
2.48
1.32
f1 (ppm)
O
N

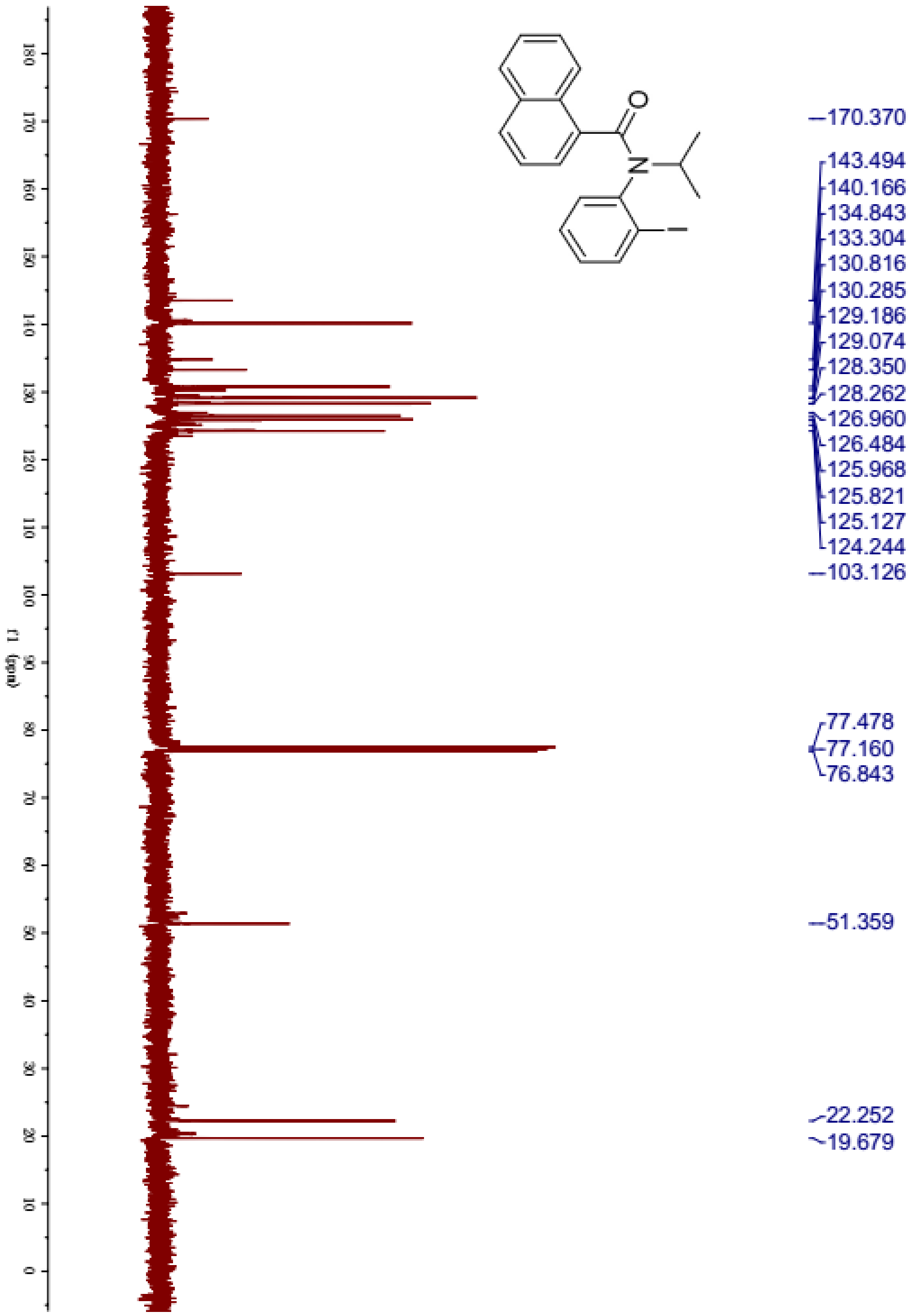
170.370
143.494
140.166
134.843
133.304
130.816
130.285
129.186
129.074
128.350
128.262
126.960
126.484
125.968
125.821
125.127
124.244
103.126
77.478
77.160
76.843
51.359
22.252
19.679
O
N
f1 (ppm)

1c

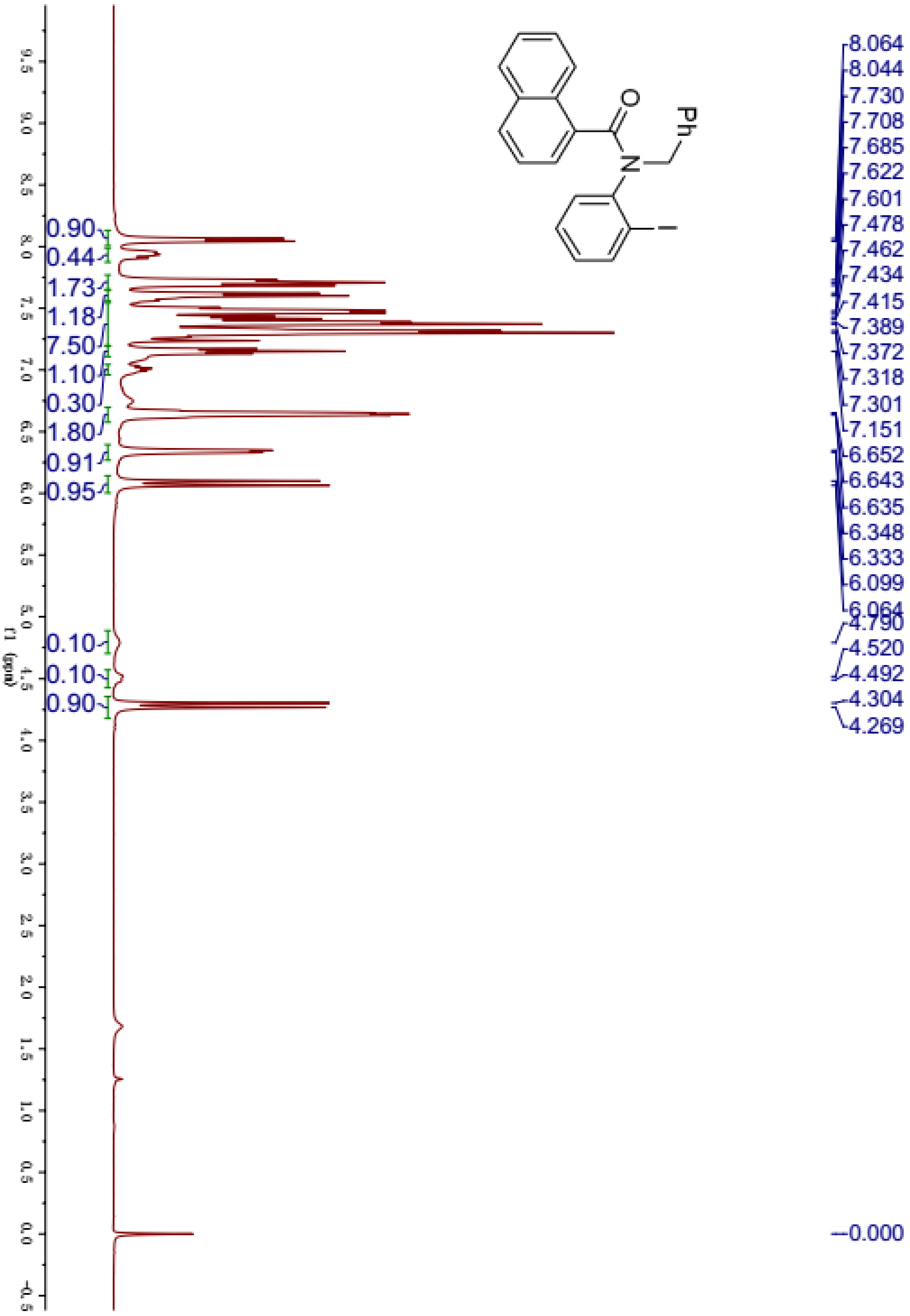
8.064
8.044
7.730
7.708
7.685
7.622
7.601
7.478
7.462
7.434
7.415
7.389
7.372
7.318
7.301
7.151
6.652
6.643
6.635
6.348
6.333
6.099
6.064
4.790
4.520
4.492
4.304
4.269
0.000
0.90
0.44
1.73
1.18
7.50
1.10
0.30
1.80
0.91
0.95
0.10
0.10
0.90
f1 (ppm)
O
N
Ph
I

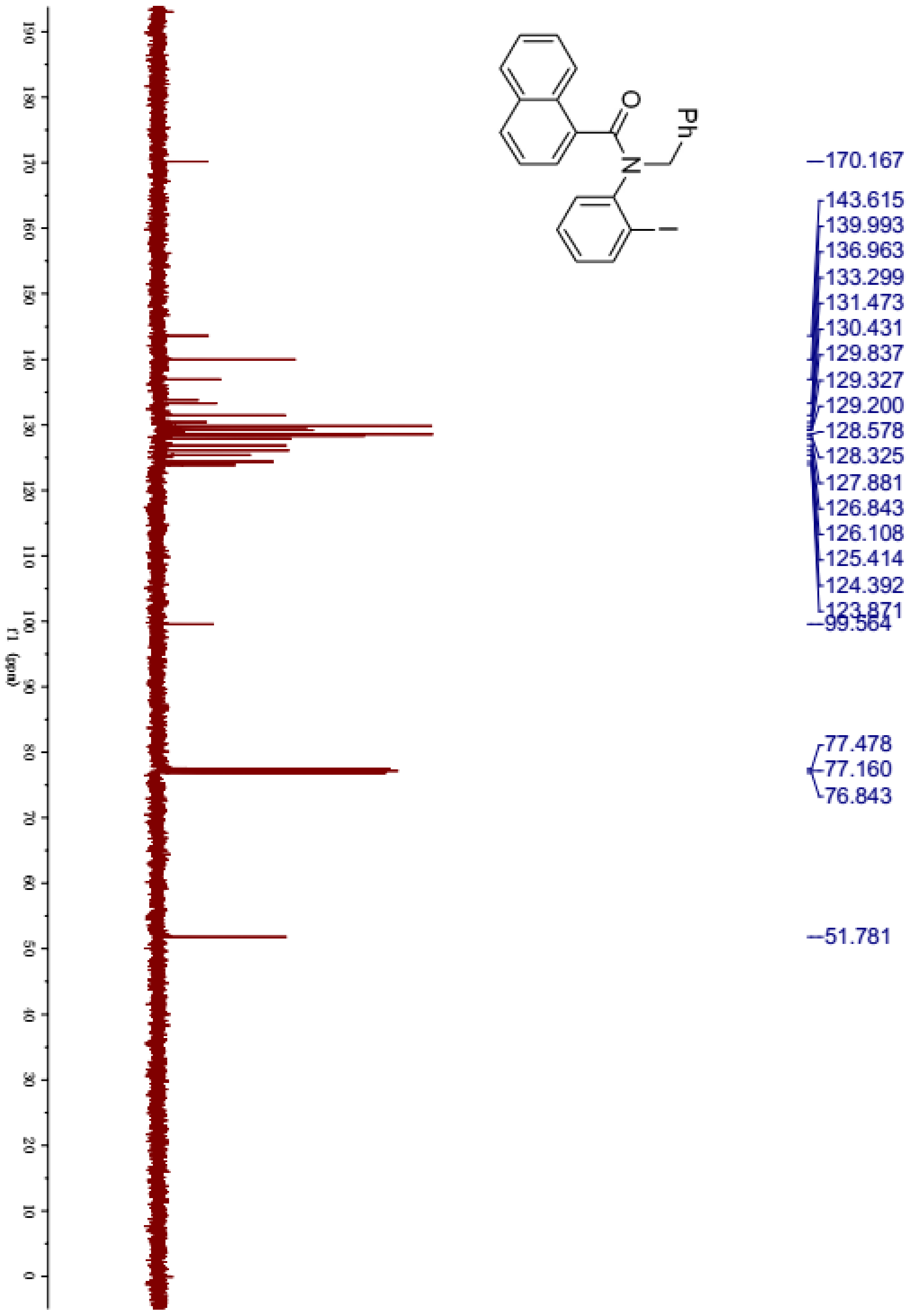
170.167
143.615
139.993
136.963
133.299
131.473
130.431
129.837
129.327
129.200
128.578
128.325
127.881
126.843
126.108
125.414
124.392
123.871
99.564
77.478
77.160
76.843
51.781
f1 (ppm)

1d

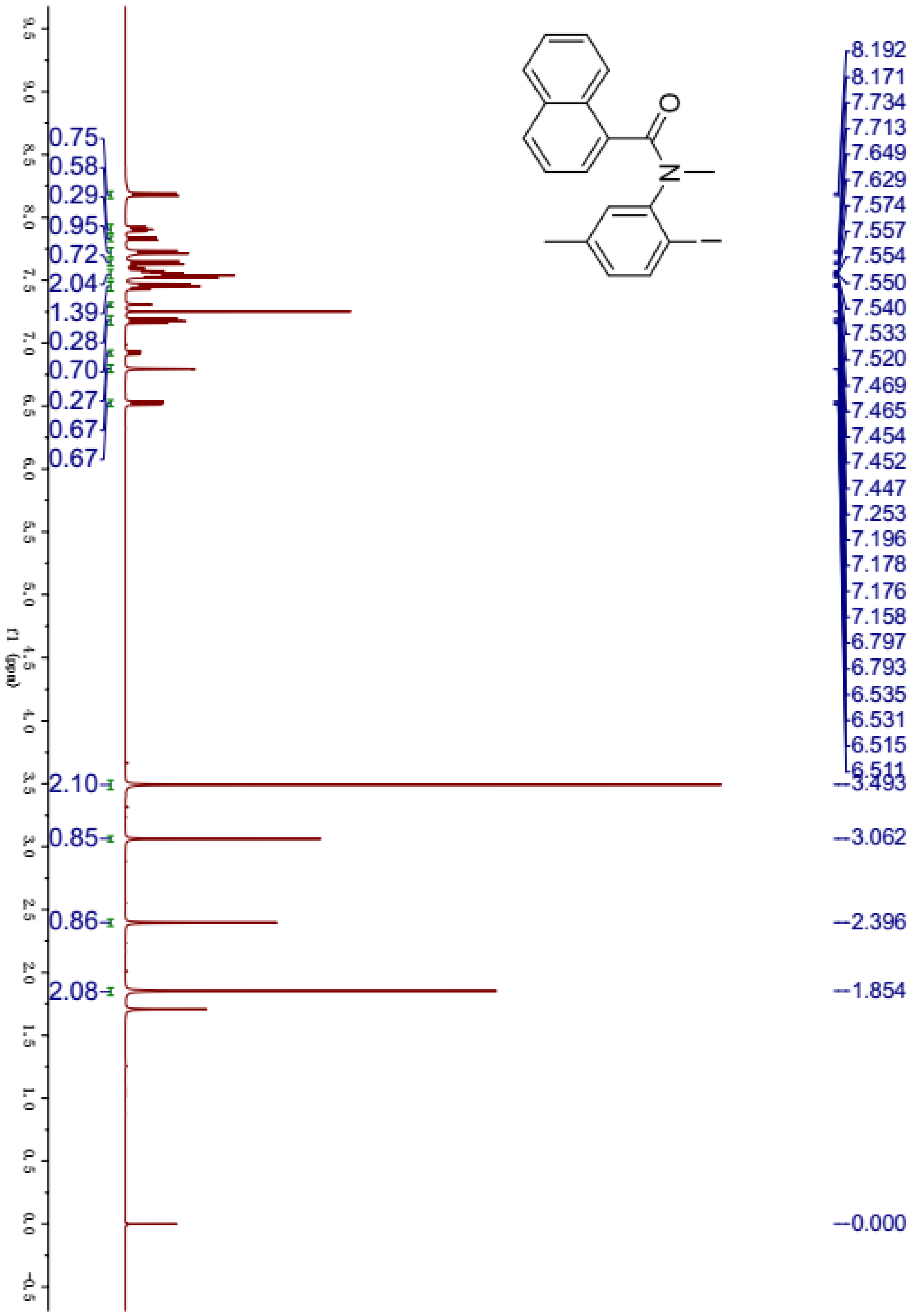
8.192
8.171
7.734
7.713
7.649
7.629
7.574
7.557
7.554
7.550
7.540
7.533
7.520
7.469
7.465
7.454
7.452
7.447
7.253
7.196
7.178
7.176
7.158
6.797
6.793
6.535
6.531
6.515
6.511
3.493
3.062
2.396
1.854
0.000
0.75
0.58
0.29
0.95
0.72
2.04
1.39
0.28
0.70
0.27
0.67
0.67
2.10
0.85
0.86
2.08
f1 (ppm)

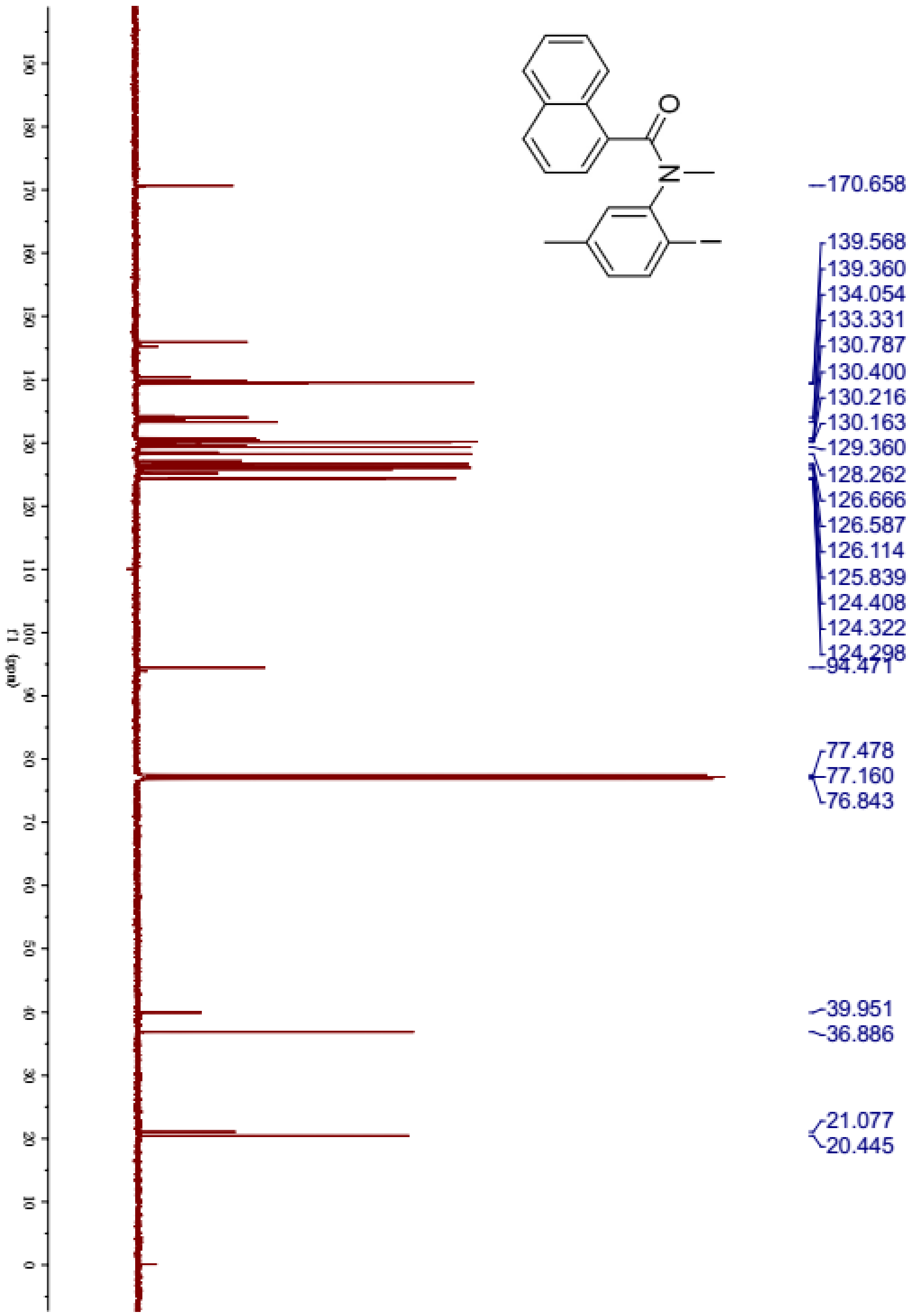

170.658
139.568
139.360
134.054
133.331
130.787
130.400
130.216
130.163
129.360
128.262
126.666
126.587
126.114
125.839
124.408
124.322
124.298
94.471
77.478
77.160
76.843
39.951
36.886
21.077
20.445
f1 (ppm)

1e

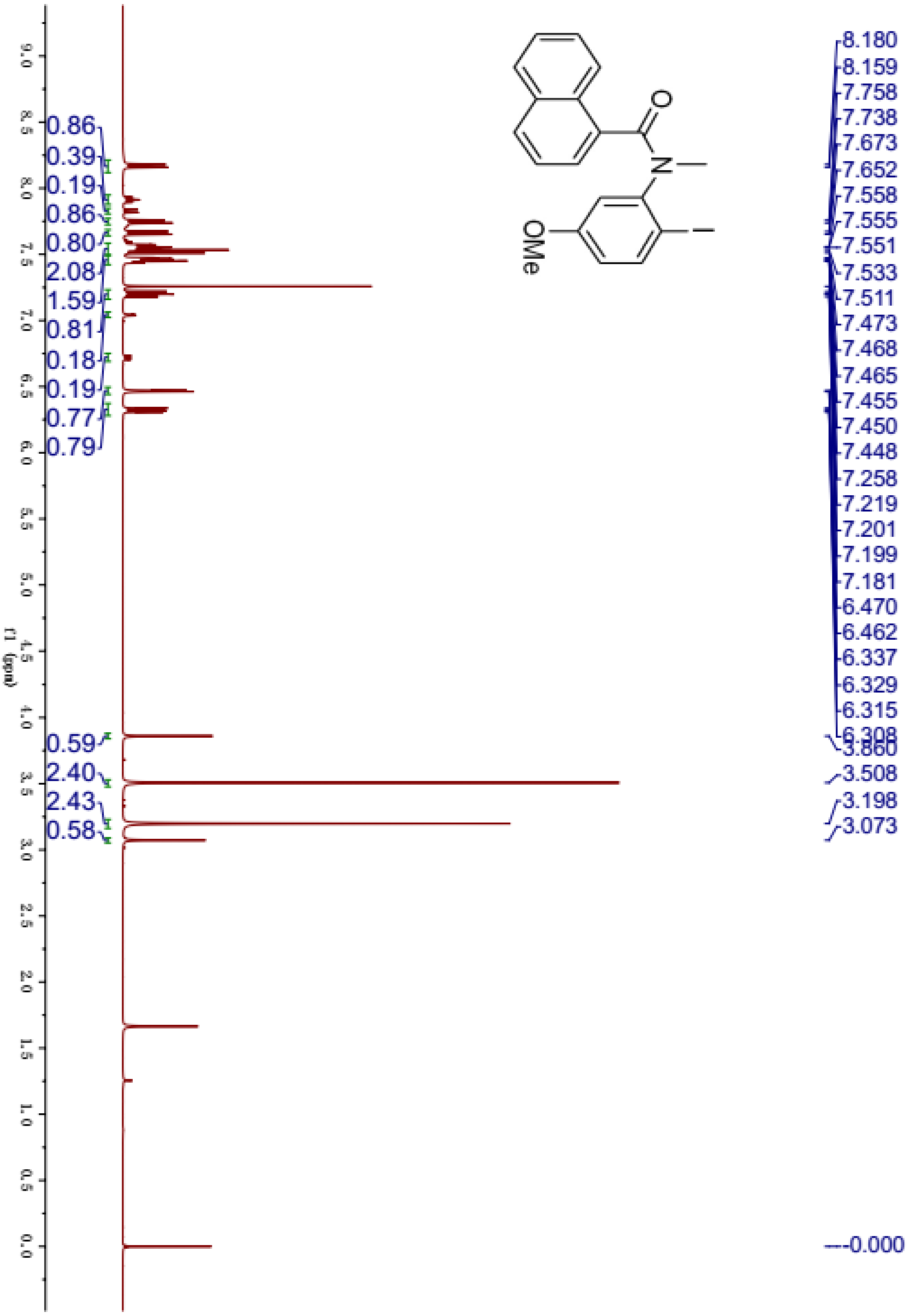
8.180
8.159
7.758
7.738
7.673
7.652
7.558
7.555
7.551
7.533
7.511
7.473
7.468
7.465
7.455
7.450
7.448
7.258
7.219
7.201
7.199
7.181
6.470
6.462
6.337
6.329
6.315
6.308
3.860
3.508
3.198
3.073
-0.000
0.86
0.39
0.19
0.86
0.80
2.08
1.59
0.81
0.18
0.19
0.77
0.79
0.59
2.40
2.43
0.58
O
N
I
OMe
f1 (ppm)

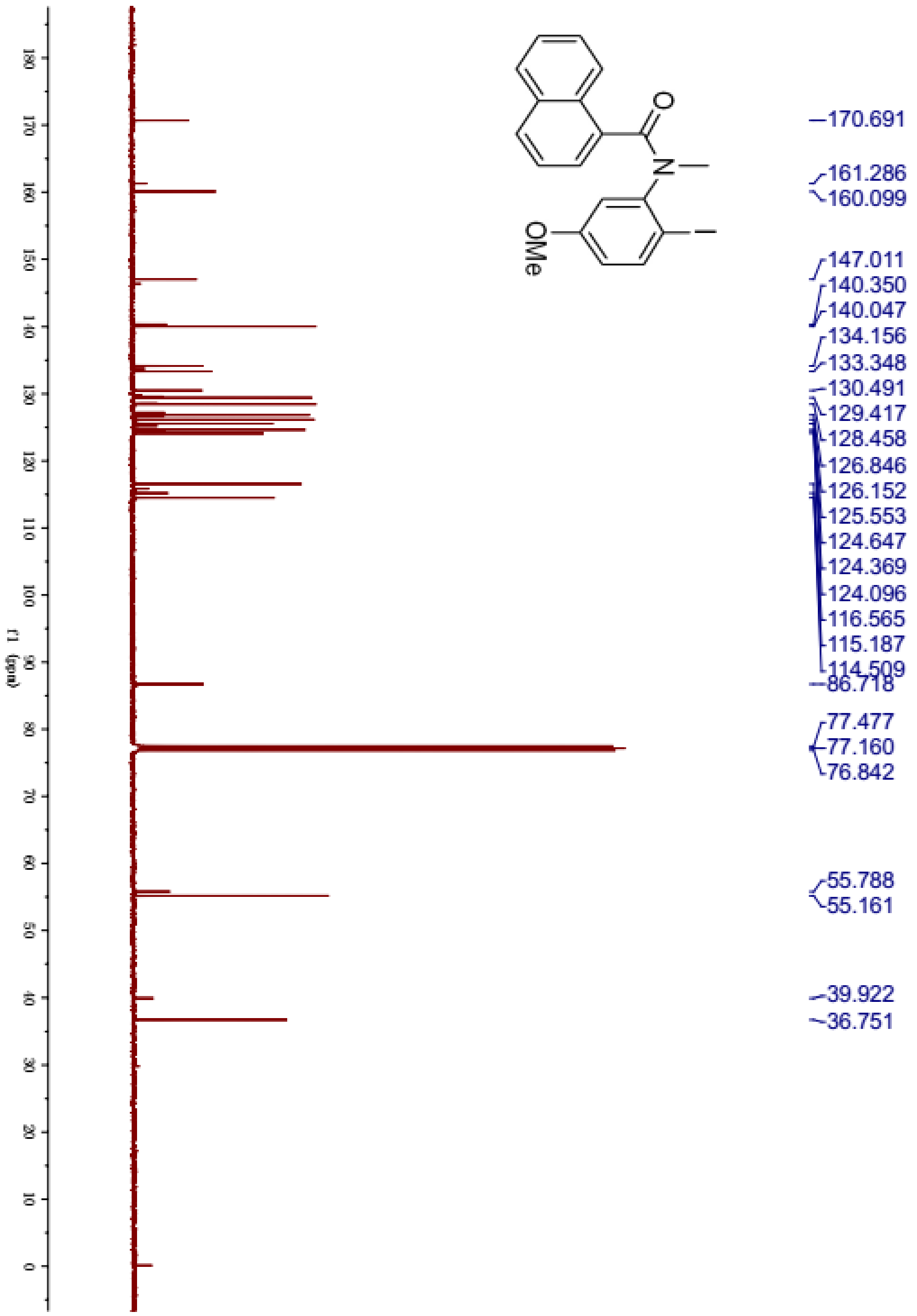
OMe
O
N
170.691
161.286
160.099
147.011
140.350
140.047
134.156
133.348
130.491
129.417
128.458
126.846
126.152
125.553
124.647
124.369
124.096
116.565
115.187
114.509
86.718
77.477
77.160
76.842
55.788
55.161
39.922
36.751
f1 (ppm)

1f

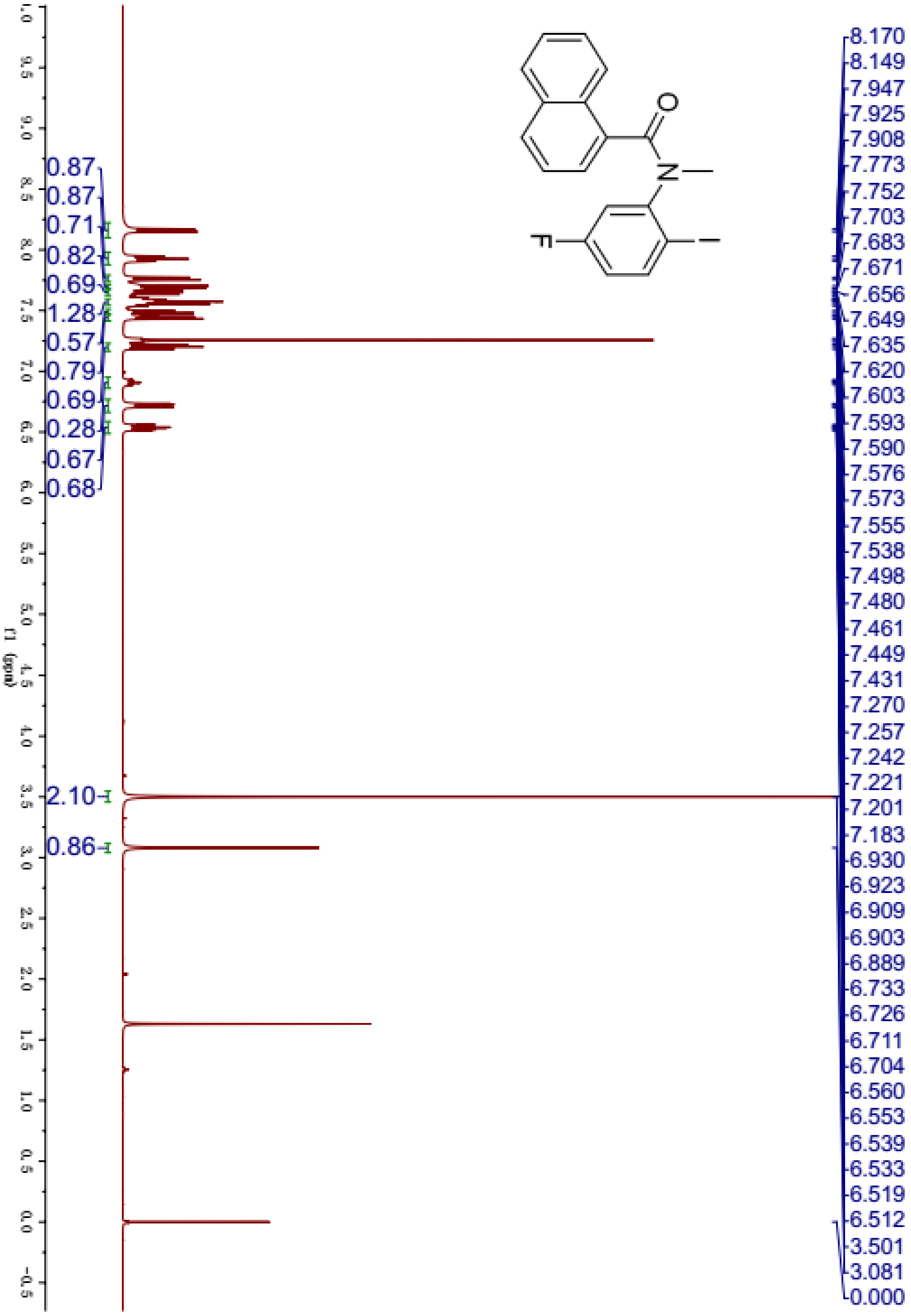
8.170
8.149
7.947
7.925
7.908
7.773
7.752
7.703
7.683
7.671
7.656
7.649
7.635
7.620
7.603
7.593
7.590
7.576
7.573
7.555
7.538
7.498
7.480
7.461
7.449
7.431
7.270
7.257
7.242
7.221
7.201
7.183
6.930
6.923
6.909
6.903
6.889
6.733
6.726
6.711
6.704
6.560
6.553
6.539
6.533
6.519
6.512
3.501
3.081
0.000
0.87
0.87
0.71
0.82
0.69
1.28
0.57
0.79
0.69
0.28
0.67
0.68
2.10
0.86
O
N
F
I

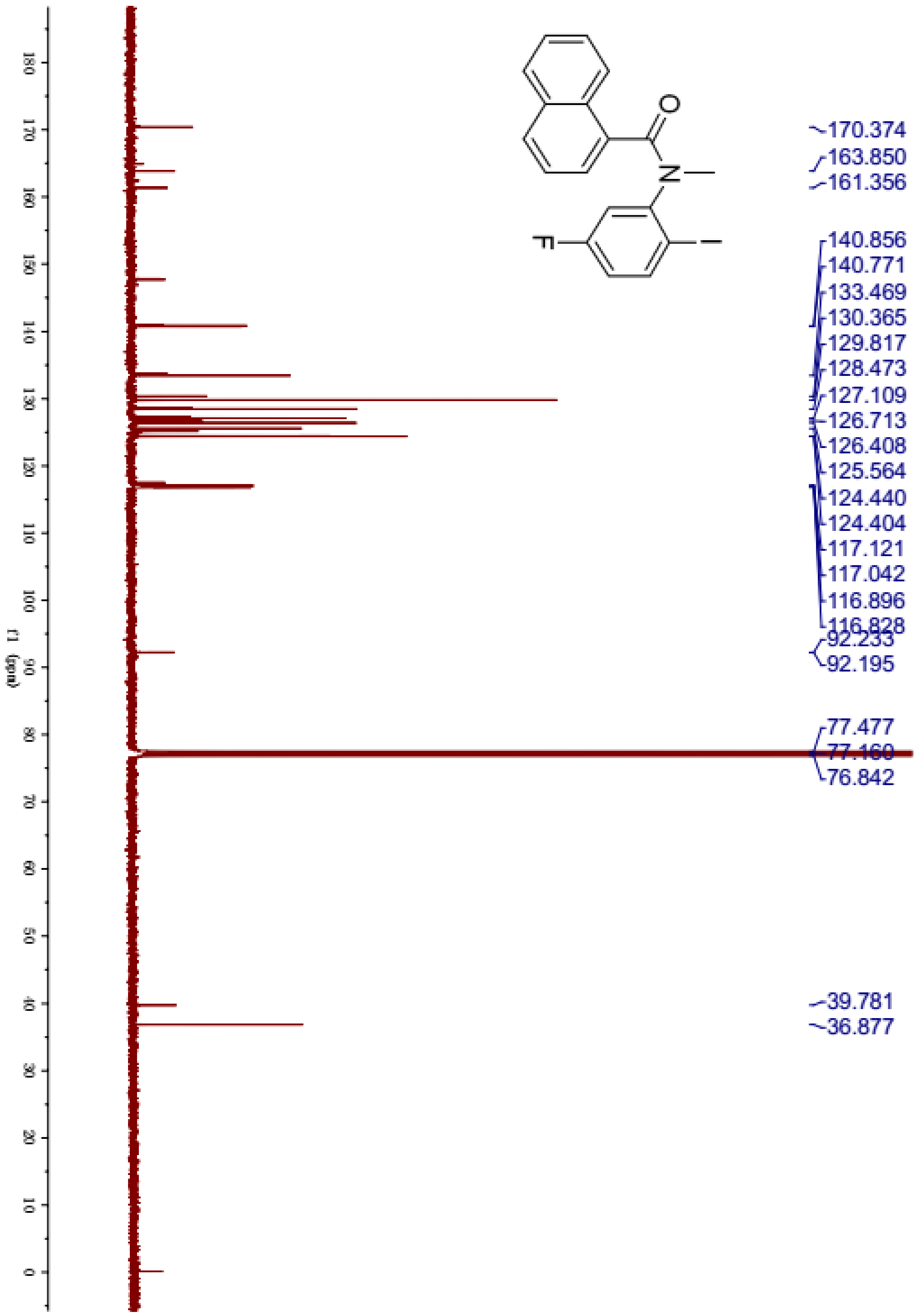

O
N
F
I
170.374
163.850
161.356
140.856
140.771
133.469
130.365
129.817
128.473
127.109
126.713
126.408
125.564
124.440
124.404
117.121
117.042
116.896
116.828
92.233
92.195
77.477
77.160
76.842
39.781
36.877

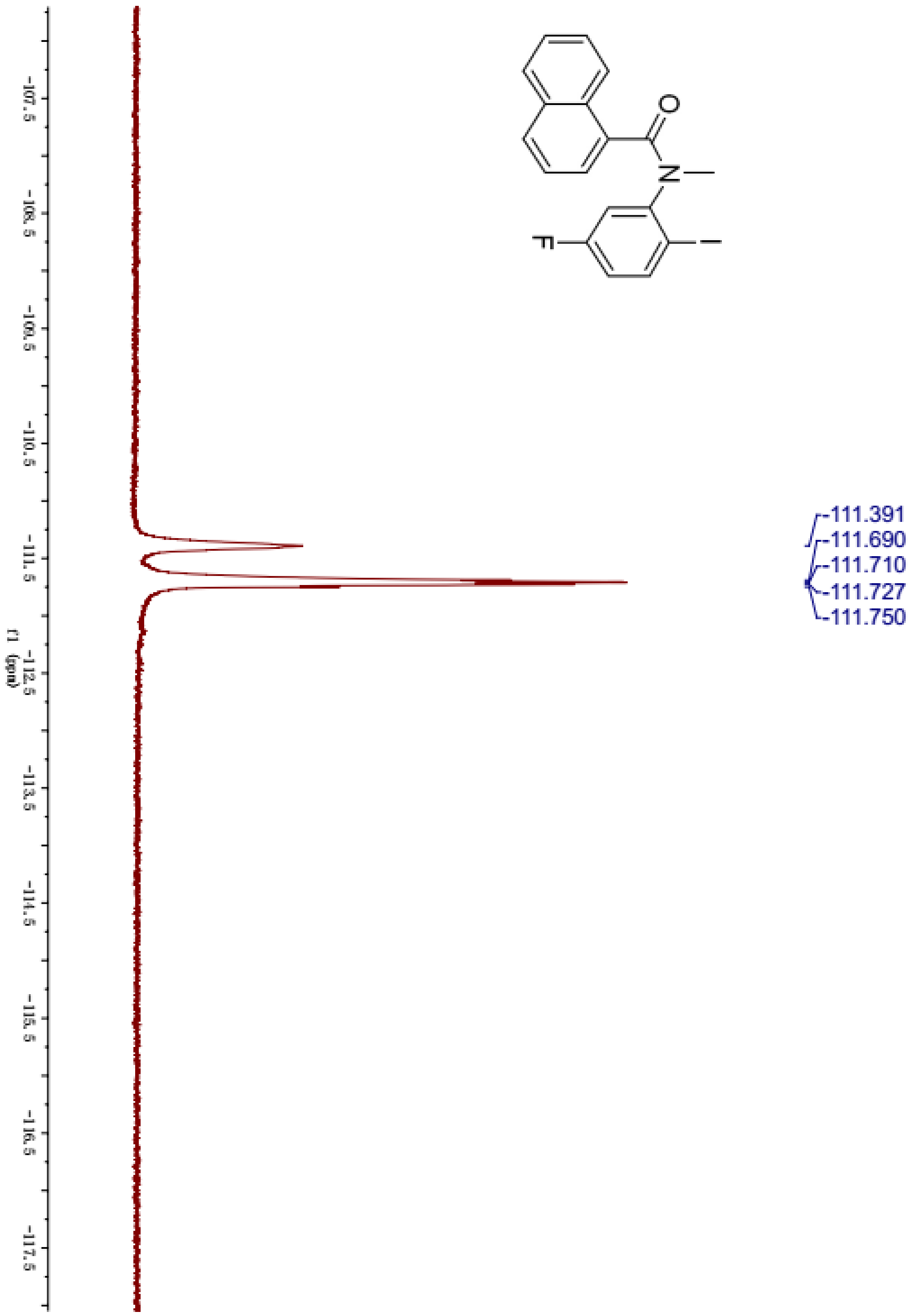

-111.391
-111.690
-111.710
-111.727
-111.750
-107.5
-108.5
-109.5
-110.5
-111.5
-112.5
-113.5
-114.5
-115.5
-116.5
-117.5
f1 (ppm)
O
N
F
I

1g

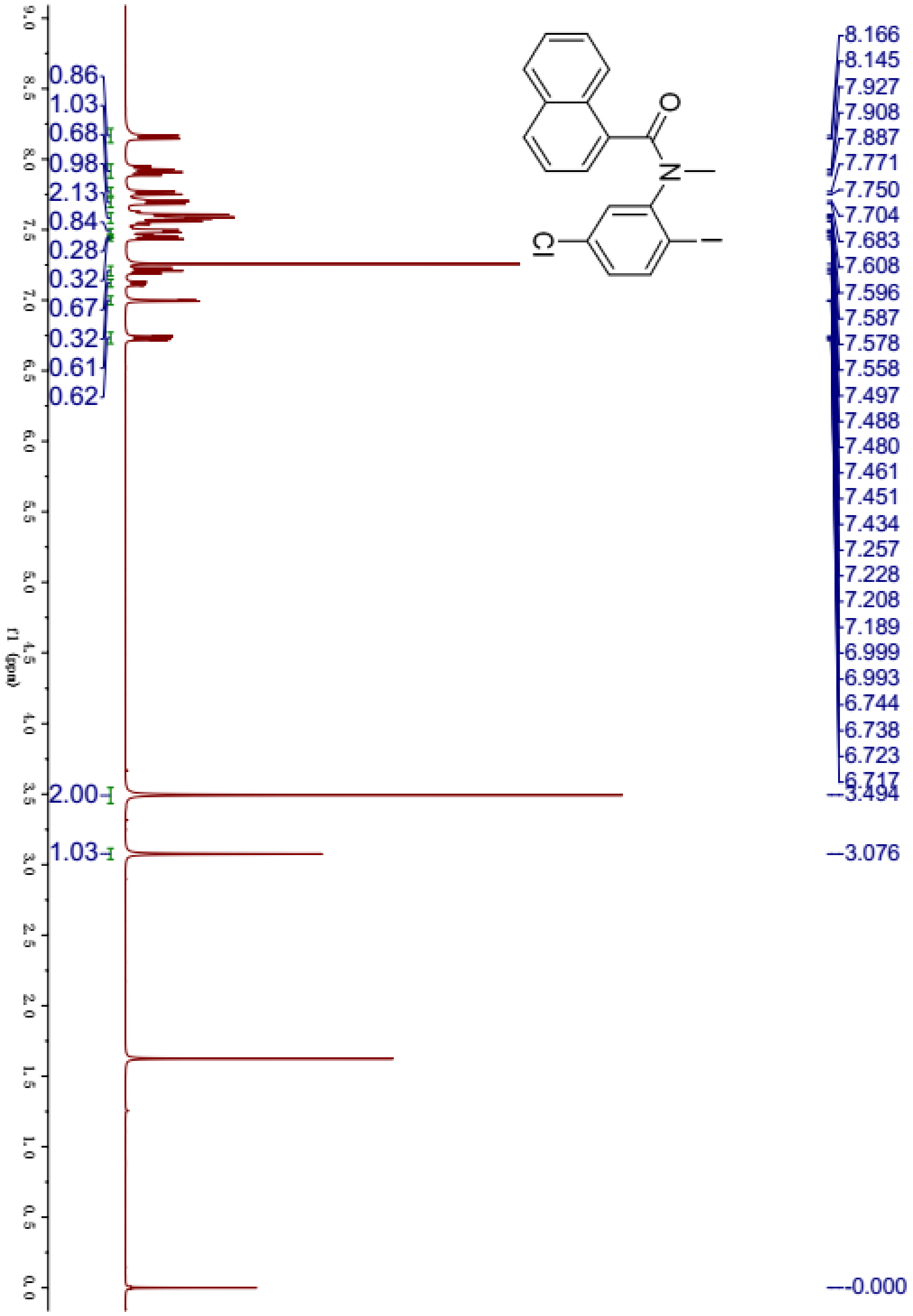
8.166
8.145
7.927
7.908
7.887
7.771
7.750
7.704
7.683
7.608
7.596
7.587
7.578
7.558
7.497
7.488
7.480
7.461
7.451
7.434
7.257
7.228
7.208
7.189
6.999
6.993
6.744
6.738
6.723
6.717
3.494
3.076
-0.000
0.86
1.03
0.68
0.98
2.13
0.84
0.28
0.32
0.67
0.32
0.61
0.62
2.00
1.03
O
N
Cl
I
f1 (ppm)

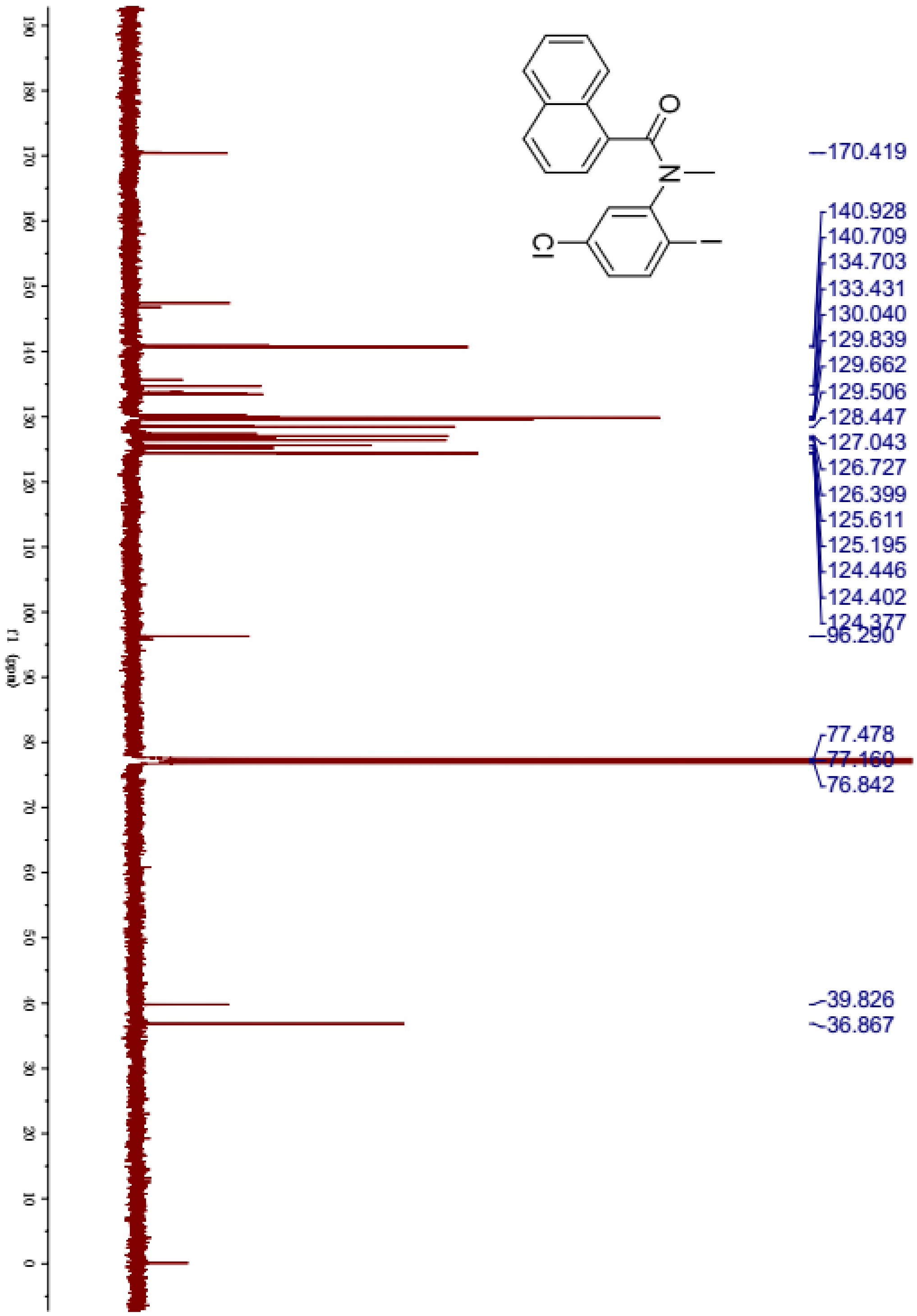
O
N
Cl
I
170.419
140.928
140.709
134.703
133.431
130.040
129.839
129.662
129.506
128.447
127.043
126.727
126.399
125.611
125.195
124.446
124.402
124.377
96.290
77.478
77.160
76.842
39.826
36.867

1h

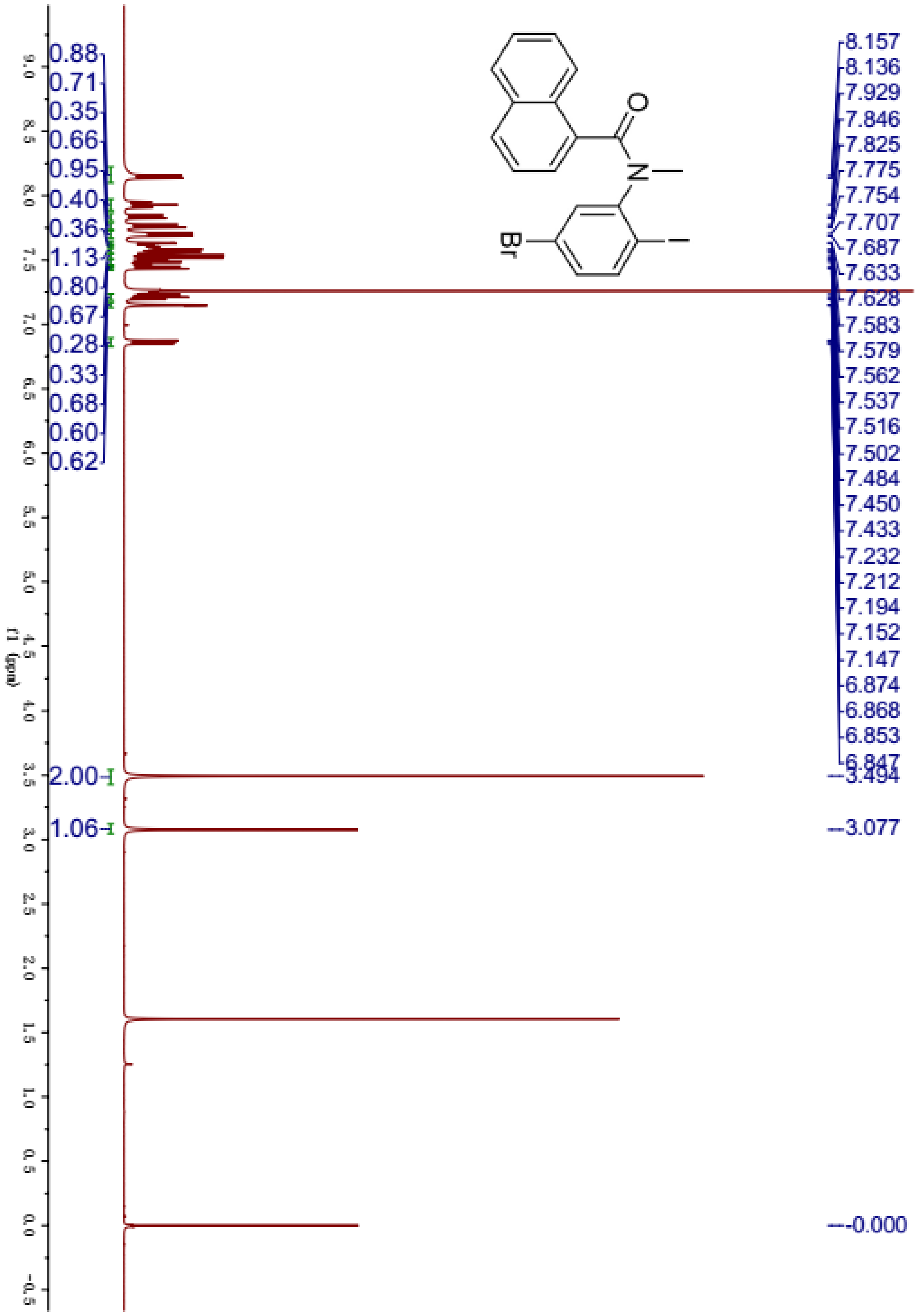
8.157
8.136
7.929
7.846
7.825
7.775
7.754
7.707
7.687
7.633
7.628
7.583
7.579
7.562
7.537
7.516
7.502
7.484
7.450
7.433
7.232
7.212
7.194
7.152
7.147
6.874
6.868
6.853
6.847
3.494
3.077
-0.000
0.88
0.71
0.35
0.66
0.95
0.40
0.36
1.13
0.80
0.67
0.28
0.33
0.68
0.60
0.62
2.00
1.06
Br
O
N
f1 (ppm)

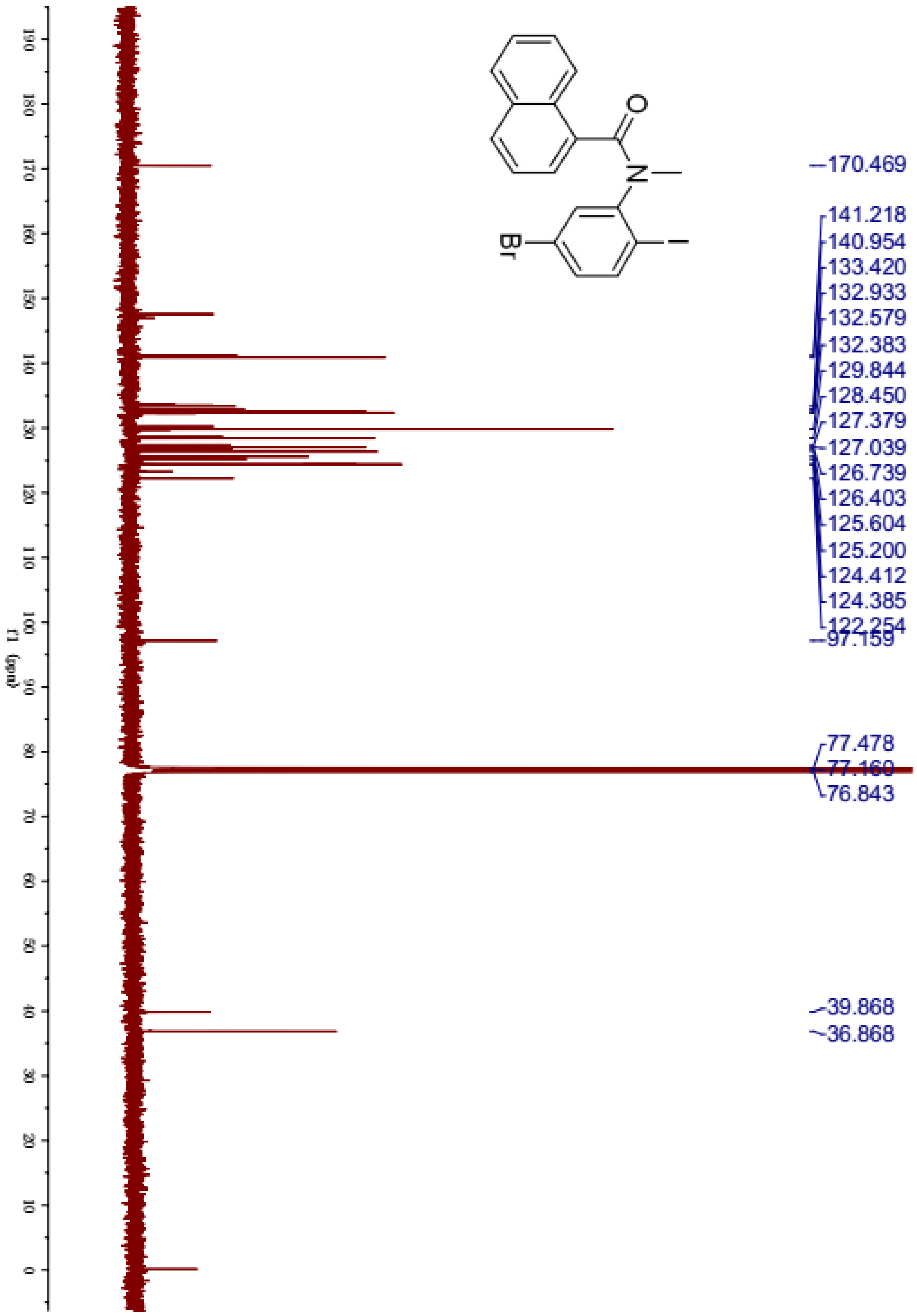

170.469
141.218
140.954
133.420
132.933
132.579
132.383
129.844
128.450
127.379
127.039
126.739
126.403
125.604
125.200
124.412
124.385
122.254
97.159
77.478
77.160
76.843
39.868
36.868
Br
O
N
f1 (ppm)

1i

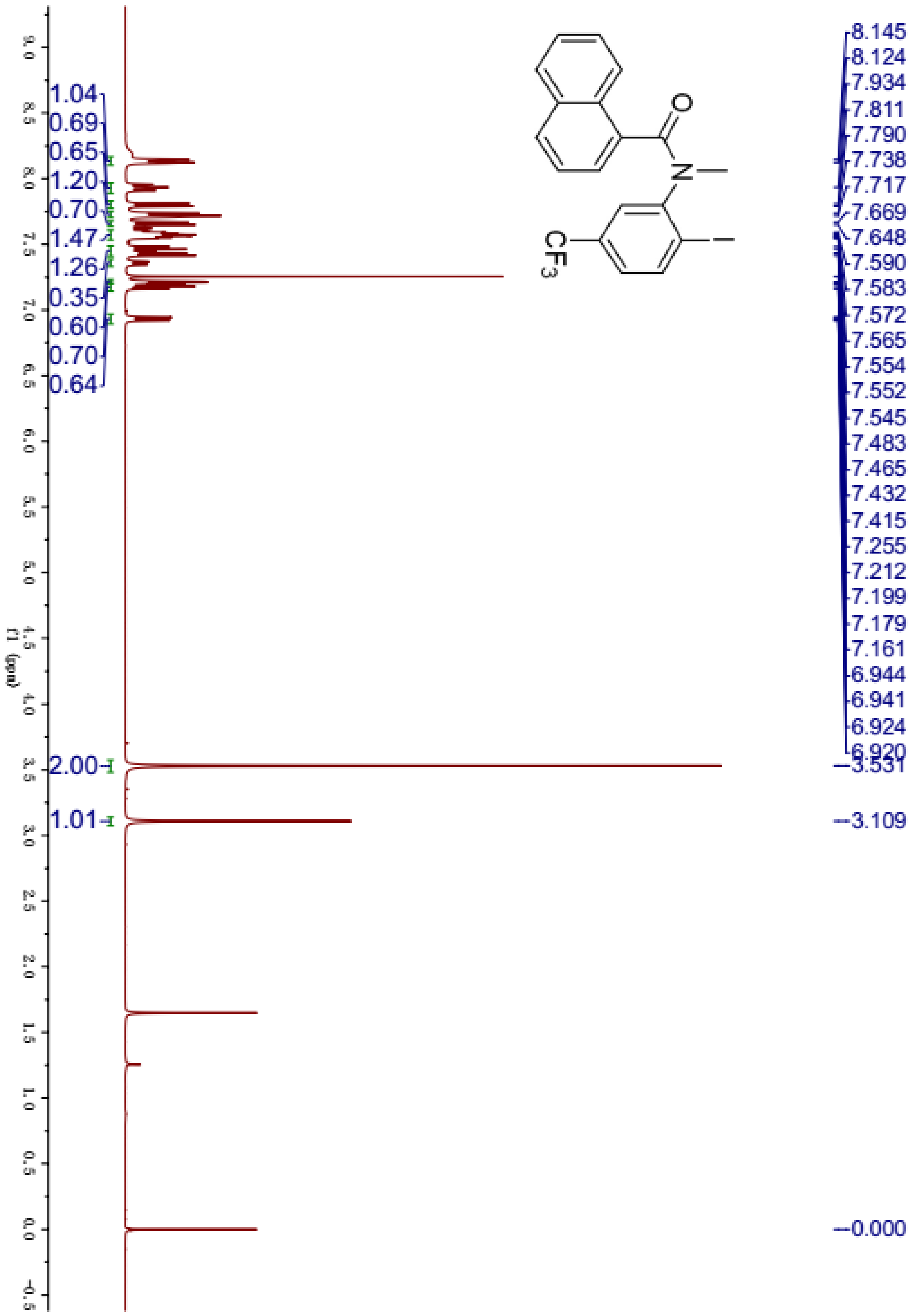

8.145
8.124
7.934
7.811
7.790
7.738
7.717
7.669
7.648
7.590
7.583
7.572
7.565
7.554
7.552
7.545
7.483
7.465
7.432
7.415
7.255
7.212
7.199
7.179
7.161
6.944
6.941
6.924
6.920
3.531
3.109
0.000
1.04
0.69
0.65
1.20
0.70
1.47
1.26
0.35
0.60
0.70
0.64
2.00
1.01
f1 (ppm)
O
N
I
CF3

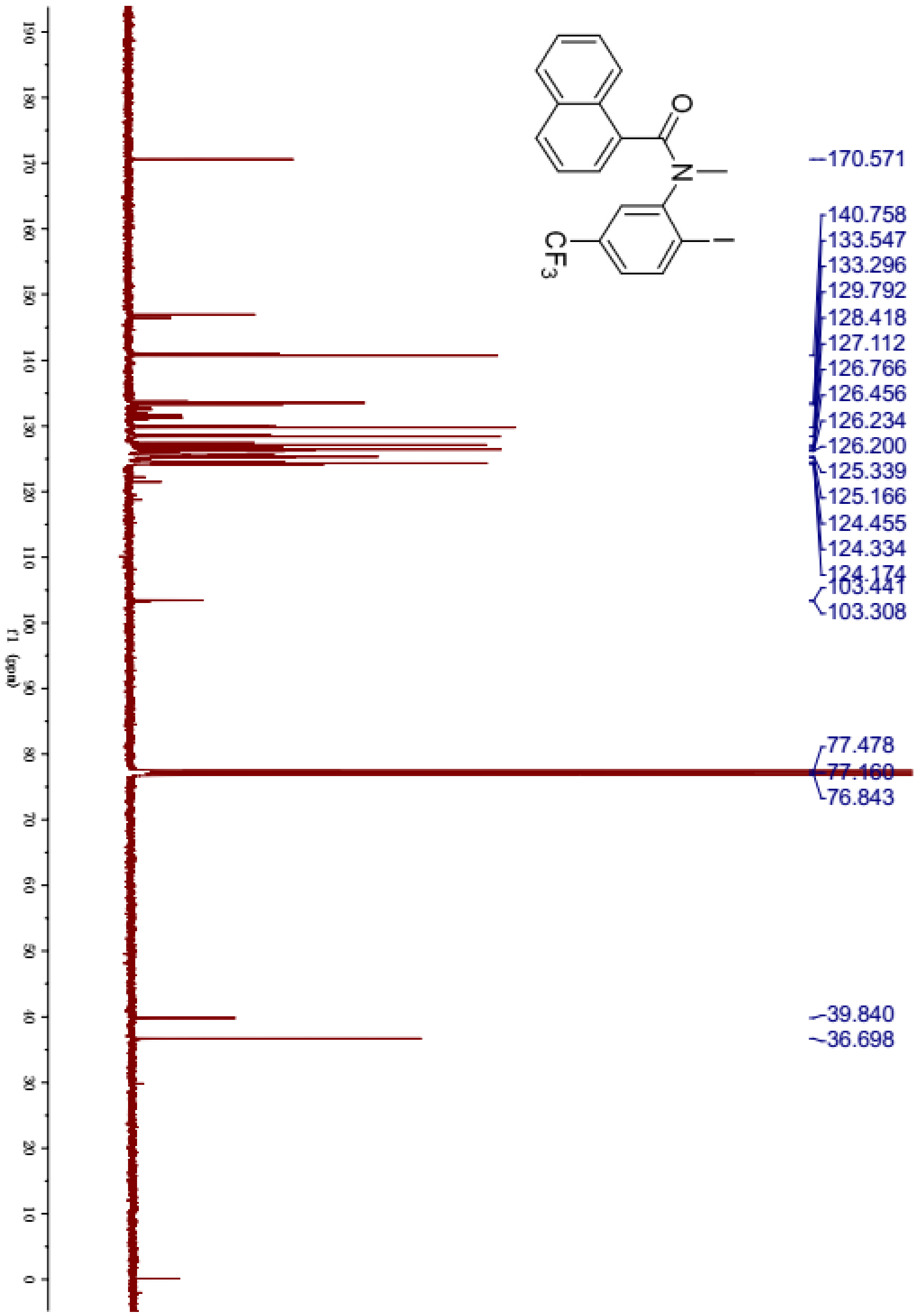

O
N
I
CF3
170.571
140.758
133.547
133.296
129.792
128.418
127.112
126.766
126.456
126.234
126.200
125.339
125.166
124.455
124.334
124.174
103.441
103.308
77.478
77.160
76.843
39.840
36.698
f1 (ppm)

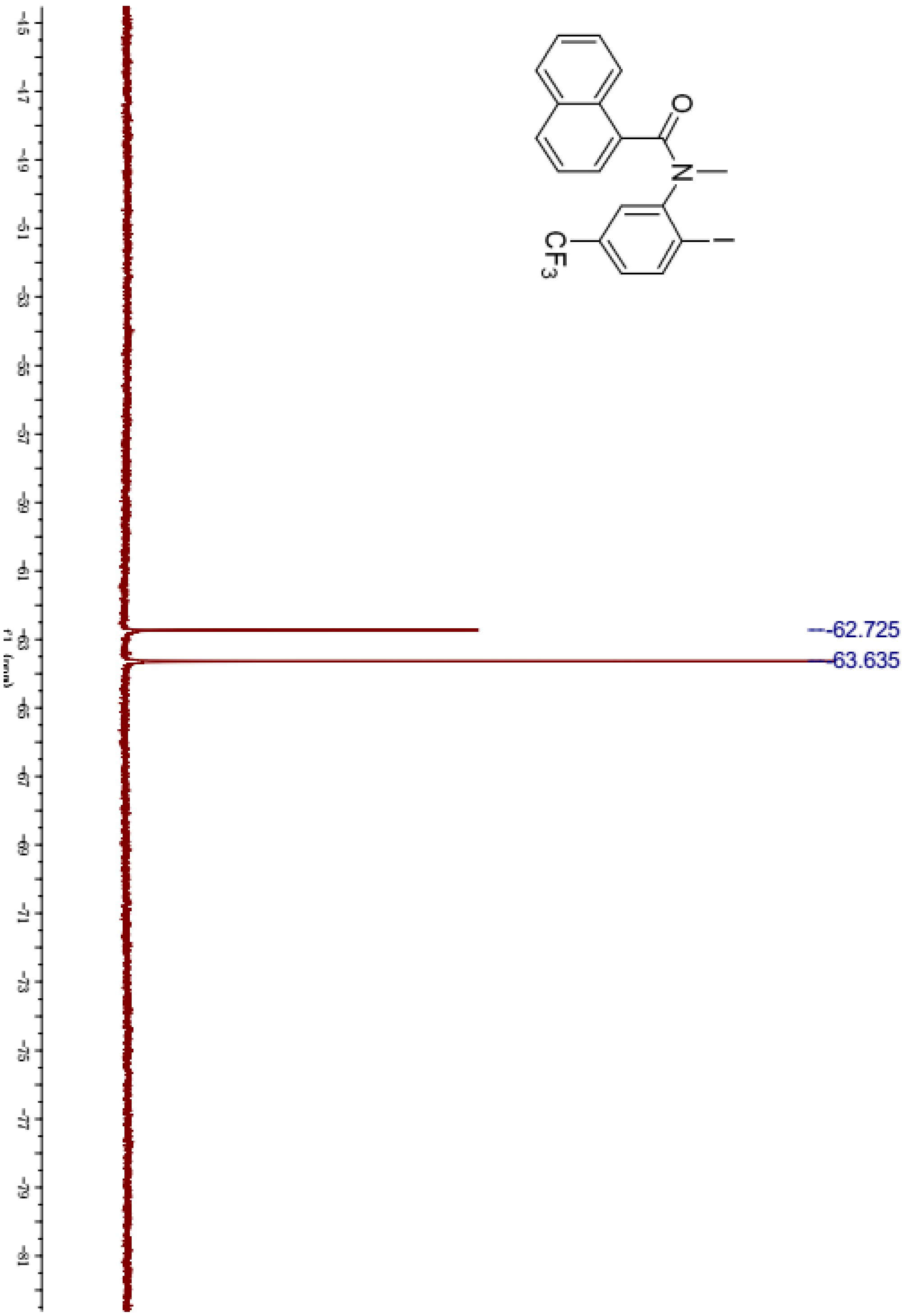
-62.725
-63.635
CF3
O
N
I

1j

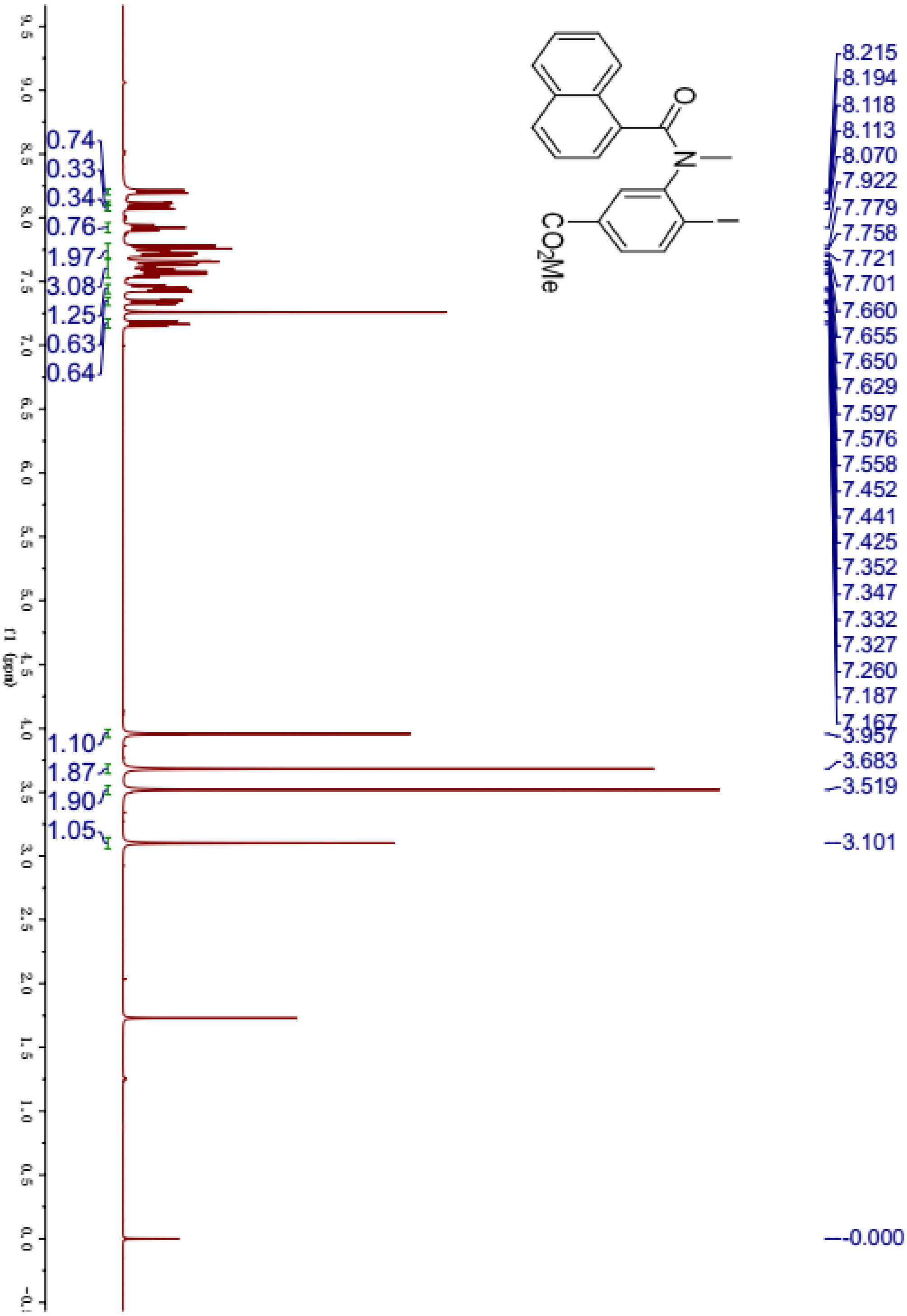

CO2Me
O
N
8.215
8.194
8.118
8.113
8.070
7.922
7.779
7.758
7.721
7.701
7.660
7.655
7.650
7.629
7.597
7.576
7.558
7.452
7.441
7.425
7.352
7.347
7.332
7.327
7.260
7.187
7.167
3.957
3.683
3.519
3.101
-0.000
0.74
0.33
0.34
0.76
1.97
3.08
1.25
0.63
0.64
1.10
1.87
1.90
1.05
f1 (ppm)

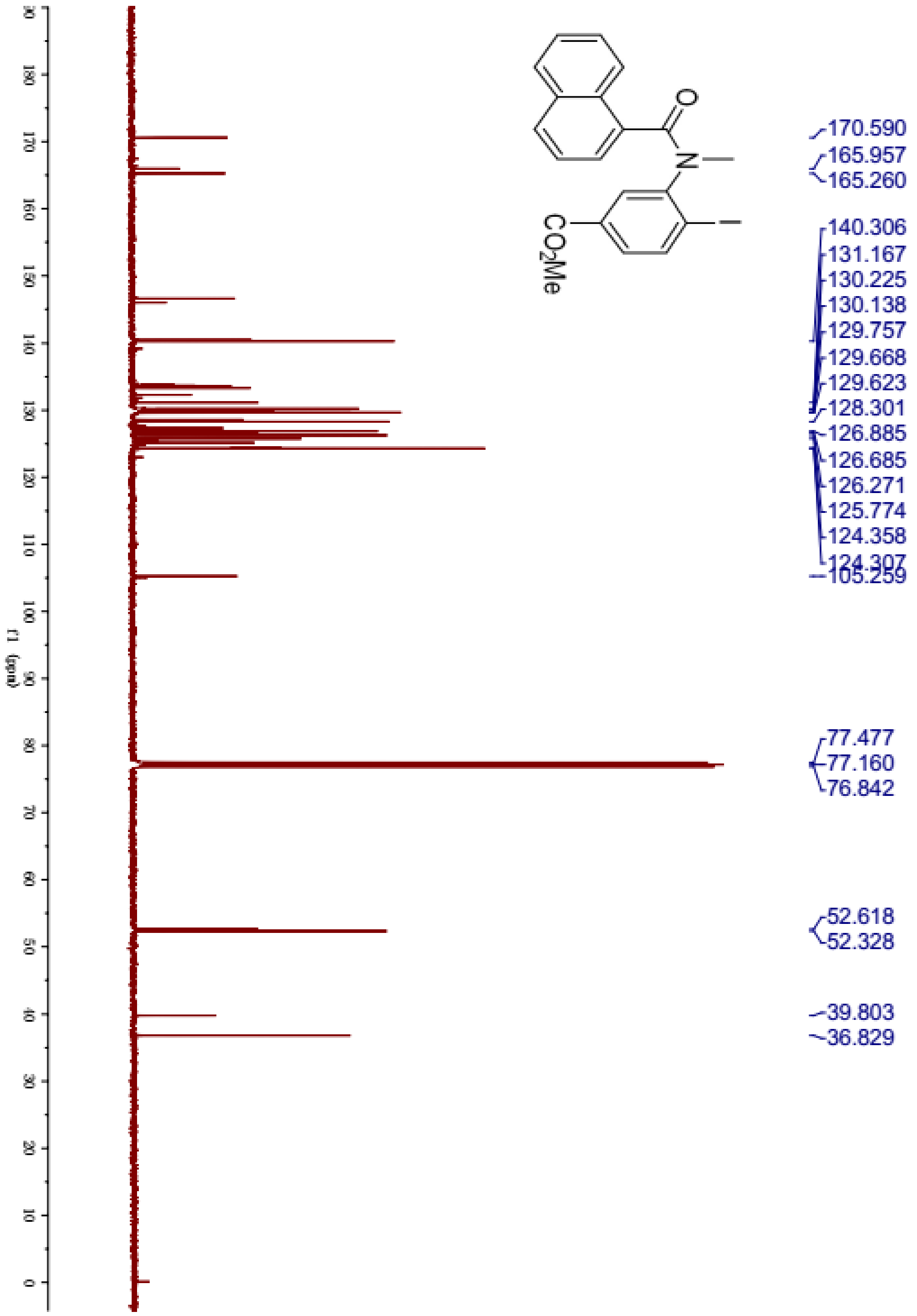
170.590
165.957
165.260
140.306
131.167
130.225
130.138
129.757
129.668
129.623
128.301
126.885
126.685
126.271
125.774
124.358
124.307
105.259
77.477
77.160
76.842
52.618
52.328
39.803
36.829
O
N
I
CO2Me
f1 (ppm)

1k

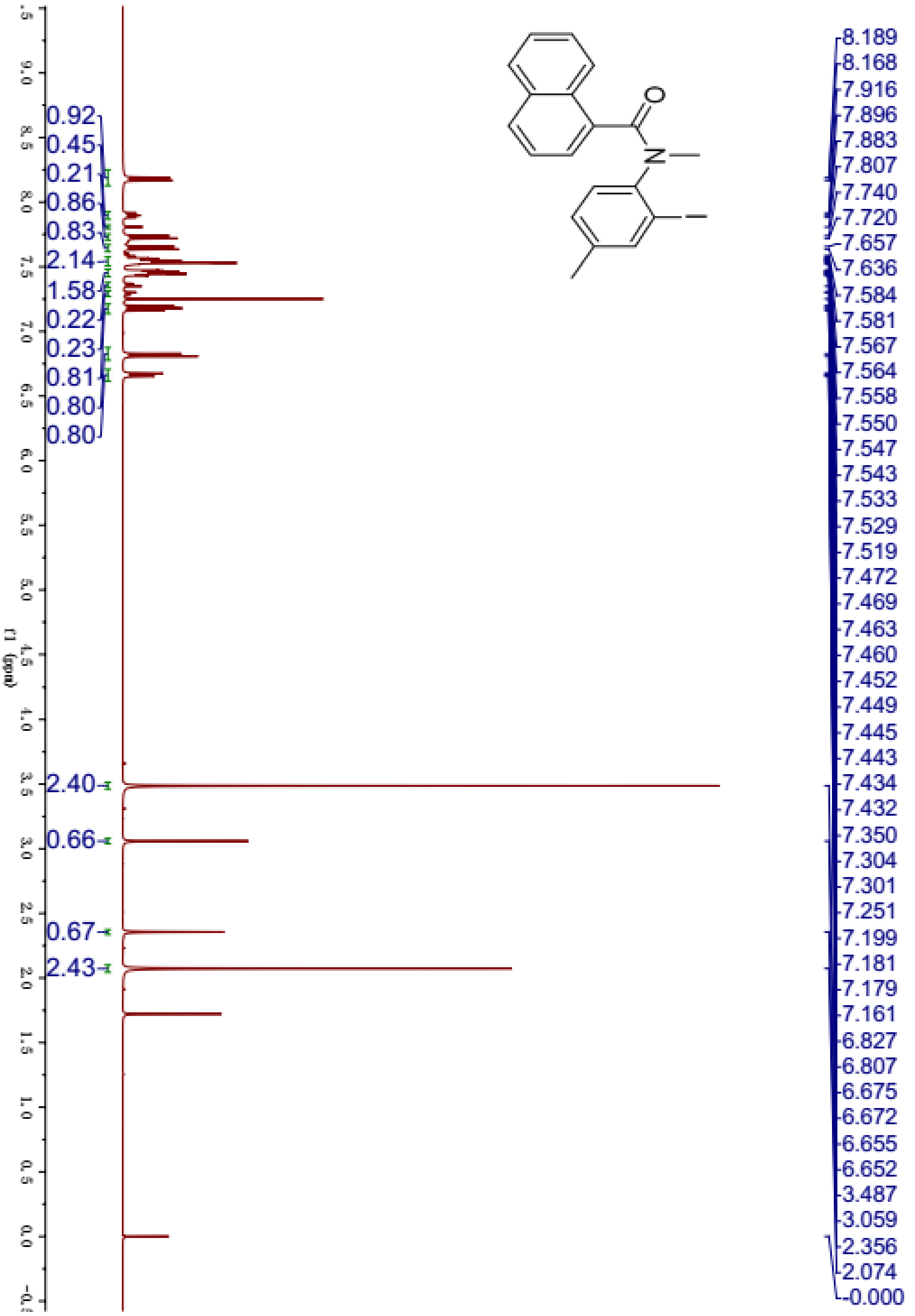
8.189
8.168
7.916
7.896
7.883
7.807
7.740
7.720
7.657
7.636
7.584
7.581
7.567
7.564
7.558
7.550
7.547
7.543
7.533
7.529
7.519
7.472
7.469
7.463
7.460
7.452
7.449
7.445
7.443
7.434
7.432
7.350
7.304
7.301
7.251
7.199
7.181
7.179
7.161
6.827
6.807
6.675
6.672
6.655
6.652
3.487
3.059
2.356
2.074
-0.000
0.92
0.45
0.21
0.86
0.83
2.14
1.58
0.22
0.23
0.81
0.80
0.80
2.40
0.66
0.67
2.43

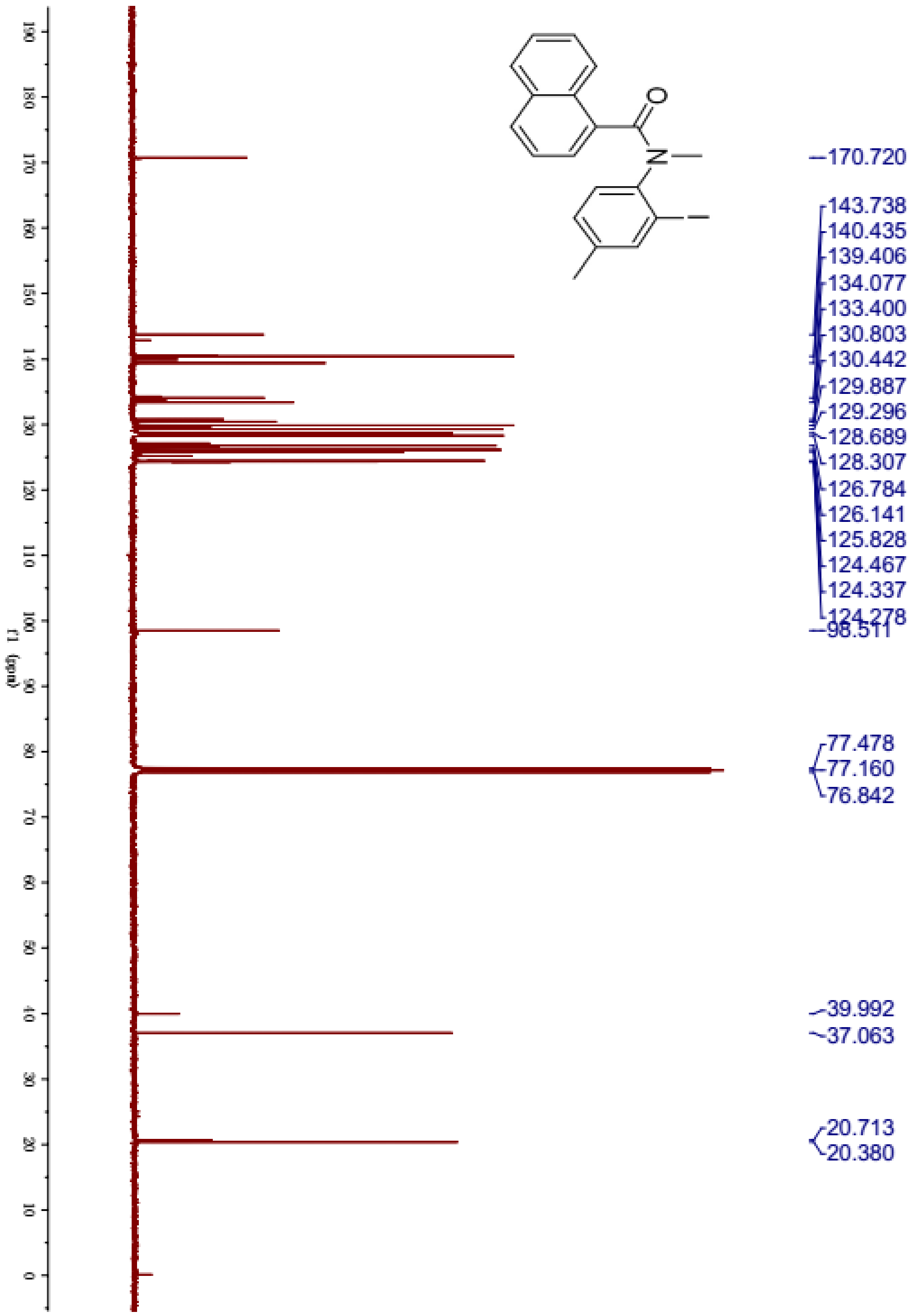
170.720
143.738
140.435
139.406
134.077
133.400
130.803
130.442
129.887
129.296
128.689
128.307
126.784
126.141
125.828
124.467
124.337
124.278
98.511
77.478
77.160
76.842
39.992
37.063
20.713
20.380

1l

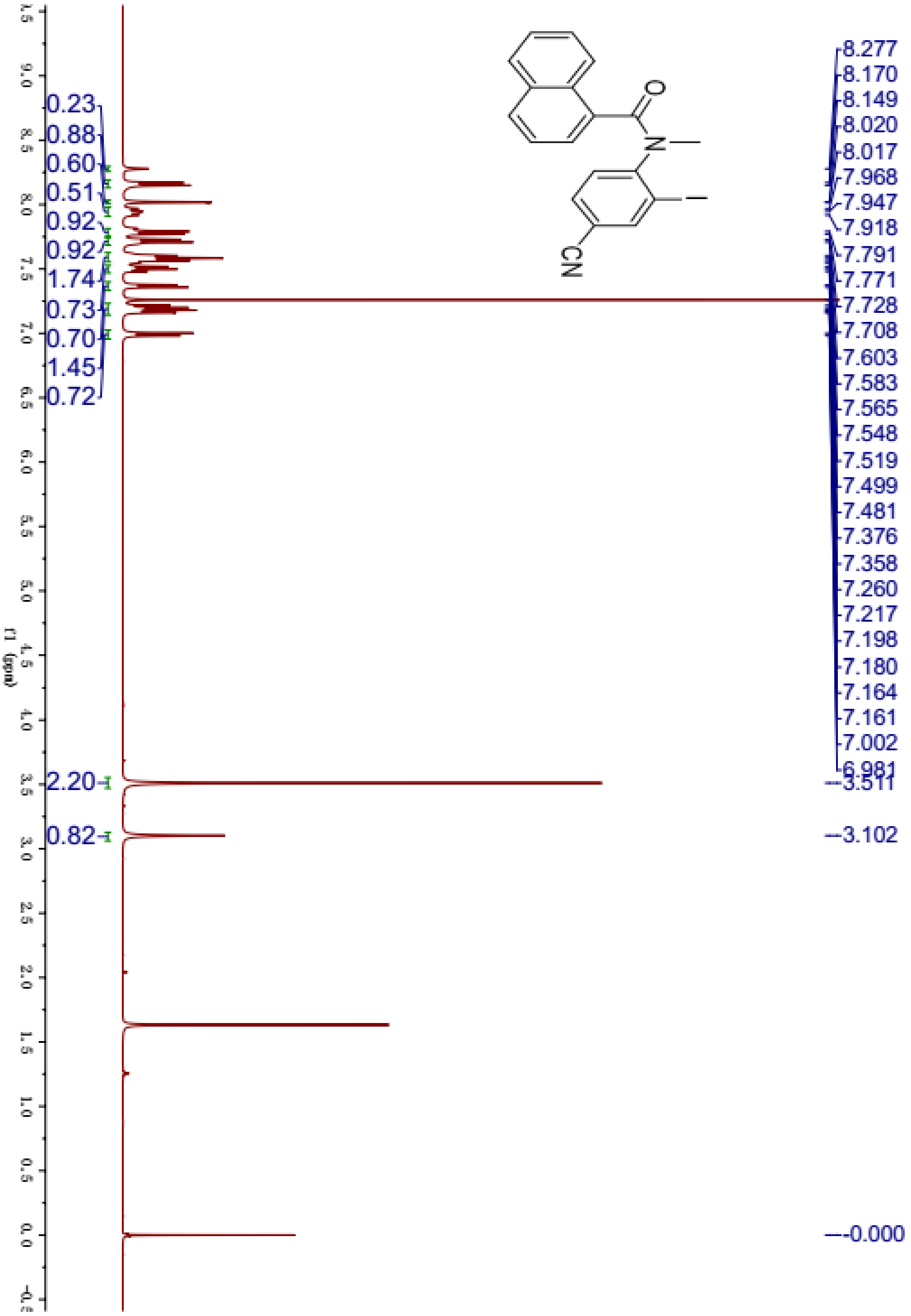
8.277
8.170
8.149
8.020
8.017
7.968
7.947
7.918
7.791
7.771
7.728
7.708
7.603
7.583
7.565
7.548
7.519
7.499
7.481
7.376
7.358
7.260
7.217
7.198
7.180
7.164
7.161
7.002
6.981
3.511
3.102
-0.000
0.23
0.88
0.60
0.51
0.92
0.92
1.74
0.73
0.70
1.45
0.72
2.20
0.82
f1 (ppm)
CN
O
N

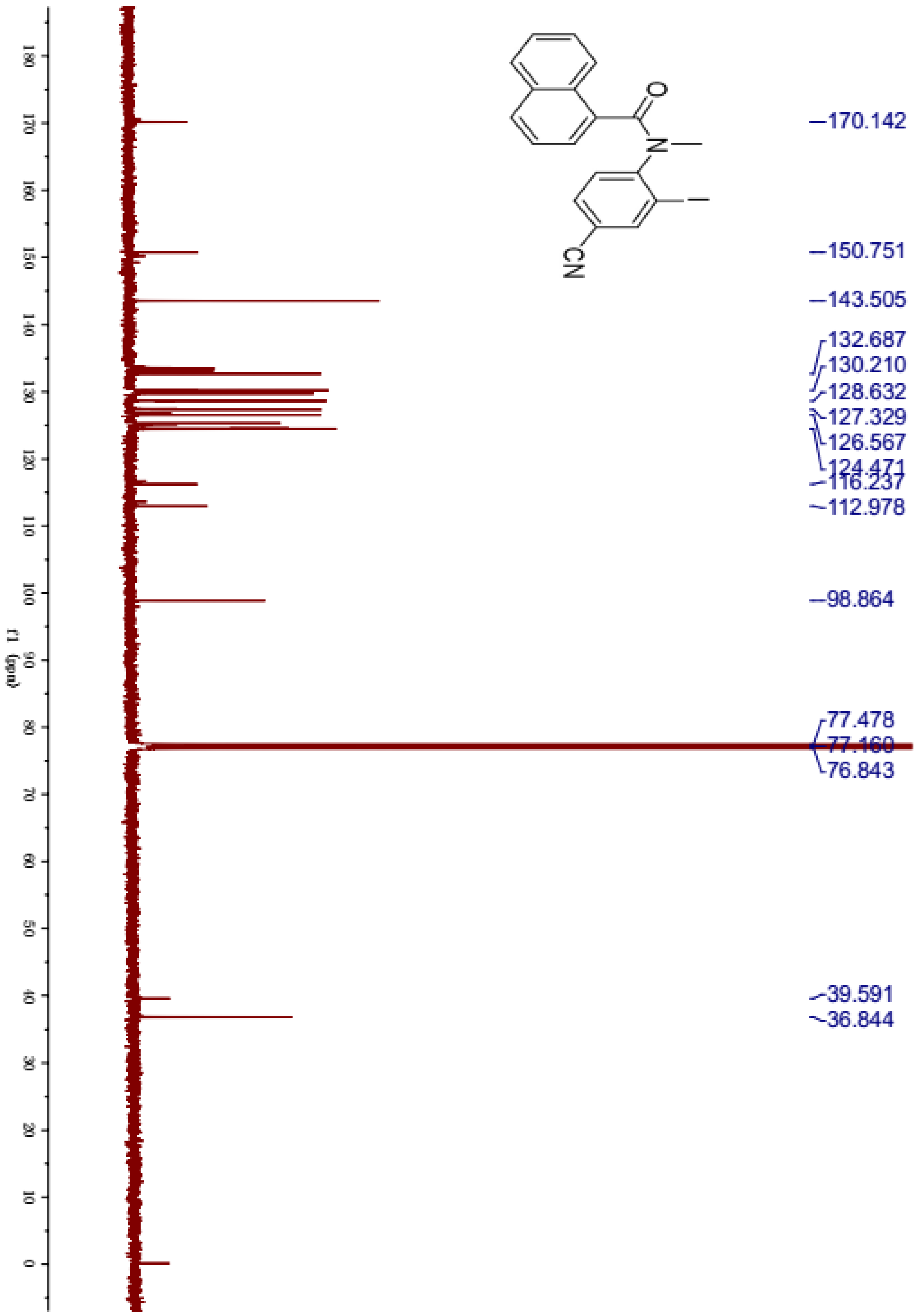
170.142
150.751
143.505
132.687
130.210
128.632
127.329
126.567
124.471
116.237
112.978
98.864
77.478
77.160
76.843
39.591
36.844
O
N
I
CN
f1 (ppm)

1m

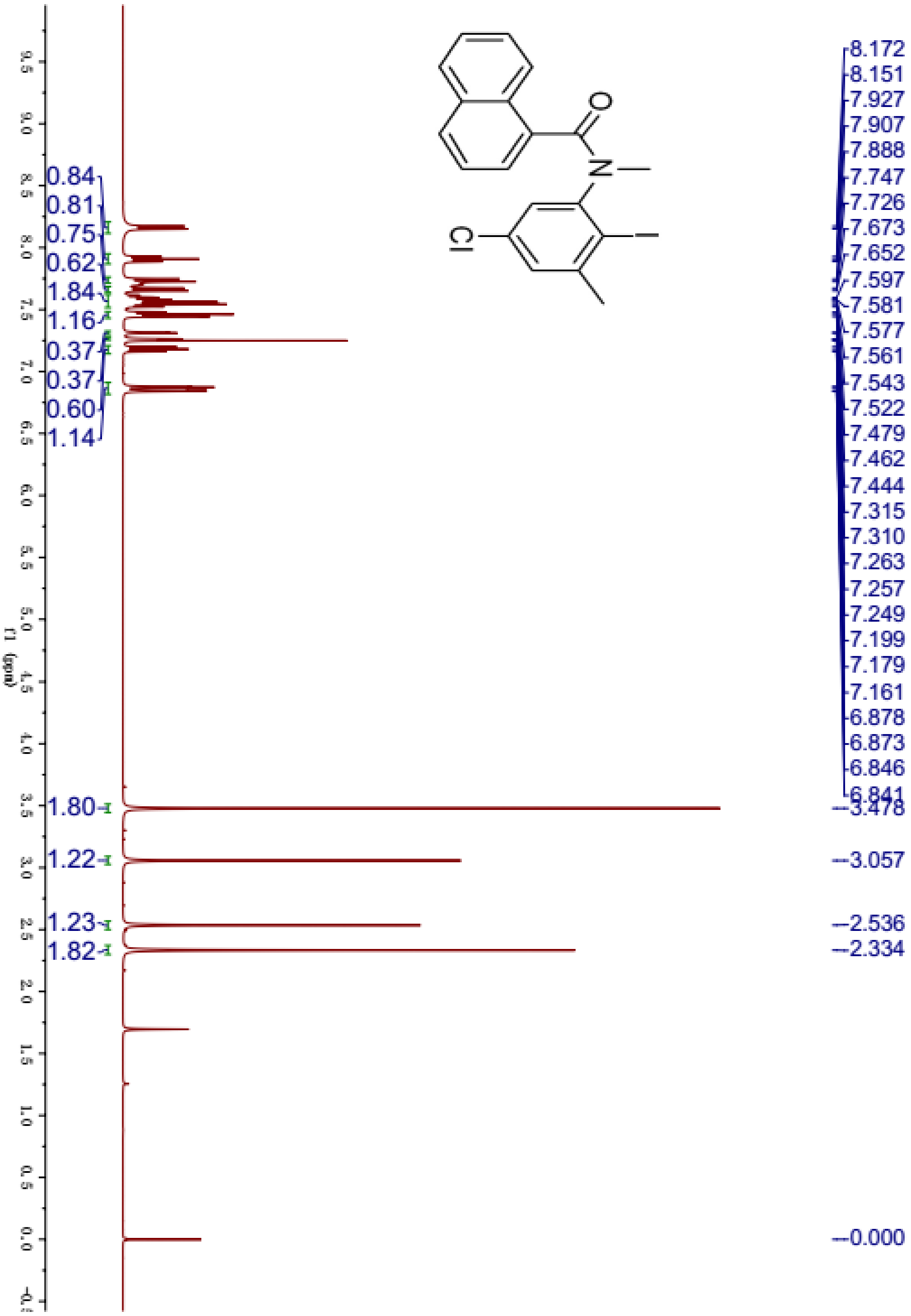
8.172
8.151
7.927
7.907
7.888
7.747
7.726
7.673
7.652
7.597
7.581
7.577
7.561
7.543
7.522
7.479
7.462
7.444
7.315
7.310
7.263
7.257
7.249
7.199
7.179
7.161
6.878
6.873
6.846
6.841
3.478
3.057
2.536
2.334
0.000
0.84
0.81
0.75
0.62
1.84
1.16
0.37
0.37
0.60
1.14
1.80
1.22
1.23
1.82
O
N
Cl

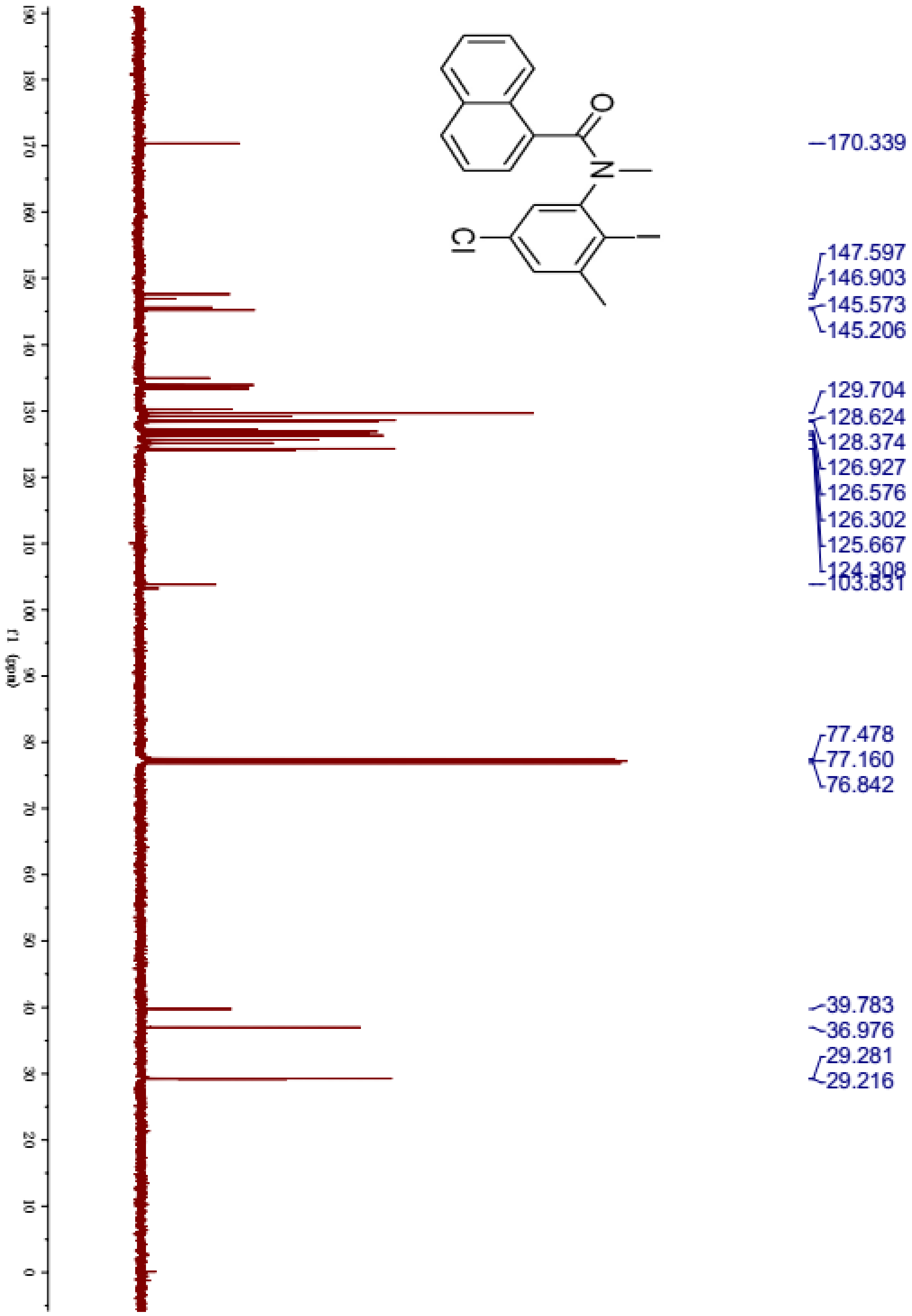
170.339
147.597
146.903
145.573
145.206
129.704
128.624
128.374
126.927
126.576
126.302
125.667
124.308
103.831
77.478
77.160
76.842
39.783
36.976
29.281
29.216
Cl
O
N
f1 (ppm)

1n

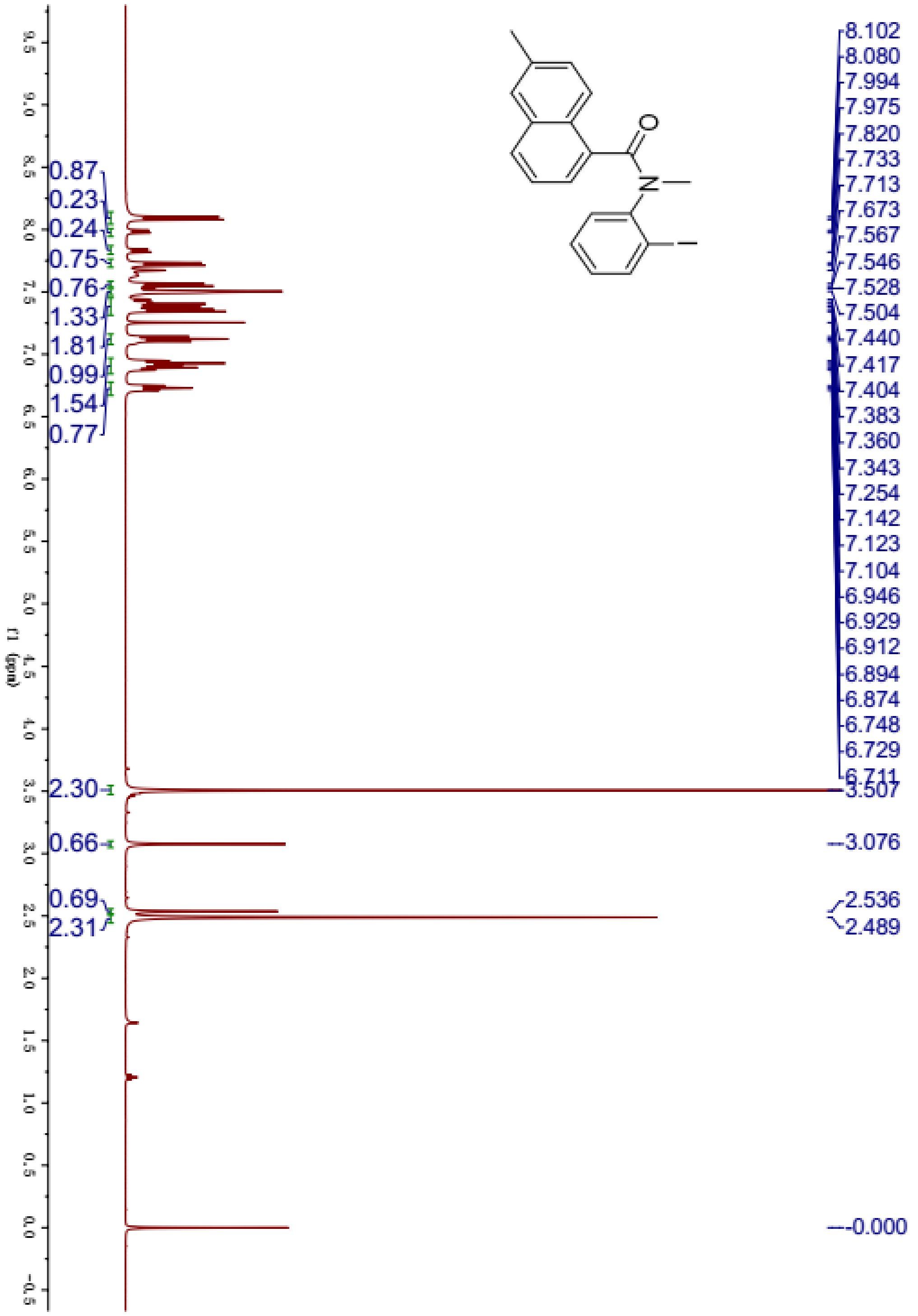
8.102
8.080
7.994
7.975
7.820
7.733
7.713
7.673
7.567
7.546
7.528
7.504
7.440
7.417
7.404
7.383
7.360
7.343
7.254
7.142
7.123
7.104
6.946
6.929
6.912
6.894
6.874
6.748
6.729
6.711
3.507
3.076
2.536
2.489
-0.000
0.87
0.23
0.24
0.75
0.76
1.33
1.81
0.99
1.54
0.77
2.30
0.66
0.69
2.31
f1 (ppm)

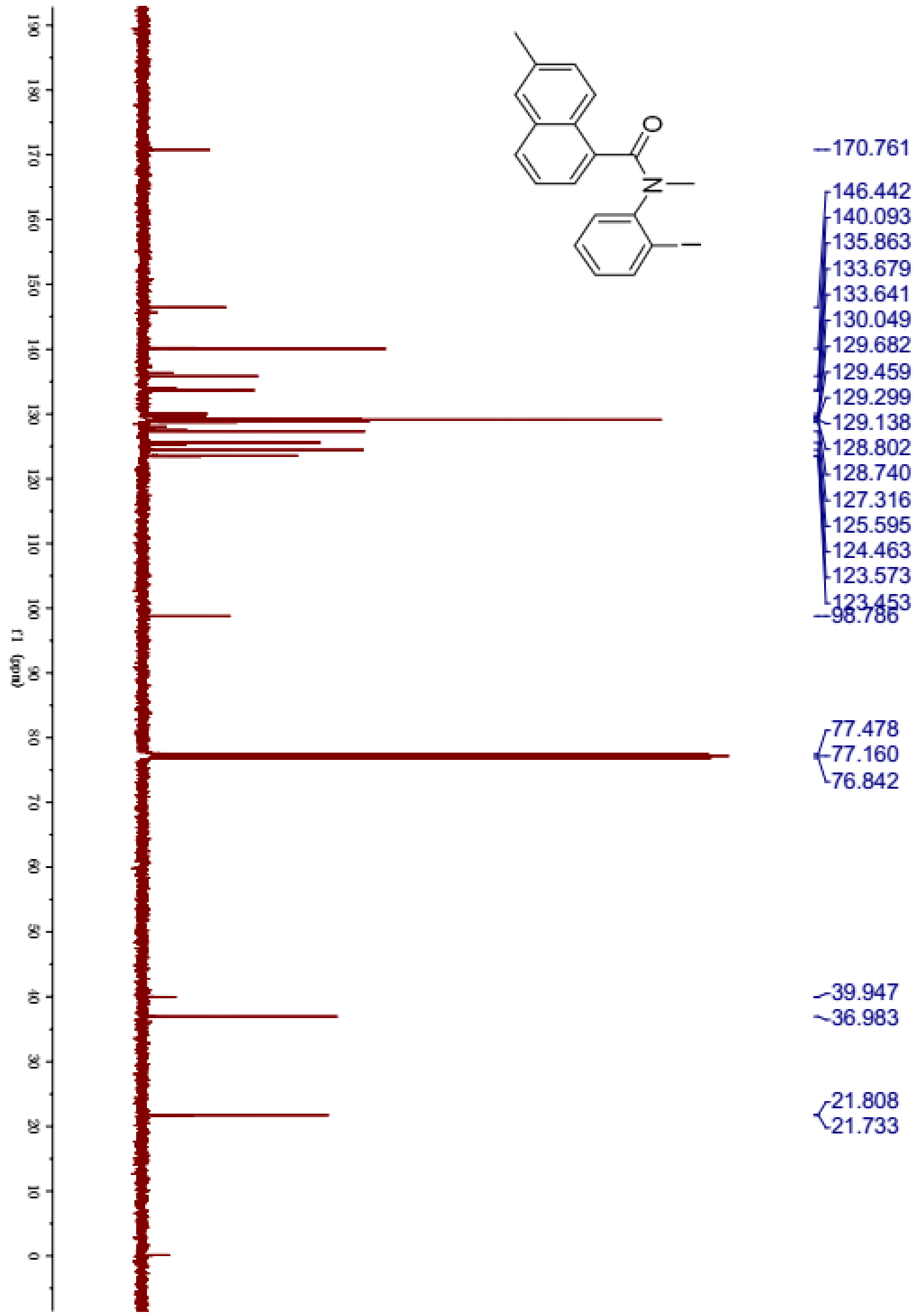
170.761
146.442
140.093
135.863
133.679
133.641
130.049
129.682
129.459
129.299
129.138
128.802
128.740
127.316
125.595
124.463
123.573
123.453
98.786
77.478
77.160
76.842
39.947
36.983
21.808
21.733
f1 (ppm)

1o

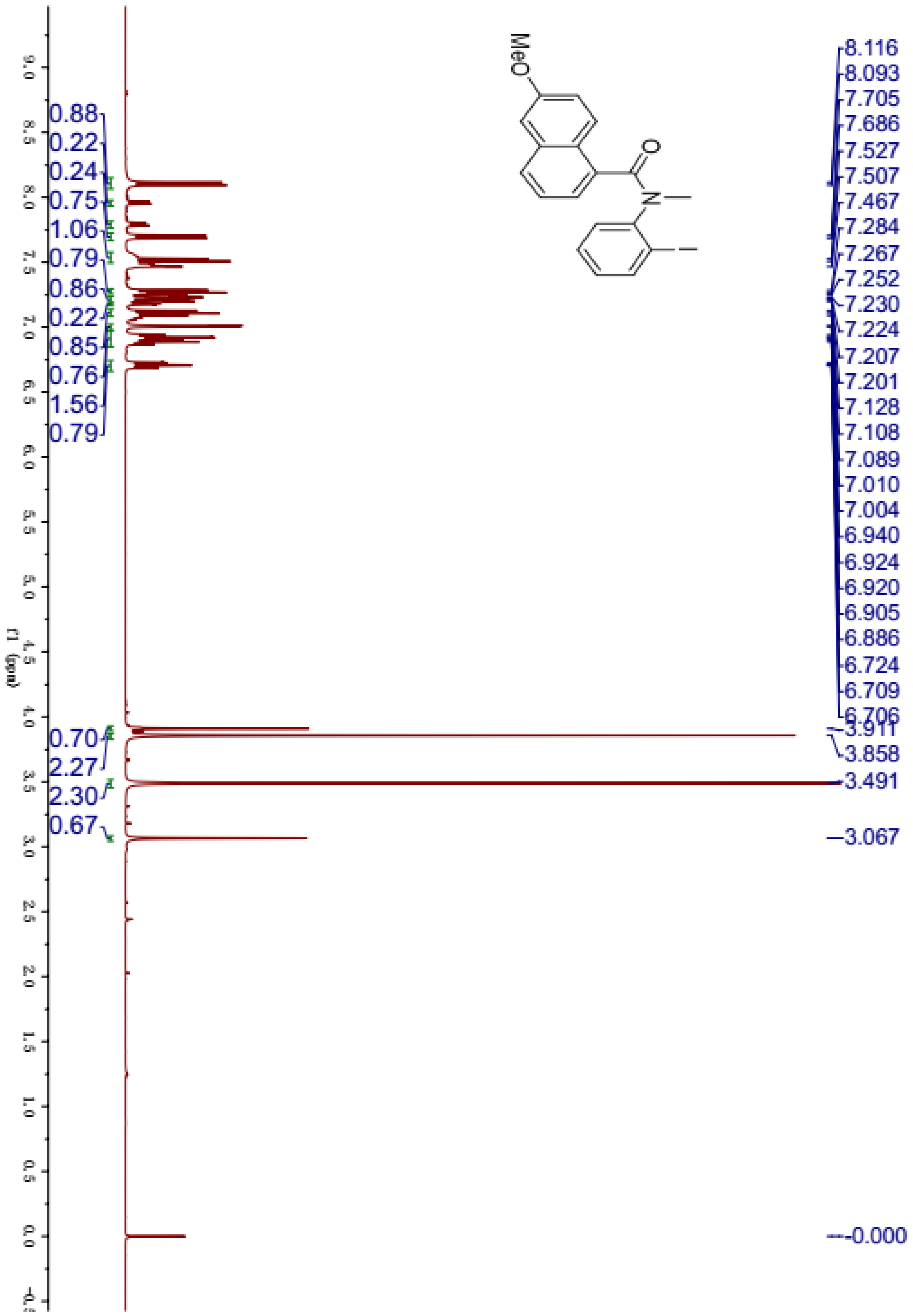
MeO
O
N
8.116
8.093
7.705
7.686
7.527
7.507
7.467
7.284
7.267
7.252
7.230
7.224
7.207
7.201
7.128
7.108
7.089
7.010
7.004
6.940
6.924
6.920
6.905
6.886
6.724
6.709
6.706
3.911
3.858
3.491
3.067
-0.000
0.88
0.22
0.24
0.75
1.06
0.79
0.86
0.22
0.85
0.76
1.56
0.79
0.70
2.27
2.30
0.67
f1 (ppm)

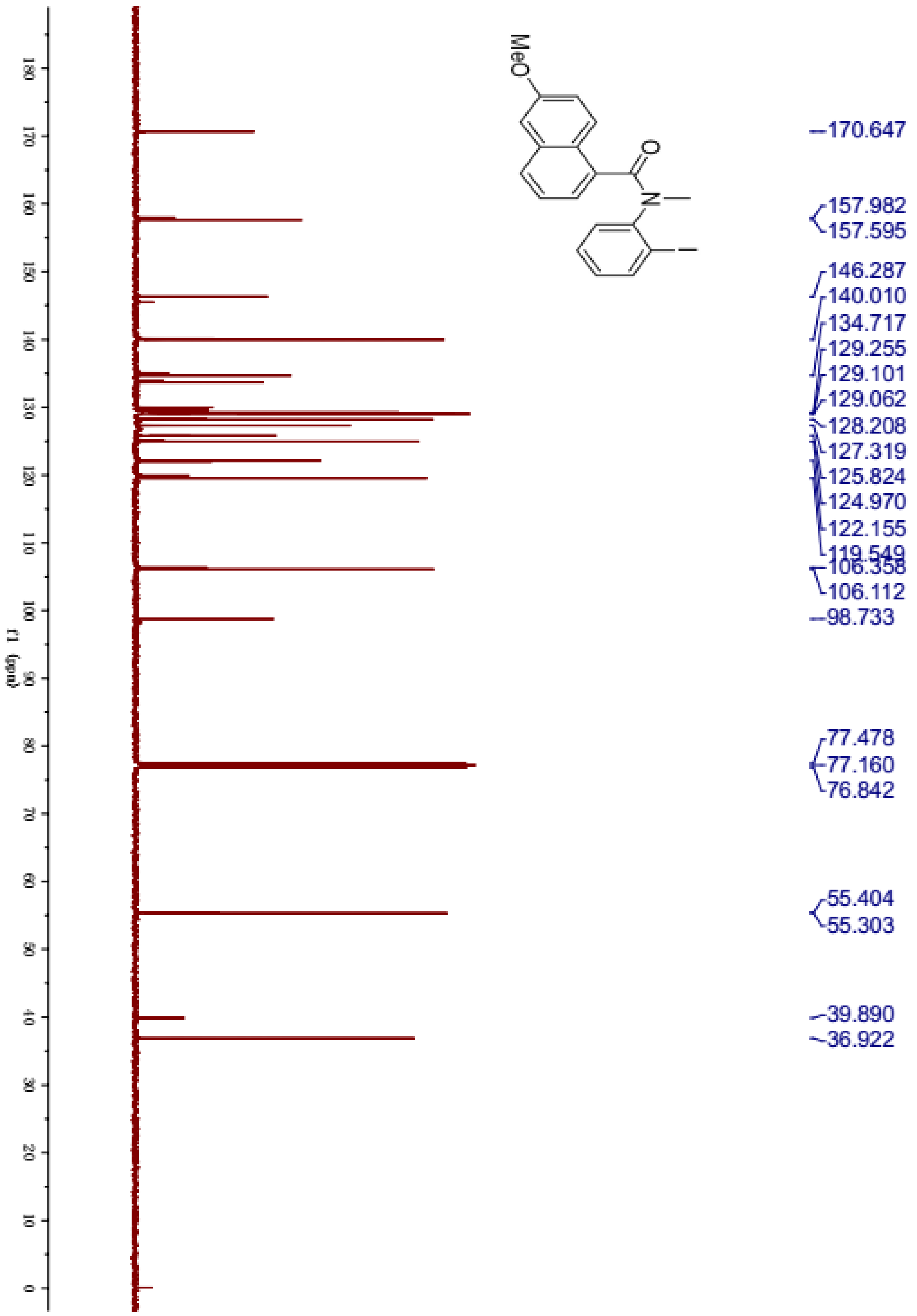

MeO
O
N
I
170.647
157.982
157.595
146.287
140.010
134.717
129.255
129.101
129.062
128.208
127.319
125.824
124.970
122.155
119.549
106.358
106.112
98.733
77.478
77.160
76.842
55.404
55.303
39.890
36.922
f1 (ppm)

1p

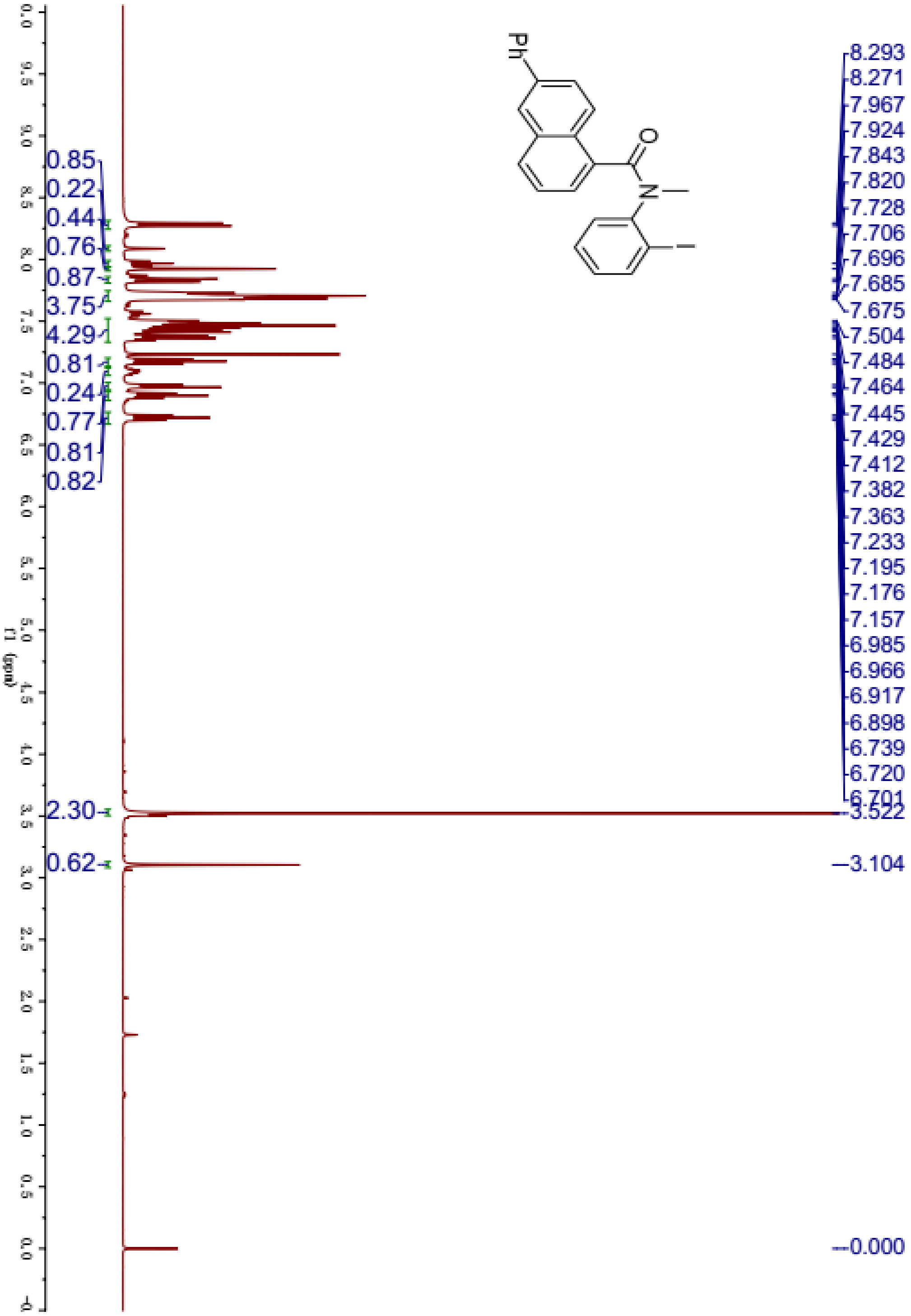

Ph
O
N
8.293
8.271
7.967
7.924
7.843
7.820
7.728
7.706
7.696
7.685
7.675
7.504
7.484
7.464
7.445
7.429
7.412
7.382
7.363
7.233
7.195
7.176
7.157
6.985
6.966
6.917
6.898
6.739
6.720
6.701
3.522
3.104
0.000
0.85
0.22
0.44
0.76
0.87
3.75
4.29
0.81
0.24
0.77
0.81
0.82
2.30
0.62
f1 (ppm)

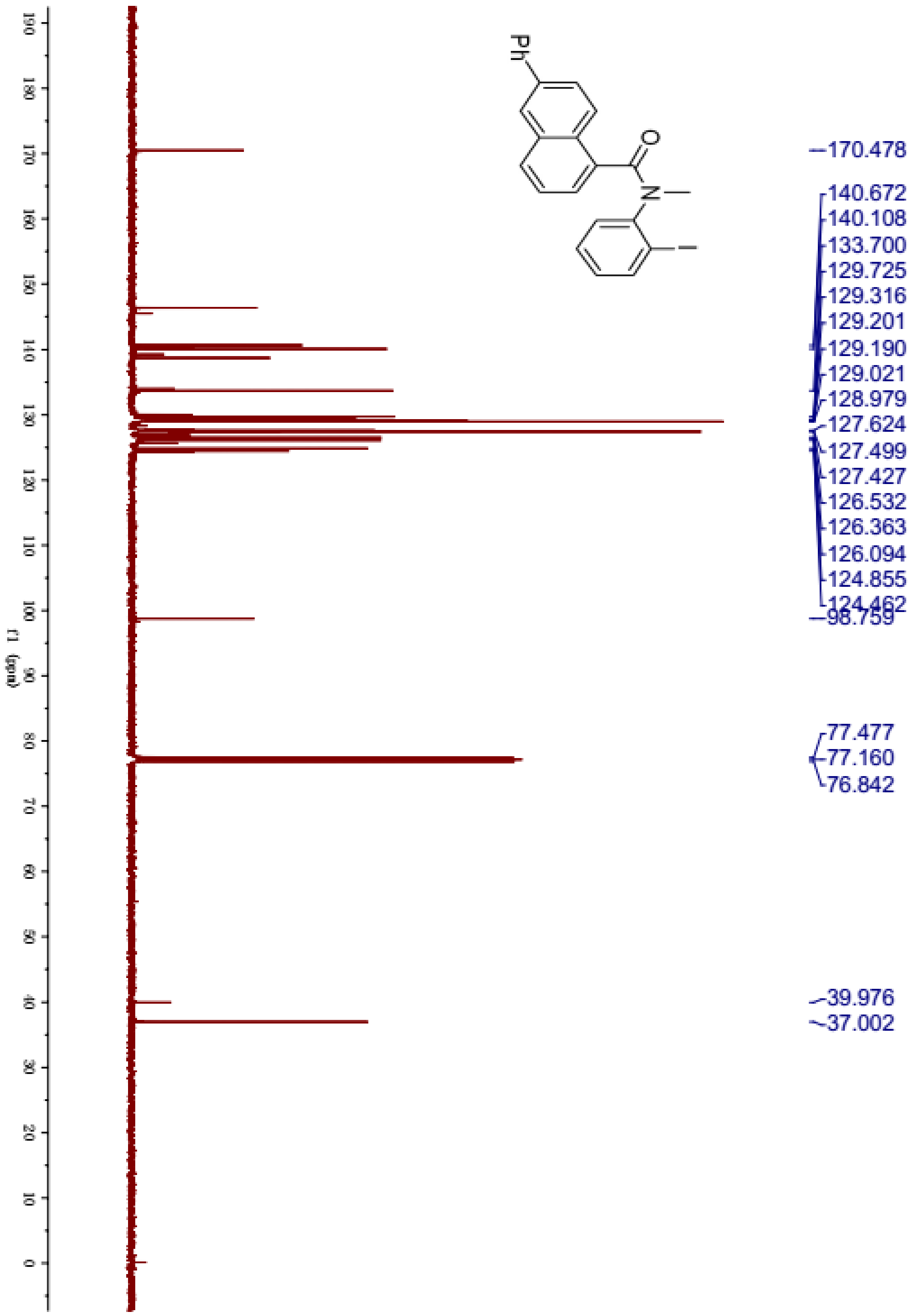
Ph
O
N
170.478
140.672
140.108
133.700
129.725
129.316
129.201
129.190
129.021
128.979
127.624
127.499
127.427
126.532
126.363
126.094
124.855
124.462
98.759
77.477
77.160
76.842
39.976
37.002
f1 (ppm)

3a

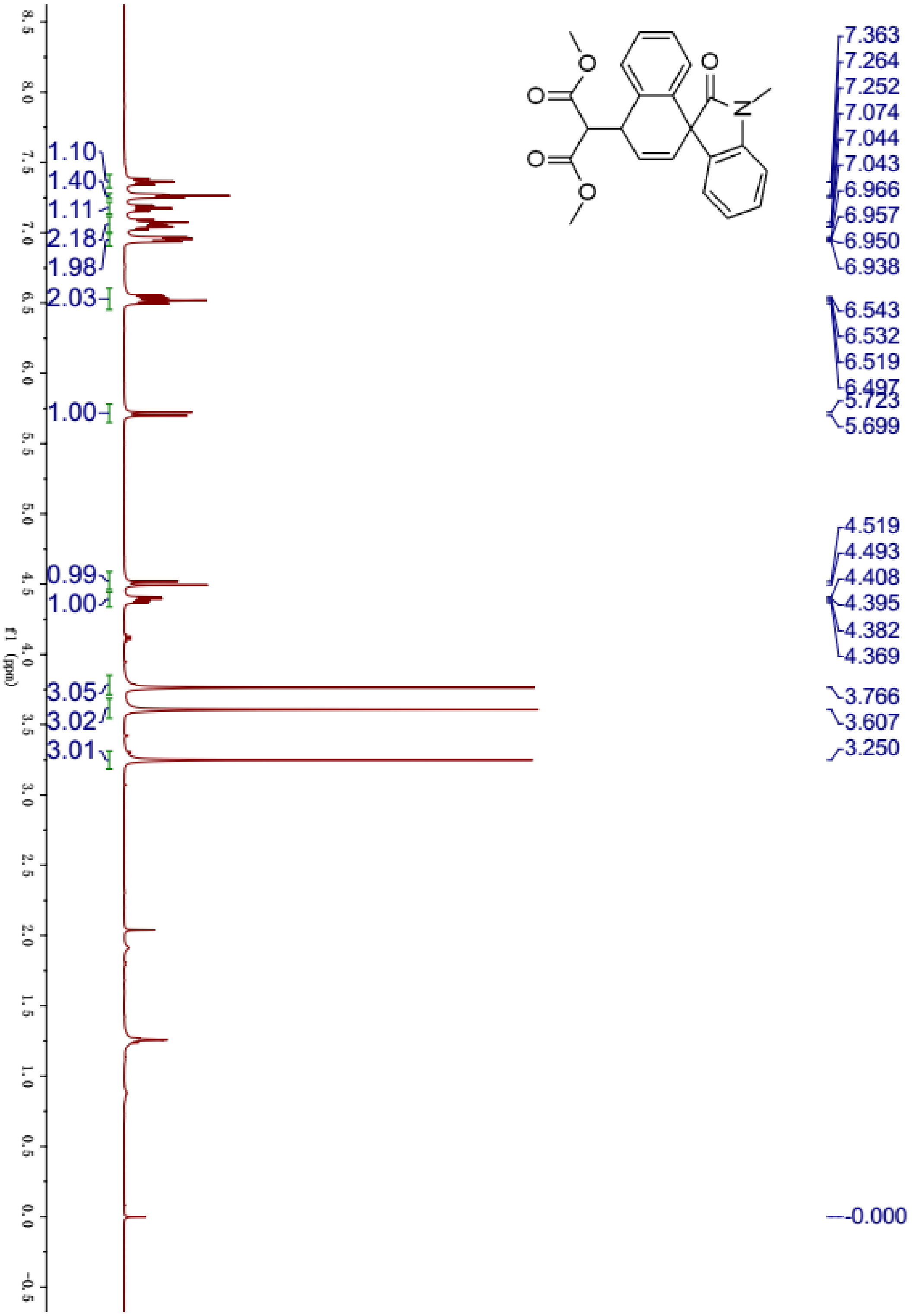

7.363
7.264
7.252
7.074
7.044
7.043
6.966
6.957
6.950
6.938
6.543
6.532
6.519
6.497
5.723
5.699
4.519
4.493
4.408
4.395
4.382
4.369
3.766
3.607
3.250
-0.000
1.10
1.40
1.11
2.18
1.98
2.03
1.00
0.99
1.00
3.05
3.02
3.01
f1 (ppm)
O
N

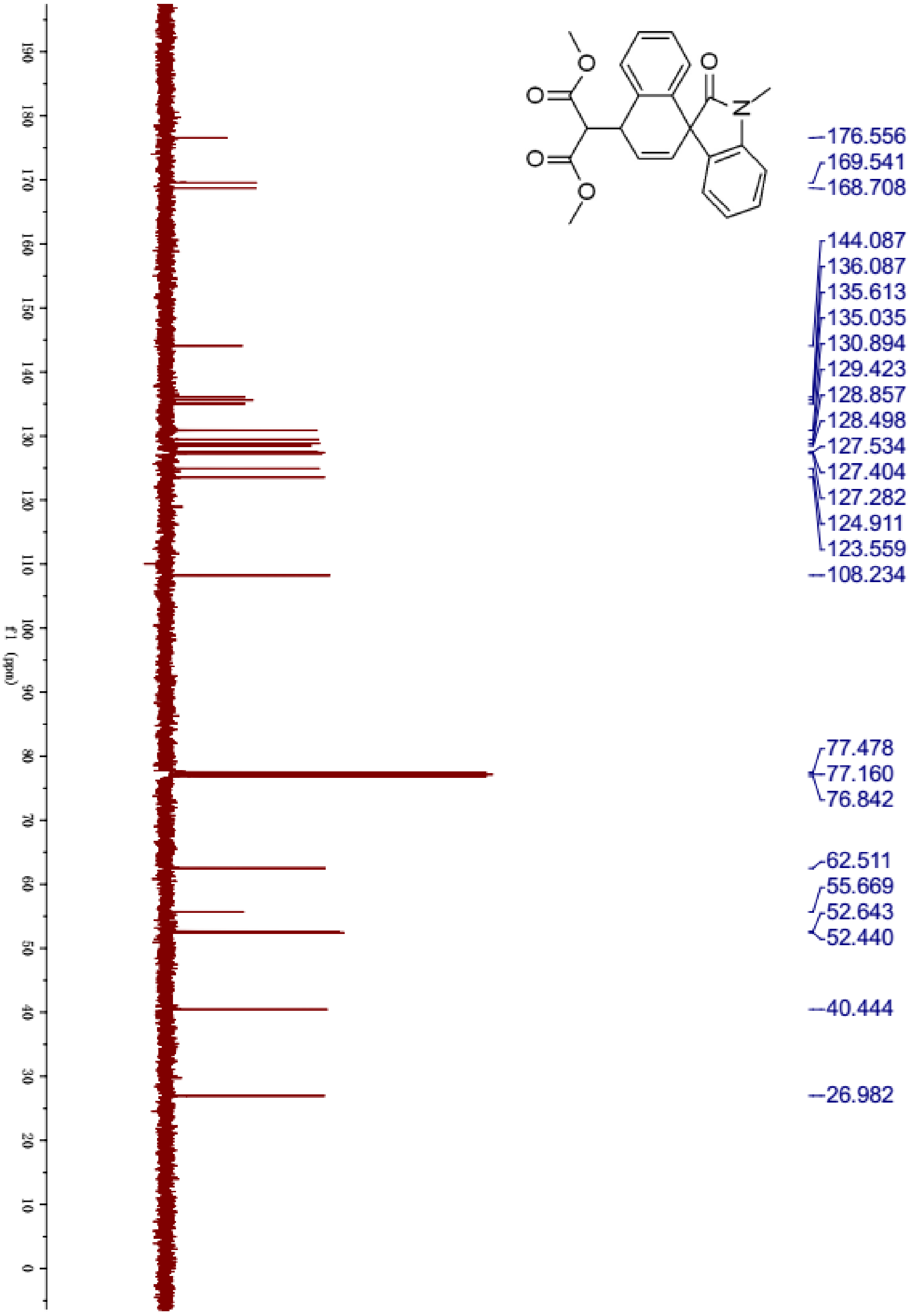

f1 (ppm)
176.556
169.541
168.708
144.087
136.087
135.613
135.035
130.894
129.423
128.857
128.498
127.534
127.404
127.282
124.911
123.559
108.234
77.478
77.160
76.842
62.511
55.669
52.643
52.440
40.444
26.982

3b

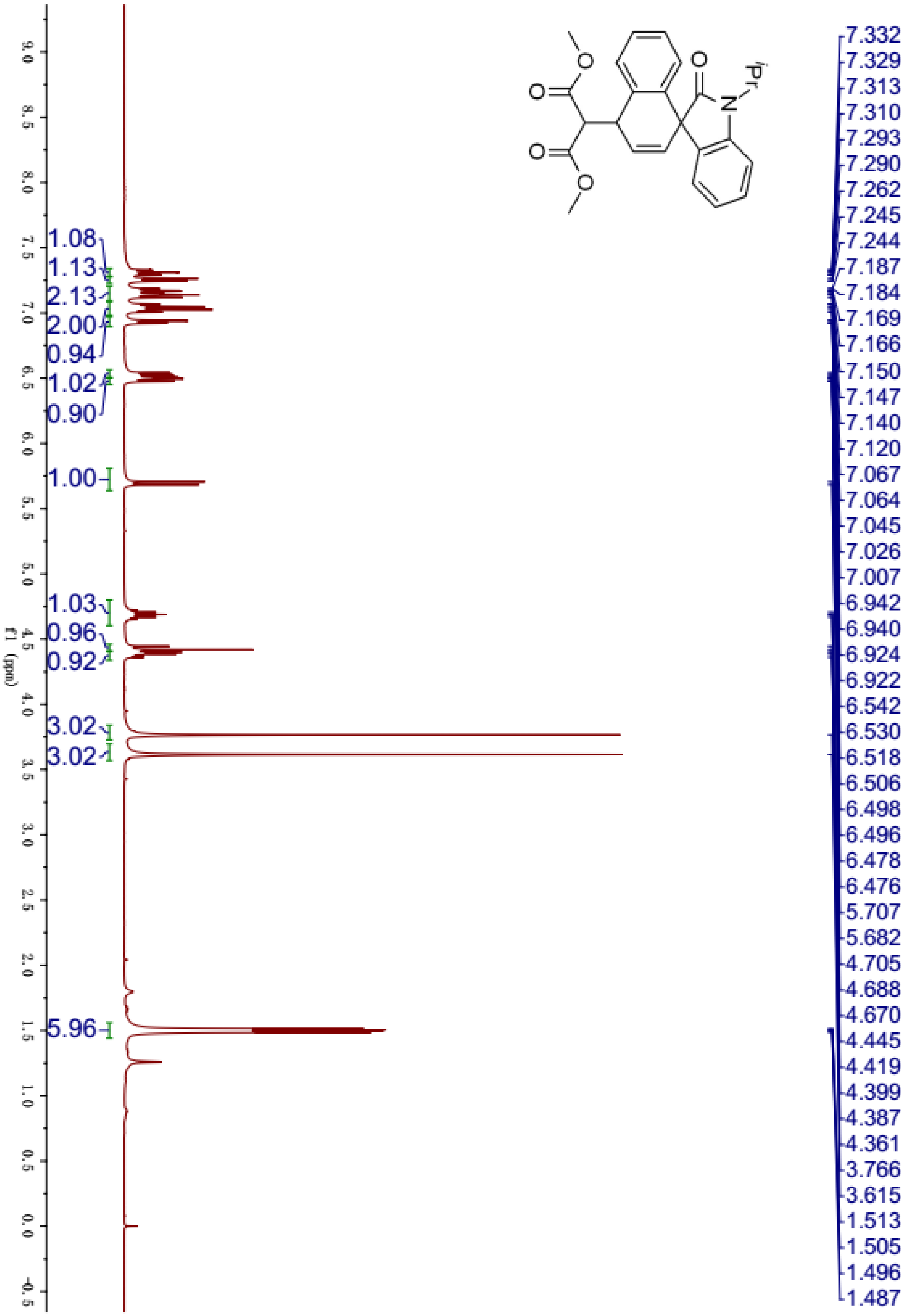
7.332
7.329
7.313
7.310
7.293
7.290
7.262
7.245
7.244
7.187
7.184
7.169
7.166
7.150
7.147
7.140
7.120
7.067
7.064
7.045
7.026
7.007
6.942
6.940
6.924
6.922
6.542
6.530
6.518
6.506
6.498
6.496
6.478
6.476
5.707
5.682
4.705
4.688
4.670
4.445
4.419
4.399
4.387
4.361
3.766
3.615
1.513
1.505
1.496
1.487
1.08
1.13
2.13
2.00
0.94
1.02
0.90
1.00
1.03
0.96
0.92
3.02
3.02
5.96
f1 (ppm)
O
N
iPr

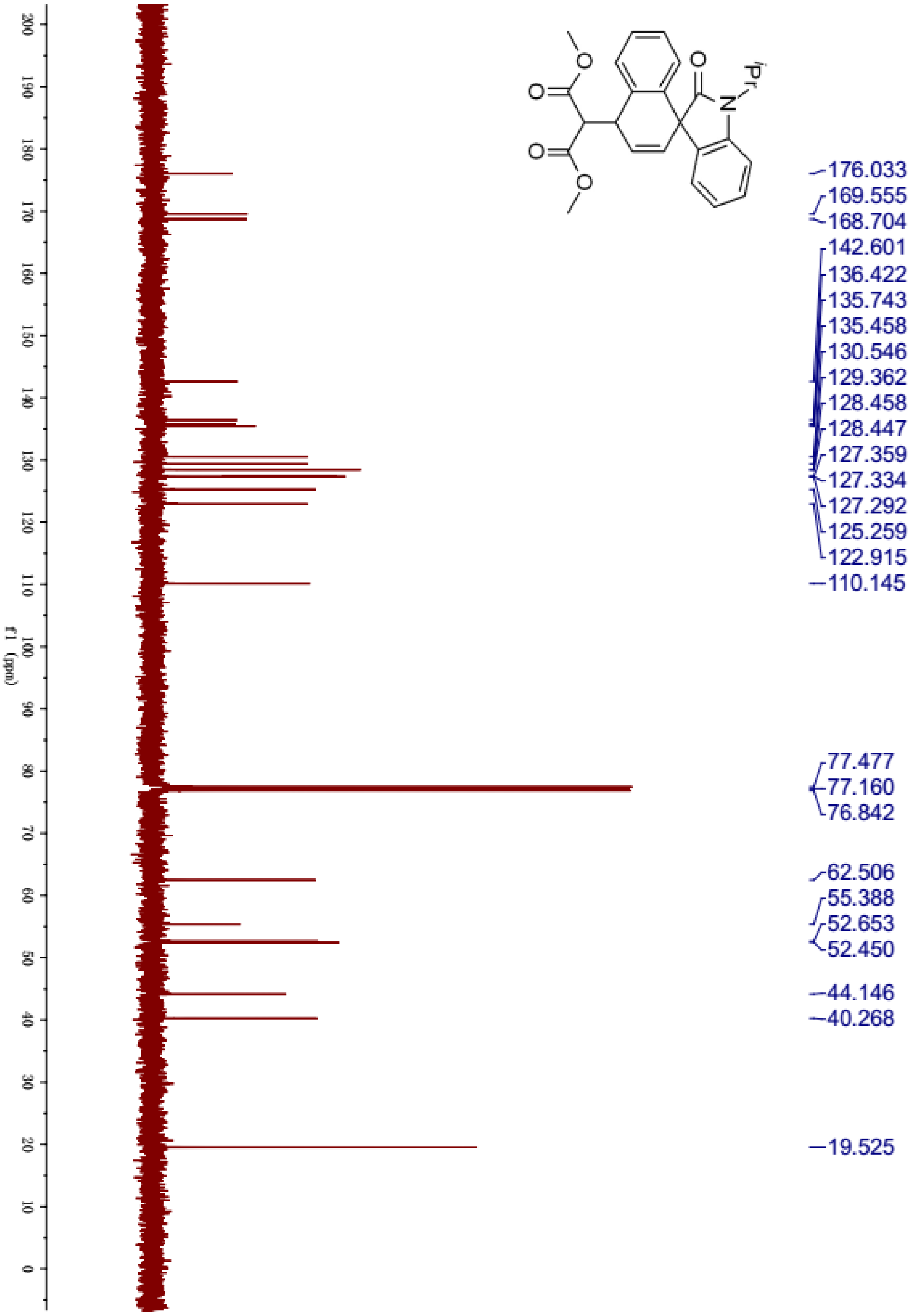

-176.033
169.555
168.704
142.601
136.422
135.743
135.458
130.546
129.362
128.458
128.447
127.359
127.334
127.292
125.259
122.915
-110.145
77.477
77.160
76.842
62.506
55.388
52.653
52.450
-44.146
-40.268
-19.525
f1 (ppm)

3c

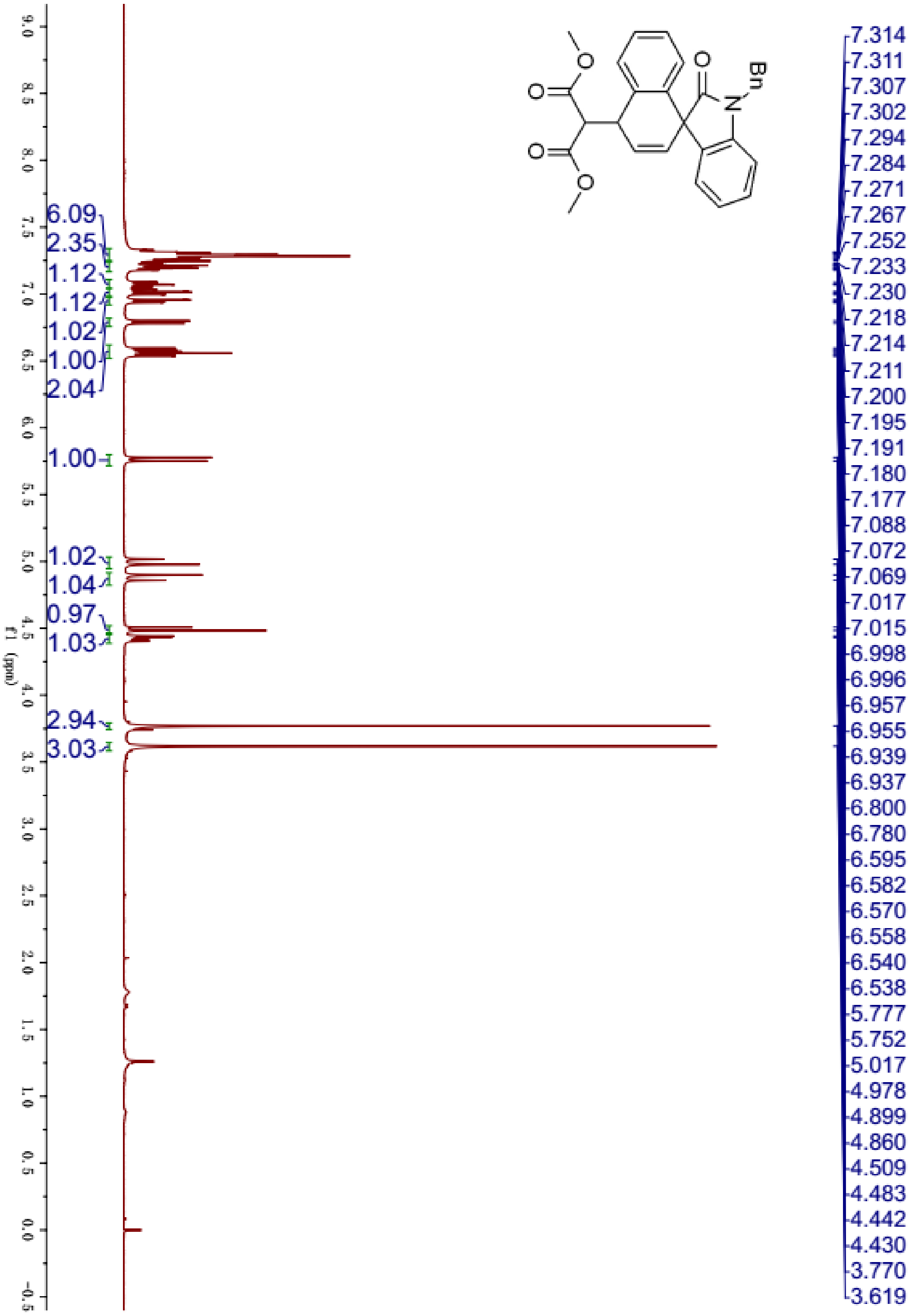
7.314
7.311
7.307
7.302
7.294
7.284
7.271
7.267
7.252
7.233
7.230
7.218
7.214
7.211
7.200
7.195
7.191
7.180
7.177
7.088
7.072
7.069
7.017
7.015
6.998
6.996
6.957
6.955
6.939
6.937
6.800
6.780
6.595
6.582
6.570
6.558
6.540
6.538
5.777
5.752
5.017
4.978
4.899
4.860
4.509
4.483
4.442
4.430
3.770
3.619
6.09
2.35
1.12
1.12
1.02
1.00
2.04
1.00
1.02
1.04
0.97
1.03
2.94
3.03
Bn
N
O
O
O
O
O

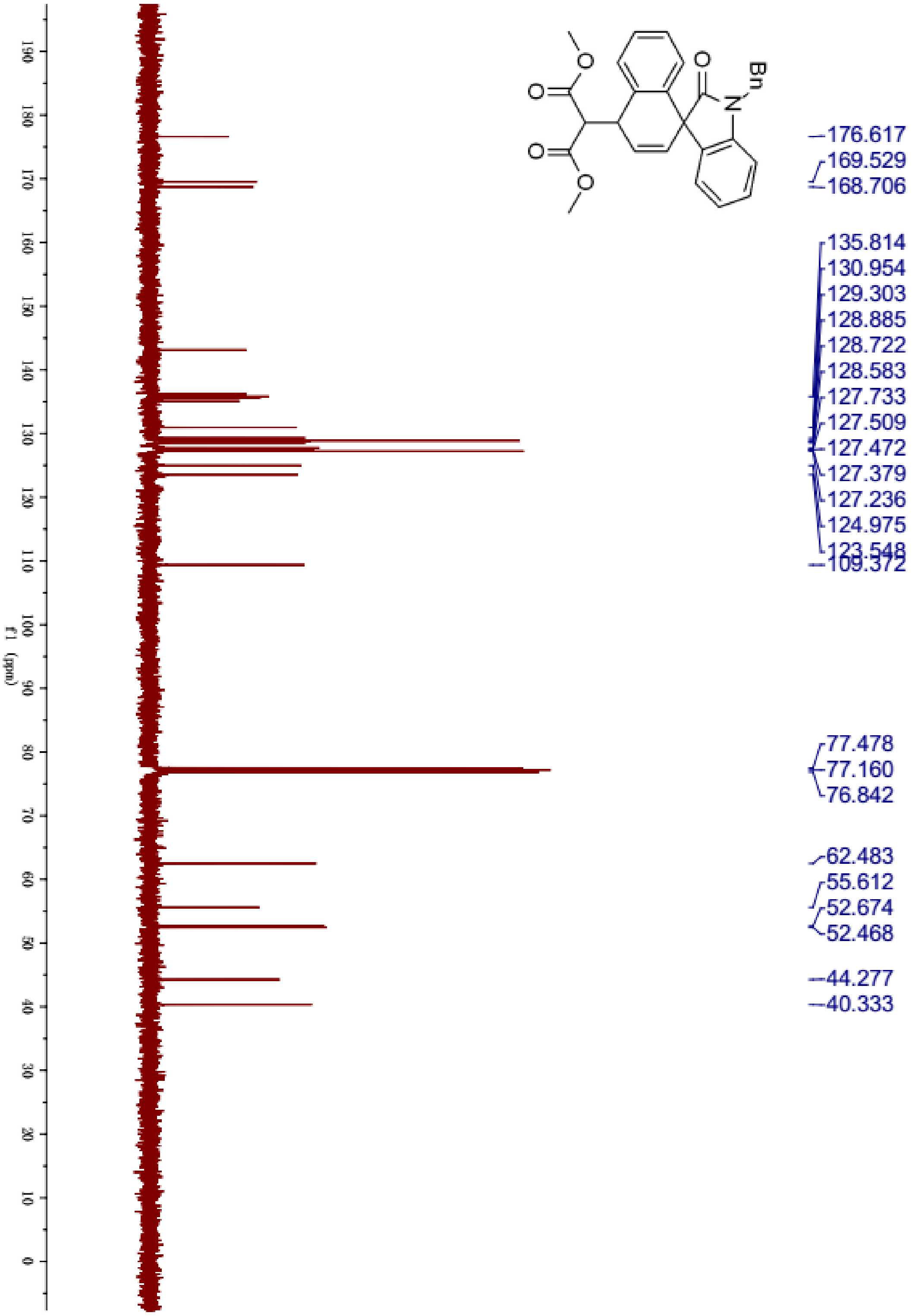
176.617
169.529
168.706
135.814
130.954
129.303
128.885
128.722
128.583
127.733
127.509
127.472
127.379
127.236
124.975
123.548
109.372
77.478
77.160
76.842
62.483
55.612
52.674
52.468
44.277
40.333
f1 (ppm)
Bn

3d

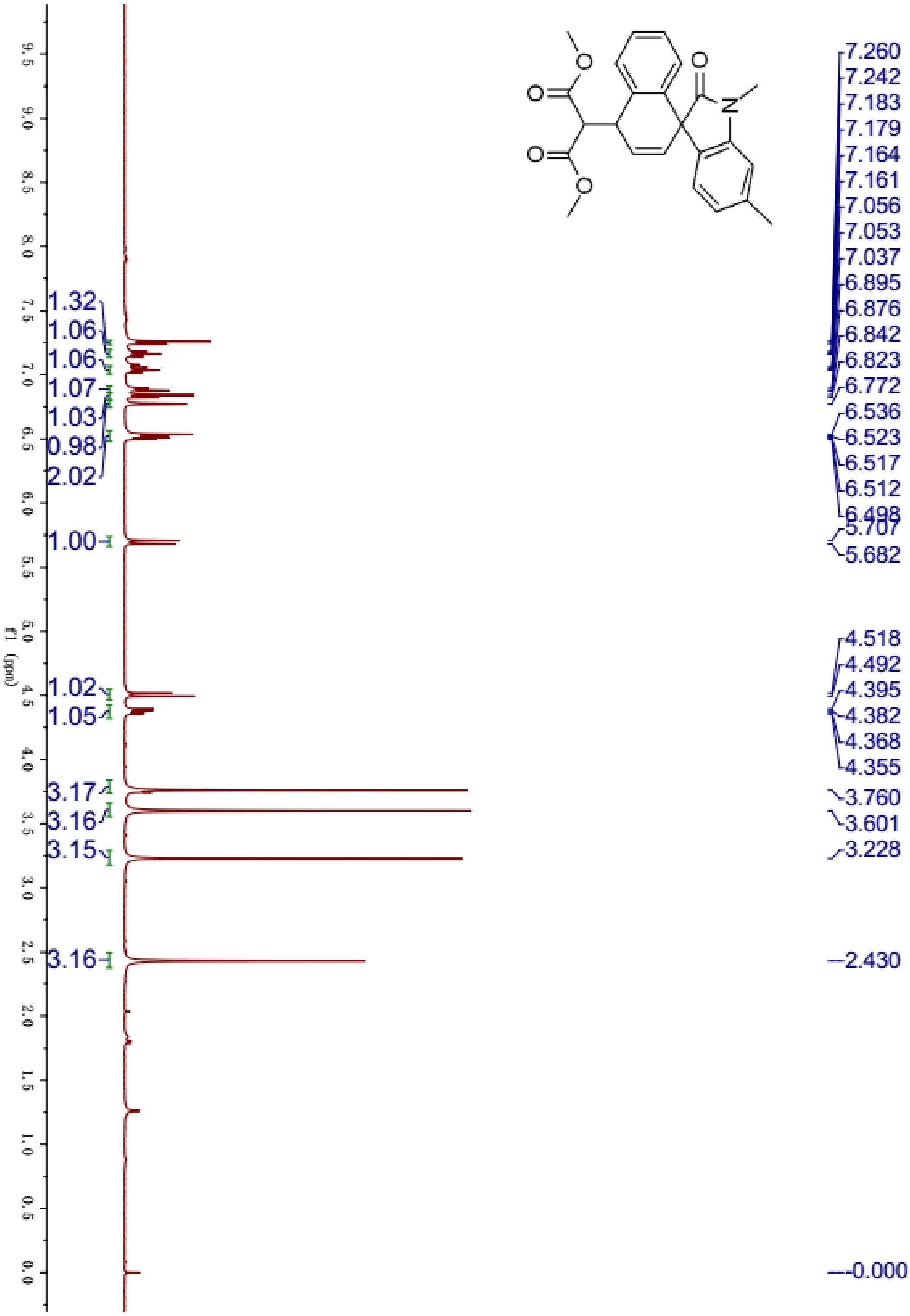
7.260
7.242
7.183
7.179
7.164
7.161
7.056
7.053
7.037
6.895
6.876
6.842
6.823
6.772
6.536
6.523
6.517
6.512
6.498
5.707
5.682
4.518
4.492
4.395
4.382
4.368
4.355
3.760
3.601
3.228
2.430
-0.000
1.32
1.06
1.06
1.07
1.03
0.98
2.02
1.00
1.02
1.05
3.17
3.16
3.15
3.16
f1 (ppm)

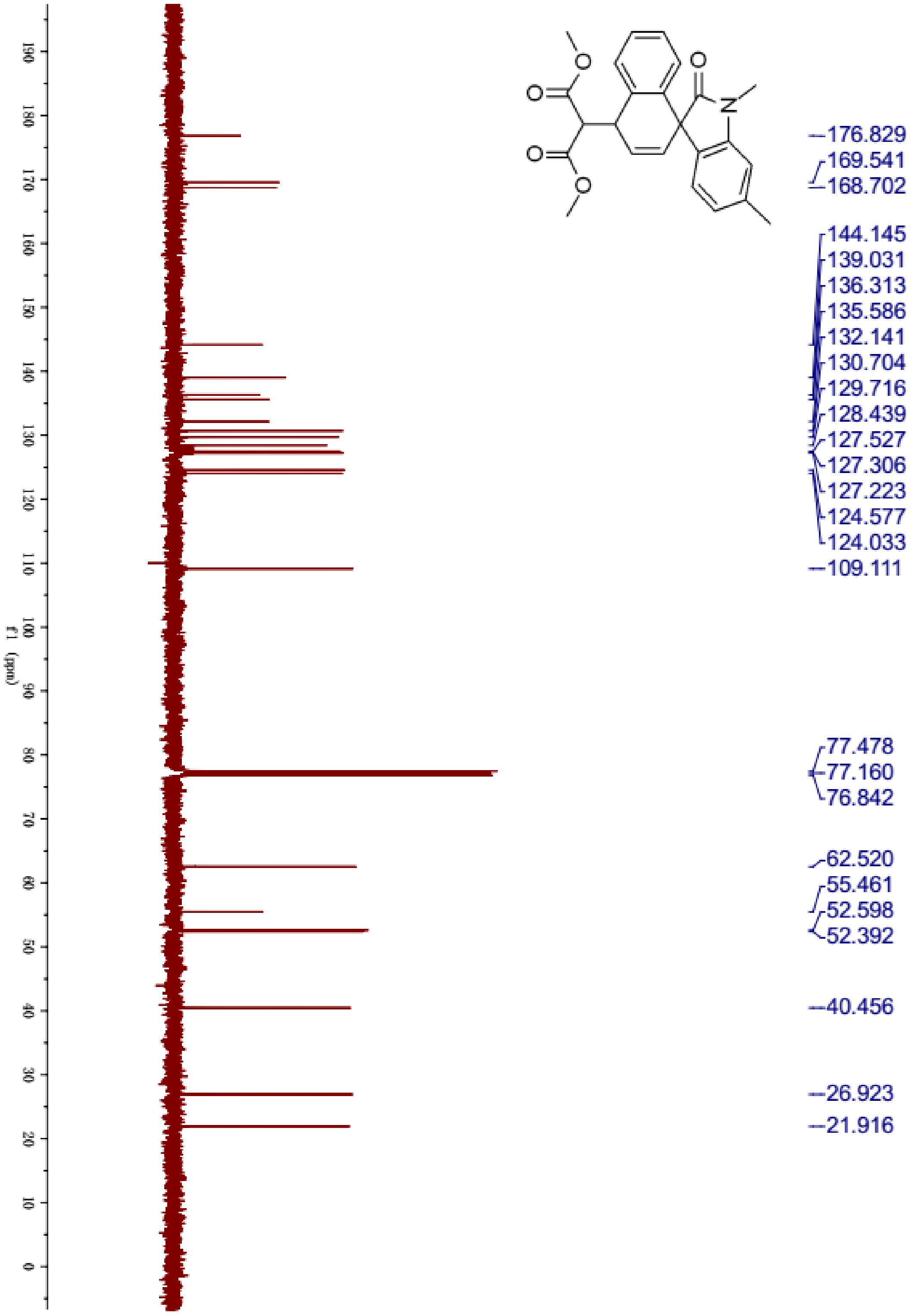
176.829
169.541
168.702
144.145
139.031
136.313
135.586
132.141
130.704
129.716
128.439
127.527
127.306
127.223
124.577
124.033
109.111
77.478
77.160
76.842
62.520
55.461
52.598
52.392
40.456
26.923
21.916
f1 (ppm)

3e

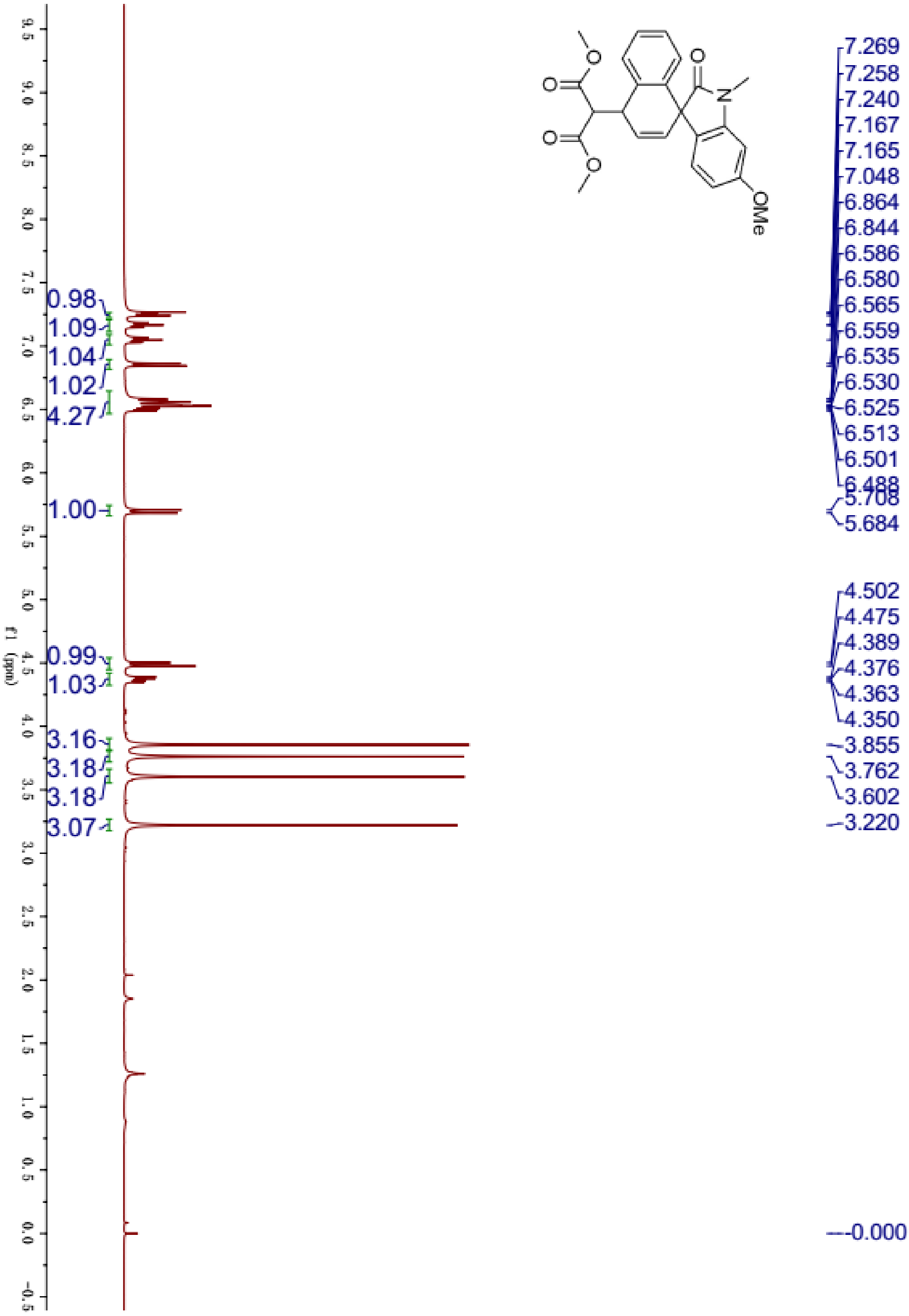
7.269
7.258
7.240
7.167
7.165
7.048
6.864
6.844
6.586
6.580
6.565
6.559
6.535
6.530
6.525
6.513
6.501
6.488
5.708
5.684
4.502
4.475
4.389
4.376
4.363
4.350
3.855
3.762
3.602
3.220
-0.000
0.98
1.09
1.04
1.02
4.27
1.00
0.99
1.03
3.16
3.18
3.18
3.07
OMe
f1 (ppm)

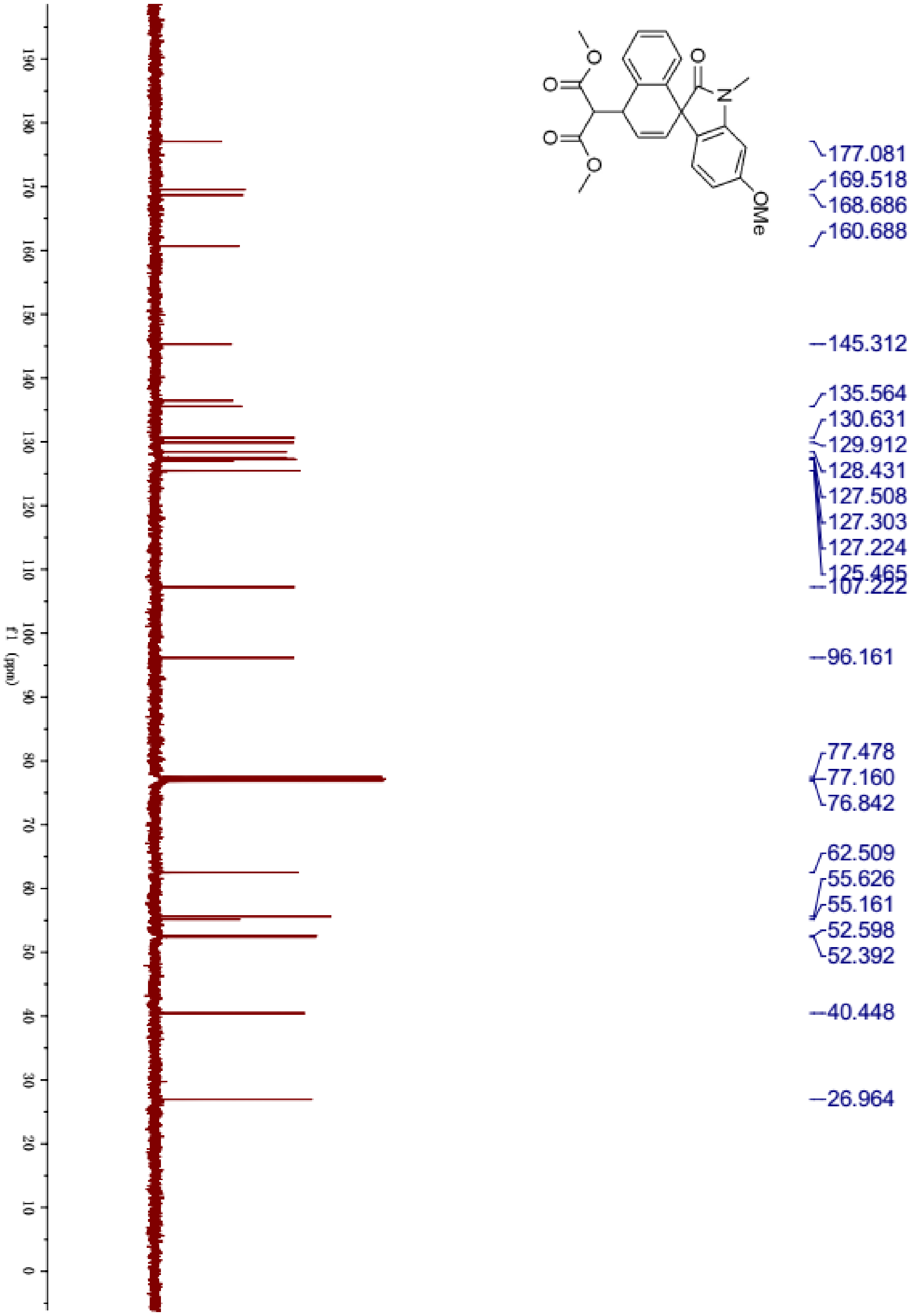

177.081
169.518
168.686
160.688
145.312
135.564
130.631
129.912
128.431
127.508
127.303
127.224
125.465
107.222
96.161
77.478
77.160
76.842
62.509
55.626
55.161
52.598
52.392
40.448
26.964
OMe
f1 (ppm)

3f

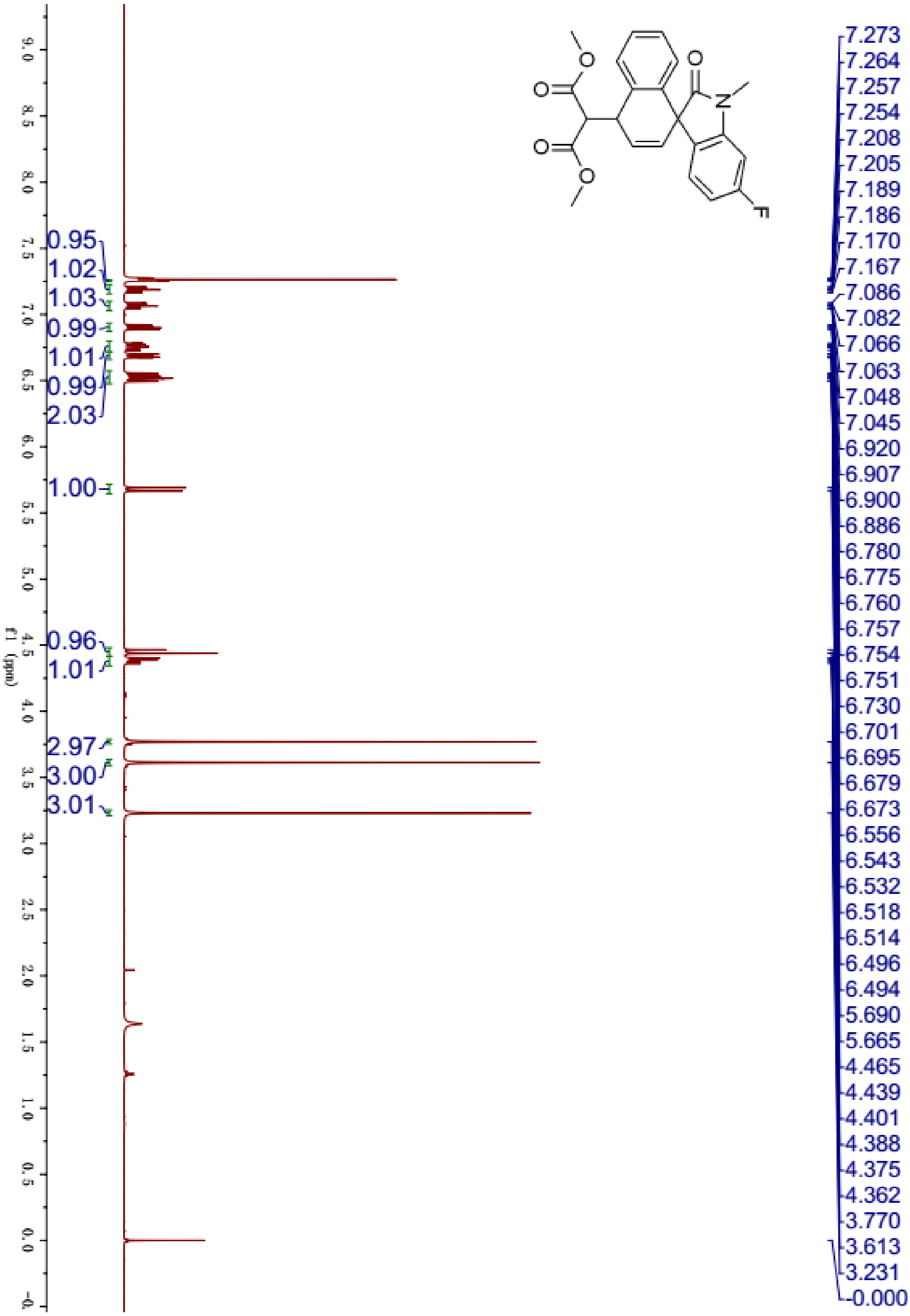
7.273
7.264
7.257
7.254
7.208
7.205
7.189
7.186
7.170
7.167
7.086
7.082
7.066
7.063
7.048
7.045
6.920
6.907
6.900
6.886
6.780
6.775
6.760
6.757
6.754
6.751
6.730
6.701
6.695
6.679
6.673
6.556
6.543
6.532
6.518
6.514
6.496
6.494
5.690
5.665
4.465
4.439
4.401
4.388
4.375
4.362
3.770
3.613
3.231
-0.000
0.95
1.02
1.03
0.99
1.01
0.99
2.03
1.00
0.96
1.01
2.97
3.00
3.01
f1 (ppm)
F

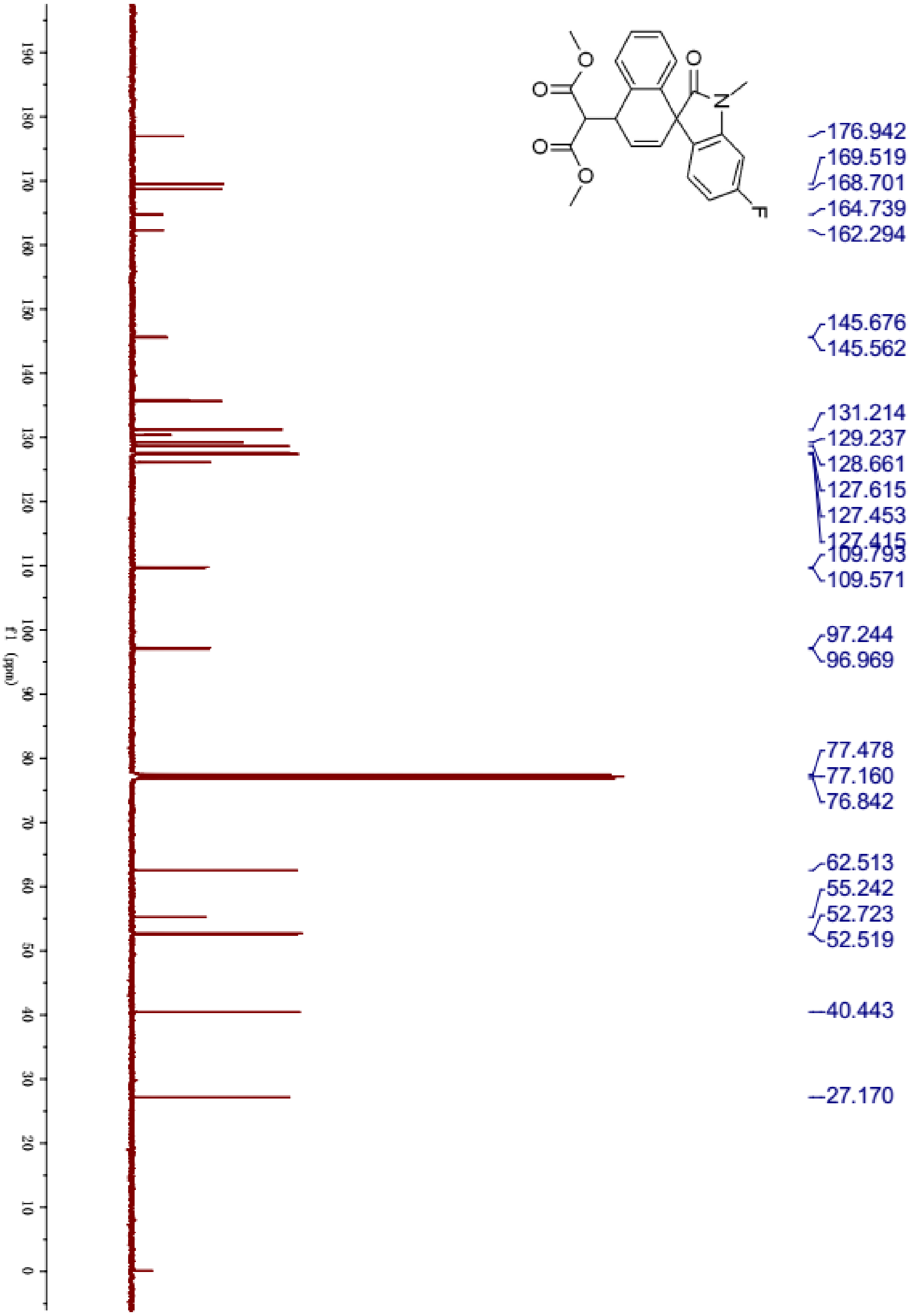

O
N
F
176.942
169.519
168.701
164.739
162.294
145.676
145.562
131.214
129.237
128.661
127.615
127.453
127.415
109.793
109.571
97.244
96.969
77.478
77.160
76.842
62.513
55.242
52.723
52.519
40.443
27.170
f1 (ppm)

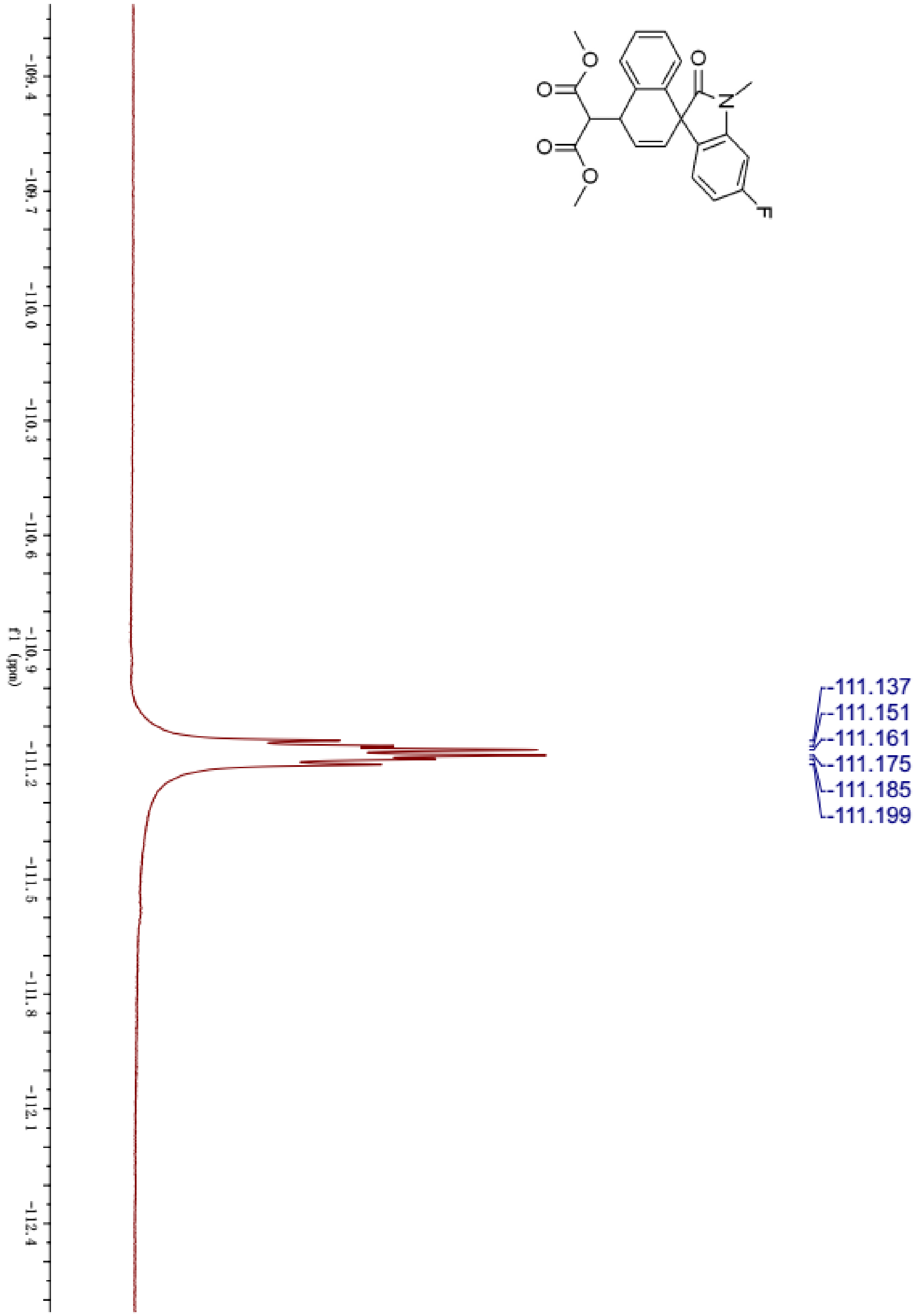
-111.137
-111.151
-111.161
-111.175
-111.185
-111.199
-109.4
-109.7
-110.0
-110.3
-110.6
-110.9
-111.2
-111.5
-111.8
-112.1
-112.4
f1 (ppm)
O
O
O
O
O
N
F

3g

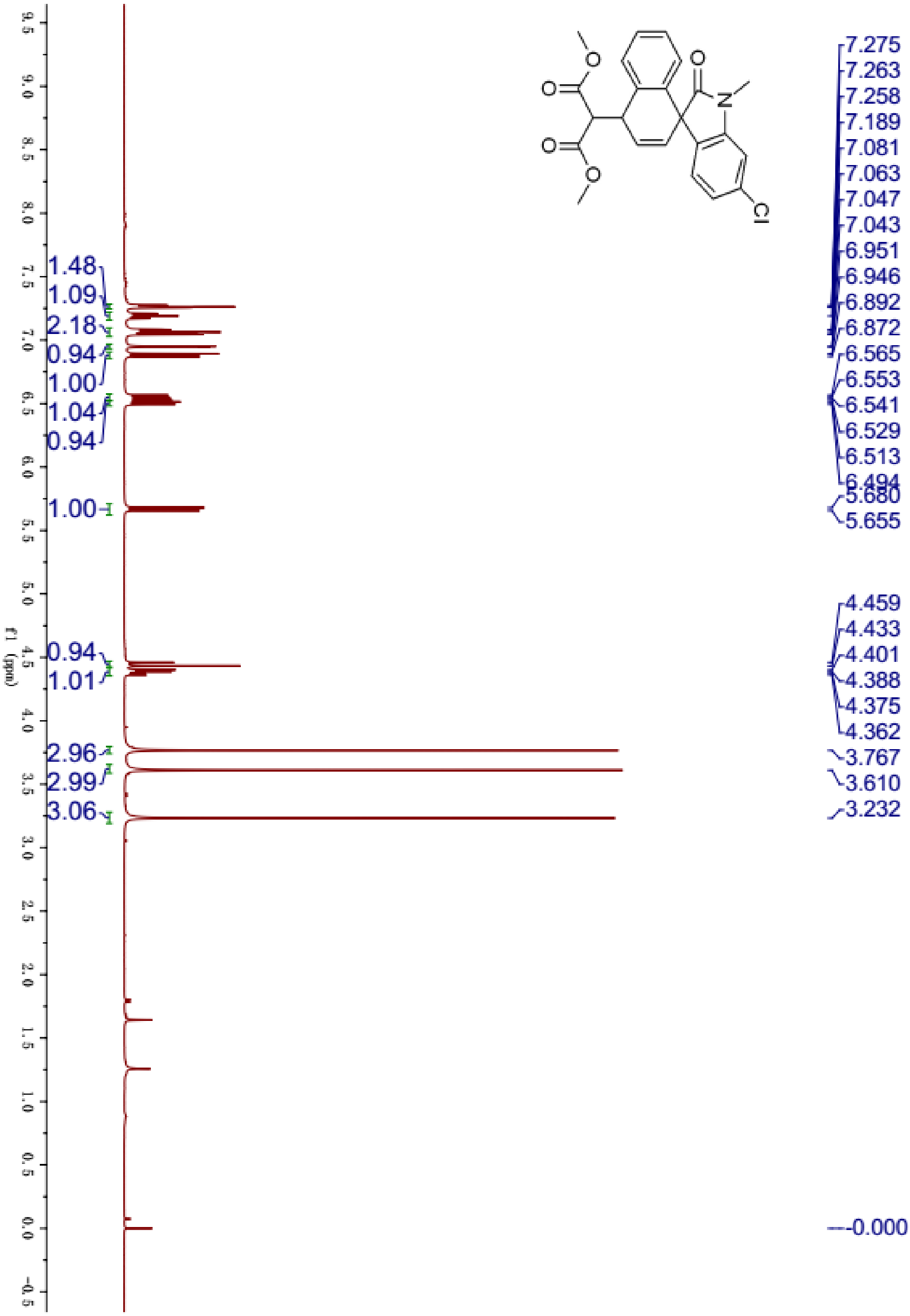
7.275
7.263
7.258
7.189
7.081
7.063
7.047
7.043
6.951
6.946
6.892
6.872
6.565
6.553
6.541
6.529
6.513
6.494
5.680
5.655
4.459
4.433
4.401
4.388
4.375
4.362
3.767
3.610
3.232
-0.000
1.48
1.09
2.18
0.94
1.00
1.04
0.94
1.00
0.94
1.01
2.96
2.99
3.06
f1 (ppm)
Cl

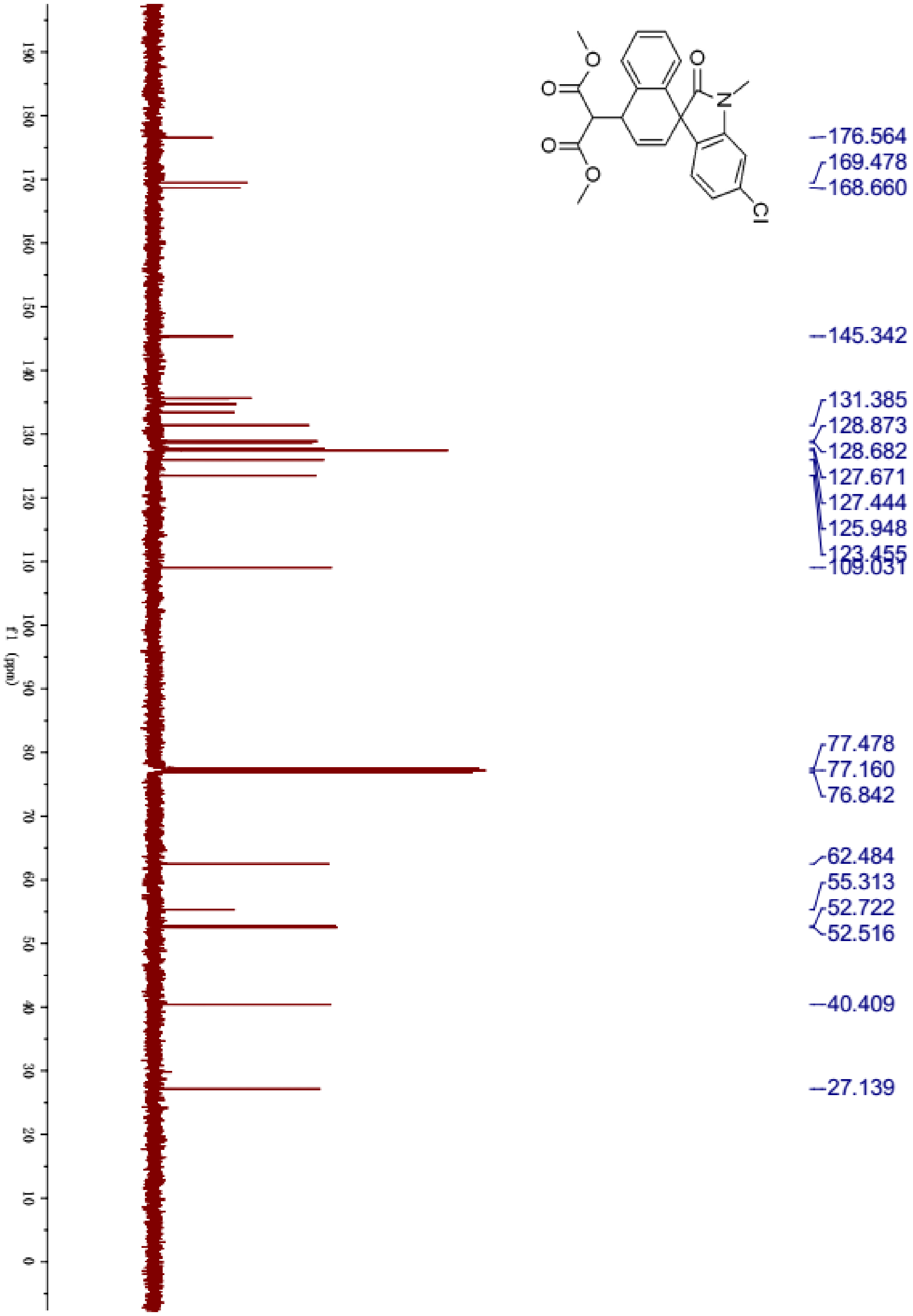

176.564
169.478
168.660
145.342
131.385
128.873
128.682
127.671
127.444
125.948
123.455
109.031
77.478
77.160
76.842
62.484
55.313
52.722
52.516
40.409
27.139
f1 (ppm)
O
N
Cl

3h

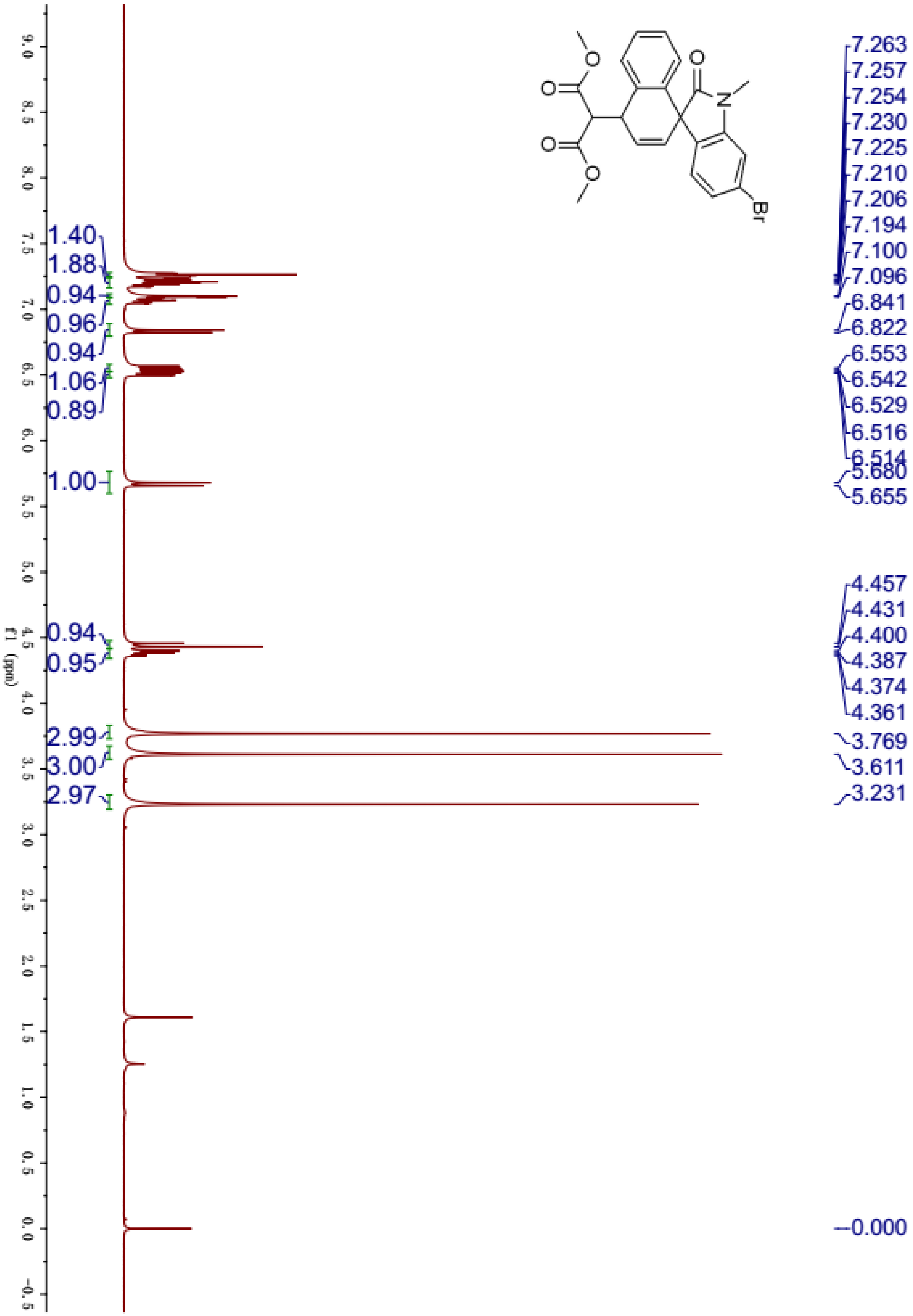
7.263
7.257
7.254
7.230
7.225
7.210
7.206
7.194
7.100
7.096
6.841
6.822
6.553
6.542
6.529
6.516
6.514
5.680
5.655
4.457
4.431
4.400
4.387
4.374
4.361
3.769
3.611
3.231
0.000
1.40
1.88
0.94
0.96
0.94
1.06
0.89
1.00
0.94
0.95
2.99
3.00
2.97
f1 (ppm)
Br

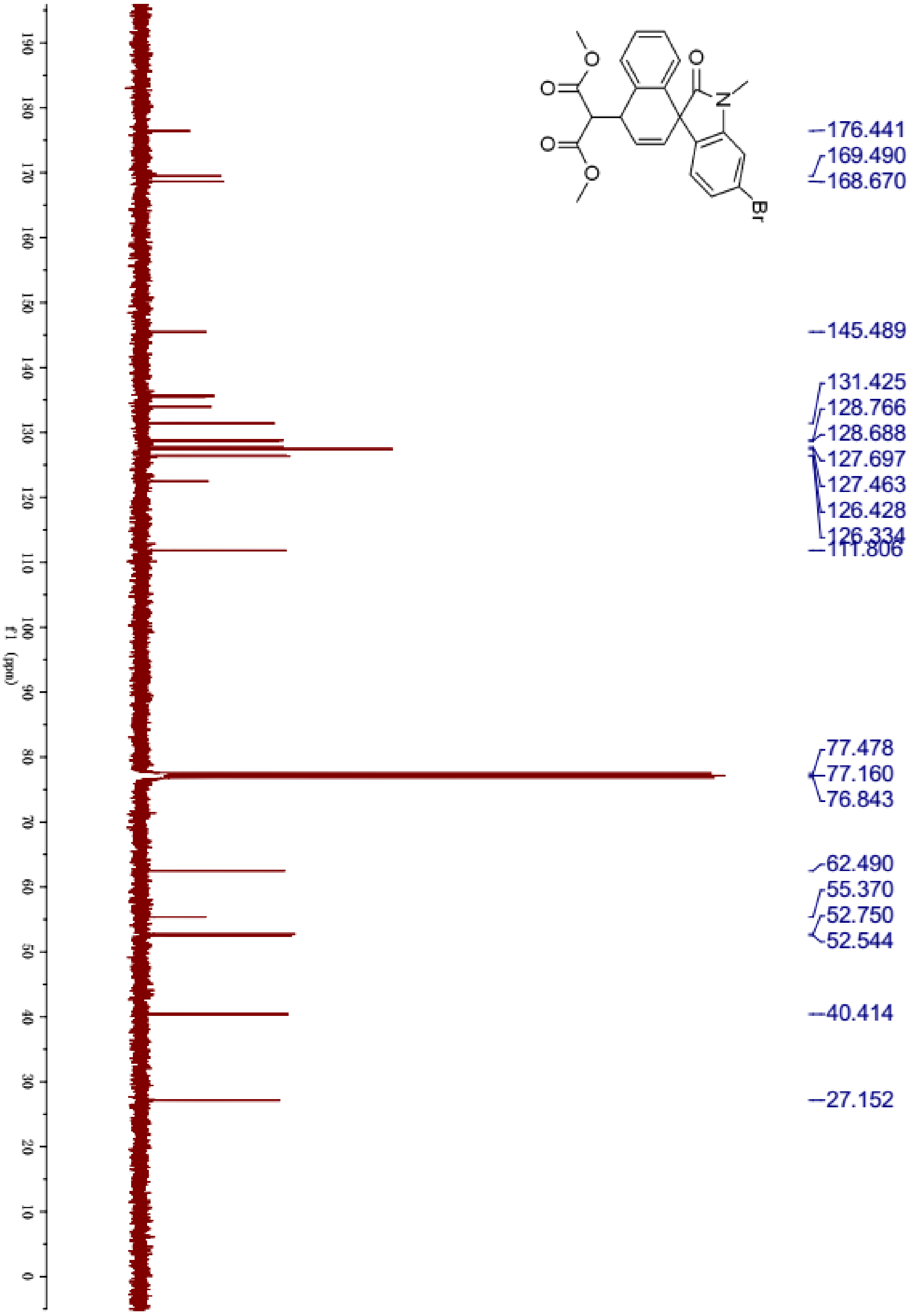

O
O
O
O
O
N
Br
176.441
169.490
168.670
145.489
131.425
128.766
128.688
127.697
127.463
126.428
126.334
111.806
77.478
77.160
76.843
62.490
55.370
52.750
52.544
40.414
27.152
f1 (ppm)

3i

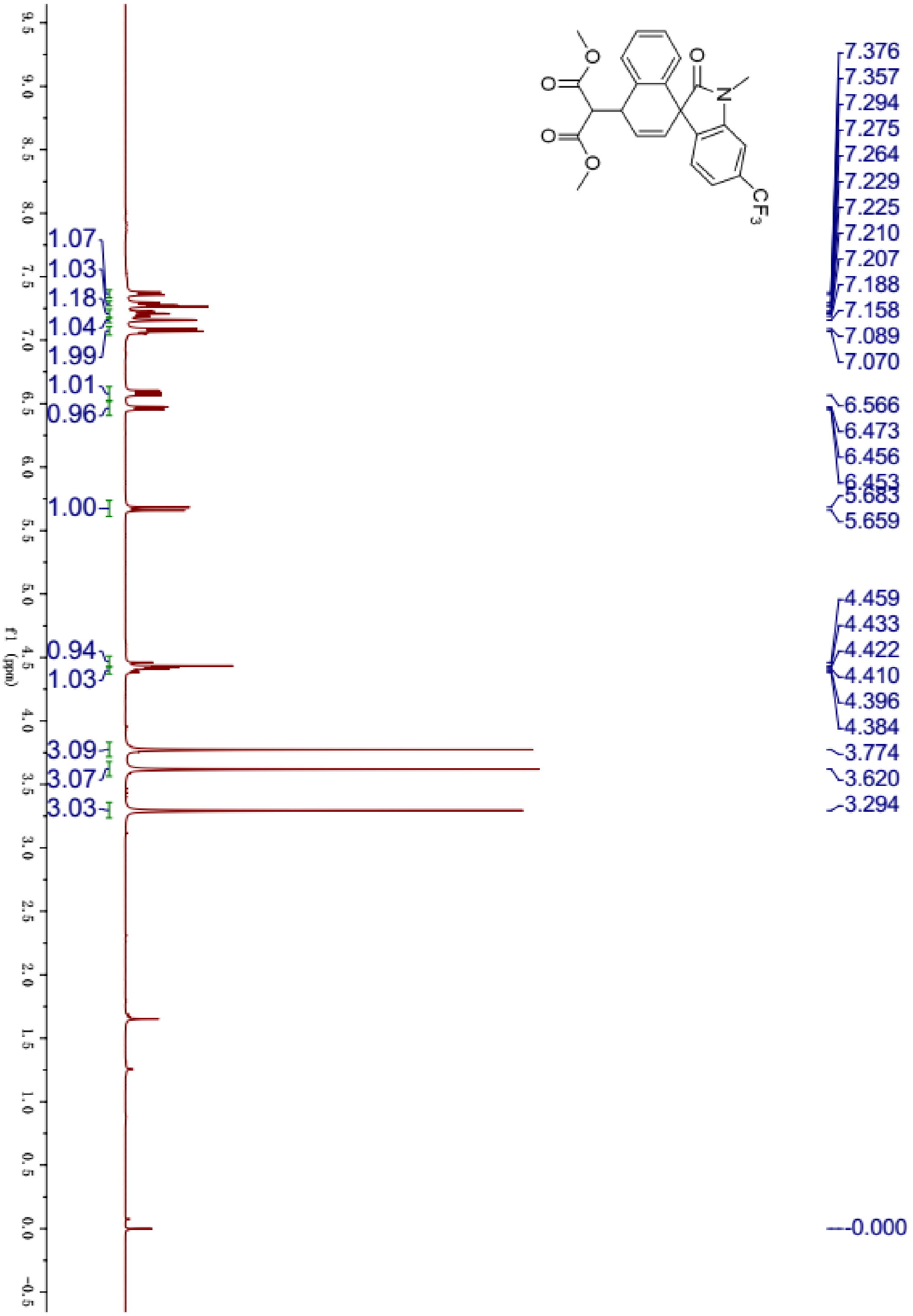
7.376
7.357
7.294
7.275
7.264
7.229
7.225
7.210
7.207
7.188
7.158
7.089
7.070
6.566
6.473
6.456
6.453
5.683
5.659
4.459
4.433
4.422
4.410
4.396
4.384
3.774
3.620
3.294
-0.000
1.07
1.03
1.18
1.04
1.99
1.01
0.96
1.00
0.94
1.03
3.09
3.07
3.03
f1 (ppm)
CF3

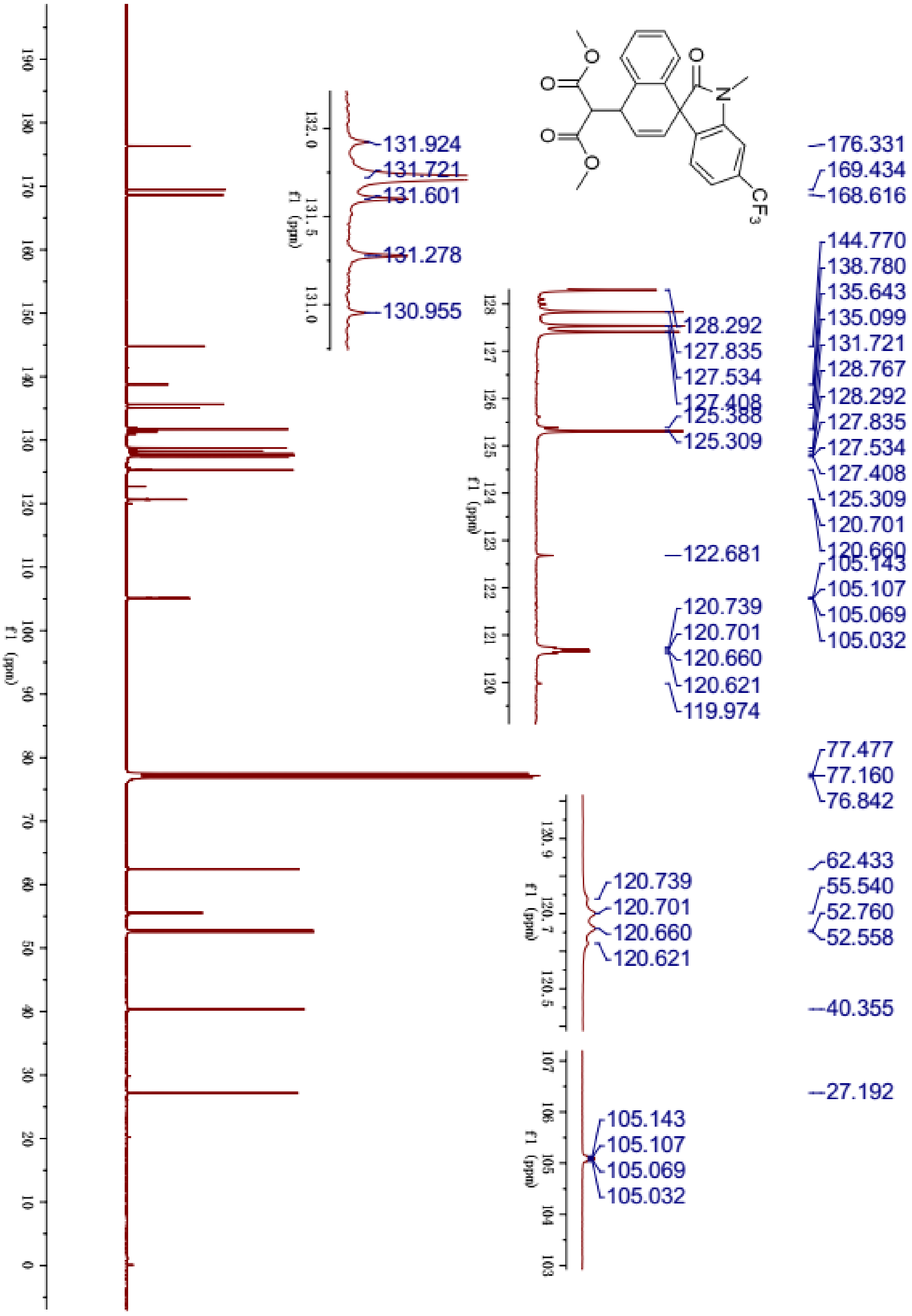
176.331
169.434
168.616
144.770
138.780
135.643
135.099
131.721
128.767
128.292
127.835
127.534
127.408
125.309
120.701
120.660
105.143
105.107
105.069
105.032
77.477
77.160
76.842
62.433
55.540
52.760
52.558
40.355
27.192
131.924
131.721
131.601
131.278
130.955
128.292
127.835
127.534
127.408
125.388
125.309
122.681
120.739
120.701
120.660
120.621
119.974
120.739
120.701
120.660
120.621
105.143
105.107
105.069
105.032
CF3

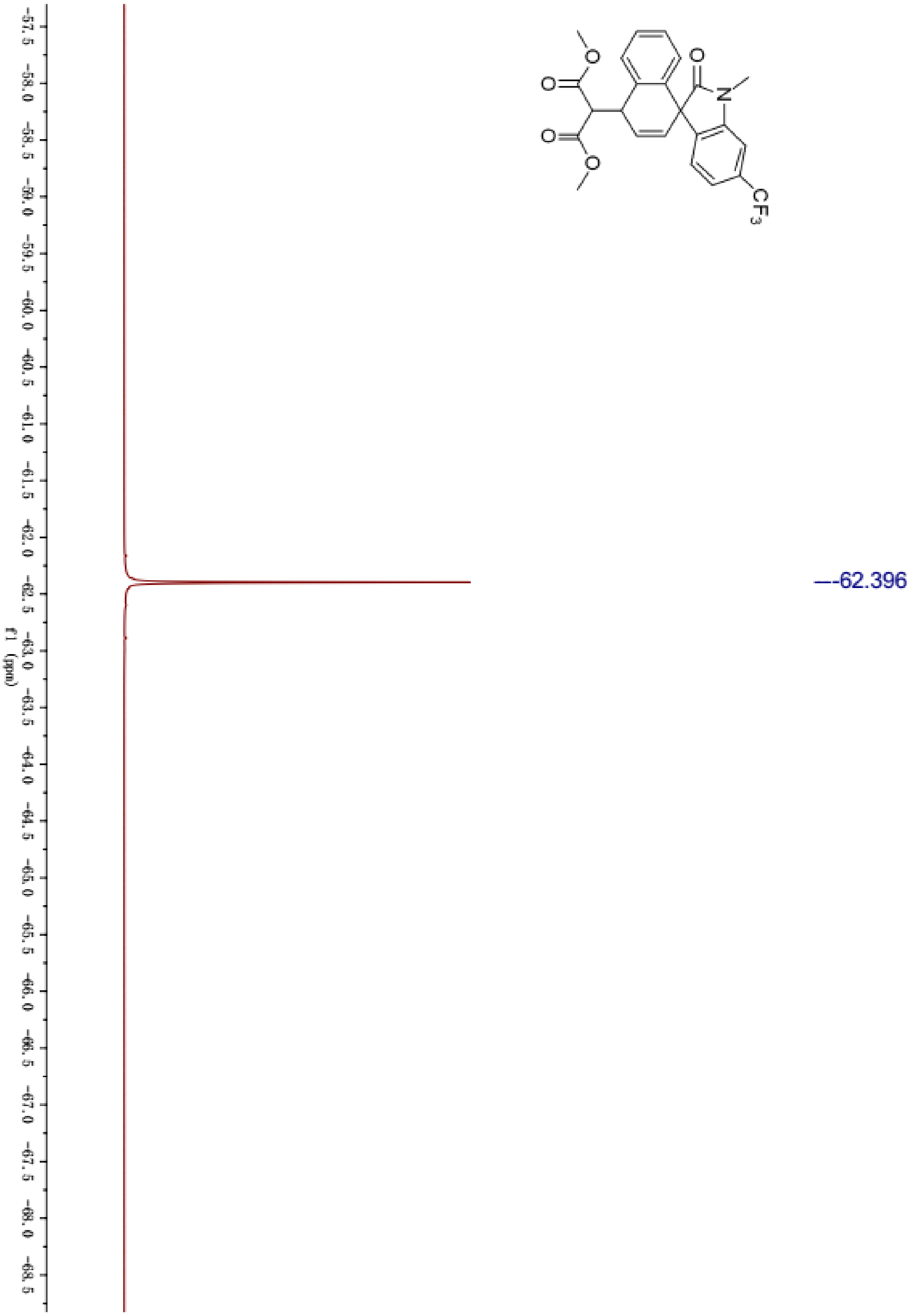

--62.396
f1 (ppm)
-57.5 -58.0 -58.5 -59.0 -59.5 -60.0 -60.5 -61.0 -61.5 -62.0 -62.5 -63.0 -63.5 -64.0 -64.5 -65.0 -65.5 -66.0 -66.5 -67.0 -67.5 -68.0 -68.5
O
N
CF_3

3j

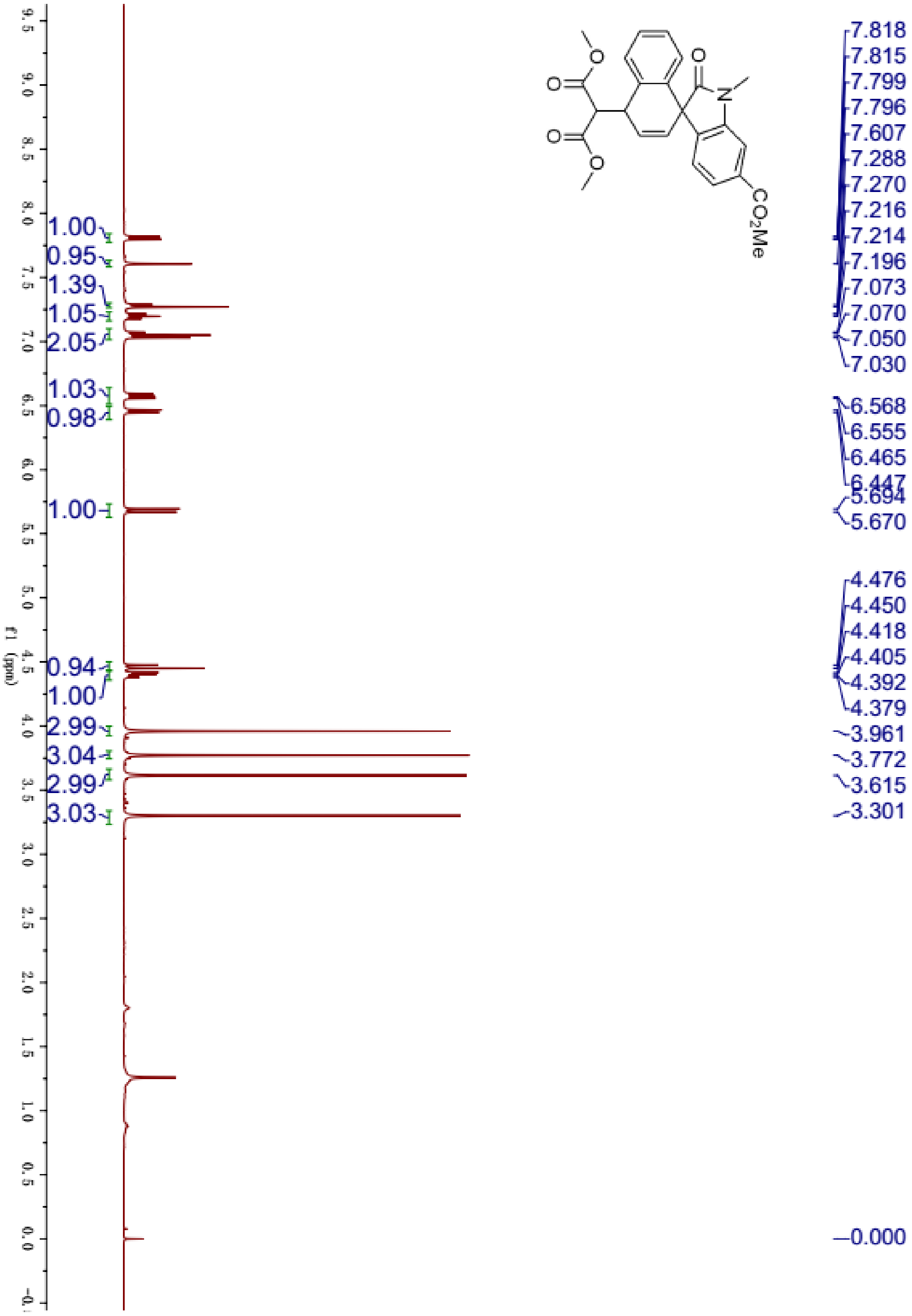
7.818
7.815
7.799
7.796
7.607
7.288
7.270
7.216
7.214
7.196
7.073
7.070
7.050
7.030
6.568
6.555
6.465
6.447
5.694
5.670
4.476
4.450
4.418
4.405
4.392
4.379
3.961
3.772
3.615
3.301
0.000
1.00
0.95
1.39
1.05
2.05
1.03
0.98
1.00
0.94
1.00
2.99
3.04
2.99
3.03
f1 (ppm)
CO2Me

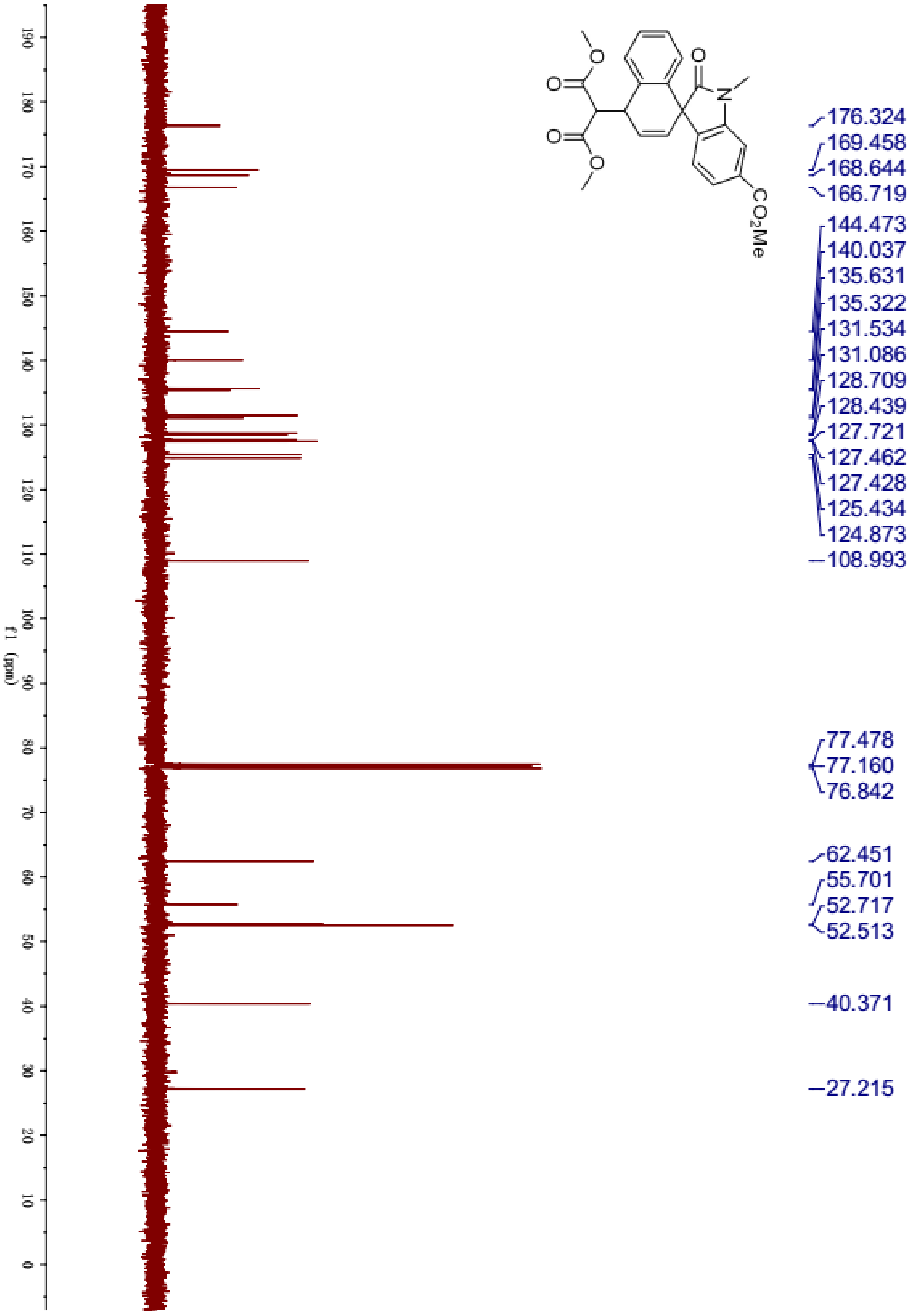
176.324
169.458
168.644
166.719
144.473
140.037
135.631
135.322
131.534
131.086
128.709
128.439
127.721
127.462
127.428
125.434
124.873
108.993
77.478
77.160
76.842
62.451
55.701
52.717
52.513
40.371
27.215
f1 (ppm)

3k

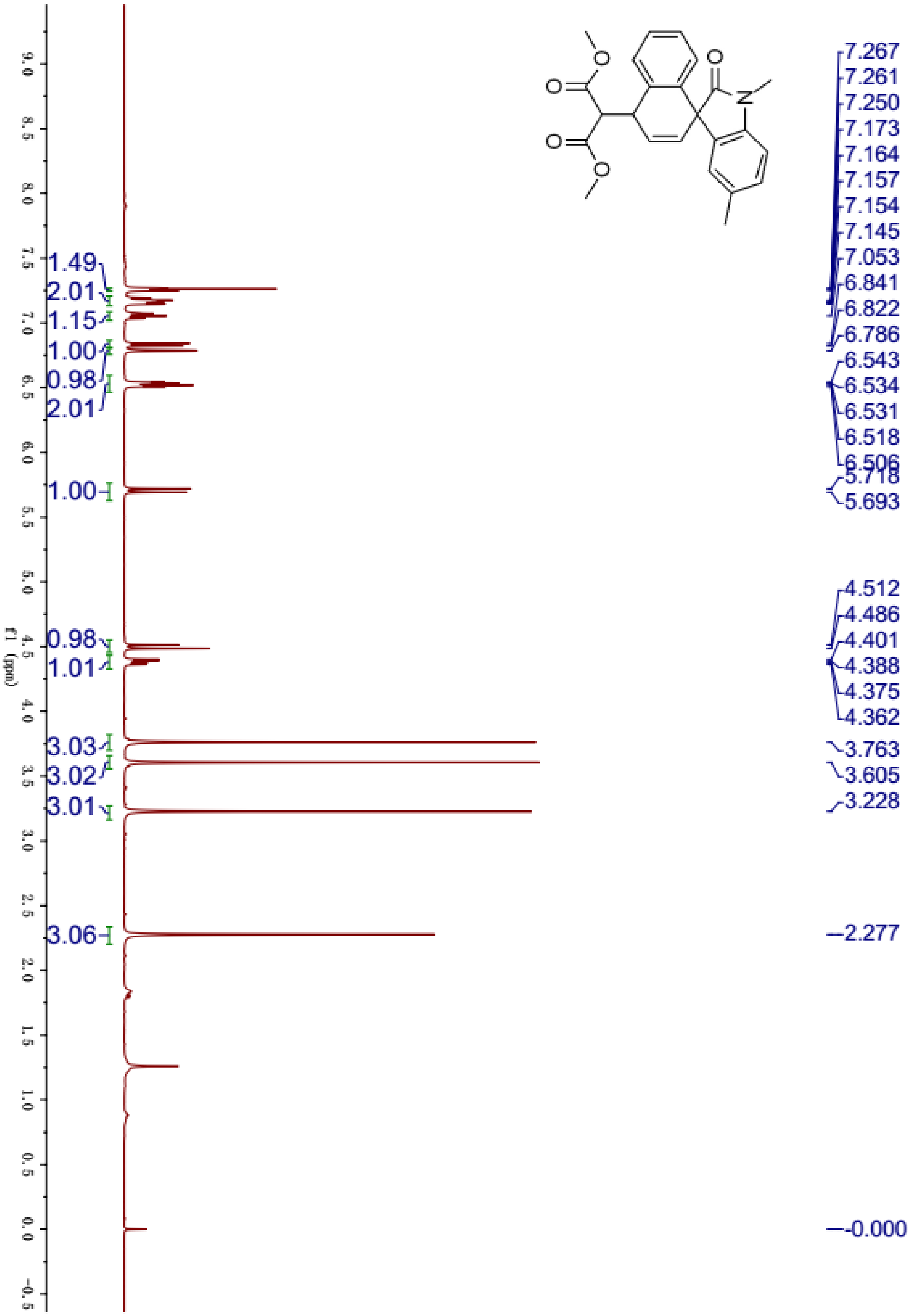
7.267
7.261
7.250
7.173
7.164
7.157
7.154
7.145
7.053
6.841
6.822
6.786
6.543
6.534
6.531
6.518
6.506
5.718
5.693
4.512
4.486
4.401
4.388
4.375
4.362
3.763
3.605
3.228
2.277
-0.000
1.49
2.01
1.15
1.00
0.98
2.01
1.00
0.98
1.01
3.03
3.02
3.01
3.06
f1 (ppm)

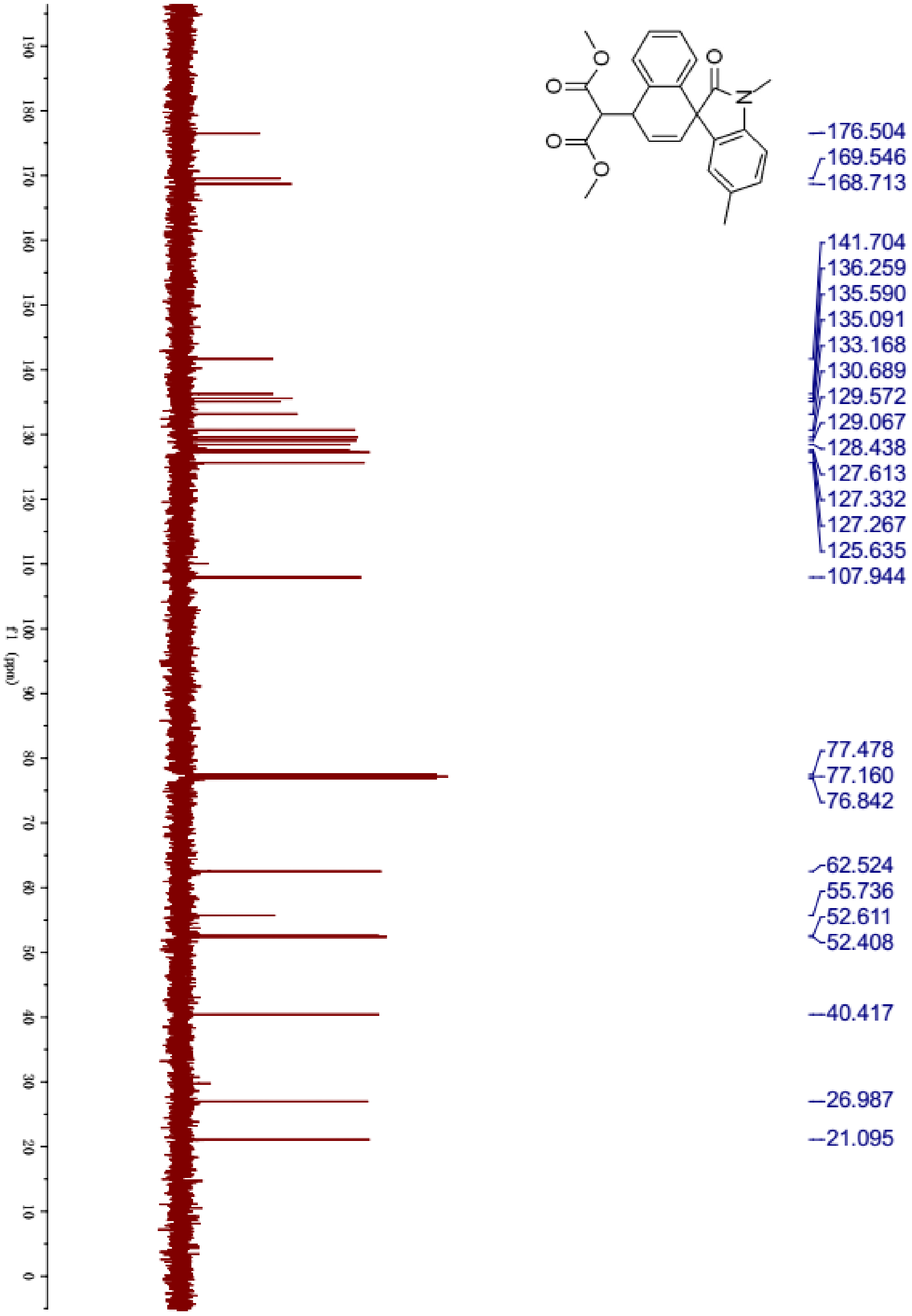
176.504
169.546
168.713
141.704
136.259
135.590
135.091
133.168
130.689
129.572
129.067
128.438
127.613
127.332
127.267
125.635
107.944
77.478
77.160
76.842
62.524
55.736
52.611
52.408
40.417
26.987
21.095
f1 (ppm)

3l

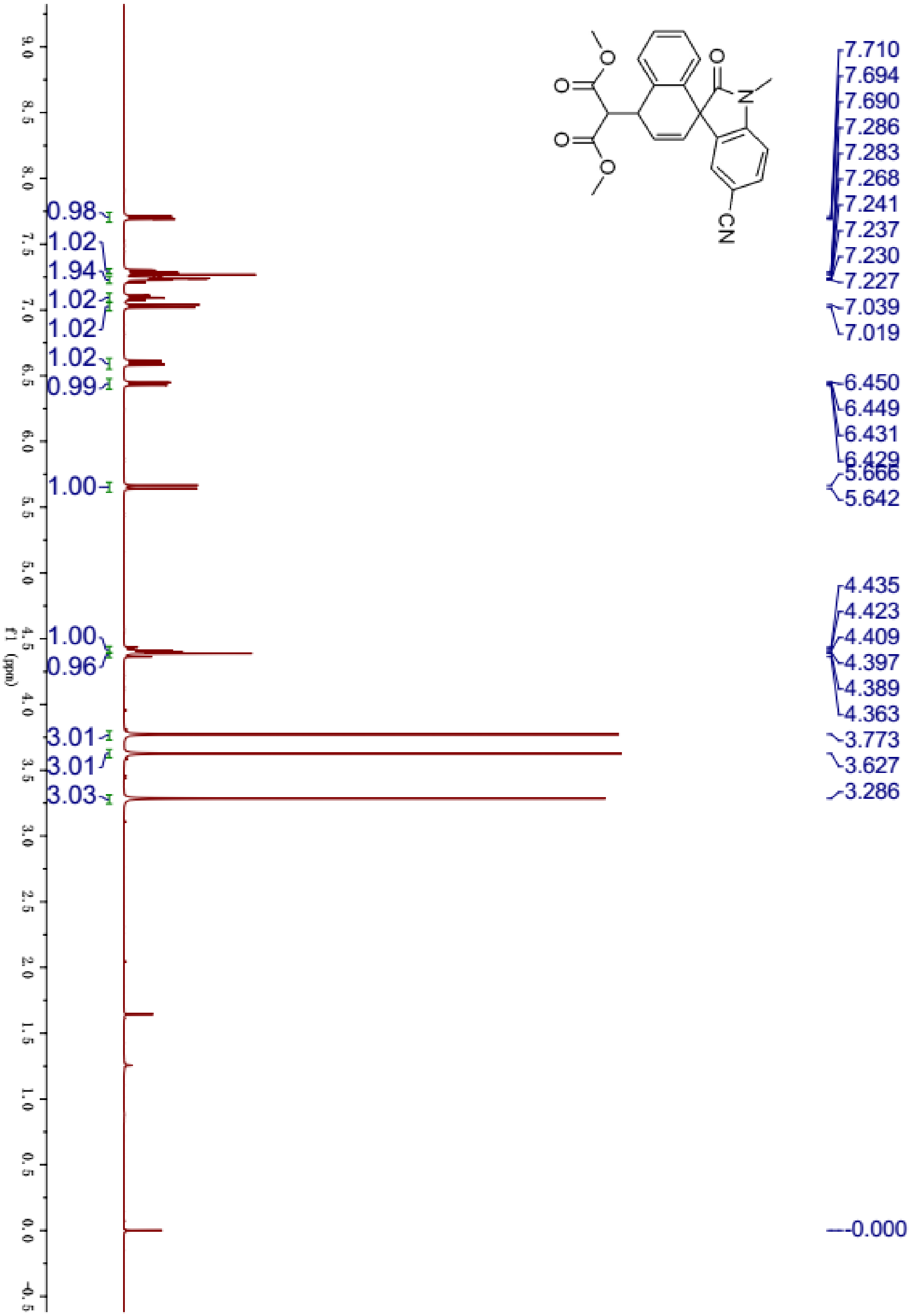
7.710
7.694
7.690
7.286
7.283
7.268
7.241
7.237
7.230
7.227
7.039
7.019
6.450
6.449
6.431
6.429
5.666
5.642
4.435
4.423
4.409
4.397
4.389
4.363
3.773
3.627
3.286
-0.000
0.98
1.02
1.94
1.02
1.02
1.02
0.99
1.00
1.00
0.96
3.01
3.01
3.03
f1 (ppm)
CN
O
N

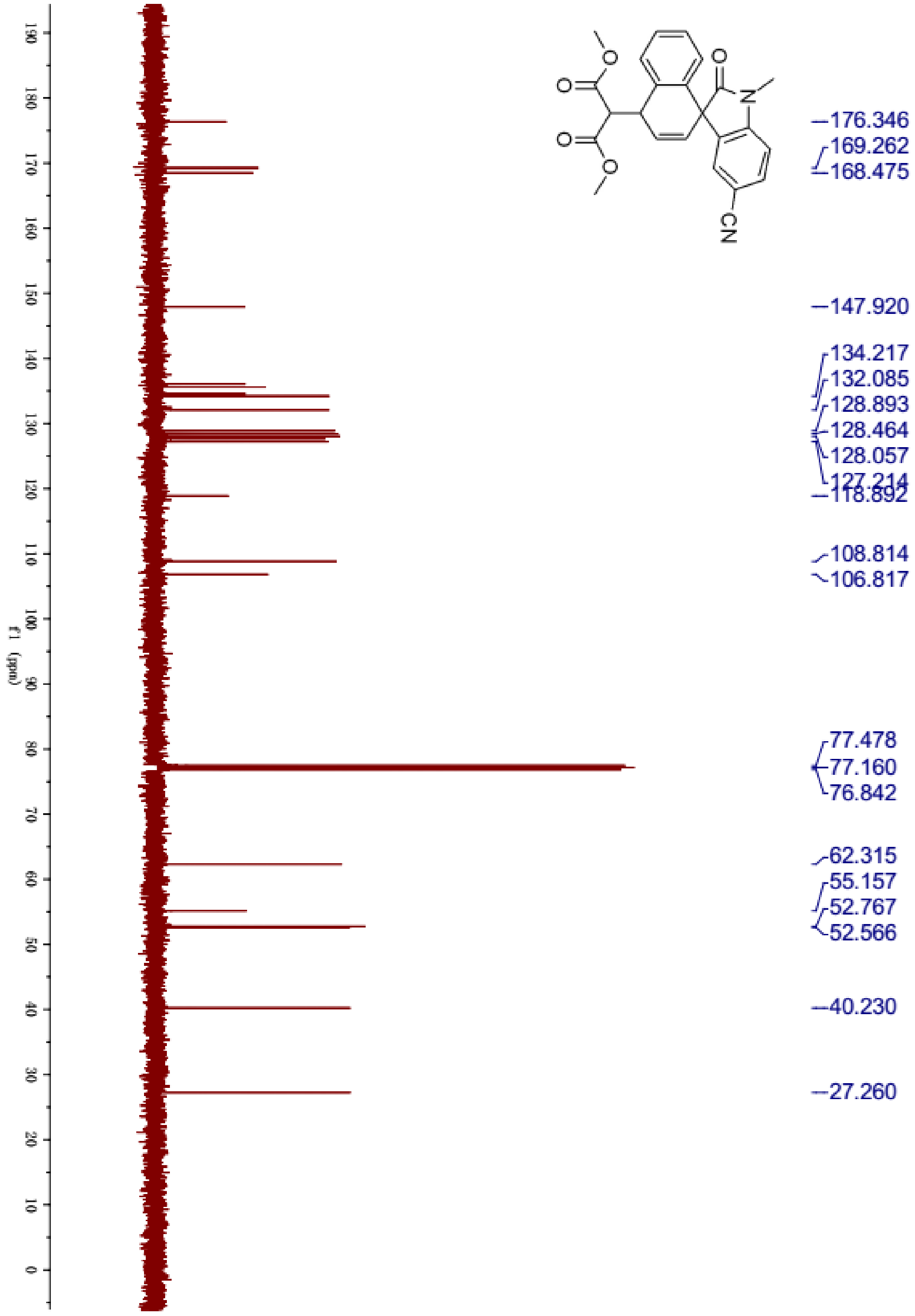

176.346
169.262
168.475
147.920
134.217
132.085
128.893
128.464
128.057
127.214
118.892
108.814
106.817
77.478
77.160
76.842
62.315
55.157
52.767
52.566
40.230
27.260
f1 (ppm)
CN

3m

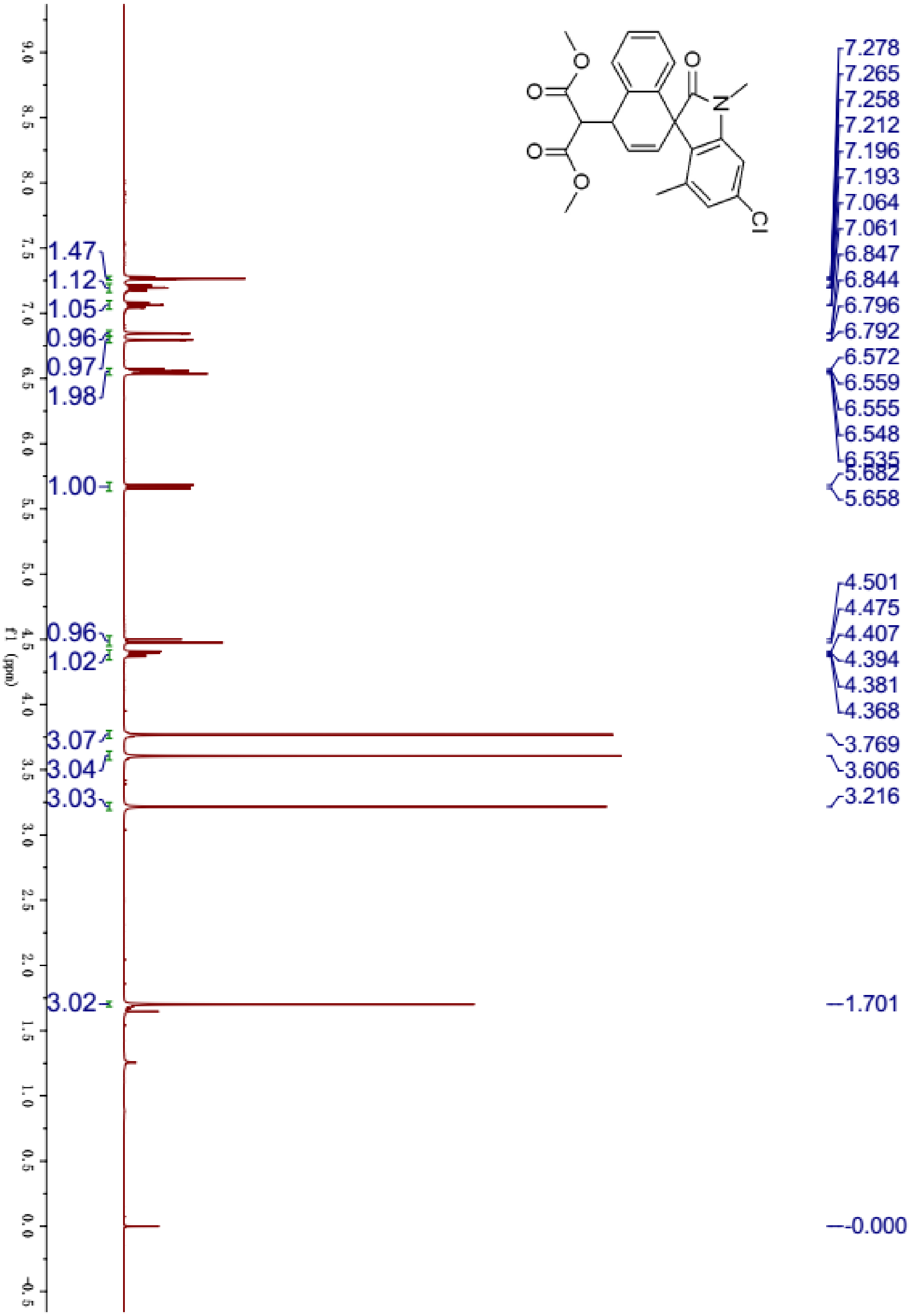
7.278
7.265
7.258
7.212
7.196
7.193
7.064
7.061
6.847
6.844
6.796
6.792
6.572
6.559
6.555
6.548
6.535
5.682
5.658
4.501
4.475
4.407
4.394
4.381
4.368
3.769
3.606
3.216
1.701
-0.000
1.47
1.12
1.05
0.96
0.97
1.98
1.00
0.96
1.02
3.07
3.04
3.03
3.02
f1 (ppm)
O
N
Cl

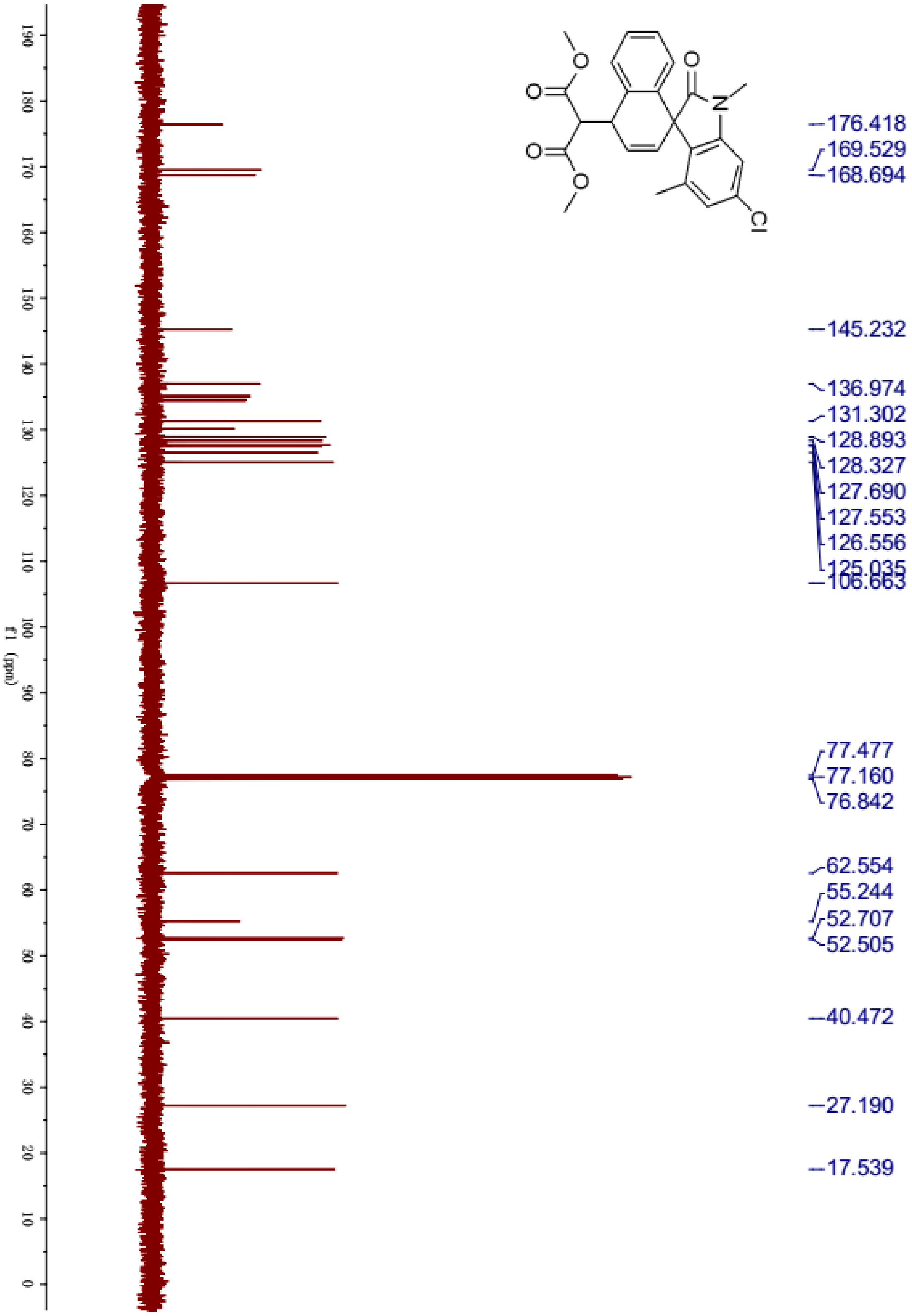
O
O
O
O
O
N
Cl
176.418
169.529
168.694
145.232
136.974
131.302
128.893
128.327
127.690
127.553
126.556
125.035
106.663
77.477
77.160
76.842
62.554
55.244
52.707
52.505
40.472
27.190
17.539
f1 (ppm)

3n

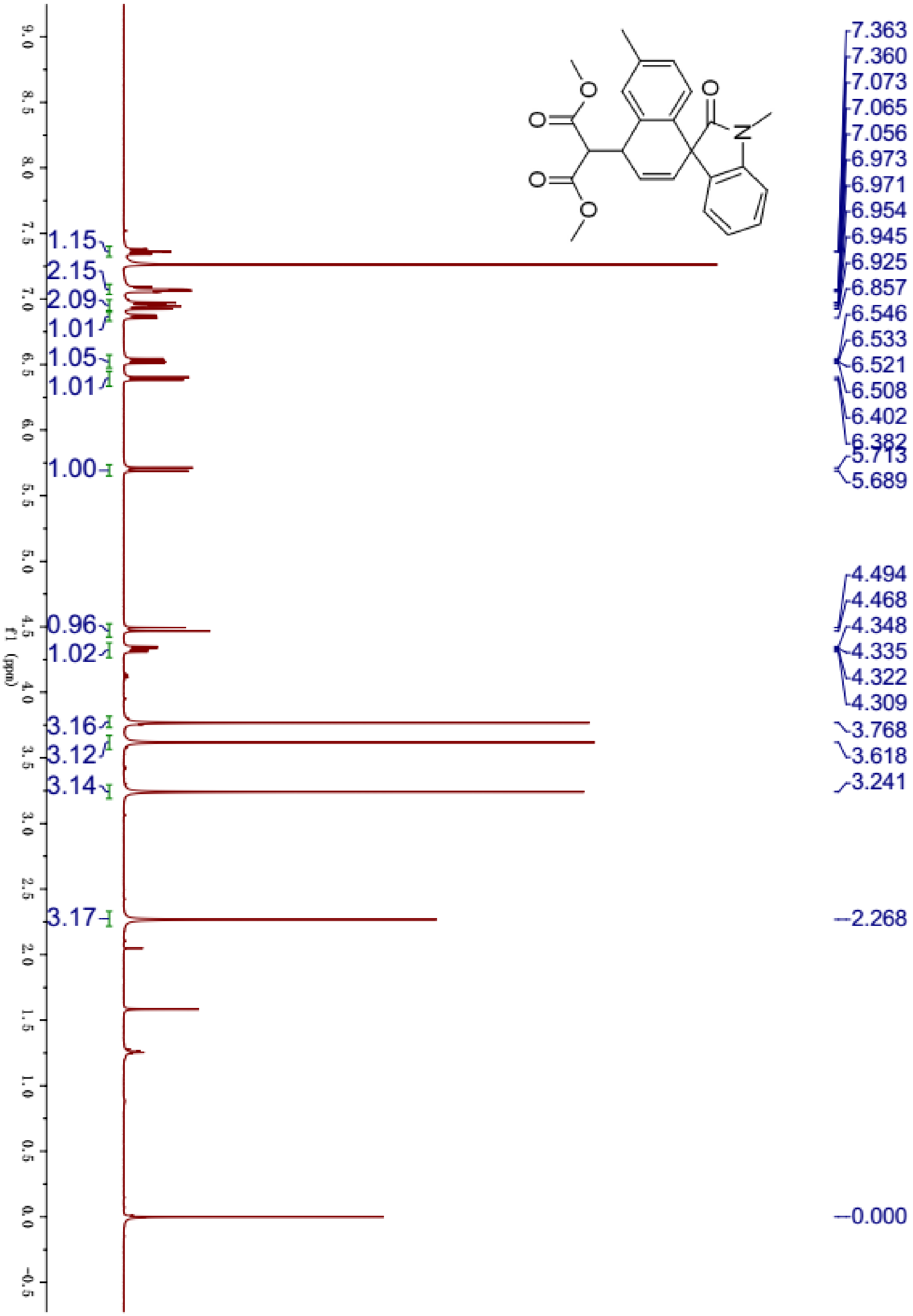

7.363
7.360
7.073
7.065
7.056
6.973
6.971
6.954
6.945
6.925
6.857
6.546
6.533
6.521
6.508
6.402
6.382
5.713
5.689
4.494
4.468
4.348
4.335
4.322
4.309
3.768
3.618
3.241
2.268
0.000
1.15
2.15
2.09
1.01
1.05
1.01
1.00
0.96
1.02
3.16
3.12
3.14
3.17
f1 (ppm)

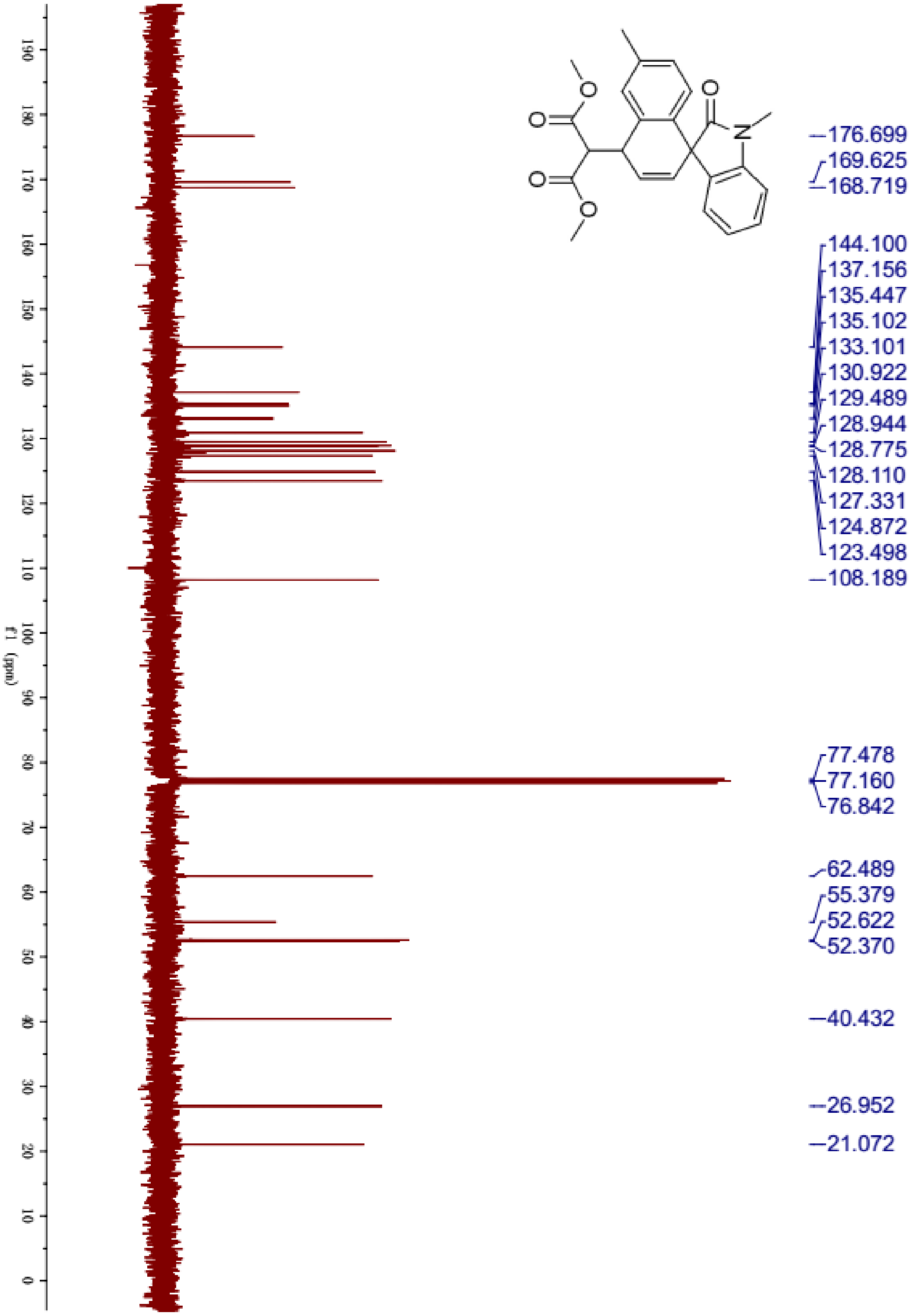

176.699
169.625
168.719
144.100
137.156
135.447
135.102
133.101
130.922
129.489
128.944
128.775
128.110
127.331
124.872
123.498
108.189
77.478
77.160
76.842
62.489
55.379
52.622
52.370
40.432
26.952
21.072
f1 (ppm)

3o

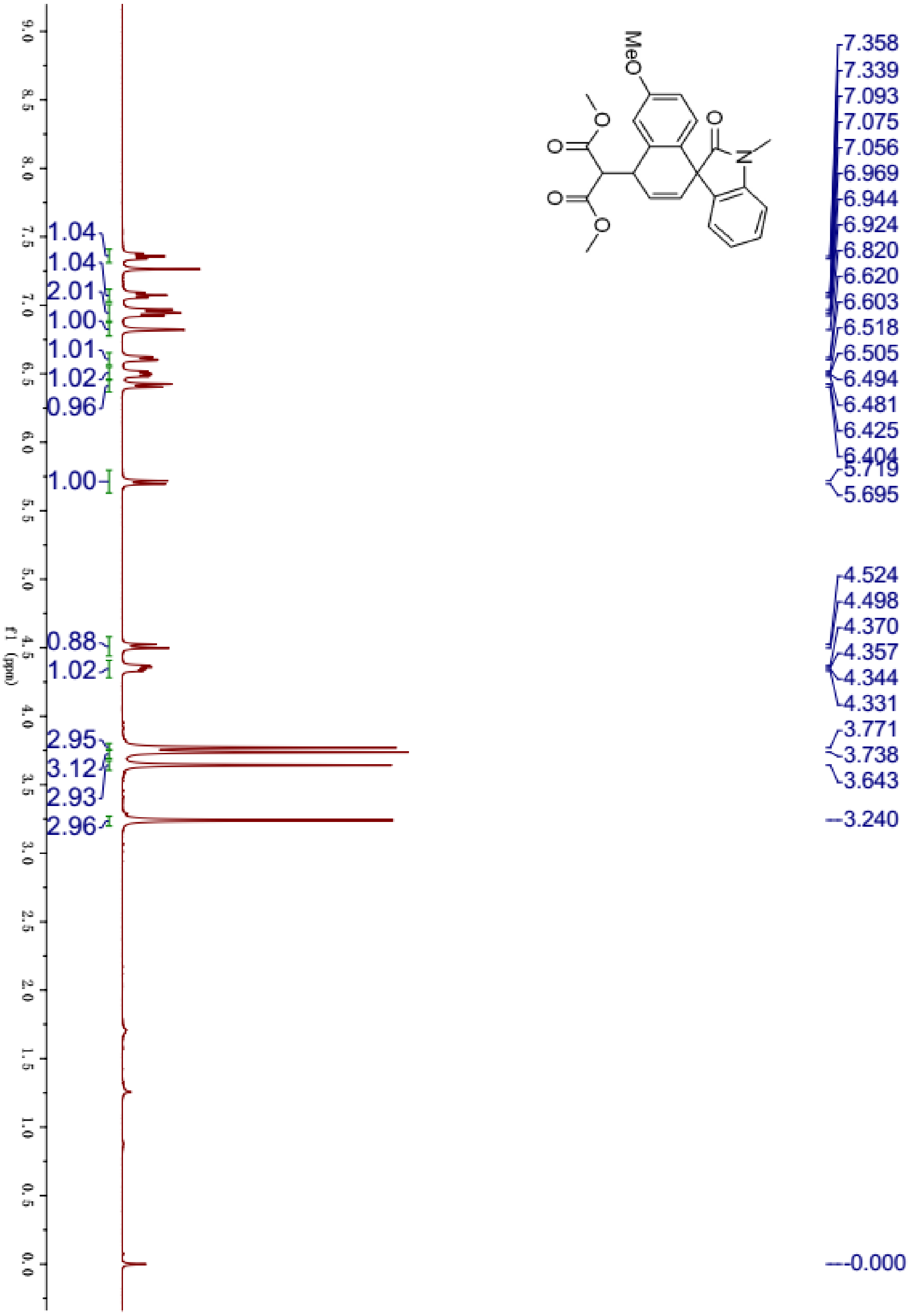
MeO
O
N
7.358
7.339
7.093
7.075
7.056
6.969
6.944
6.924
6.820
6.620
6.603
6.518
6.505
6.494
6.481
6.425
6.404
5.719
5.695
4.524
4.498
4.370
4.357
4.344
4.331
3.771
3.738
3.643
3.240
-0.000
1.04
1.04
2.01
1.00
1.01
1.02
0.96
1.00
0.88
1.02
2.95
3.12
2.93
2.96

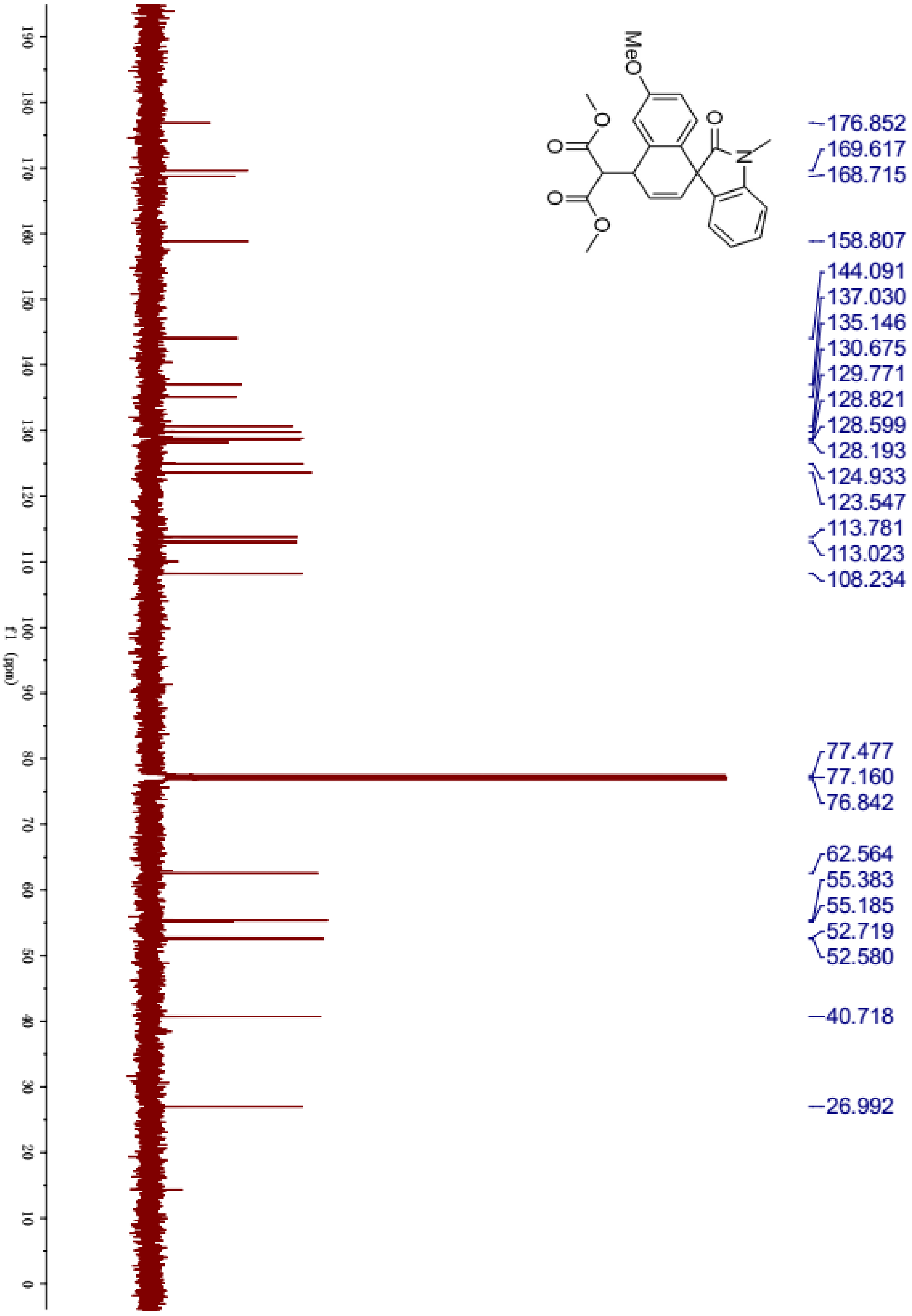
MeO
O
N
O
O
O
O
176.852
169.617
168.715
158.807
144.091
137.030
135.146
130.675
129.771
128.821
128.599
128.193
124.933
123.547
113.781
113.023
108.234
77.477
77.160
76.842
62.564
55.383
55.185
52.719
52.580
40.718
26.992
f1 (ppm)

3p

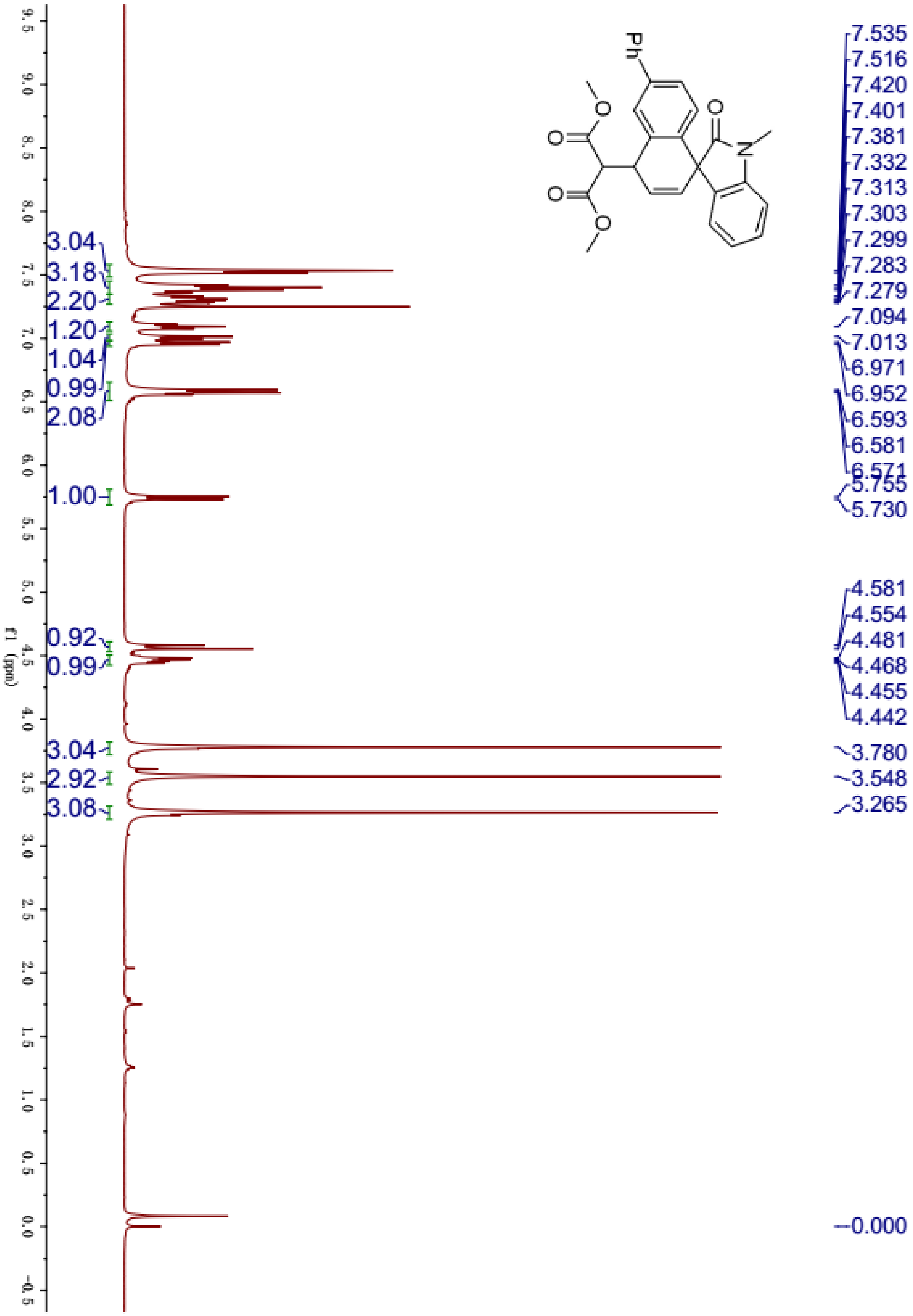

7.535
7.516
7.420
7.401
7.381
7.332
7.313
7.303
7.299
7.283
7.279
7.094
7.013
6.971
6.952
6.593
6.581
6.571
5.755
5.730
4.581
4.554
4.481
4.468
4.455
4.442
3.780
3.548
3.265
0.000
3.04
3.18
2.20
1.20
1.04
0.99
2.08
1.00
0.92
0.99
3.04
2.92
3.08
f1 (ppm)
Ph

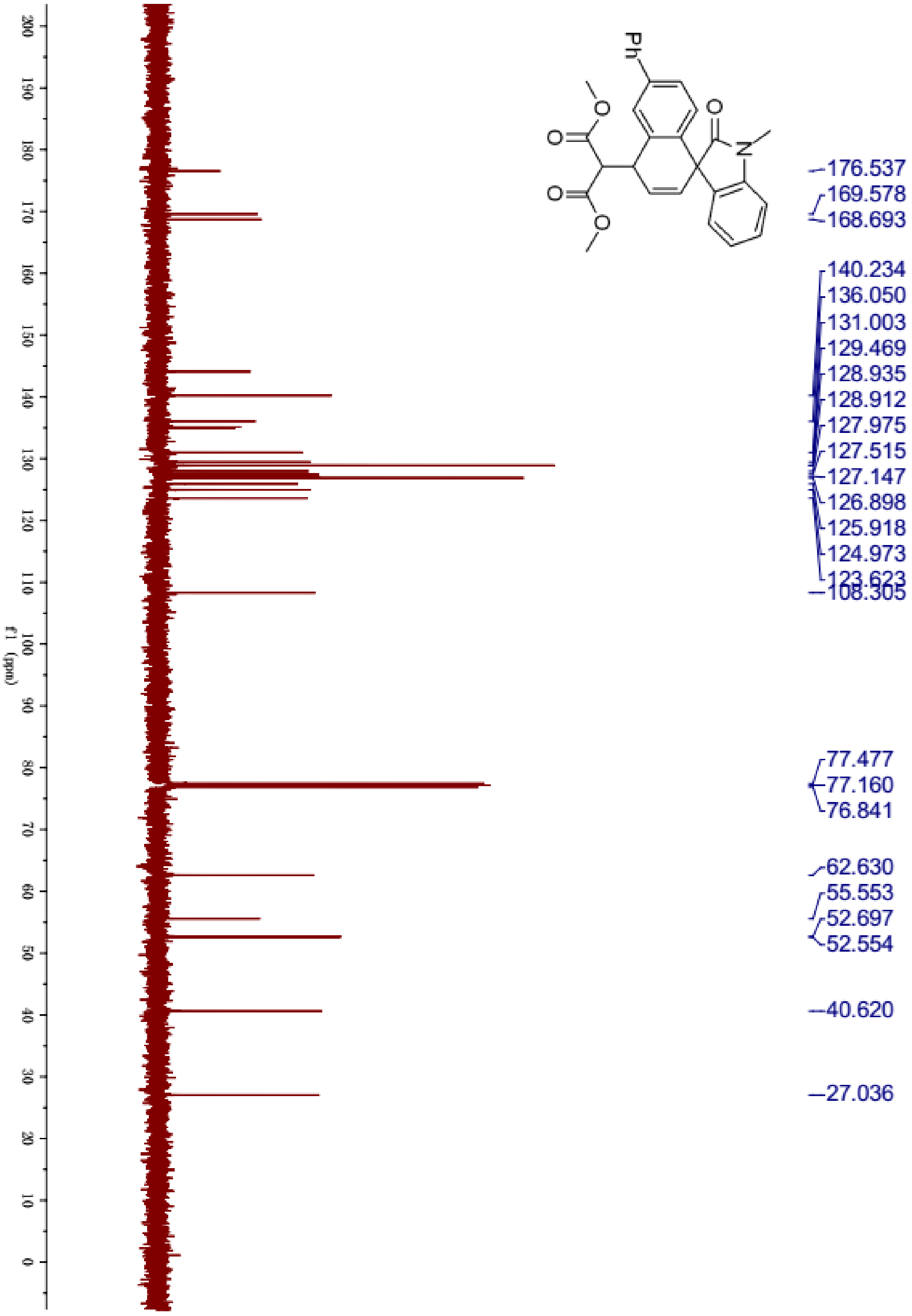
176.537
169.578
168.693
140.234
136.050
131.003
129.469
128.935
128.912
127.975
127.515
127.147
126.898
125.918
124.973
123.623
108.305
77.477
77.160
76.841
62.630
55.553
52.697
52.554
40.620
27.036
Ph
O
N
f1 (ppm)

3q

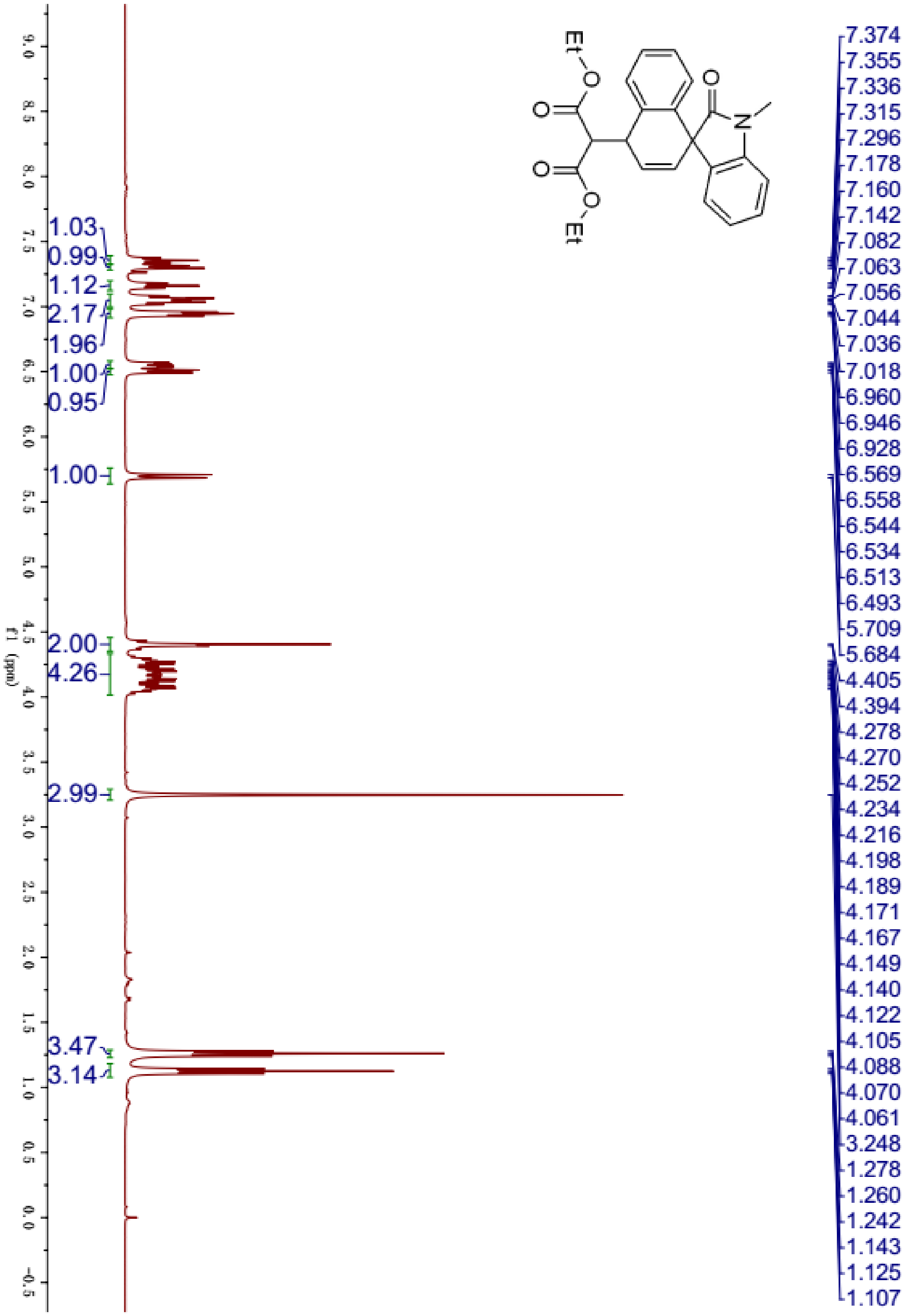

7.374
7.355
7.336
7.315
7.296
7.178
7.160
7.142
7.082
7.063
7.056
7.044
7.036
7.018
6.960
6.946
6.928
6.569
6.558
6.544
6.534
6.513
6.493
5.709
5.684
4.405
4.394
4.278
4.270
4.252
4.234
4.216
4.198
4.189
4.171
4.167
4.149
4.140
4.122
4.105
4.088
4.070
4.061
3.248
1.278
1.260
1.242
1.143
1.125
1.107
1.03
0.99
1.12
2.17
1.96
1.00
0.95
1.00
2.00
4.26
2.99
3.47
3.14
f1 (ppm)
Et
O
N

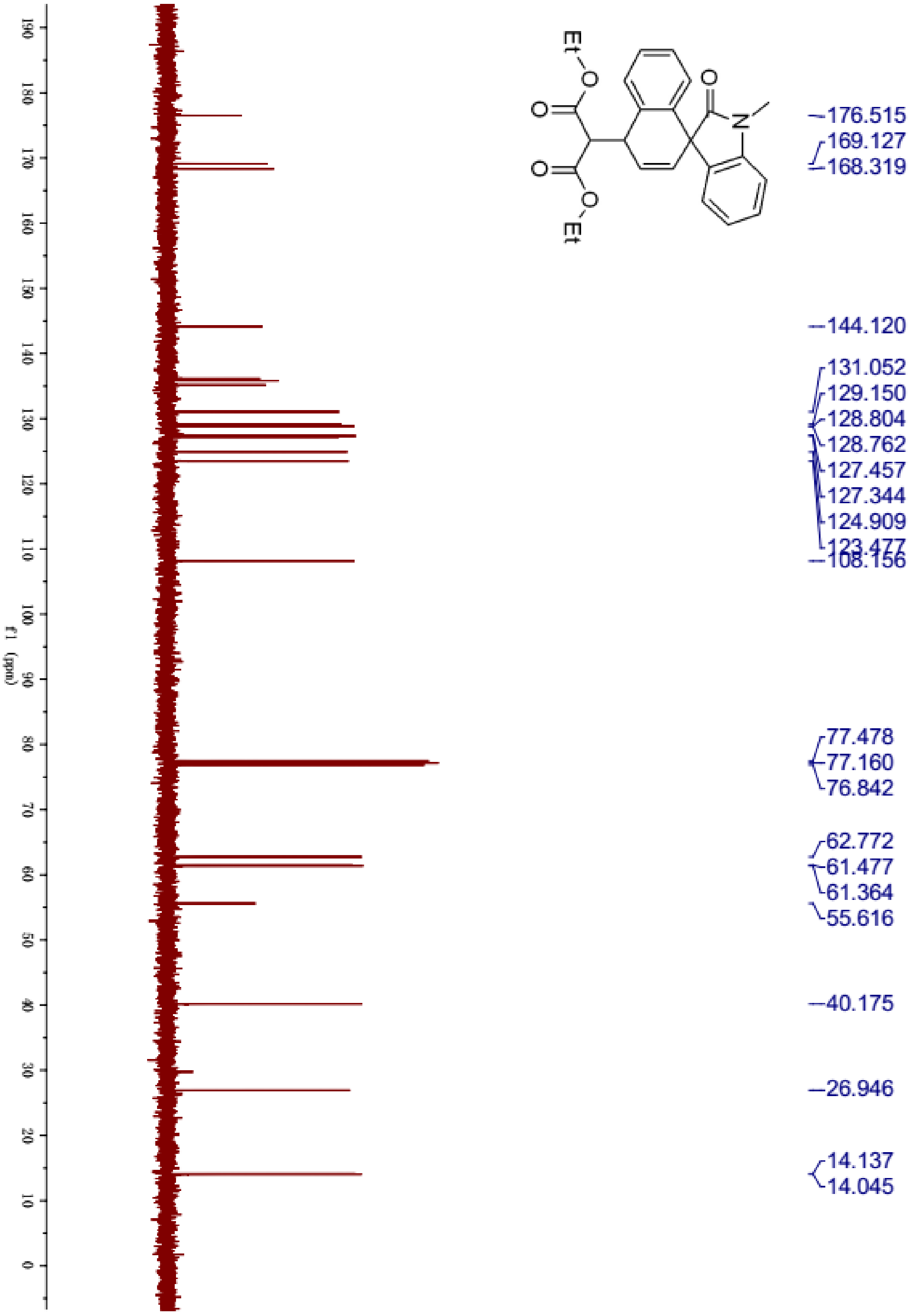

176.515
169.127
168.319
144.120
131.052
129.150
128.804
128.762
127.457
127.344
124.909
123.477
108.156
77.478
77.160
76.842
62.772
61.477
61.364
55.616
40.175
26.946
14.137
14.045
f1 (ppm)

3r

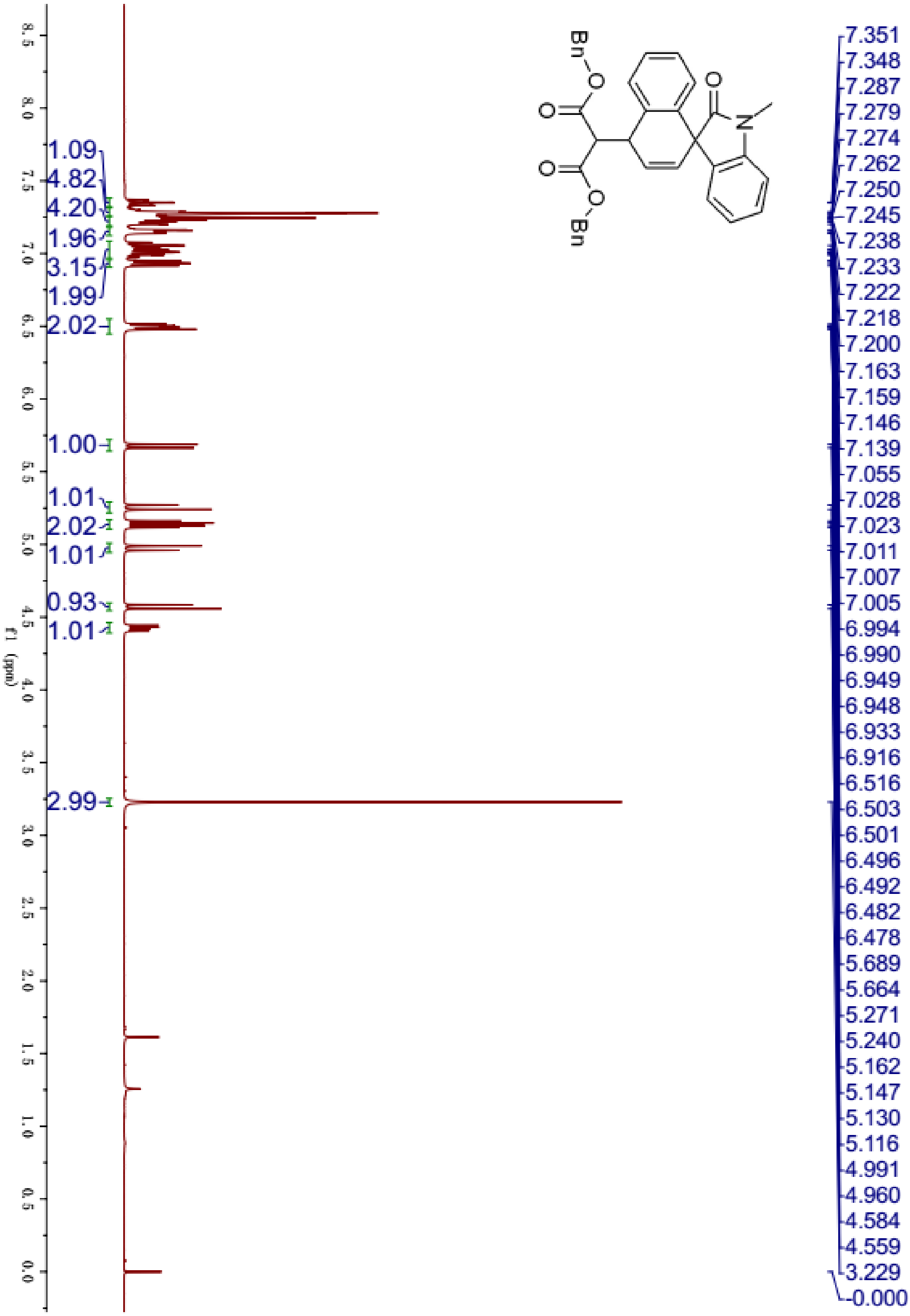
Bn
O
O
O
O
Bn
O
N
f1 (ppm)
1.09
4.82
4.20
1.96
3.15
1.99
2.02
1.00
1.01
2.02
1.01
0.93
1.01
2.99
7.351
7.348
7.287
7.279
7.274
7.262
7.250
7.245
7.238
7.233
7.222
7.218
7.200
7.163
7.159
7.146
7.139
7.055
7.028
7.023
7.011
7.007
7.005
6.994
6.990
6.949
6.948
6.933
6.916
6.516
6.503
6.501
6.496
6.492
6.482
6.478
5.689
5.664
5.271
5.240
5.162
5.147
5.130
5.116
4.991
4.960
4.584
4.559
3.229
-0.000

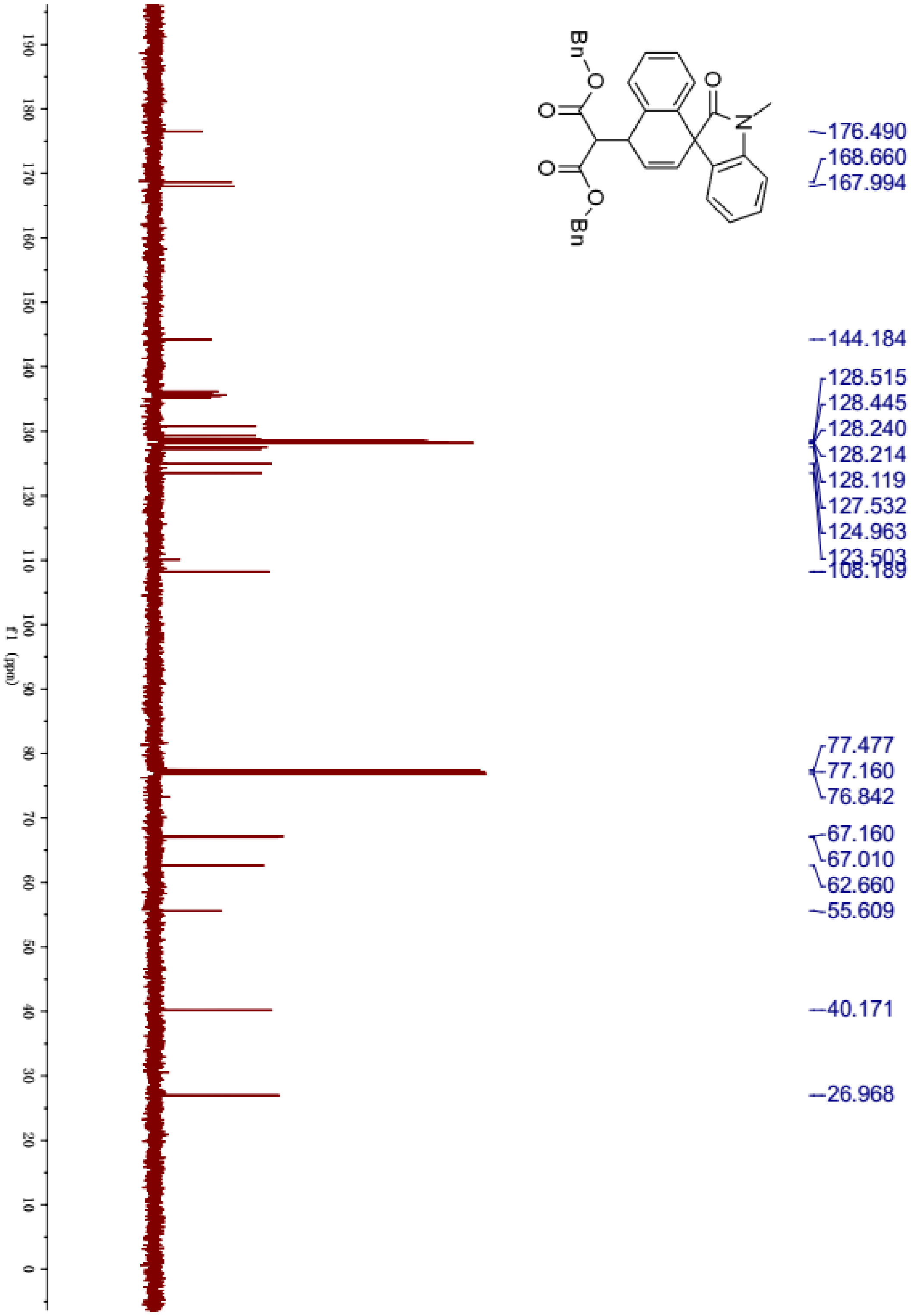
176.490
168.660
167.994
144.184
128.515
128.445
128.240
128.214
128.119
127.532
124.963
123.503
108.189
77.477
77.160
76.842
67.160
67.010
62.660
55.609
40.171
26.968

3s

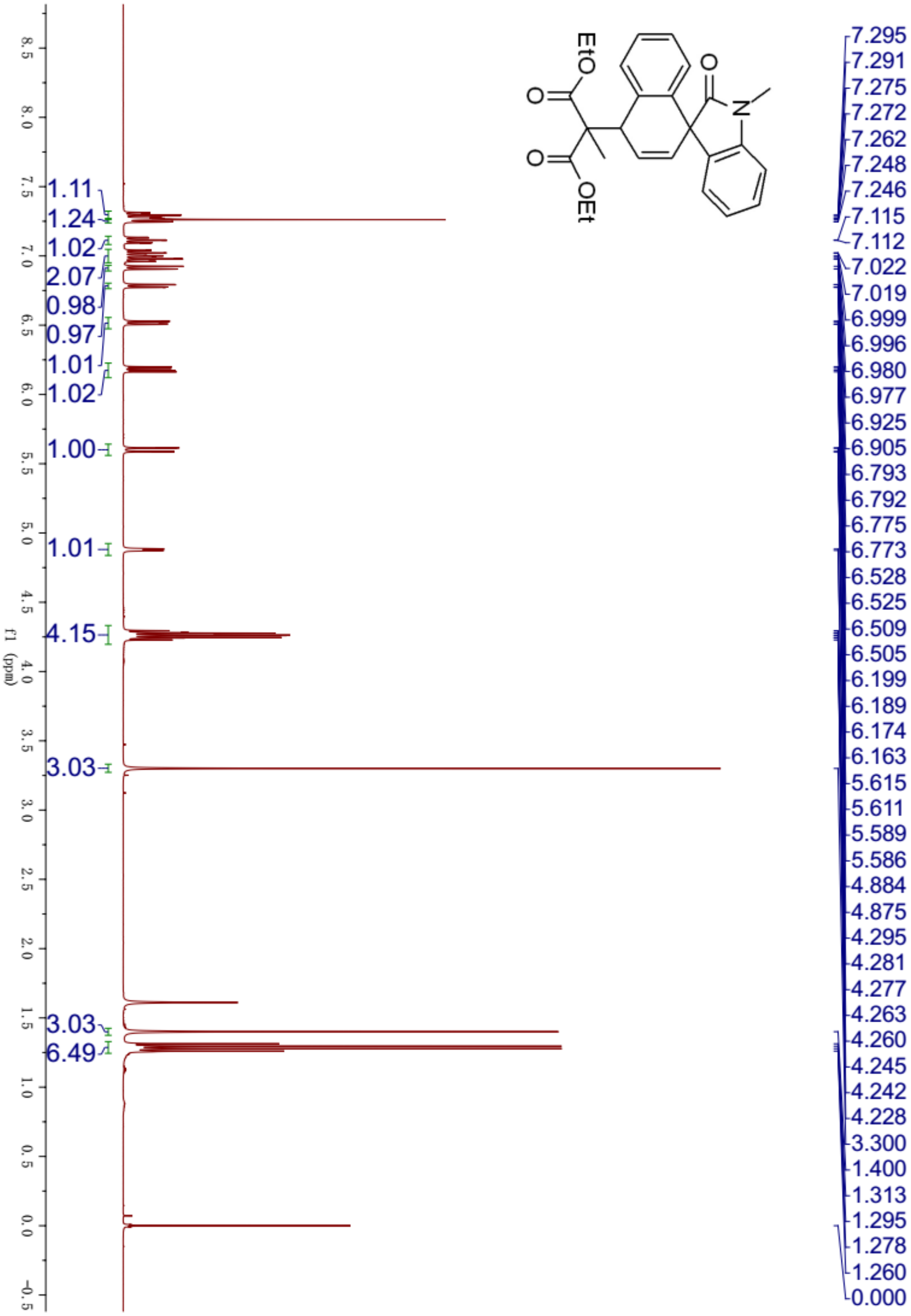

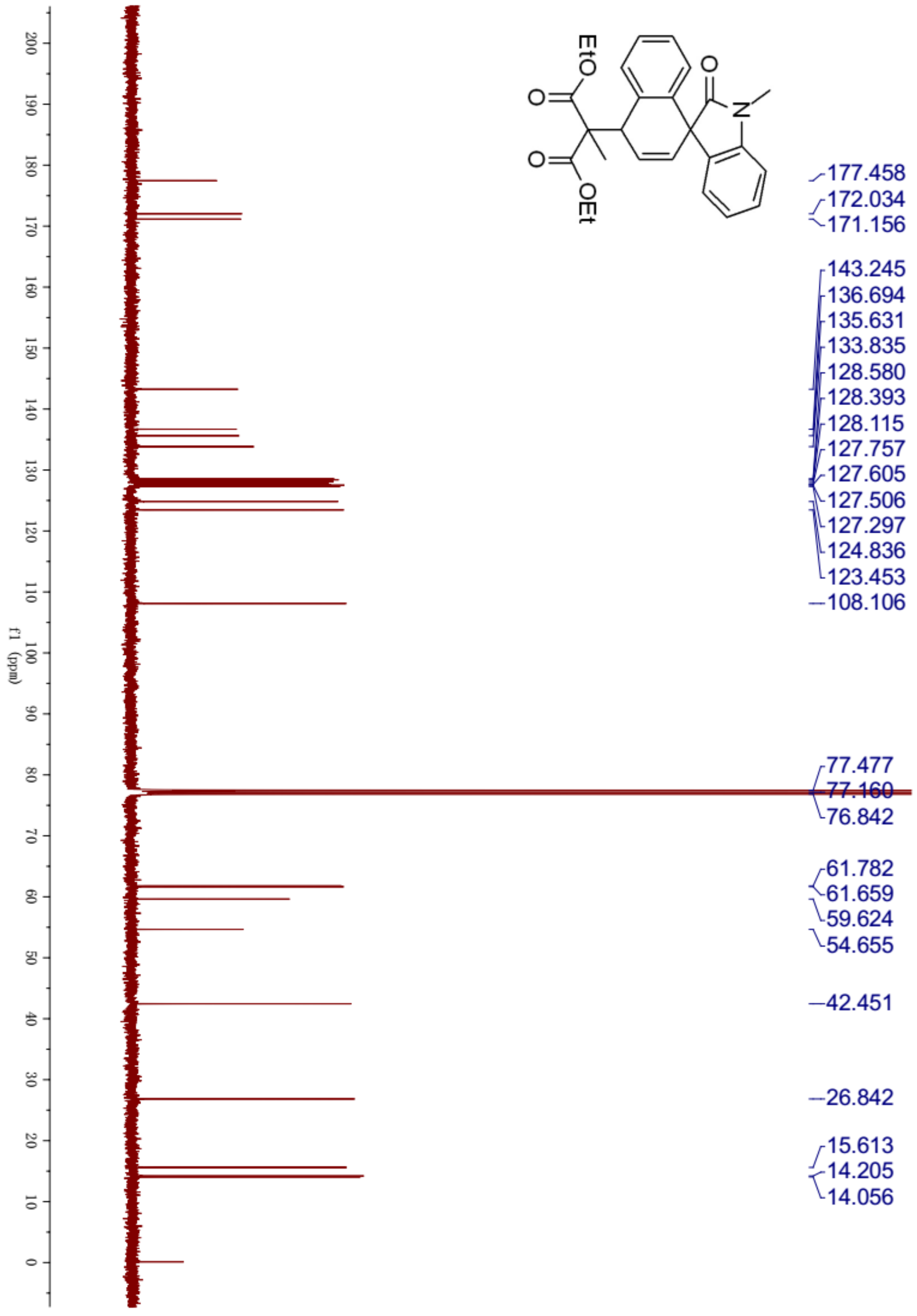

177.458
172.034
171.156
143.245
136.694
135.631
133.835
128.580
128.393
128.115
127.757
127.605
127.506
127.297
124.836
123.453
108.106
77.477
77.160
76.842
61.782
61.659
59.624
54.655
42.451
26.842
15.613
14.205
14.056
f1 (ppm)

4a

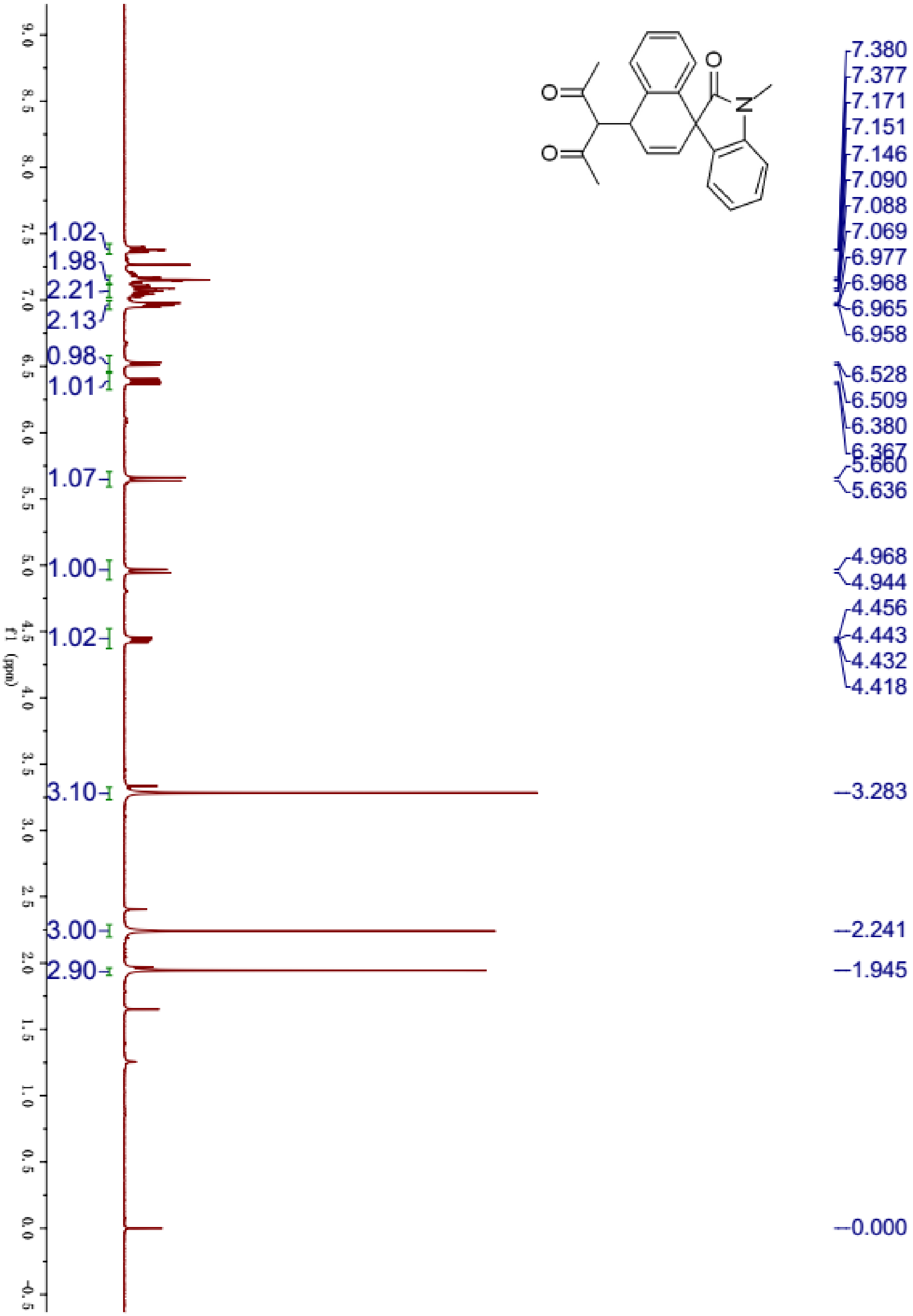
7.380
7.377
7.171
7.151
7.146
7.090
7.088
7.069
6.977
6.968
6.965
6.958
6.528
6.509
6.380
6.367
5.660
5.636
4.968
4.944
4.456
4.443
4.432
4.418
3.283
2.241
1.945
0.000
1.02
1.98
2.21
2.13
0.98
1.01
1.07
1.00
1.02
3.10
3.00
2.90
f1 (ppm)

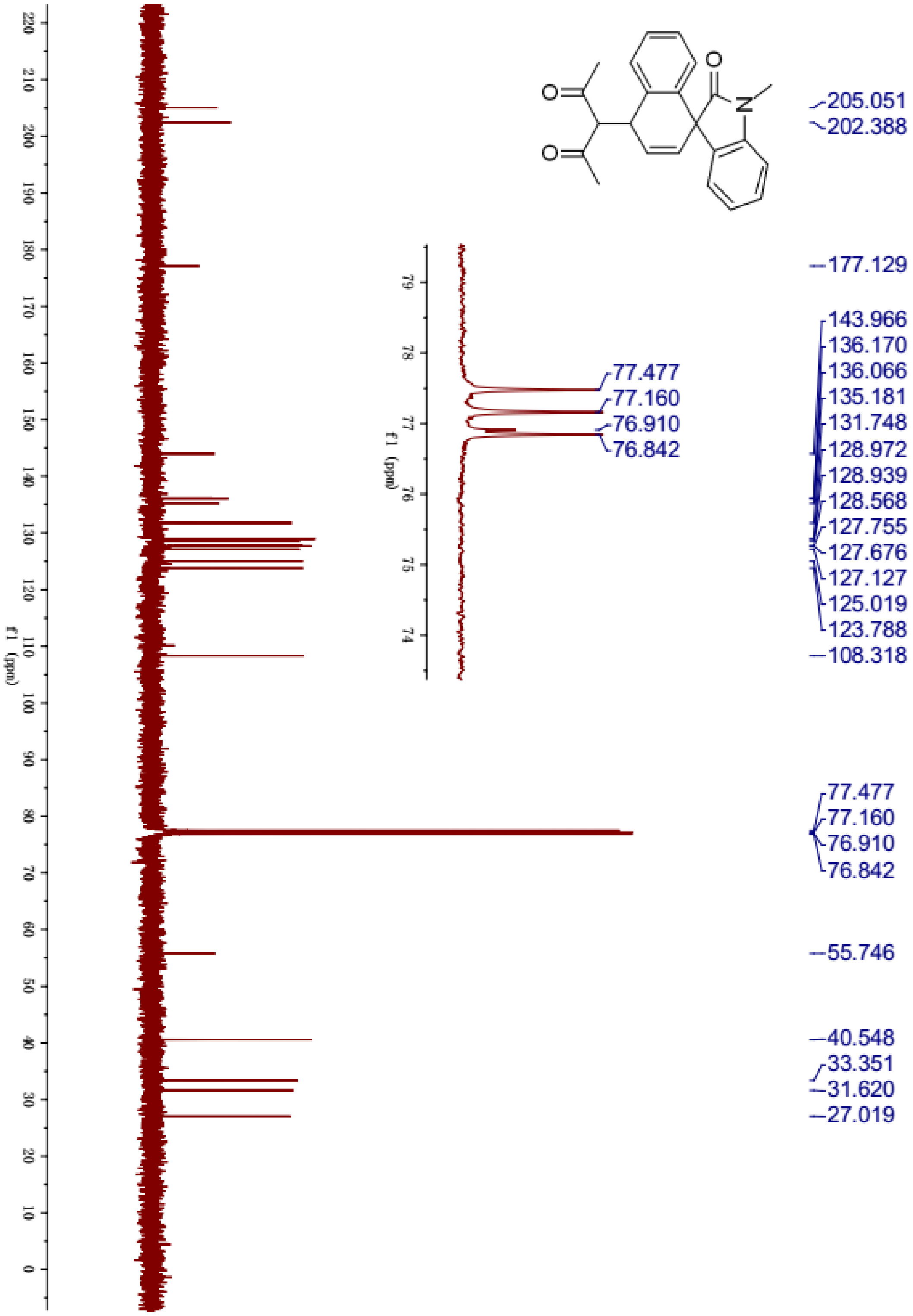
205.051
202.388
177.129
143.966
136.170
136.066
135.181
131.748
128.972
128.939
128.568
127.755
127.676
127.127
125.019
123.788
108.318
77.477
77.160
76.910
76.842
55.746
40.548
33.351
31.620
27.019
f1 (ppm)

4b

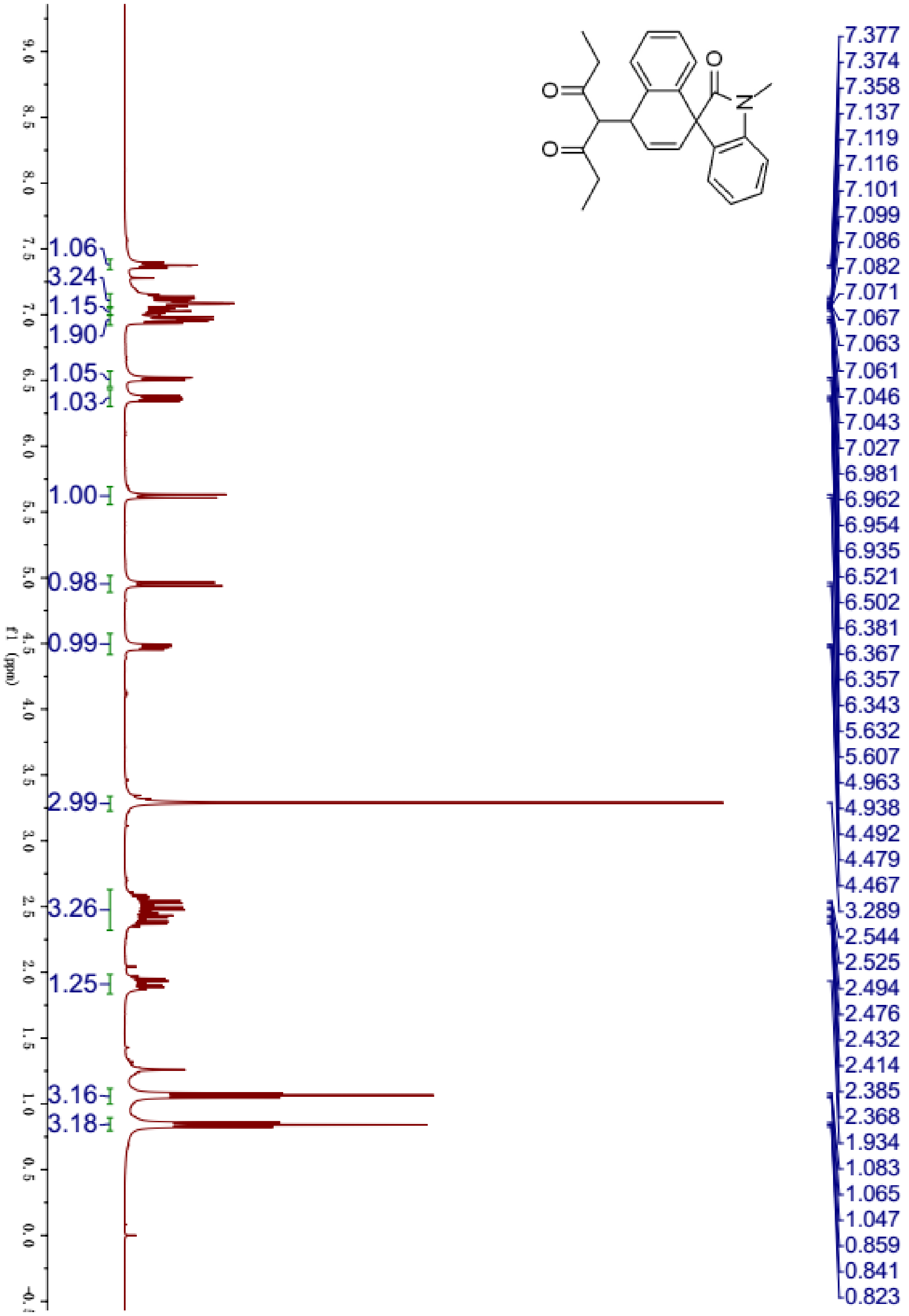
7.377
7.374
7.358
7.137
7.119
7.116
7.101
7.099
7.086
7.082
7.071
7.067
7.063
7.061
7.046
7.043
7.027
6.981
6.962
6.954
6.935
6.521
6.502
6.381
6.367
6.357
6.343
5.632
5.607
4.963
4.938
4.492
4.479
4.467
3.289
2.544
2.525
2.494
2.476
2.432
2.414
2.385
2.368
1.934
1.083
1.065
1.047
0.859
0.841
0.823
1.06
3.24
1.15
1.90
1.05
1.03
1.00
0.98
0.99
2.99
3.26
1.25
3.16
3.18
f1 (ppm)

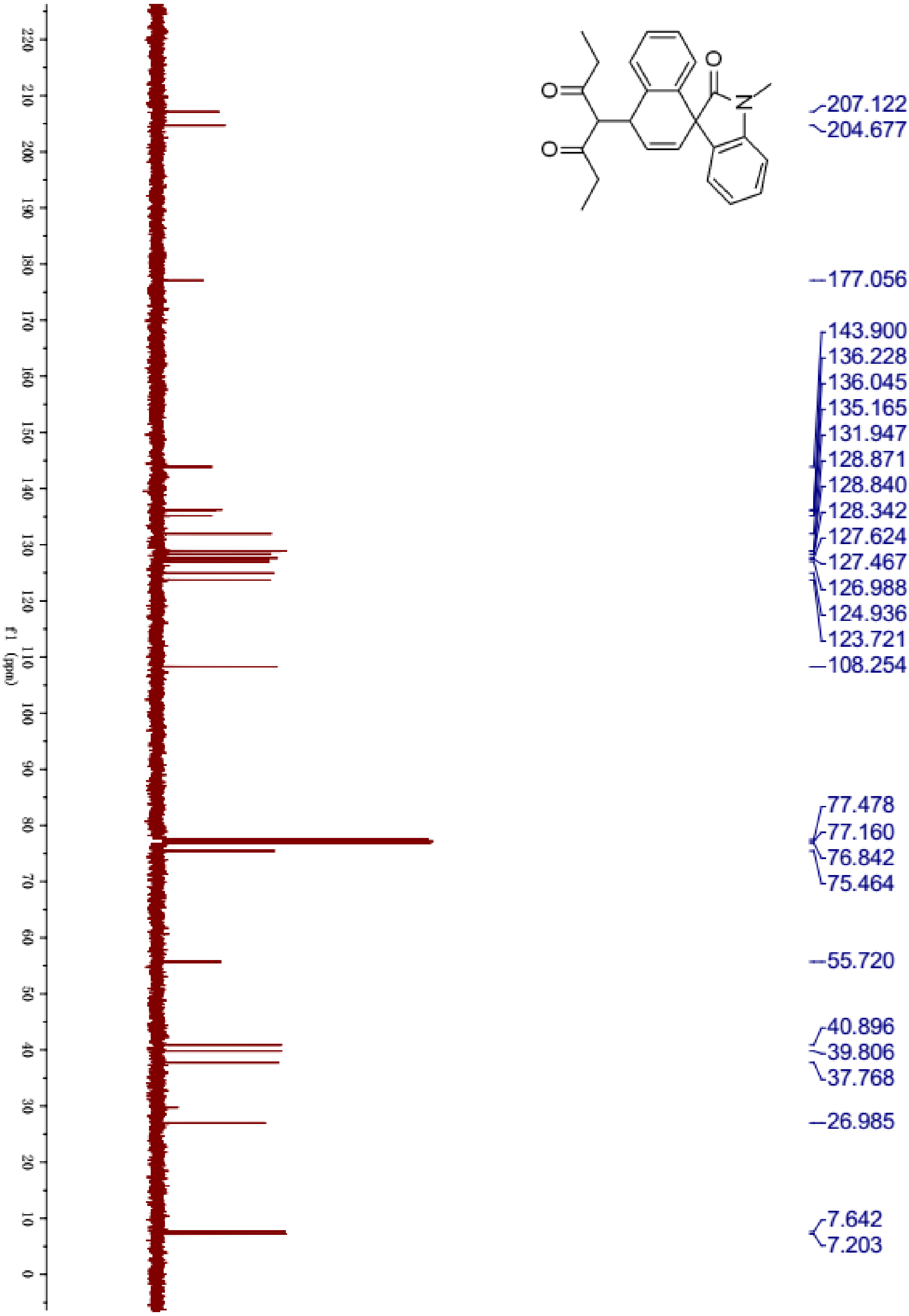
207.122
204.677
177.056
143.900
136.228
136.045
135.165
131.947
128.871
128.840
128.342
127.624
127.467
126.988
124.936
123.721
108.254
77.478
77.160
76.842
75.464
55.720
40.896
39.806
37.768
26.985
7.642
7.203
f1 (ppm)

4c

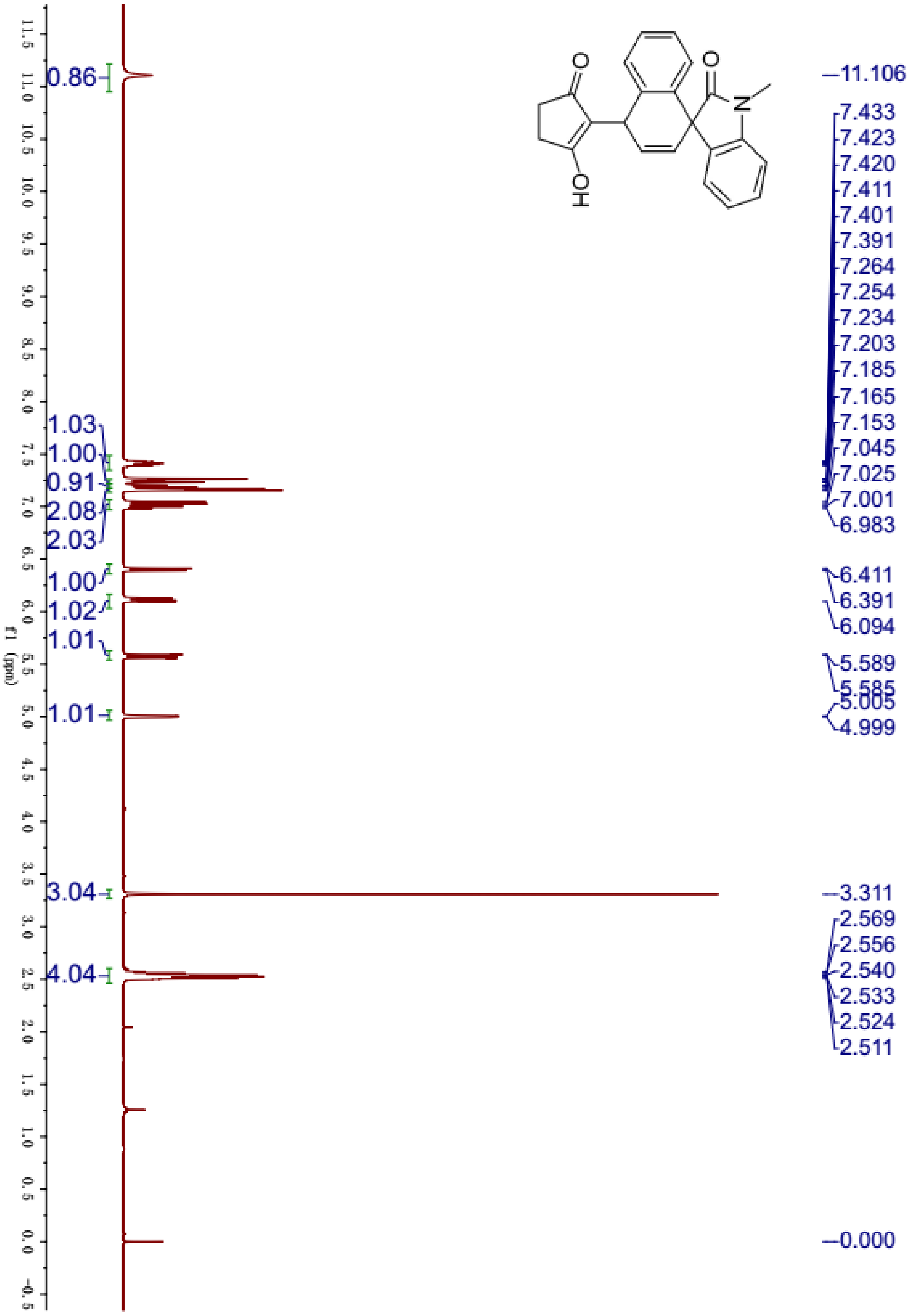

0.86
1.03
1.00
0.91
2.08
2.03
1.00
1.02
1.01
1.01
3.04
4.04
11.106
7.433
7.423
7.420
7.411
7.401
7.391
7.264
7.254
7.234
7.203
7.185
7.165
7.153
7.045
7.025
7.001
6.983
6.411
6.391
6.094
5.589
5.585
5.005
4.999
3.311
2.569
2.556
2.540
2.533
2.524
2.511
0.000
f1 (ppm)

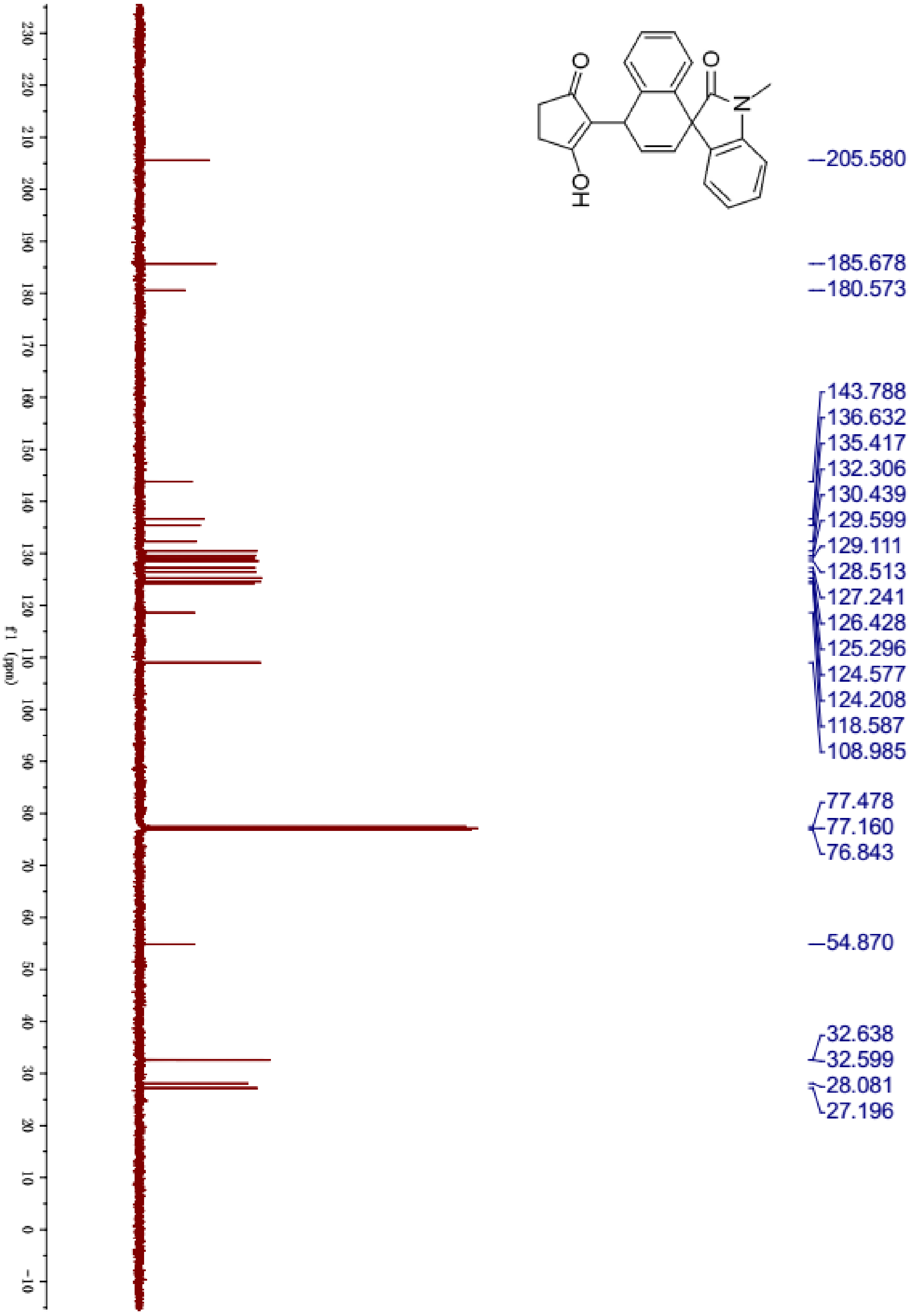
O
O
N
OH
205.580
185.678
180.573
143.788
136.632
135.417
132.306
130.439
129.599
129.111
128.513
127.241
126.428
125.296
124.577
124.208
118.587
108.985
77.478
77.160
76.843
54.870
32.638
32.599
28.081
27.196
f1 (ppm)

4d

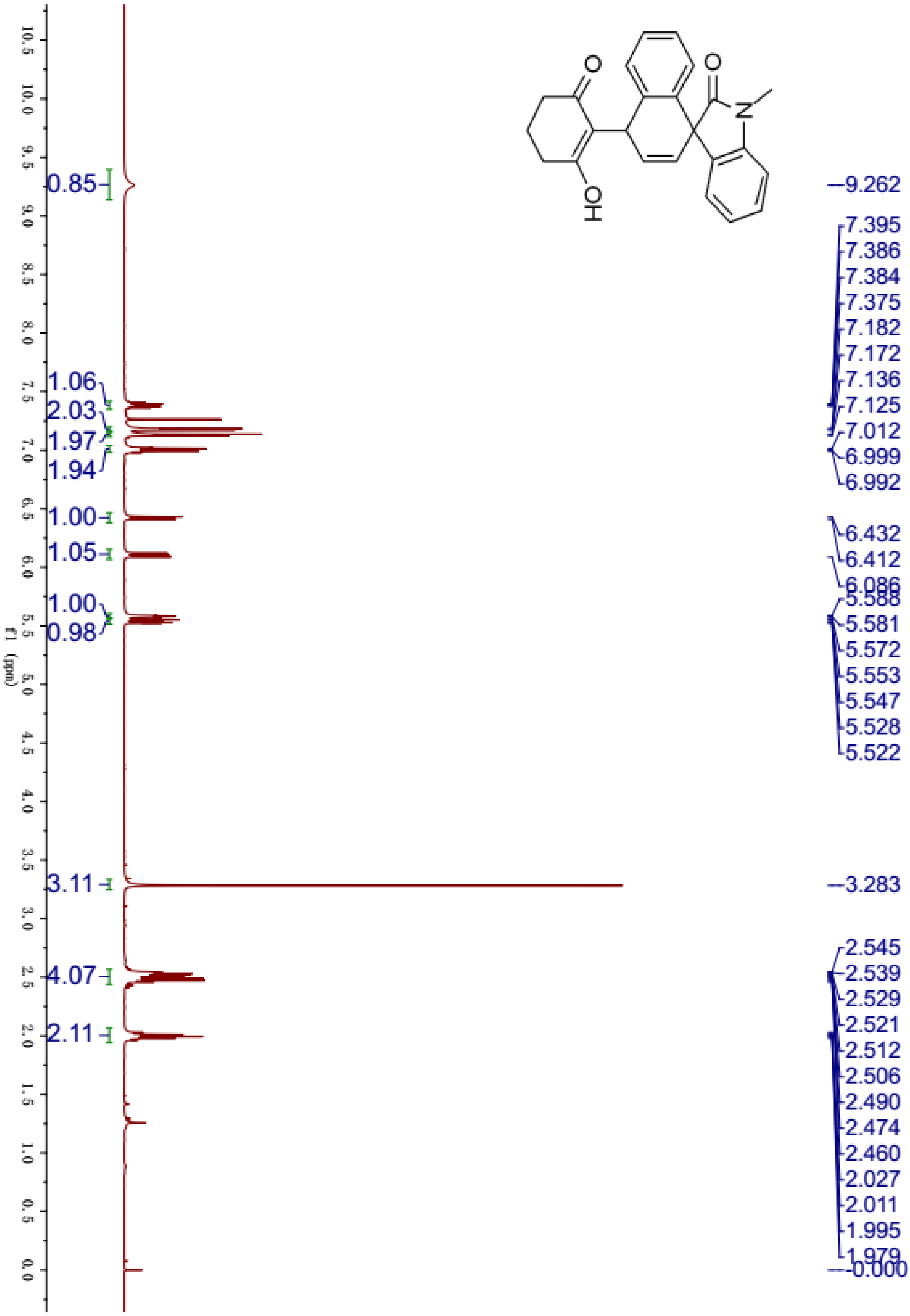

OH
O
O
N
9.262
7.395
7.386
7.384
7.375
7.182
7.172
7.136
7.125
7.012
6.999
6.992
6.432
6.412
6.086
5.588
5.581
5.572
5.553
5.547
5.528
5.522
3.283
2.545
2.539
2.529
2.521
2.512
2.506
2.490
2.474
2.460
2.027
2.011
1.995
1.979
0.000
0.85
1.06
2.03
1.97
1.94
1.00
1.05
1.00
0.98
3.11
4.07
2.11
f1 (ppm)

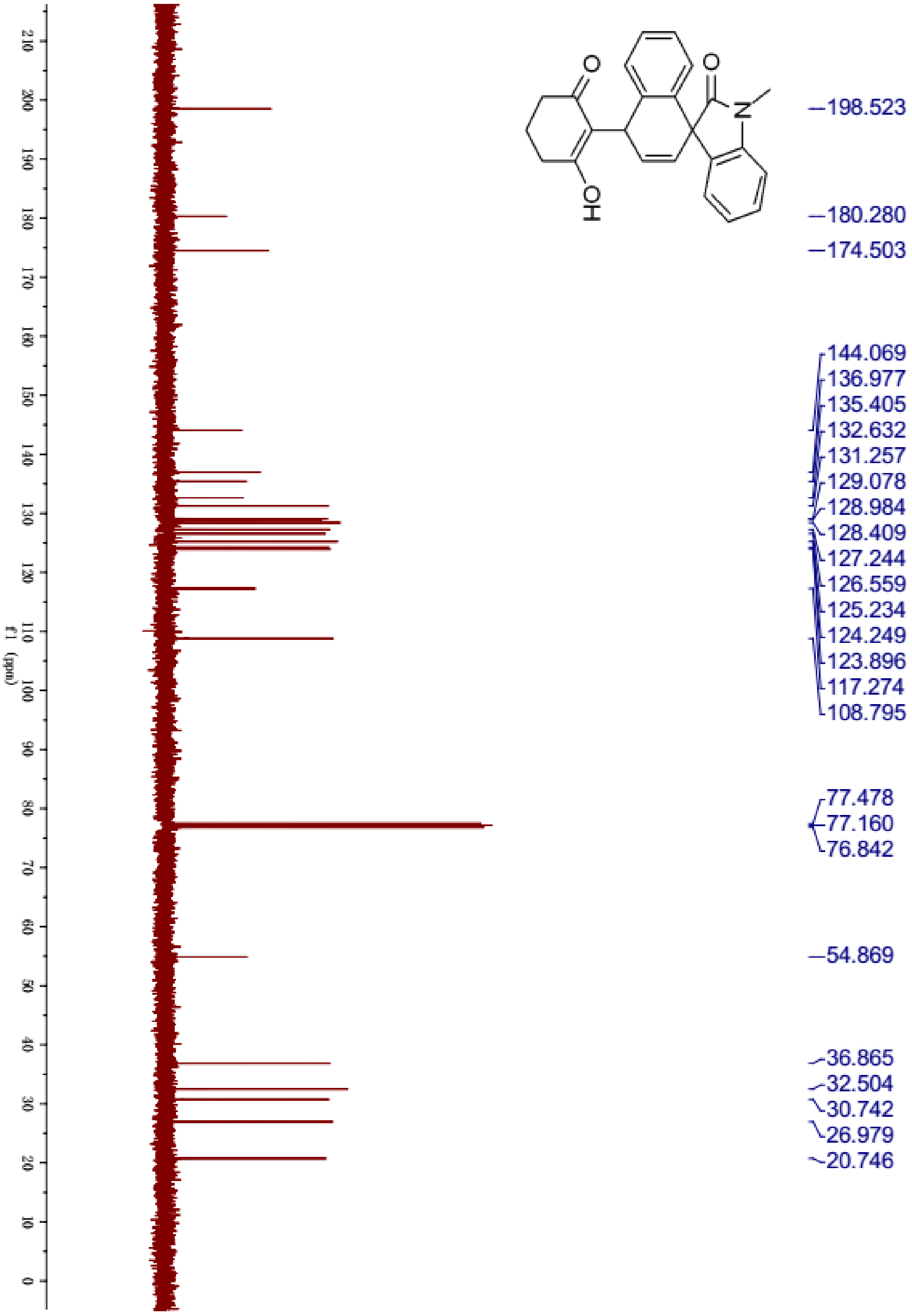
198.523
180.280
174.503
144.069
136.977
135.405
132.632
131.257
129.078
128.984
128.409
127.244
126.559
125.234
124.249
123.896
117.274
108.795
77.478
77.160
76.842
54.869
36.865
32.504
30.742
26.979
20.746
f1 (ppm)

5a

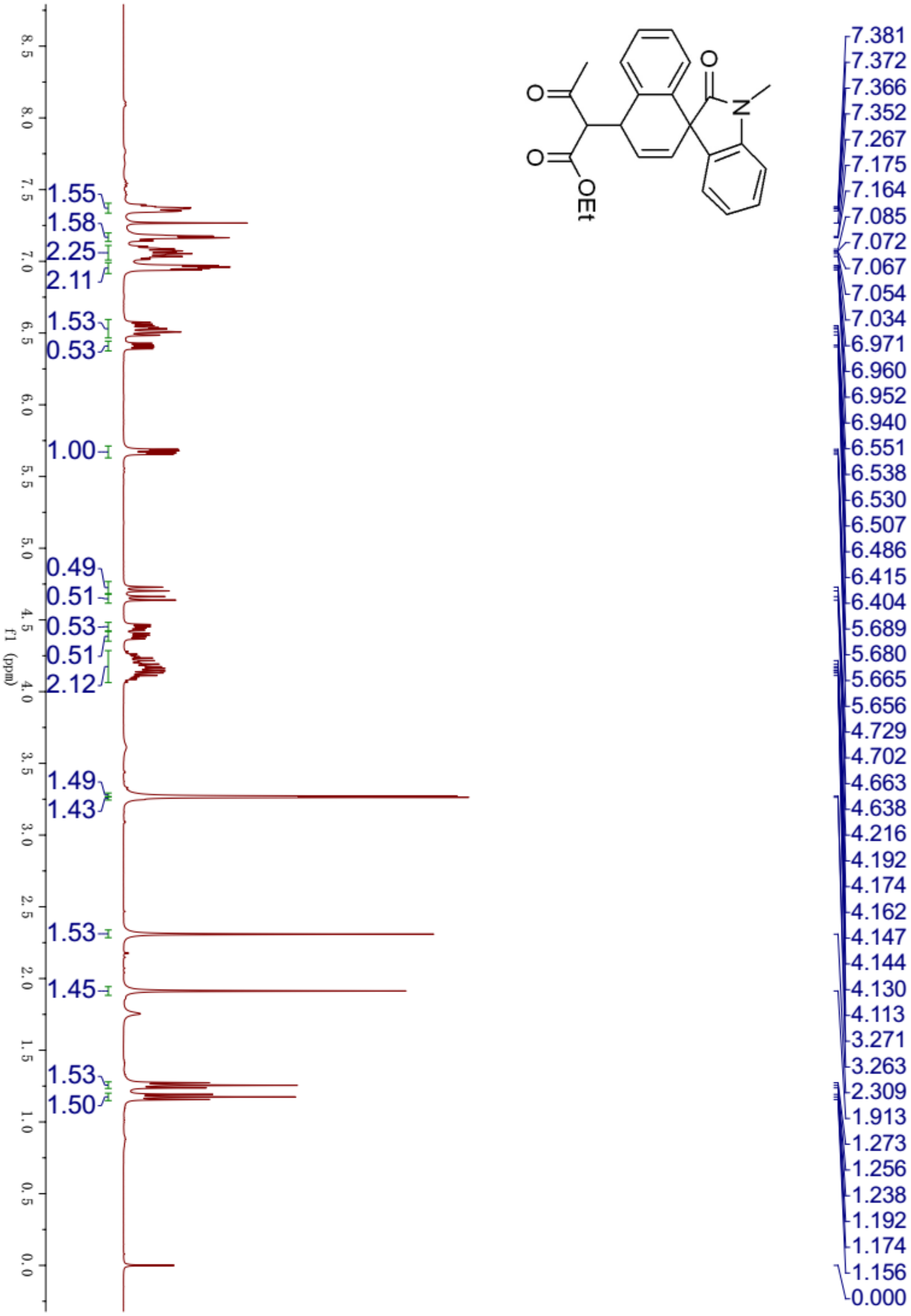
7.381
7.372
7.366
7.352
7.267
7.175
7.164
7.085
7.072
7.067
7.054
7.034
6.971
6.960
6.952
6.940
6.551
6.538
6.530
6.507
6.486
6.415
6.404
5.689
5.680
5.665
5.656
4.729
4.702
4.663
4.638
4.216
4.192
4.174
4.162
4.147
4.144
4.130
4.113
3.271
3.263
2.309
1.913
1.273
1.256
1.238
1.192
1.174
1.156
0.000
1.55
1.58
2.25
2.11
1.53
0.53
1.00
0.49
0.51
0.53
0.51
2.12
1.49
1.43
1.53
1.45
1.53
1.50
f1 (ppm)
O
N
OEt

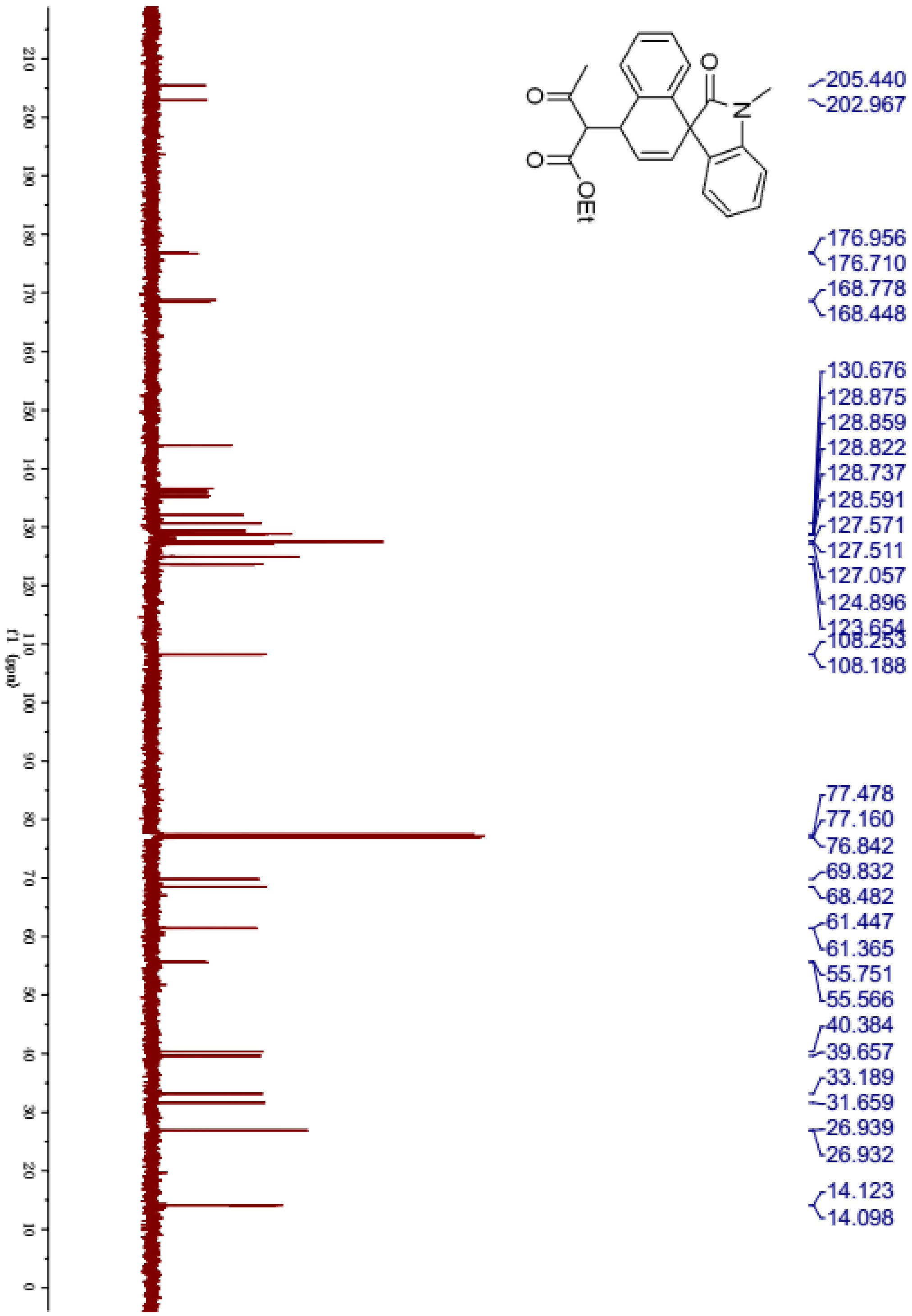

O
O
OEt
O
N
205.440
202.967
176.956
176.710
168.778
168.448
130.676
128.875
128.859
128.822
128.737
128.591
127.571
127.511
127.057
124.896
123.654
108.253
108.188
77.478
77.160
76.842
69.832
68.482
61.447
61.365
55.751
55.566
40.384
39.657
33.189
31.659
26.939
26.932
14.123
14.098
f1 (ppm)

5b

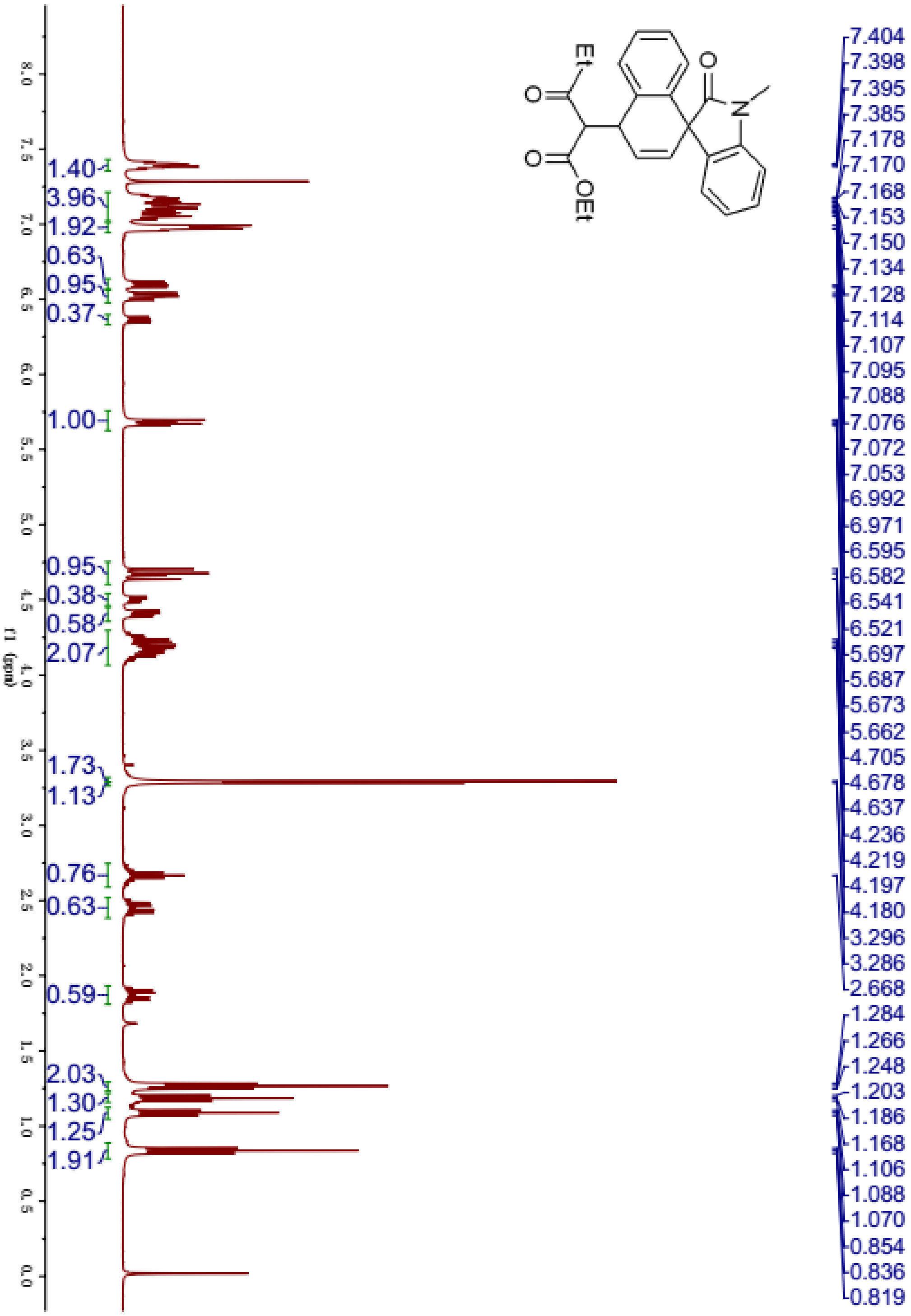
7.404
7.398
7.395
7.385
7.178
7.170
7.168
7.153
7.150
7.134
7.128
7.114
7.107
7.095
7.088
7.076
7.072
7.053
6.992
6.971
6.595
6.582
6.541
6.521
5.697
5.687
5.673
5.662
4.705
4.678
4.637
4.236
4.219
4.197
4.180
3.296
3.286
2.668
1.284
1.266
1.248
1.203
1.186
1.168
1.106
1.088
1.070
0.854
0.836
0.819
1.40
3.96
1.92
0.63
0.95
0.37
1.00
0.95
0.38
0.58
2.07
1.73
1.13
0.76
0.63
0.59
2.03
1.30
1.25
1.91
Et
O
OEt
N
f1 (ppm)

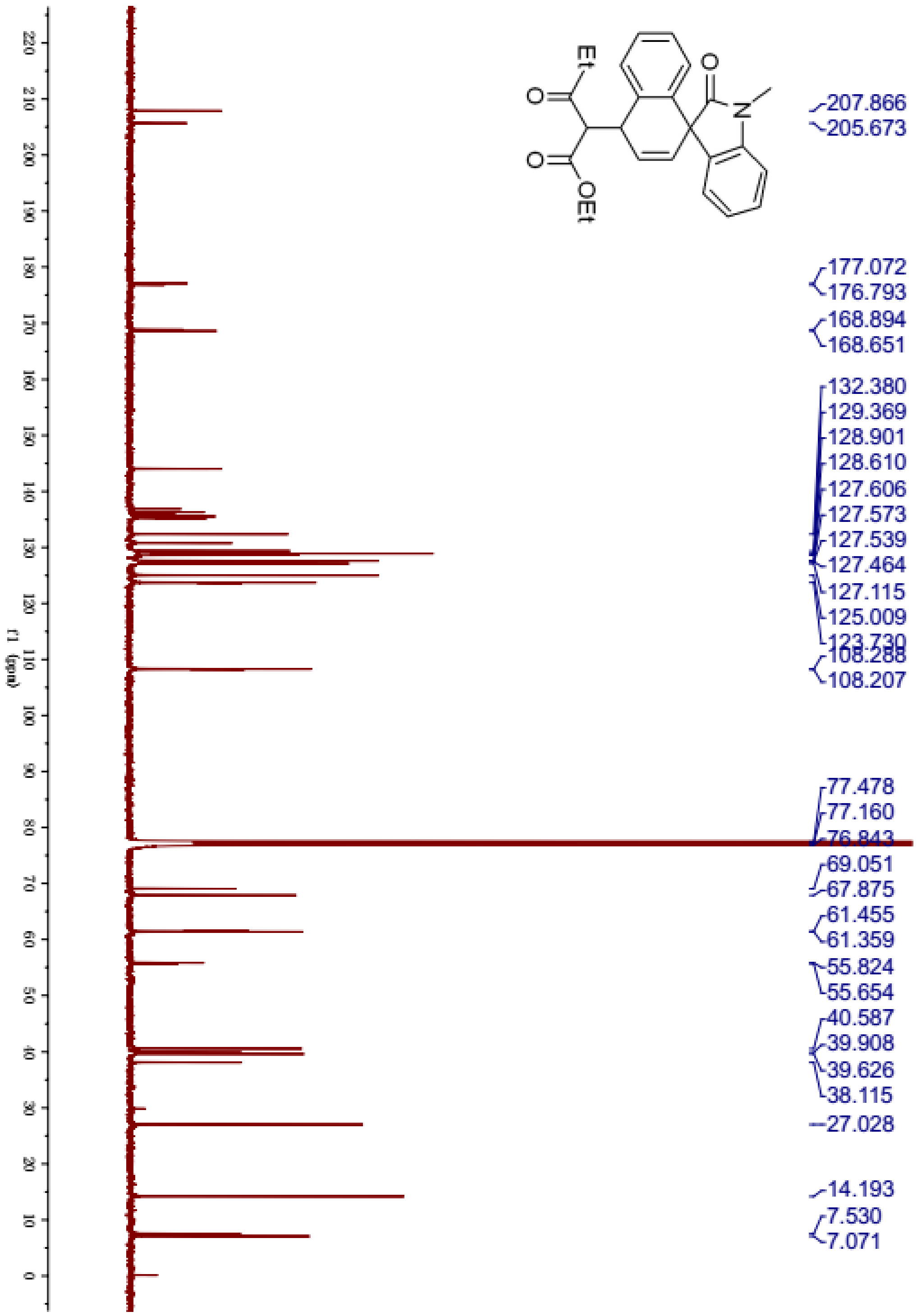
Et
O
O
OEt
O
N
207.866
205.673
177.072
176.793
168.894
168.651
132.380
129.369
128.901
128.610
127.606
127.573
127.539
127.464
127.115
125.009
123.730
108.288
108.207
77.478
77.160
76.843
69.051
67.875
61.455
61.359
55.824
55.654
40.587
39.908
39.626
38.115
27.028
14.193
7.530
7.071

5c

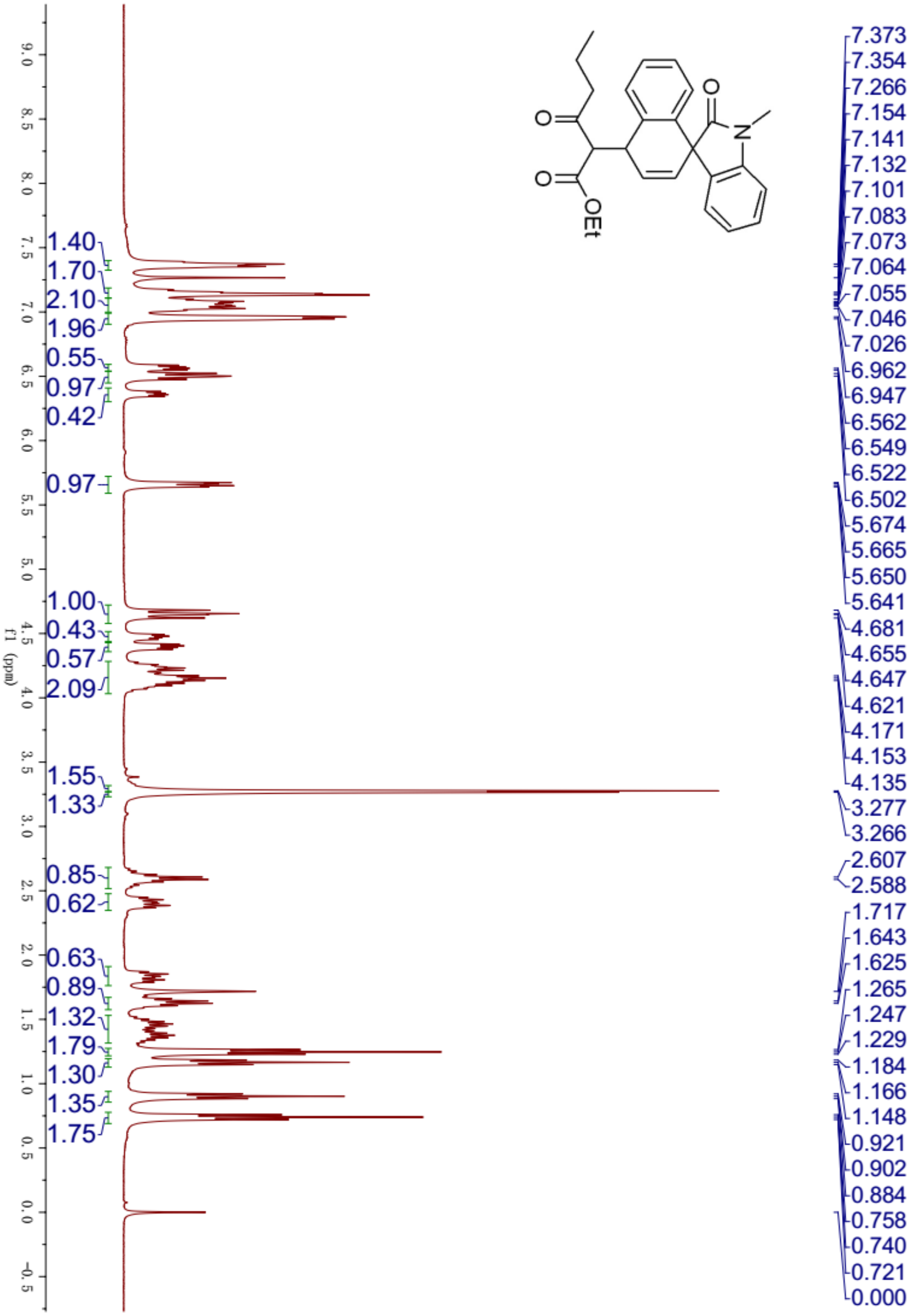
OEt
O
N
7.373
7.354
7.266
7.154
7.141
7.132
7.101
7.083
7.073
7.064
7.055
7.046
7.026
6.962
6.947
6.562
6.549
6.522
6.502
5.674
5.665
5.650
5.641
4.681
4.655
4.647
4.621
4.171
4.153
4.135
3.277
3.266
2.607
2.588
1.717
1.643
1.625
1.265
1.247
1.229
1.184
1.166
1.148
0.921
0.902
0.884
0.758
0.740
0.721
0.000
1.40
1.70
2.10
1.96
0.55
0.97
0.42
0.97
1.00
0.43
0.57
2.09
1.55
1.33
0.85
0.62
0.63
0.89
1.32
1.79
1.30
1.35
1.75
f1 (ppm)

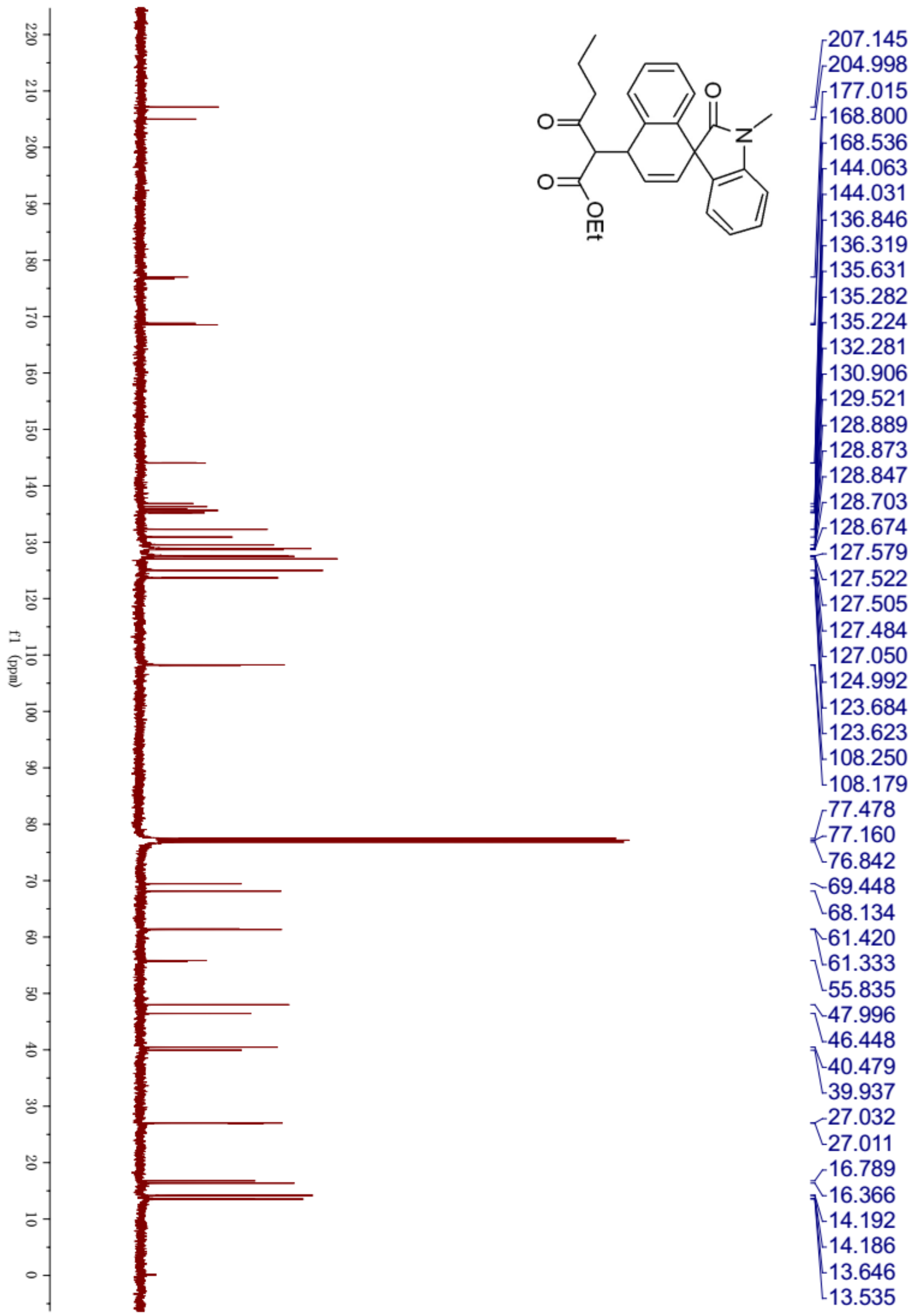
207.145
204.998
177.015
168.800
168.536
144.063
144.031
136.846
136.319
135.631
135.282
135.224
132.281
130.906
129.521
128.889
128.873
128.847
128.703
128.674
127.579
127.522
127.505
127.484
127.050
124.992
123.684
123.623
108.250
108.179
77.478
77.160
76.842
69.448
68.134
61.420
61.333
55.835
47.996
46.448
40.479
39.937
27.032
27.011
16.789
16.366
14.192
14.186
13.646
13.535
f1 (ppm)
OEt

5d

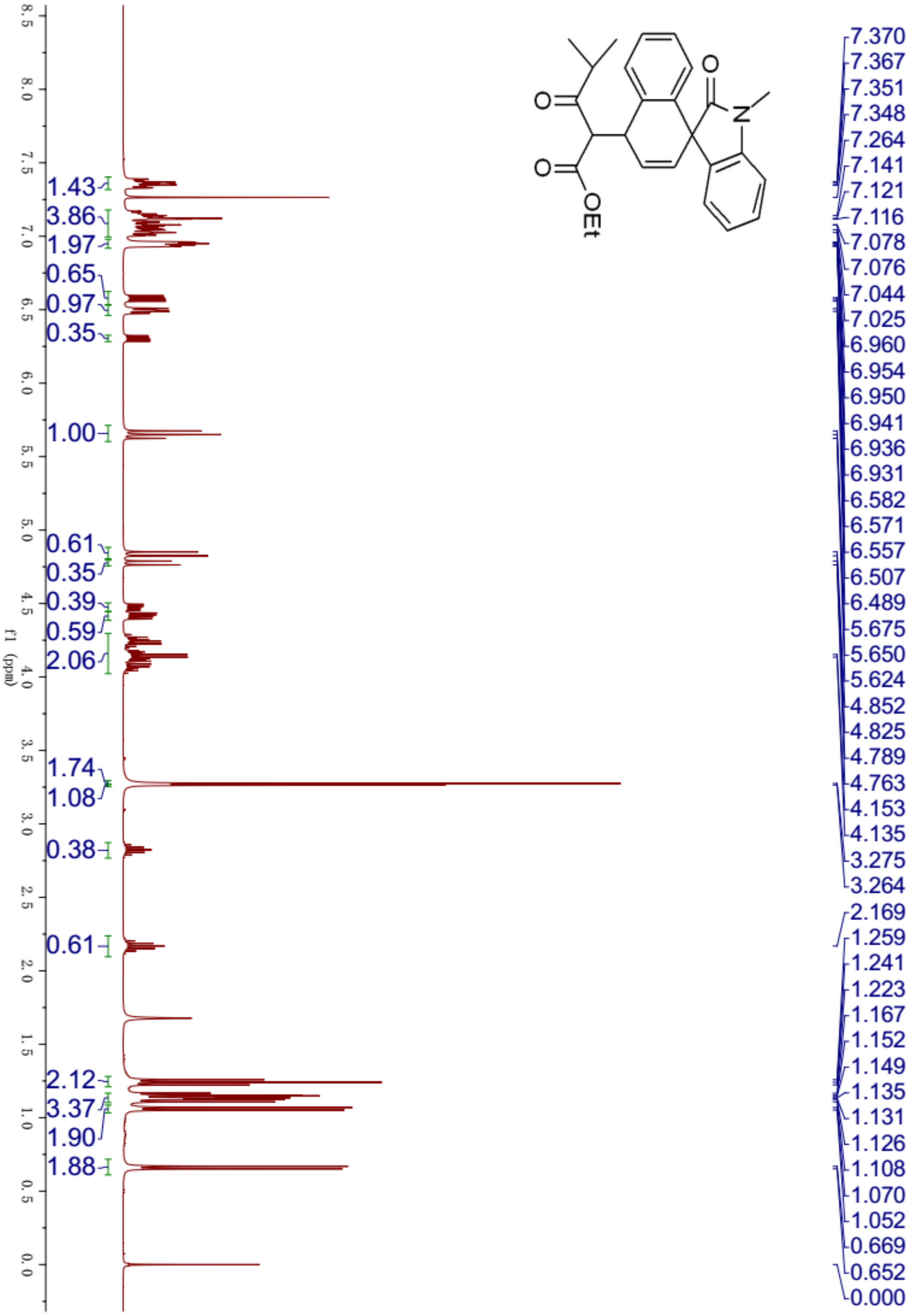

O
O
N
O
OEt
7.370
7.367
7.351
7.348
7.264
7.141
7.121
7.116
7.078
7.076
7.044
7.025
6.960
6.954
6.950
6.941
6.936
6.931
6.582
6.571
6.557
6.507
6.489
5.675
5.650
5.624
4.852
4.825
4.789
4.763
4.153
4.135
3.275
3.264
2.169
1.259
1.241
1.223
1.167
1.152
1.149
1.135
1.131
1.126
1.108
1.070
1.052
0.669
0.652
0.000
1.43
3.86
1.97
0.65
0.97
0.35
1.00
0.61
0.35
0.39
0.59
2.06
1.74
1.08
0.38
0.61
2.12
3.37
1.90
1.88

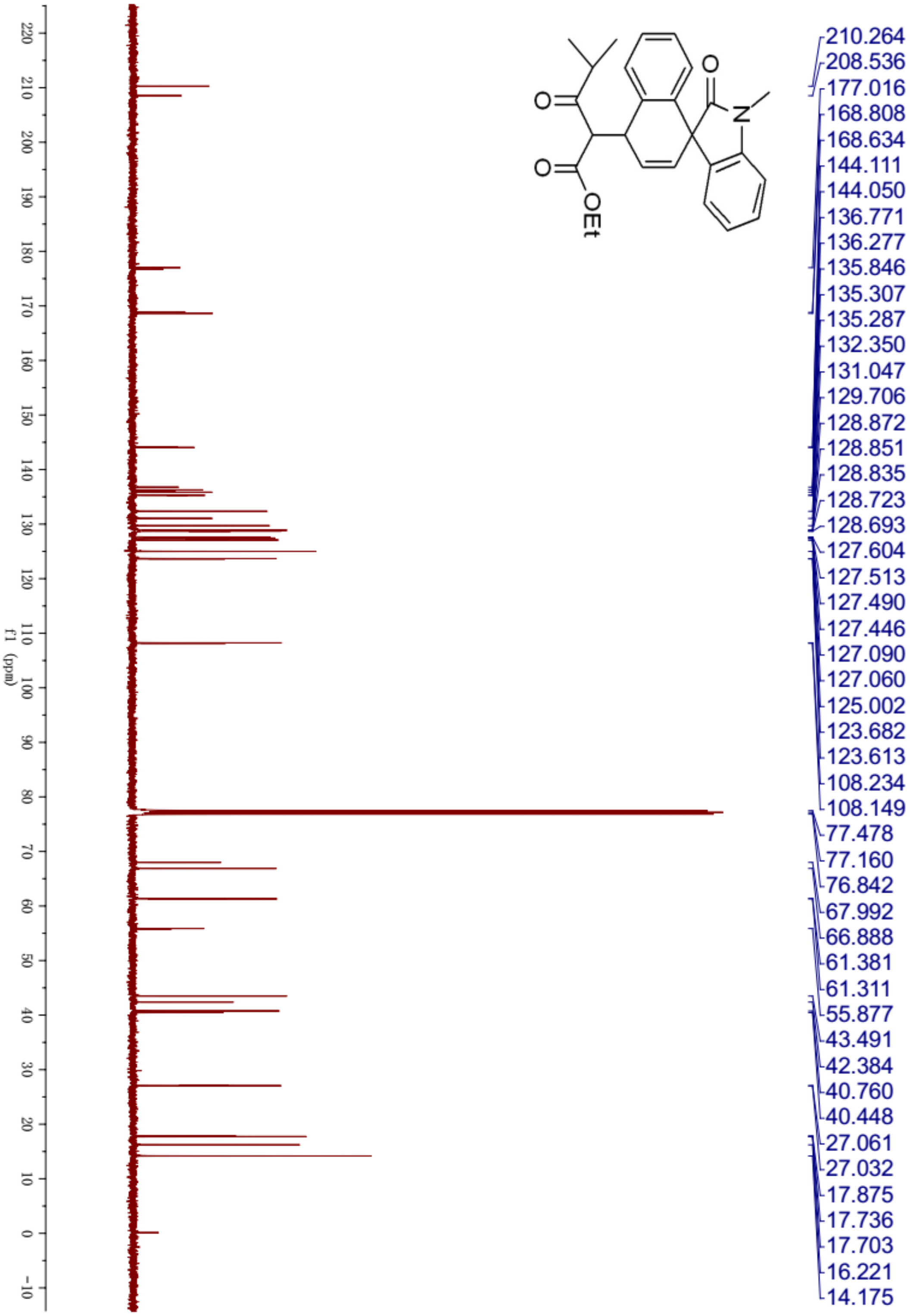
210.264
208.536
177.016
168.808
168.634
144.111
144.050
136.771
136.277
135.846
135.307
135.287
132.350
131.047
129.706
128.872
128.851
128.835
128.723
128.693
127.604
127.513
127.490
127.446
127.090
127.060
125.002
123.682
123.613
108.234
108.149
77.478
77.160
76.842
67.992
66.888
61.381
61.311
55.877
43.491
42.384
40.760
40.448
27.061
27.032
17.875
17.736
17.703
16.221
14.175
O
O
OEt
O
N
f1 (ppm)

5e

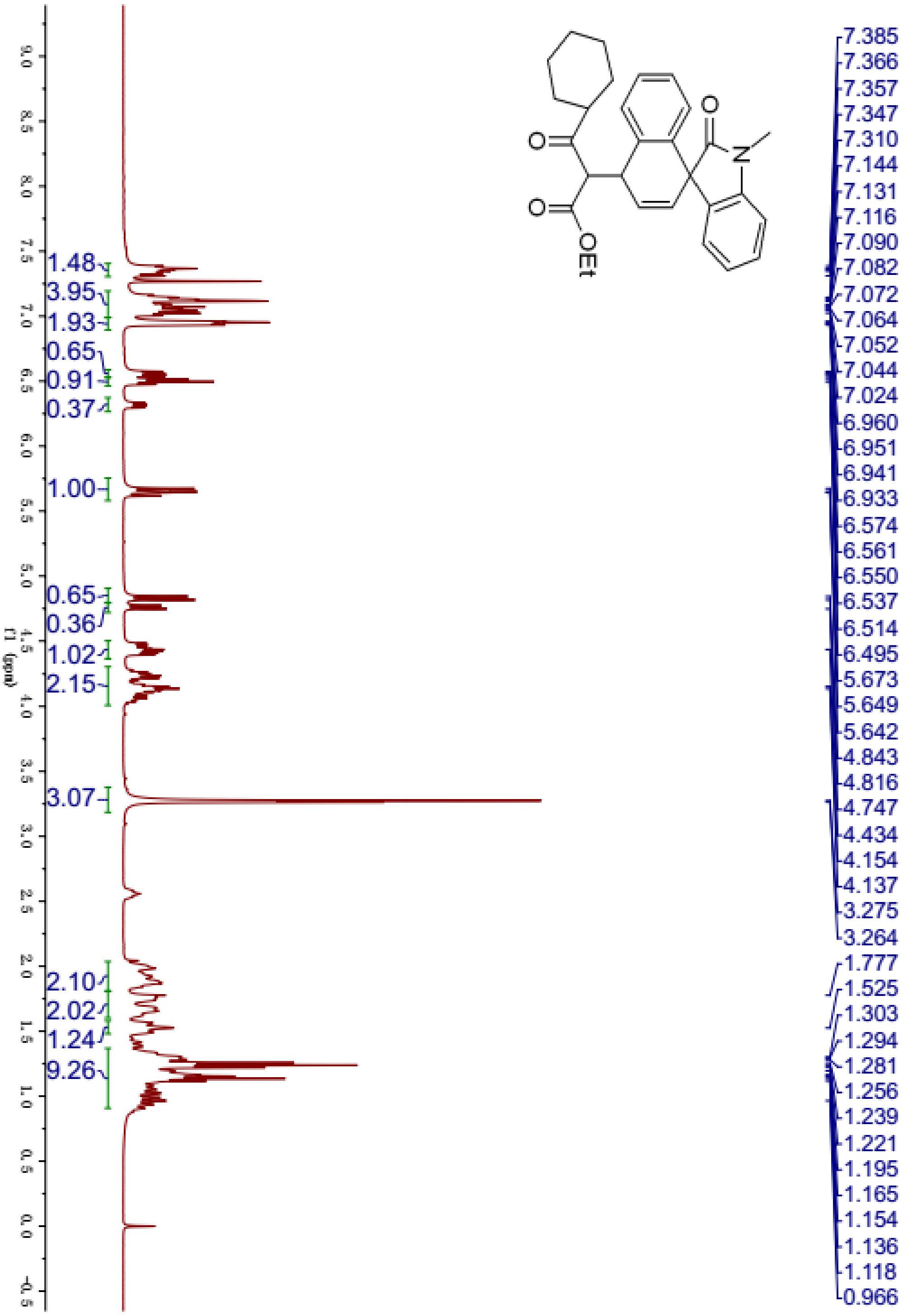
7.385
7.366
7.357
7.347
7.310
7.144
7.131
7.116
7.090
7.082
7.072
7.064
7.052
7.044
7.024
6.960
6.951
6.941
6.933
6.574
6.561
6.550
6.537
6.514
6.495
5.673
5.649
5.642
4.843
4.816
4.747
4.434
4.154
4.137
3.275
3.264
1.777
1.525
1.303
1.294
1.281
1.256
1.239
1.221
1.195
1.165
1.154
1.136
1.118
0.966
1.48
3.95
1.93
0.65
0.91
0.37
1.00
0.65
0.36
1.02
2.15
3.07
2.10
2.02
1.24
9.26
f1 (ppm)
O
O
OEt
O
N

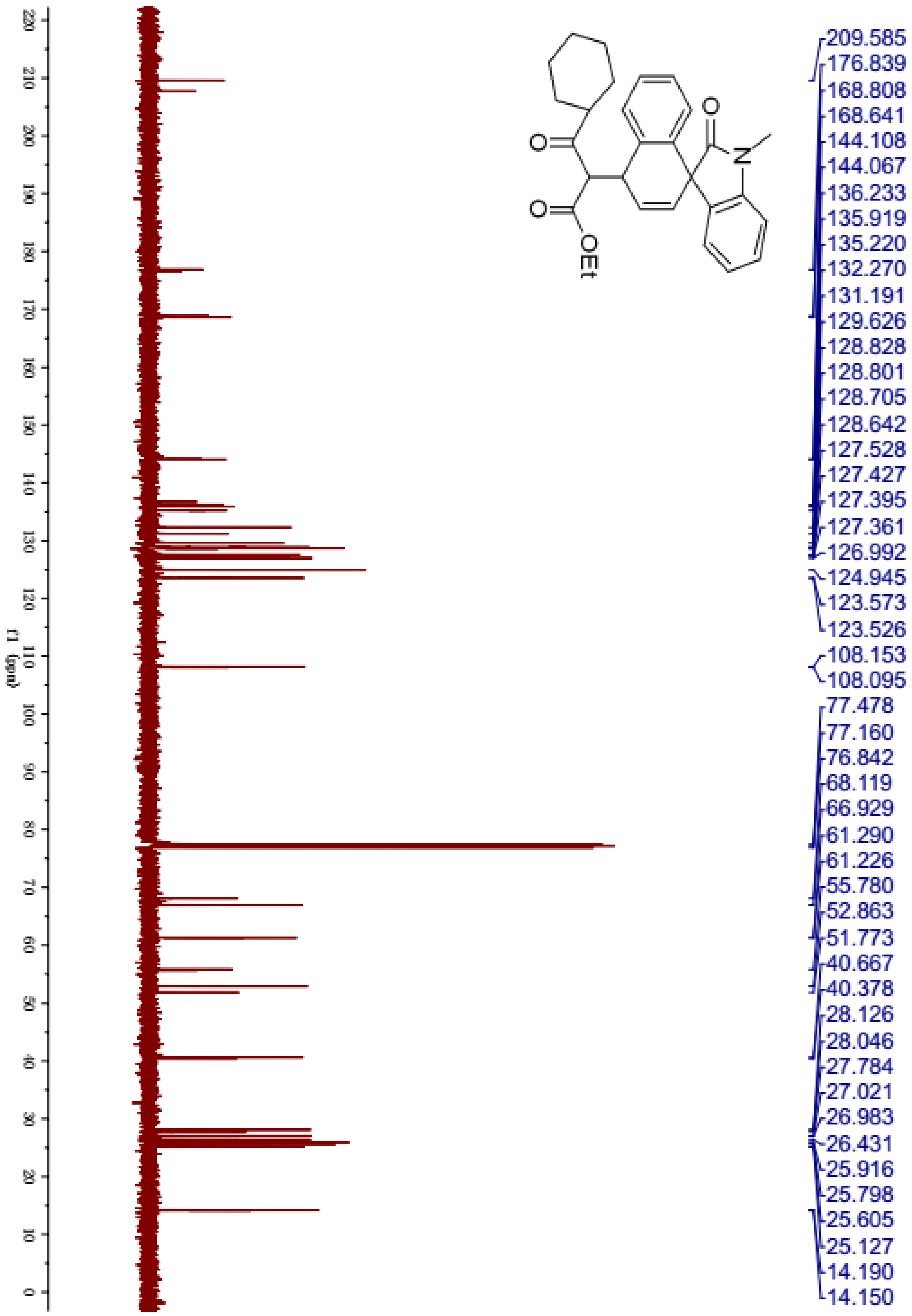
209.585
176.839
168.808
168.641
144.108
144.067
136.233
135.919
135.220
132.270
131.191
129.626
128.828
128.801
128.705
128.642
127.528
127.427
127.395
127.361
126.992
124.945
123.573
123.526
108.153
108.095
77.478
77.160
76.842
68.119
66.929
61.290
61.226
55.780
52.863
51.773
40.667
40.378
28.126
28.046
27.784
27.021
26.983
26.431
25.916
25.798
25.605
25.127
14.190
14.150
O
O
OEt
O
N
f1 (ppm)

5f

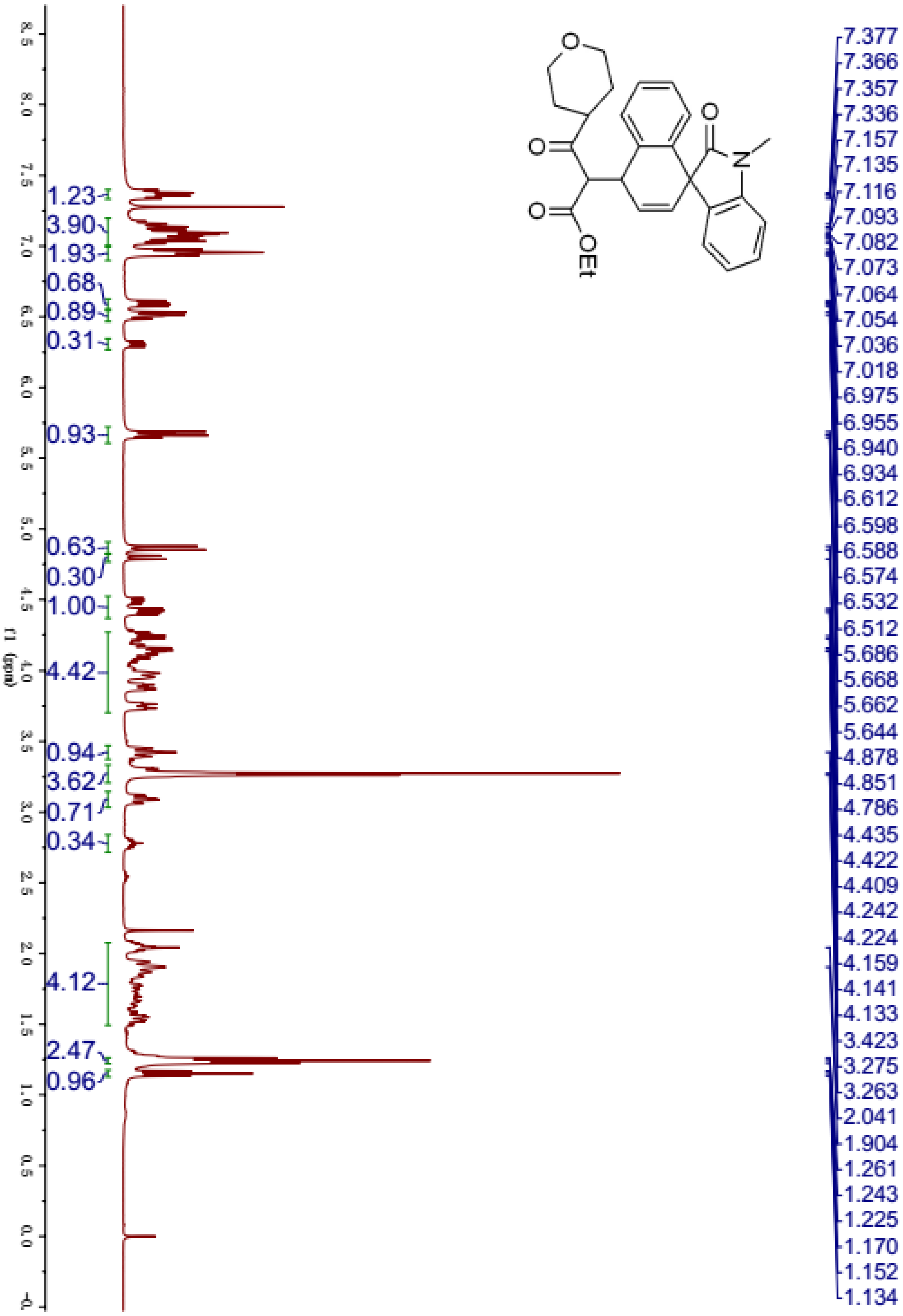

7.377
7.366
7.357
7.336
7.157
7.135
7.116
7.093
7.082
7.073
7.064
7.054
7.036
7.018
6.975
6.955
6.940
6.934
6.612
6.598
6.588
6.574
6.532
6.512
5.686
5.668
5.662
5.644
4.878
4.851
4.786
4.435
4.422
4.409
4.242
4.224
4.159
4.141
4.133
3.423
3.275
3.263
2.041
1.904
1.261
1.243
1.225
1.170
1.152
1.134
1.23
3.90
1.93
0.68
0.89
0.31
0.93
0.63
0.30
1.00
4.42
0.94
3.62
0.71
0.34
4.12
2.47
0.96
O
N
OEt
f1 (ppm)

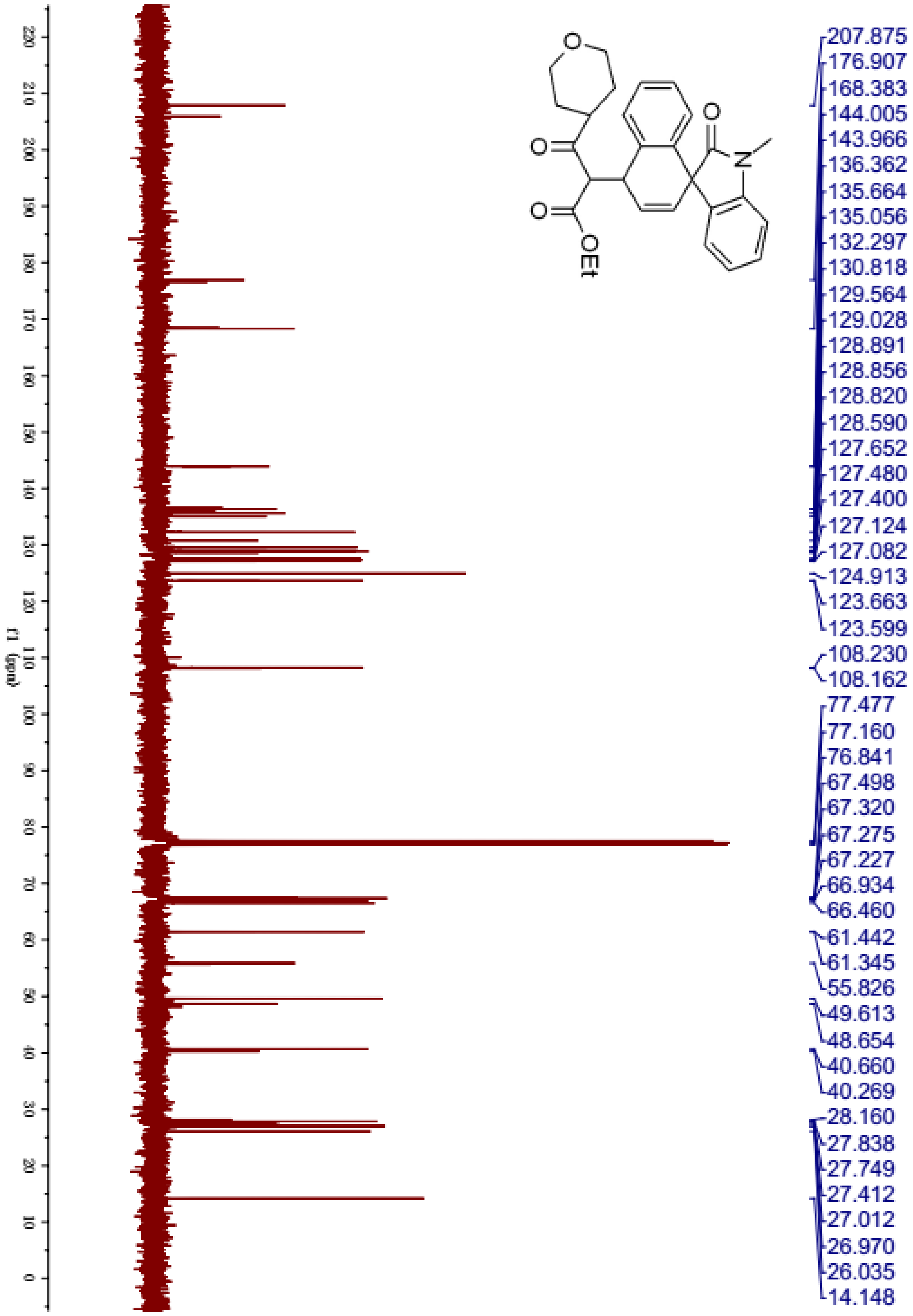
207.875
176.907
168.383
144.005
143.966
136.362
135.664
135.056
132.297
130.818
129.564
129.028
128.891
128.856
128.820
128.590
127.652
127.480
127.400
127.124
127.082
124.913
123.663
123.599
108.230
108.162
77.477
77.160
76.841
67.498
67.320
67.275
67.227
66.934
66.460
61.442
61.345
55.826
49.613
48.654
40.660
40.269
28.160
27.838
27.749
27.412
27.012
26.970
26.035
14.148
OEt
f1 (ppm)

5g

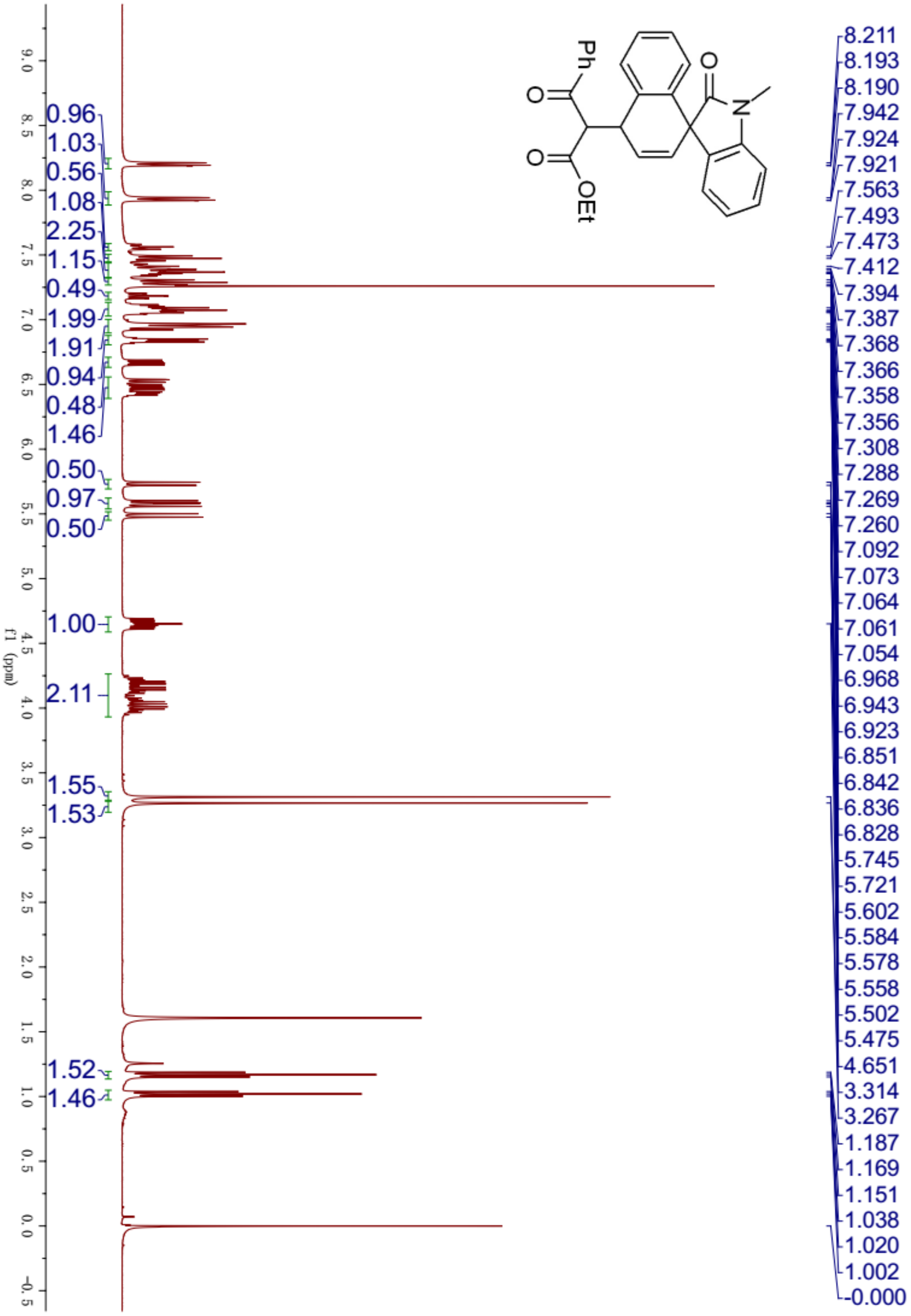

8.211
8.193
8.190
7.942
7.924
7.921
7.563
7.493
7.473
7.412
7.394
7.387
7.368
7.366
7.358
7.356
7.308
7.288
7.269
7.260
7.092
7.073
7.064
7.061
7.054
6.968
6.943
6.923
6.851
6.842
6.836
6.828
5.745
5.721
5.602
5.584
5.578
5.558
5.502
5.475
4.651
3.314
3.267
1.187
1.169
1.151
1.038
1.020
1.002
-0.000
0.96
1.03
0.56
1.08
2.25
1.15
0.49
1.99
1.91
0.94
0.48
1.46
0.50
0.97
0.50
1.00
2.11
1.55
1.53
1.52
1.46
f1 (ppm)
Ph
O
OEt
N

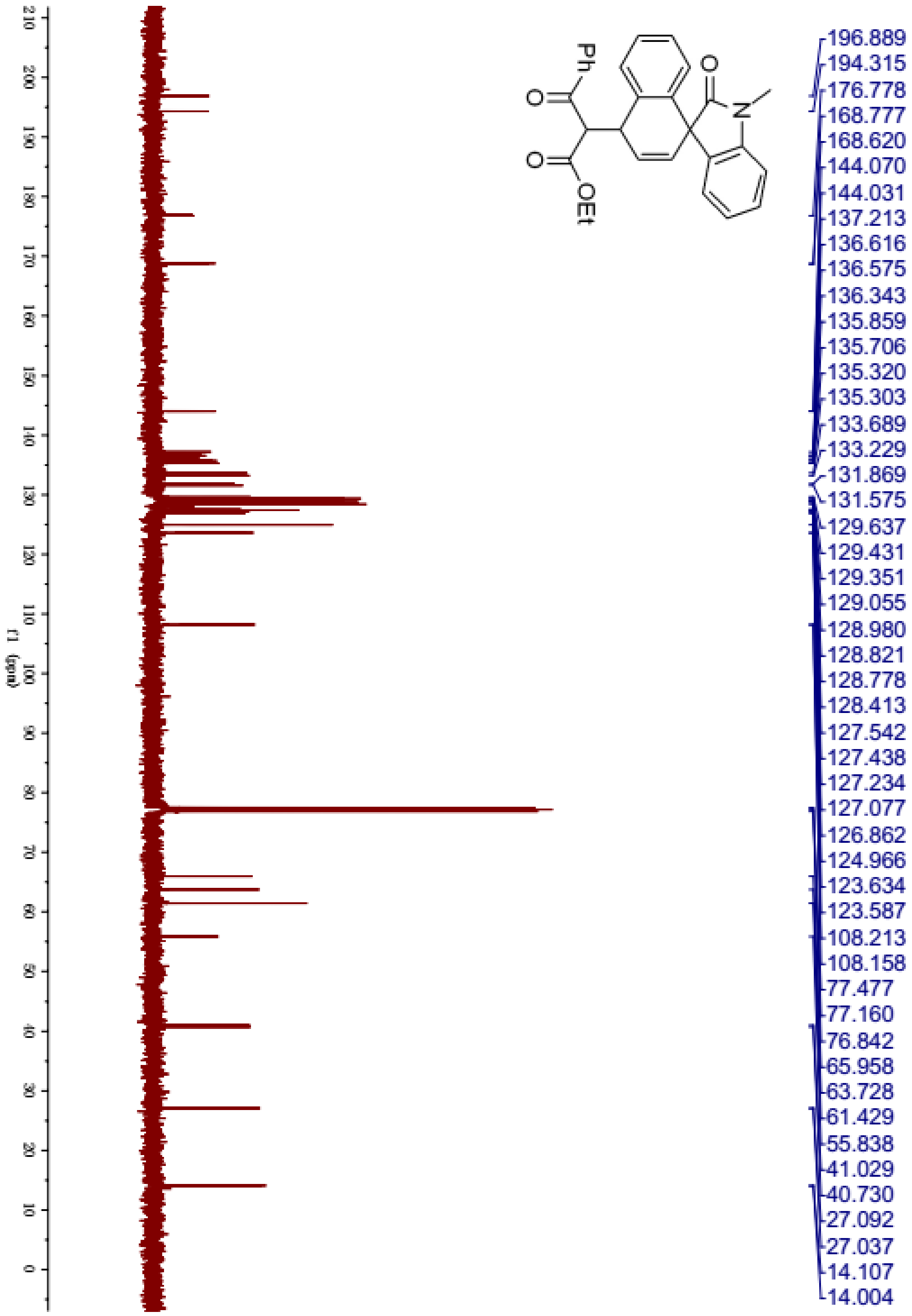
196.889
194.315
176.778
168.777
168.620
144.070
144.031
137.213
136.616
136.575
136.343
135.859
135.706
135.320
135.303
133.689
133.229
131.869
131.575
129.637
129.431
129.351
129.055
128.980
128.821
128.778
128.413
127.542
127.438
127.234
127.077
126.862
124.966
123.634
123.587
108.213
108.158
77.477
77.160
76.842
65.958
63.728
61.429
55.838
41.029
40.730
27.092
27.037
14.107
14.004
Ph
O
OEt
N
f1 (ppm)

5h

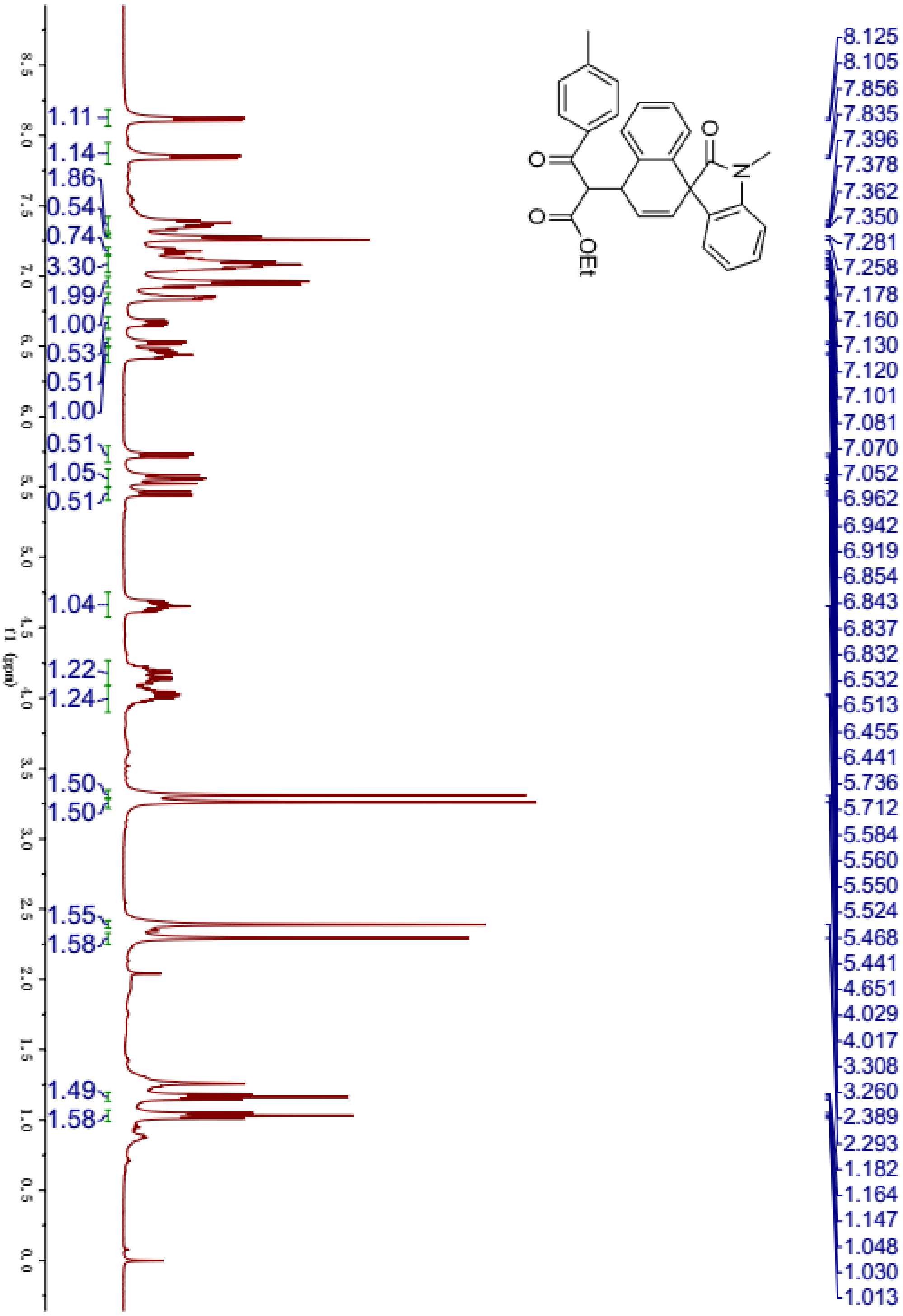
8.125
8.105
7.856
7.835
7.396
7.378
7.362
7.350
7.281
7.258
7.178
7.160
7.130
7.120
7.101
7.081
7.070
7.052
6.962
6.942
6.919
6.854
6.843
6.837
6.832
6.532
6.513
6.455
6.441
5.736
5.712
5.584
5.560
5.550
5.524
5.468
5.441
4.651
4.029
4.017
3.308
3.260
2.389
2.293
1.182
1.164
1.147
1.048
1.030
1.013
f1 (ppm)
OEt

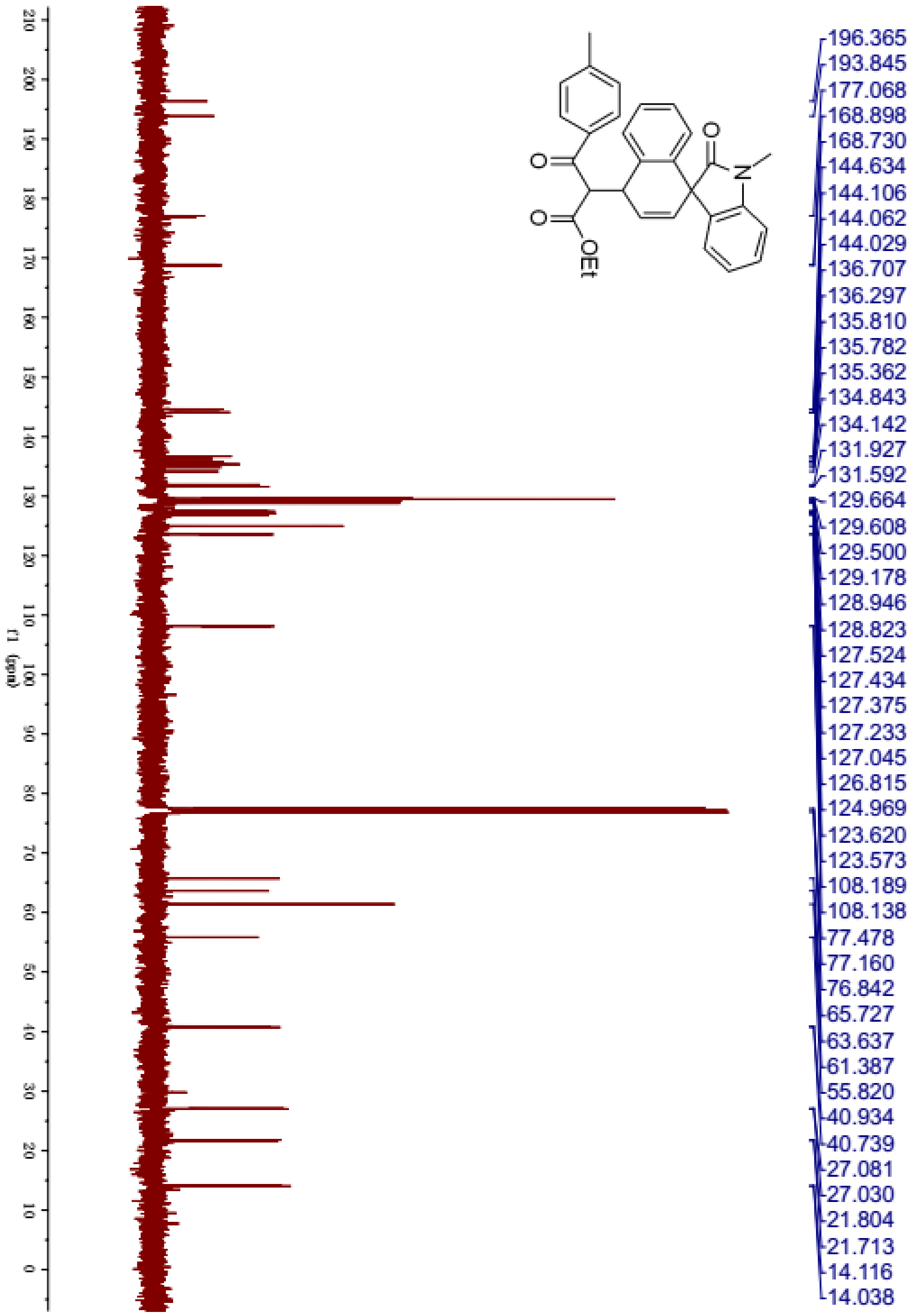
196.365
193.845
177.068
168.898
168.730
144.634
144.106
144.062
144.029
136.707
136.297
135.810
135.782
135.362
134.843
134.142
131.927
131.592
129.664
129.608
129.500
129.178
128.946
128.823
127.524
127.434
127.375
127.233
127.045
126.815
124.969
123.620
123.573
108.189
108.138
77.478
77.160
76.842
65.727
63.637
61.387
55.820
40.934
40.739
27.081
27.030
21.804
21.713
14.116
14.038
f1 (ppm)
OEt

5i

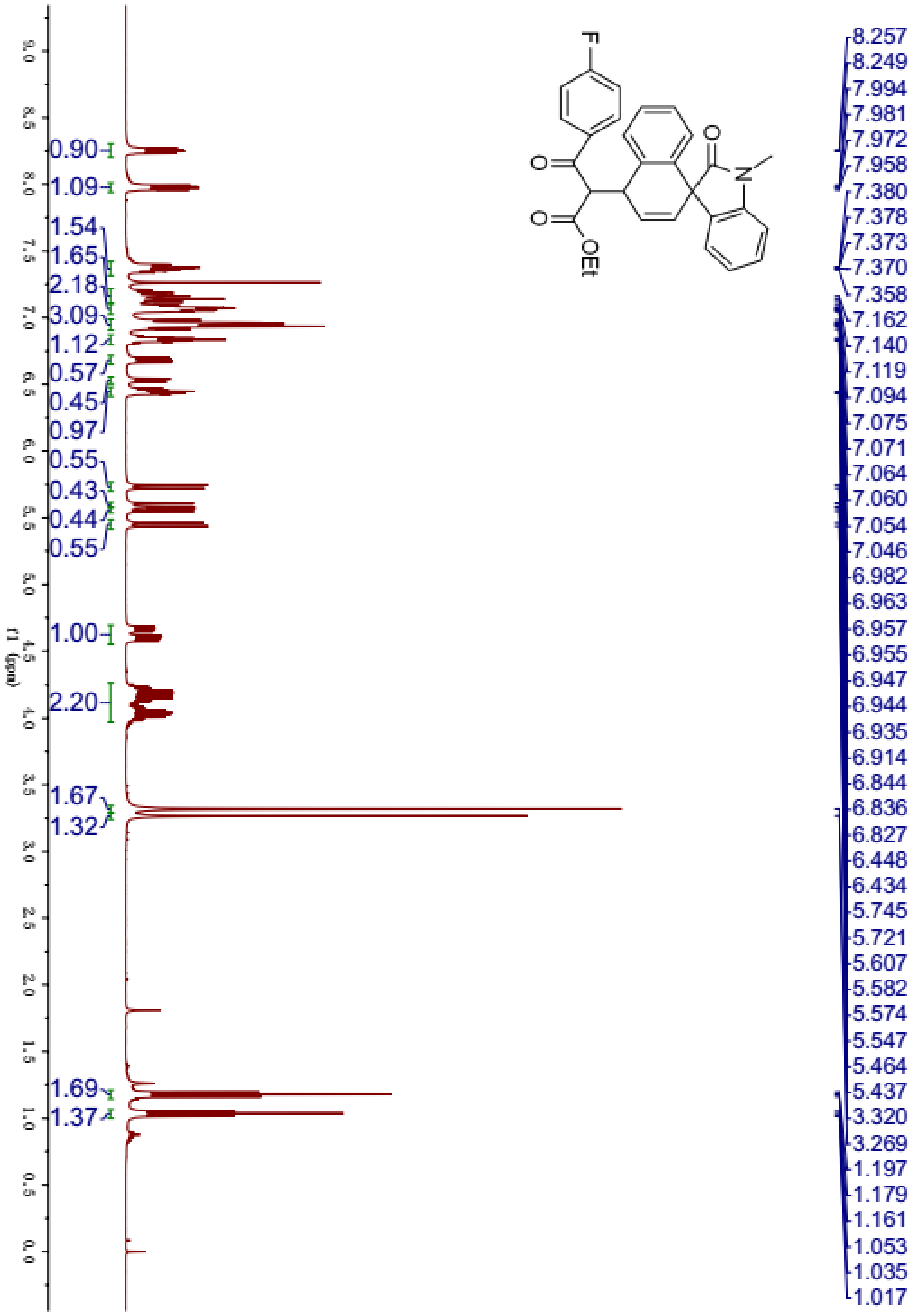
8.257
8.249
7.994
7.981
7.972
7.958
7.380
7.378
7.373
7.370
7.358
7.162
7.140
7.119
7.094
7.075
7.071
7.064
7.060
7.054
7.046
6.982
6.963
6.957
6.955
6.947
6.944
6.935
6.914
6.844
6.836
6.827
6.448
6.434
5.745
5.721
5.607
5.582
5.574
5.547
5.464
5.437
3.320
3.269
1.197
1.179
1.161
1.053
1.035
1.017
0.90
1.09
1.54
1.65
2.18
3.09
1.12
0.57
0.45
0.97
0.55
0.43
0.44
0.55
1.00
2.20
1.67
1.32
1.69
1.37
f1 (ppm)
F
O
OEt
N

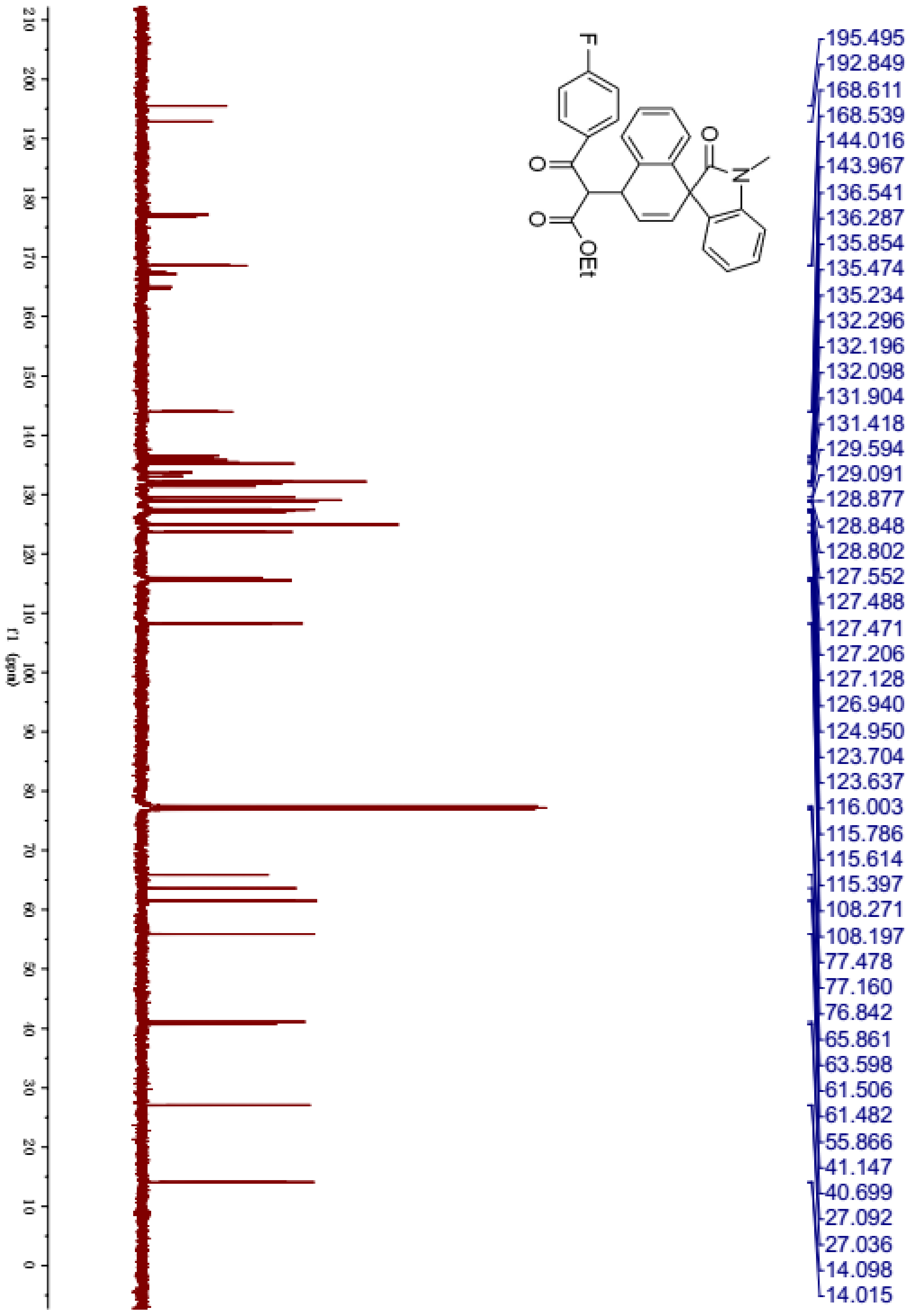
195.495
192.849
168.611
168.539
144.016
143.967
136.541
136.287
135.854
135.474
135.234
132.296
132.196
132.098
131.904
131.418
129.594
129.091
128.877
128.848
128.802
127.552
127.488
127.471
127.206
127.128
126.940
124.950
123.704
123.637
116.003
115.786
115.614
115.397
108.271
108.197
77.478
77.160
76.842
65.861
63.598
61.506
61.482
55.866
41.147
40.699
27.092
27.036
14.098
14.015
F
O
O
OEt
O
N
f1 (ppm)

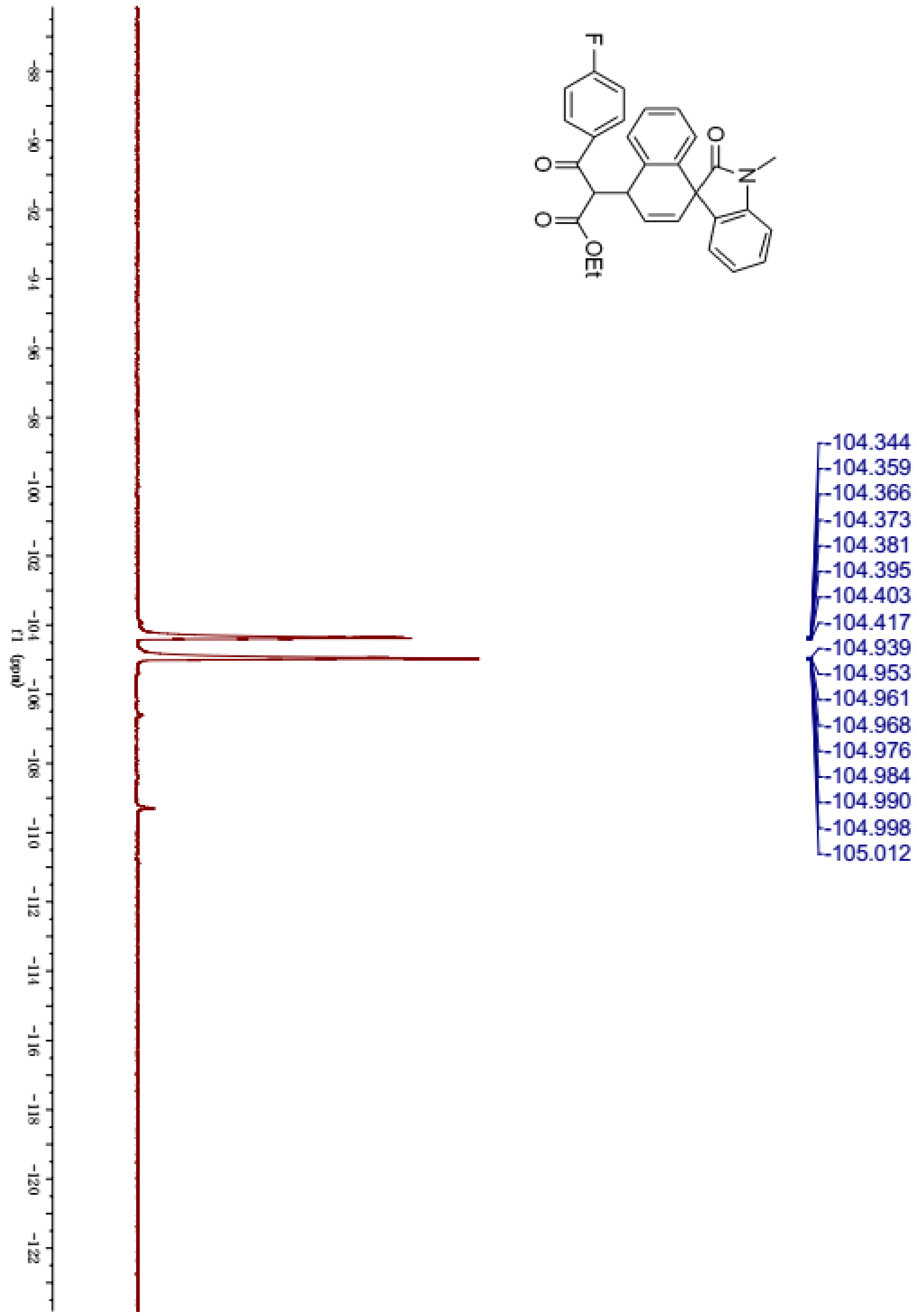
-104.344
-104.359
-104.366
-104.373
-104.381
-104.395
-104.403
-104.417
-104.939
-104.953
-104.961
-104.968
-104.976
-104.984
-104.990
-104.998
-105.012
f1 (ppm)
O
O
OEt
F
O
N

5j

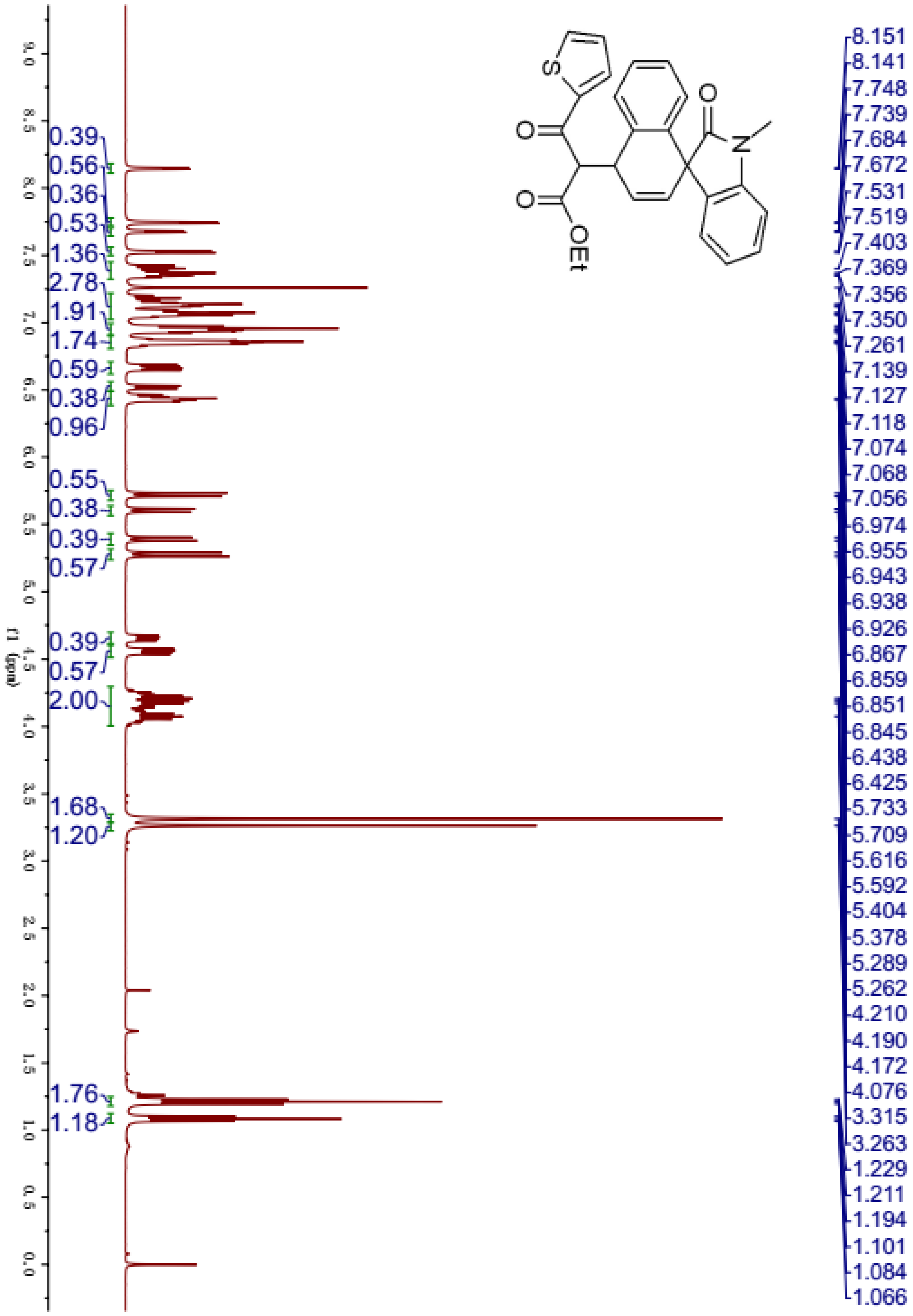
8.151
8.141
7.748
7.739
7.684
7.672
7.531
7.519
7.403
7.369
7.356
7.350
7.261
7.139
7.127
7.118
7.074
7.068
7.056
6.974
6.955
6.943
6.938
6.926
6.867
6.859
6.851
6.845
6.438
6.425
5.733
5.709
5.616
5.592
5.404
5.378
5.289
5.262
4.210
4.190
4.172
4.076
3.315
3.263
1.229
1.211
1.194
1.101
1.084
1.066
0.39
0.56
0.36
0.53
1.36
2.78
1.91
1.74
0.59
0.38
0.96
0.55
0.38
0.39
0.57
0.39
0.57
2.00
1.68
1.20
1.76
1.18
f1 (ppm)
S
O
O
OEt
O
N

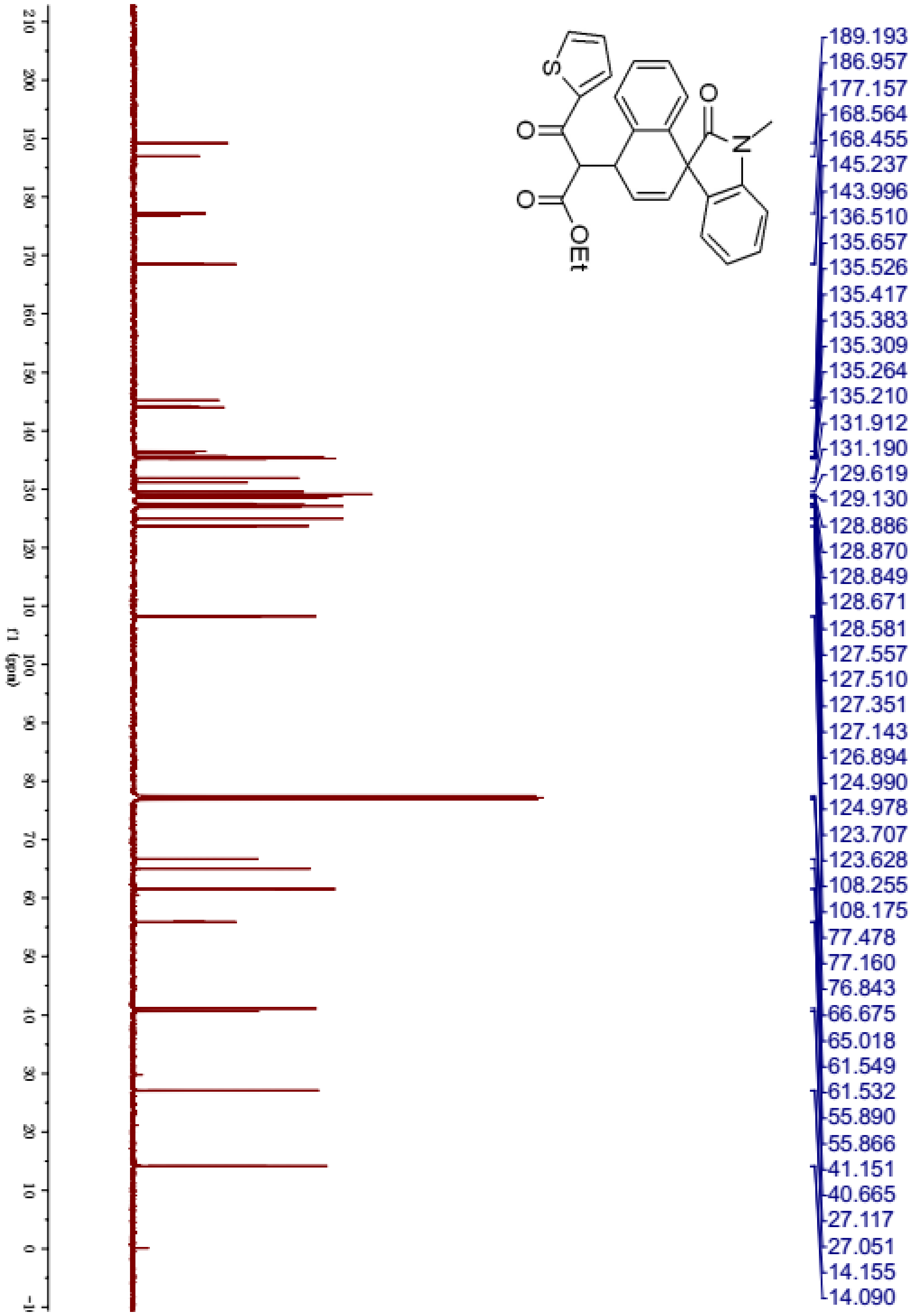
189.193
186.957
177.157
168.564
168.455
145.237
143.996
136.510
135.657
135.526
135.417
135.383
135.309
135.264
135.210
131.912
131.190
129.619
129.130
128.886
128.870
128.849
128.671
128.581
127.557
127.510
127.351
127.143
126.894
124.990
124.978
123.707
123.628
108.255
108.175
77.478
77.160
76.843
66.675
65.018
61.549
61.532
55.890
55.866
41.151
40.665
27.117
27.051
14.155
14.090
f1 (ppm)
OEt
O
O
S
O
N

5k

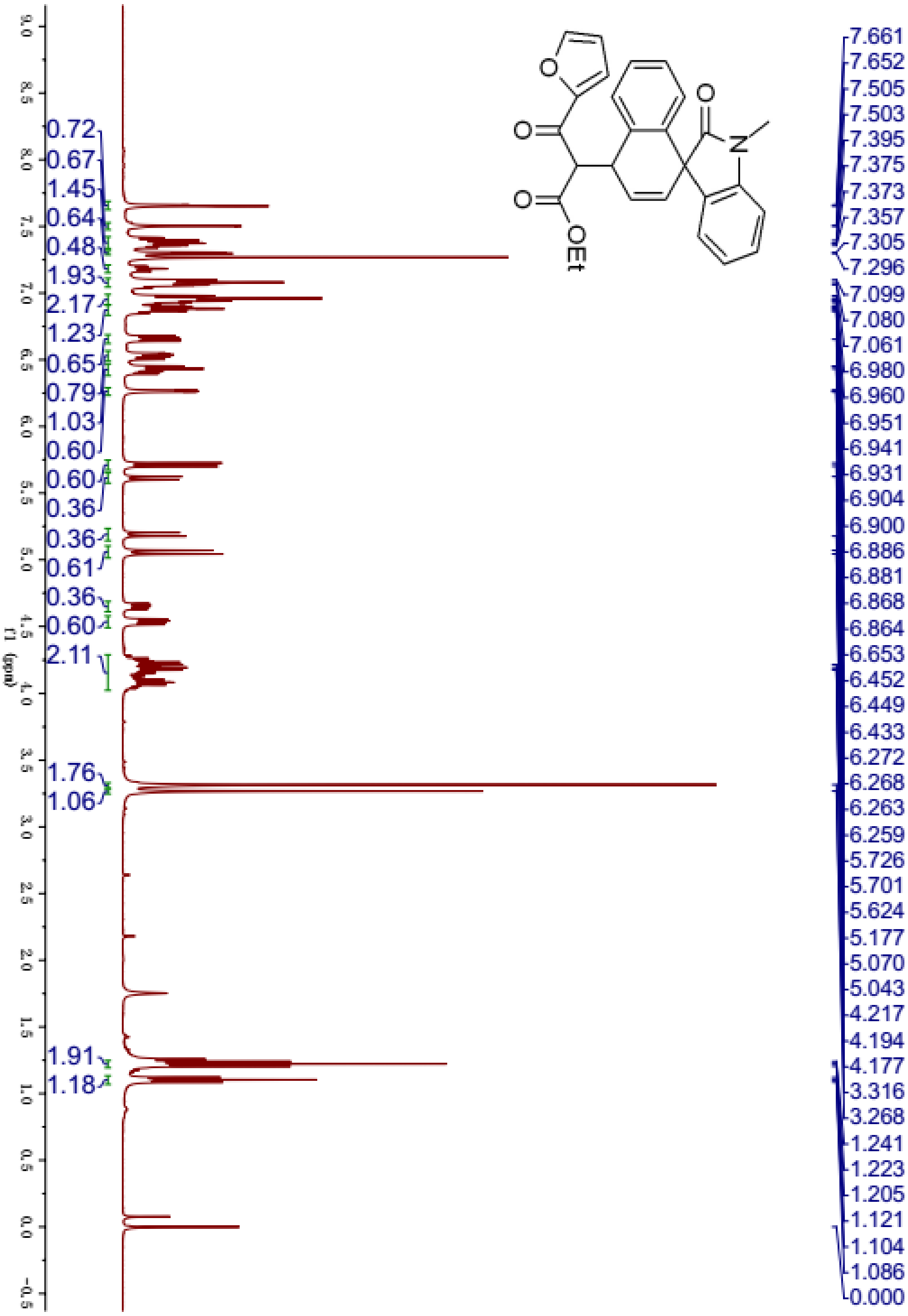
7.661
7.652
7.505
7.503
7.395
7.375
7.373
7.357
7.305
7.296
7.099
7.080
7.061
6.980
6.960
6.951
6.941
6.931
6.904
6.900
6.886
6.881
6.868
6.864
6.653
6.452
6.449
6.433
6.272
6.268
6.263
6.259
5.726
5.701
5.624
5.177
5.070
5.043
4.217
4.194
4.177
3.316
3.268
1.241
1.223
1.205
1.121
1.104
1.086
0.000
0.72
0.67
1.45
0.64
0.48
1.93
2.17
1.23
0.65
0.79
1.03
0.60
0.60
0.36
0.36
0.61
0.36
0.60
2.11
1.76
1.06
1.91
1.18
f1 (ppm)
O
N
OEt

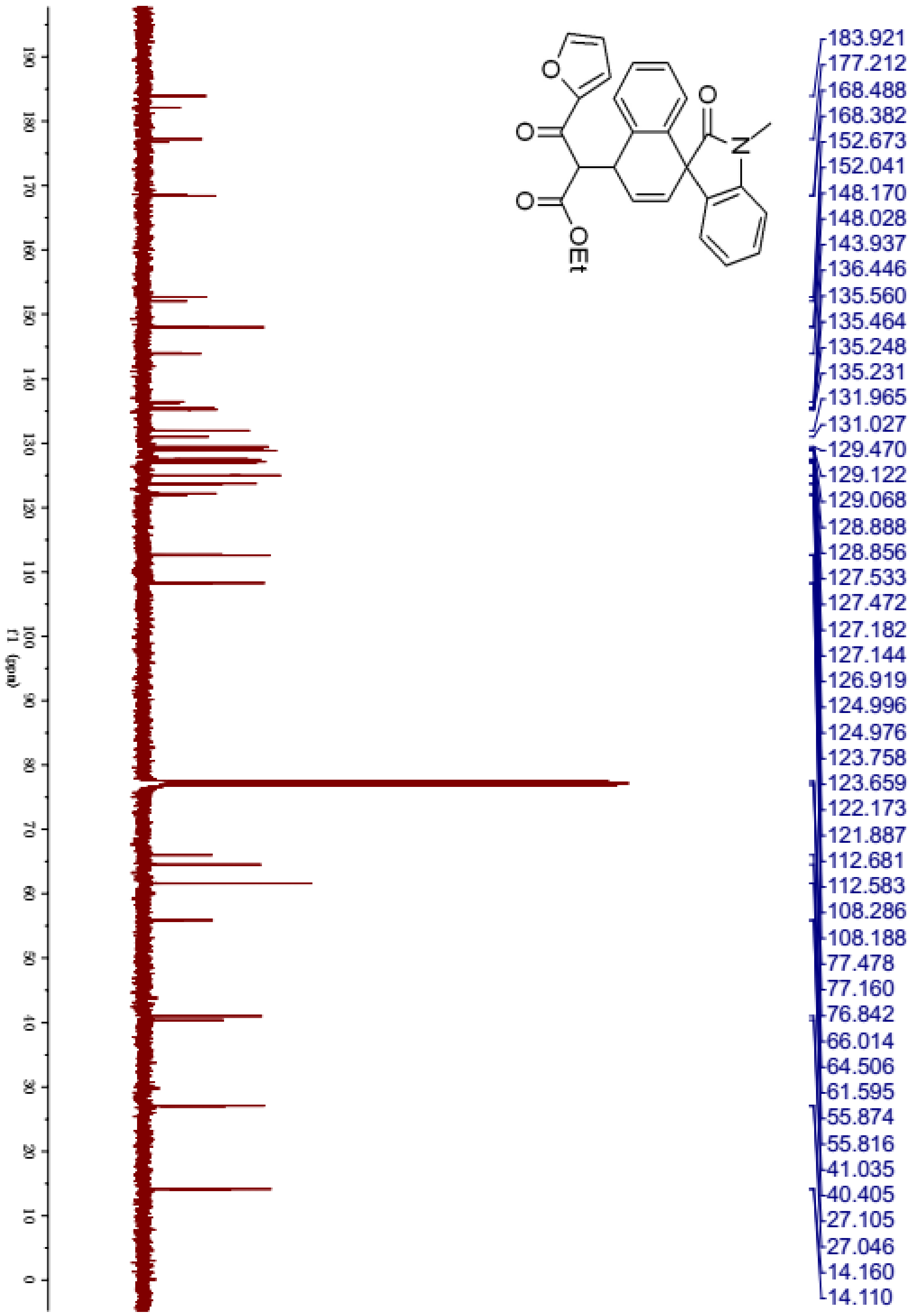
183.921
177.212
168.488
168.382
152.673
152.041
148.170
148.028
143.937
136.446
135.560
135.464
135.248
135.231
131.965
131.027
129.470
129.122
129.068
128.888
128.856
127.533
127.472
127.182
127.144
126.919
124.996
124.976
123.758
123.659
122.173
121.887
112.681
112.583
108.286
108.188
77.478
77.160
76.842
66.014
64.506
61.595
55.874
55.816
41.035
40.405
27.105
27.046
14.160
14.110
OEt

5l

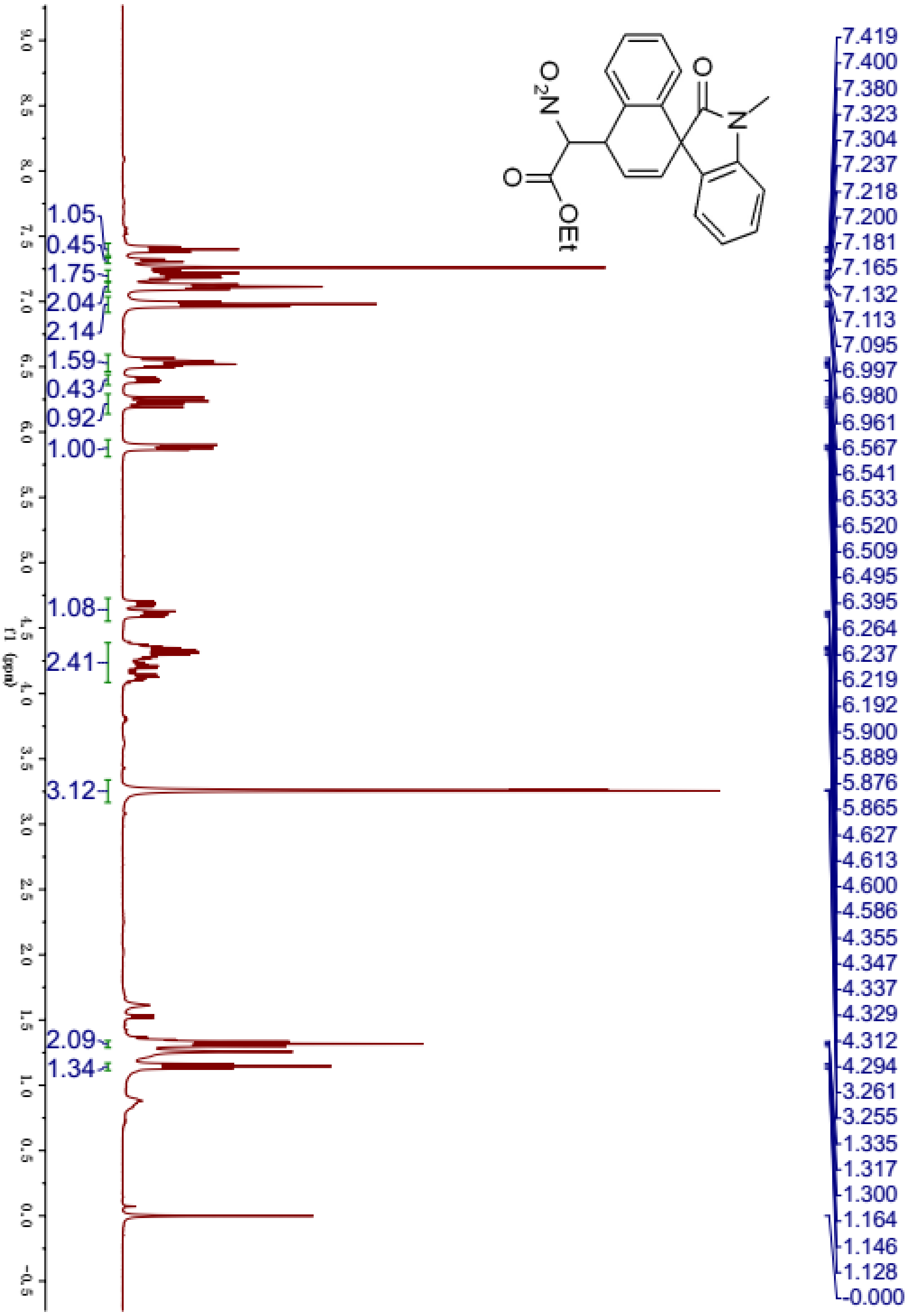
O2N
O
OEt
O
N
7.419
7.400
7.380
7.323
7.304
7.237
7.218
7.200
7.181
7.165
7.132
7.113
7.095
6.997
6.980
6.961
6.567
6.541
6.533
6.520
6.509
6.495
6.395
6.264
6.237
6.219
6.192
5.900
5.889
5.876
5.865
4.627
4.613
4.600
4.586
4.355
4.347
4.337
4.329
4.312
4.294
3.261
3.255
1.335
1.317
1.300
1.164
1.146
1.128
-0.000
1.05
0.45
1.75
2.04
2.14
1.59
0.43
0.92
1.00
1.08
2.41
3.12
2.09
1.34
f1 (ppm)

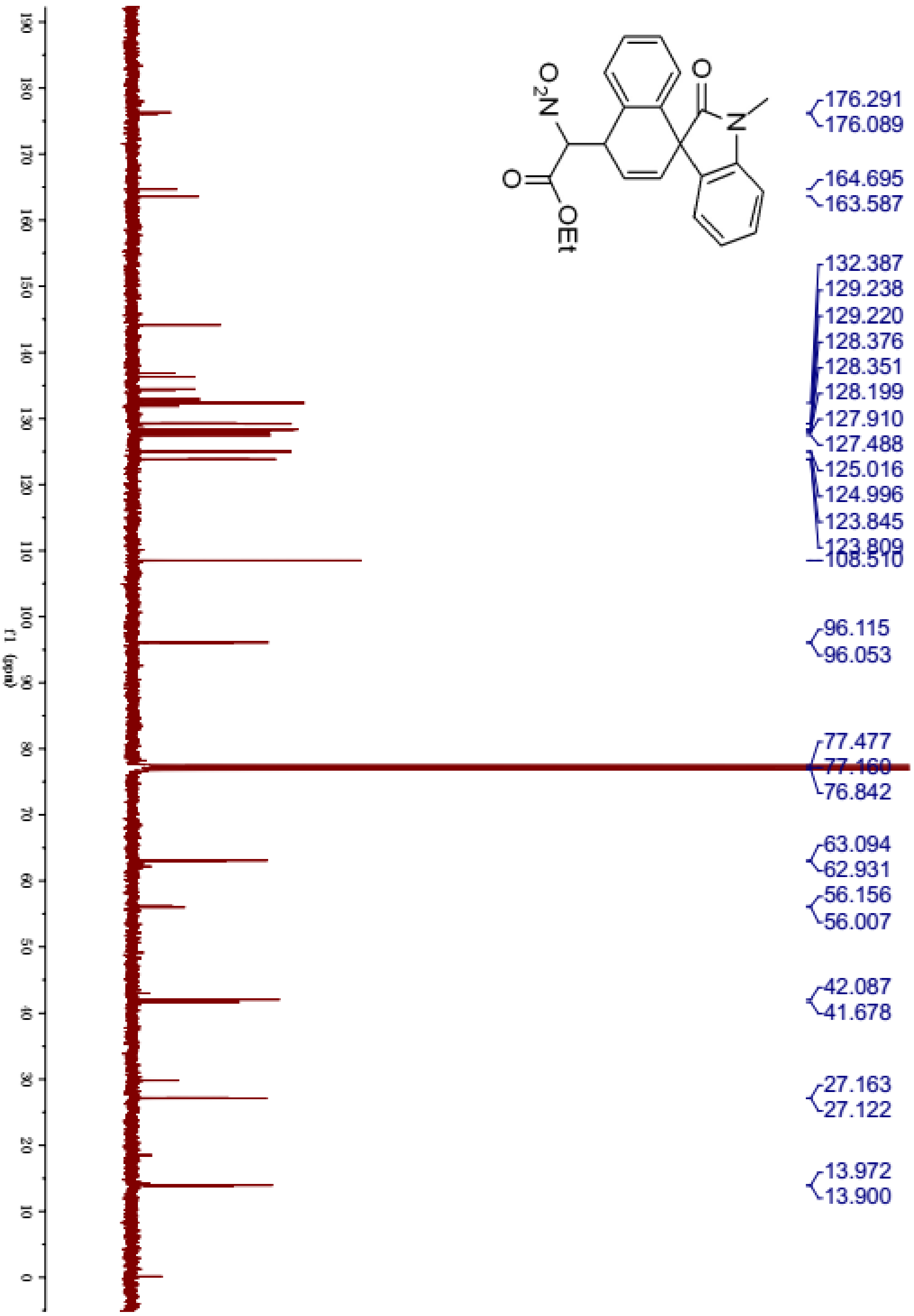
176.291
176.089
164.695
163.587
132.387
129.238
129.220
128.376
128.351
128.199
127.910
127.488
125.016
124.996
123.845
123.809
108.510
96.115
96.053
77.477
77.160
76.842
63.094
62.931
56.156
56.007
42.087
41.678
27.163
27.122
13.972
13.900
f1 (ppm)
O2N
OEt
O
N

5m

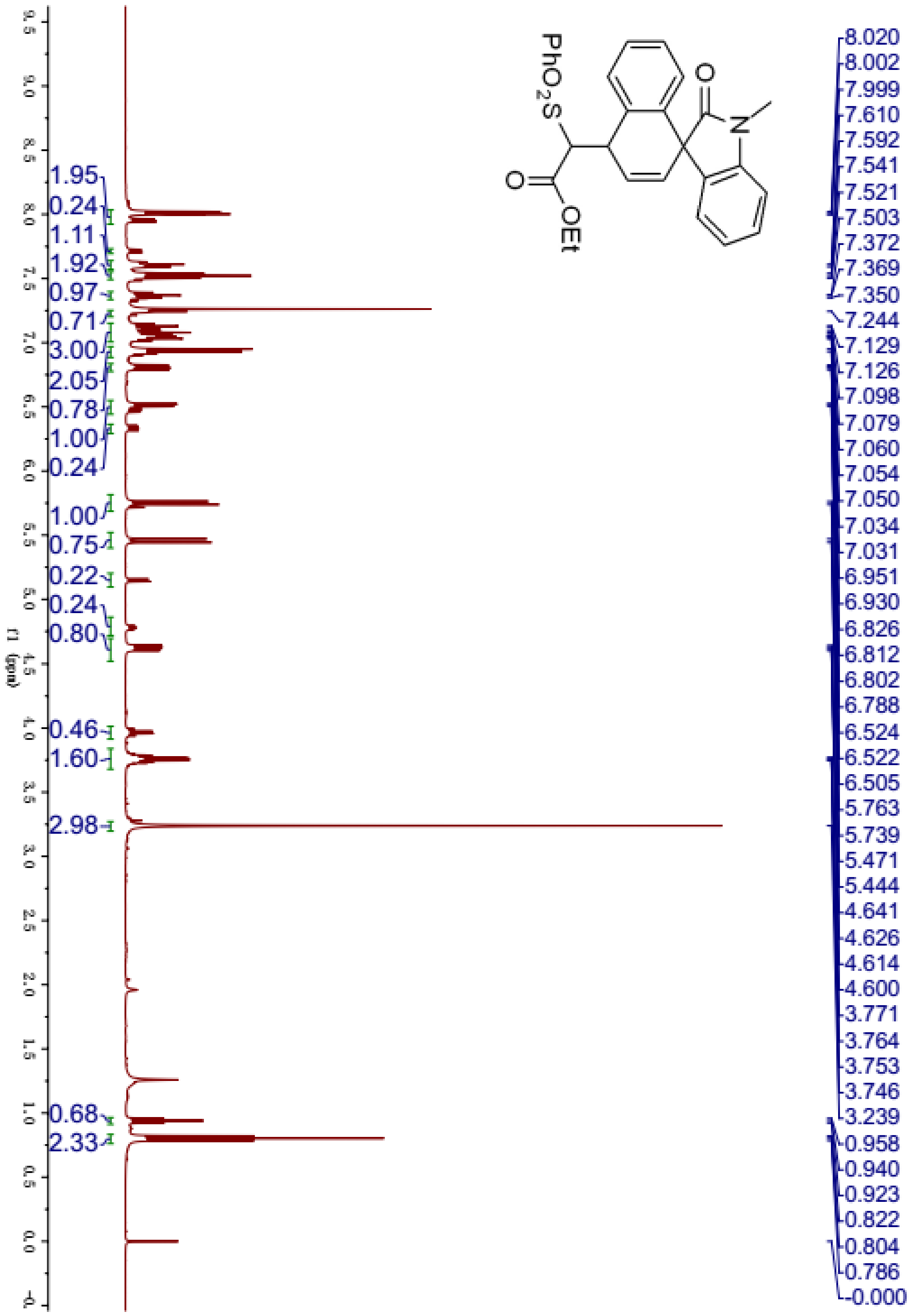
PhO2S
O
OEt
O
N
8.020
8.002
7.999
7.610
7.592
7.541
7.521
7.503
7.372
7.369
7.350
7.244
7.129
7.126
7.098
7.079
7.060
7.054
7.050
7.034
7.031
6.951
6.930
6.826
6.812
6.802
6.788
6.524
6.522
6.505
5.763
5.739
5.471
5.444
4.641
4.626
4.614
4.600
3.771
3.764
3.753
3.746
3.239
0.958
0.940
0.923
0.822
0.804
0.786
-0.000
1.95
0.24
1.11
1.92
0.97
0.71
3.00
2.05
0.78
1.00
0.24
1.00
0.75
0.22
0.24
0.80
0.46
1.60
2.98
0.68
2.33
f1 (ppm)

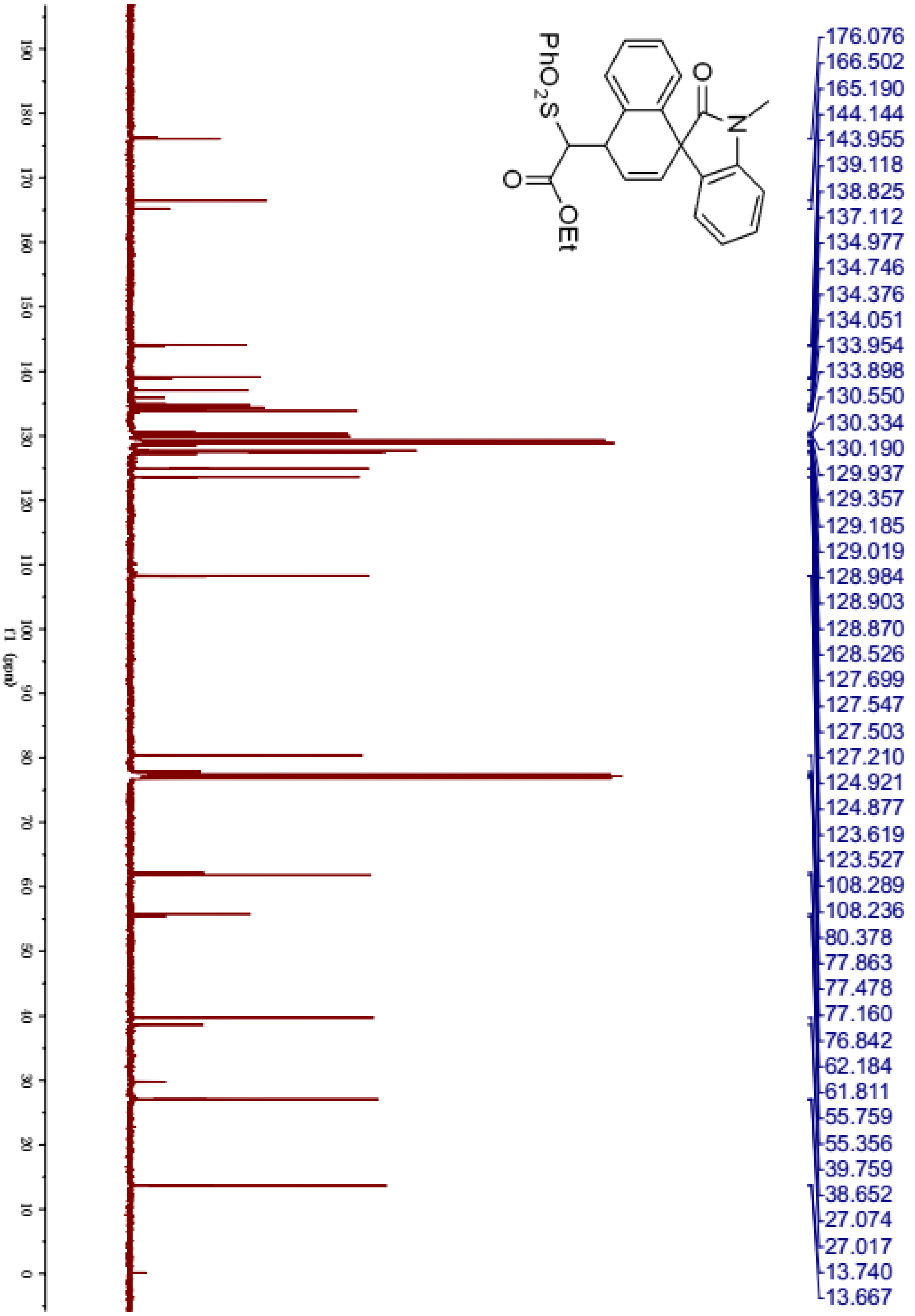
176.076
166.502
165.190
144.144
143.955
139.118
138.825
137.112
134.977
134.746
134.376
134.051
133.954
133.898
130.550
130.334
130.190
129.937
129.357
129.185
129.019
128.984
128.903
128.870
128.526
127.699
127.547
127.503
127.210
124.921
124.877
123.619
123.527
108.289
108.236
80.378
77.863
77.478
77.160
76.842
62.184
61.811
55.759
55.356
39.759
38.652
27.074
27.017
13.740
13.667
PhO2S
OEt
O
N
f1 (ppm)

6a

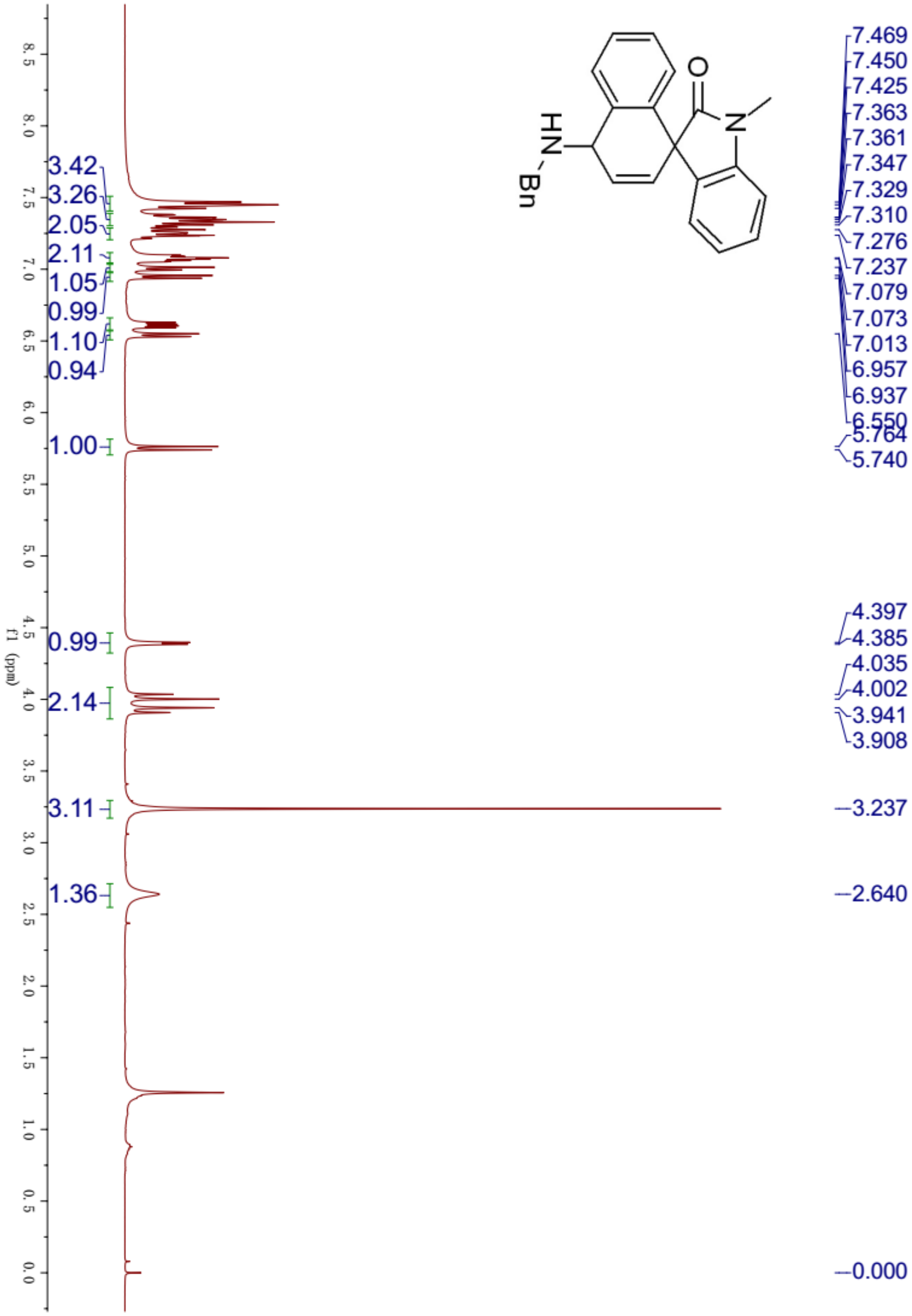
HN
Bn
O
N
7.469
7.450
7.425
7.363
7.361
7.347
7.329
7.310
7.276
7.237
7.079
7.073
7.013
6.957
6.937
6.550
5.764
5.740
4.397
4.385
4.035
4.002
3.941
3.908
3.237
2.640
0.000
3.42
3.26
2.05
2.11
1.05
0.99
1.10
0.94
1.00
0.99
2.14
3.11
1.36
f1 (ppm)

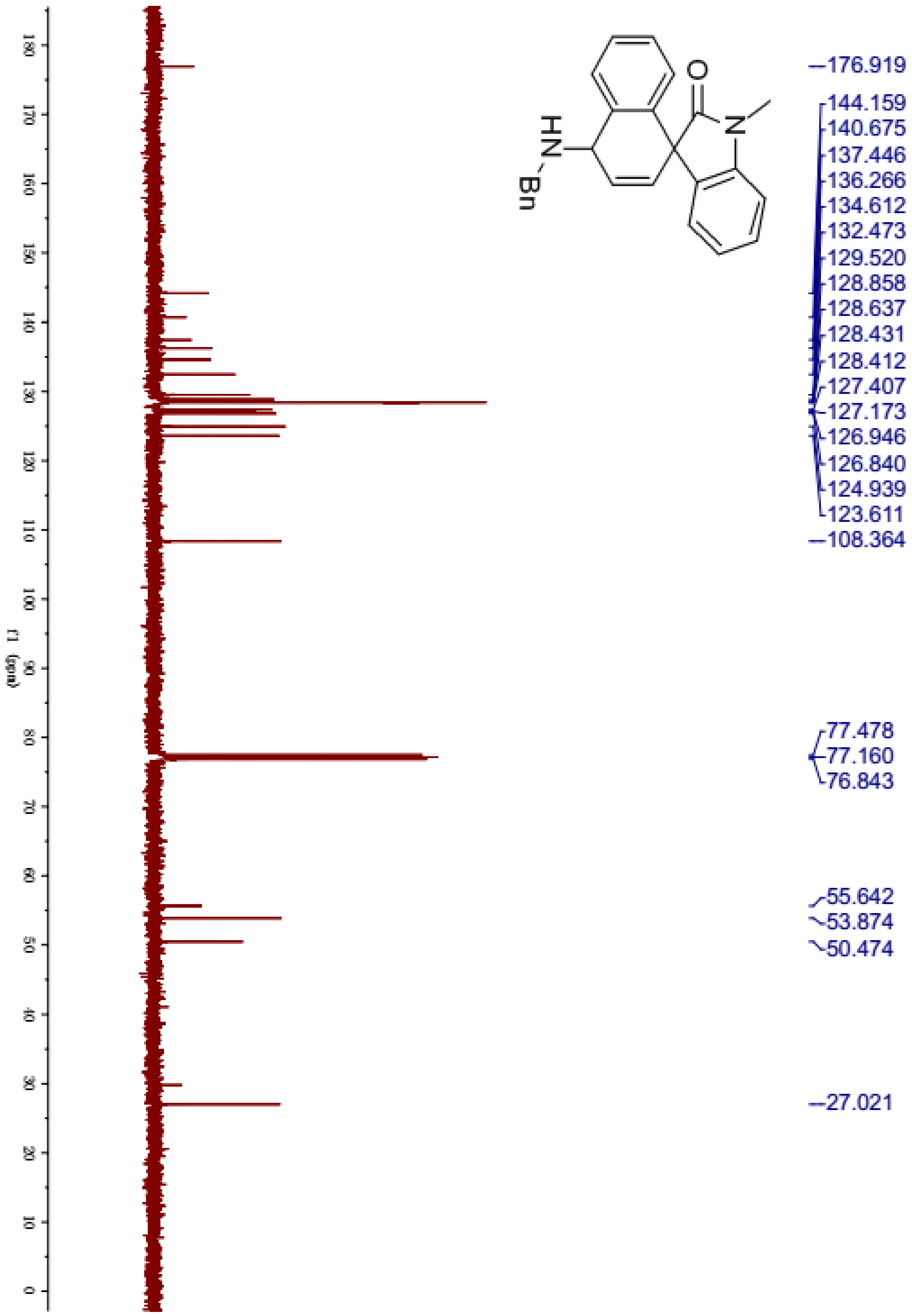

HN
Bn
O
N
176.919
144.159
140.675
137.446
136.266
134.612
132.473
129.520
128.858
128.637
128.431
128.412
127.407
127.173
126.946
126.840
124.939
123.611
108.364
77.478
77.160
76.843
55.642
53.874
50.474
27.021

6b

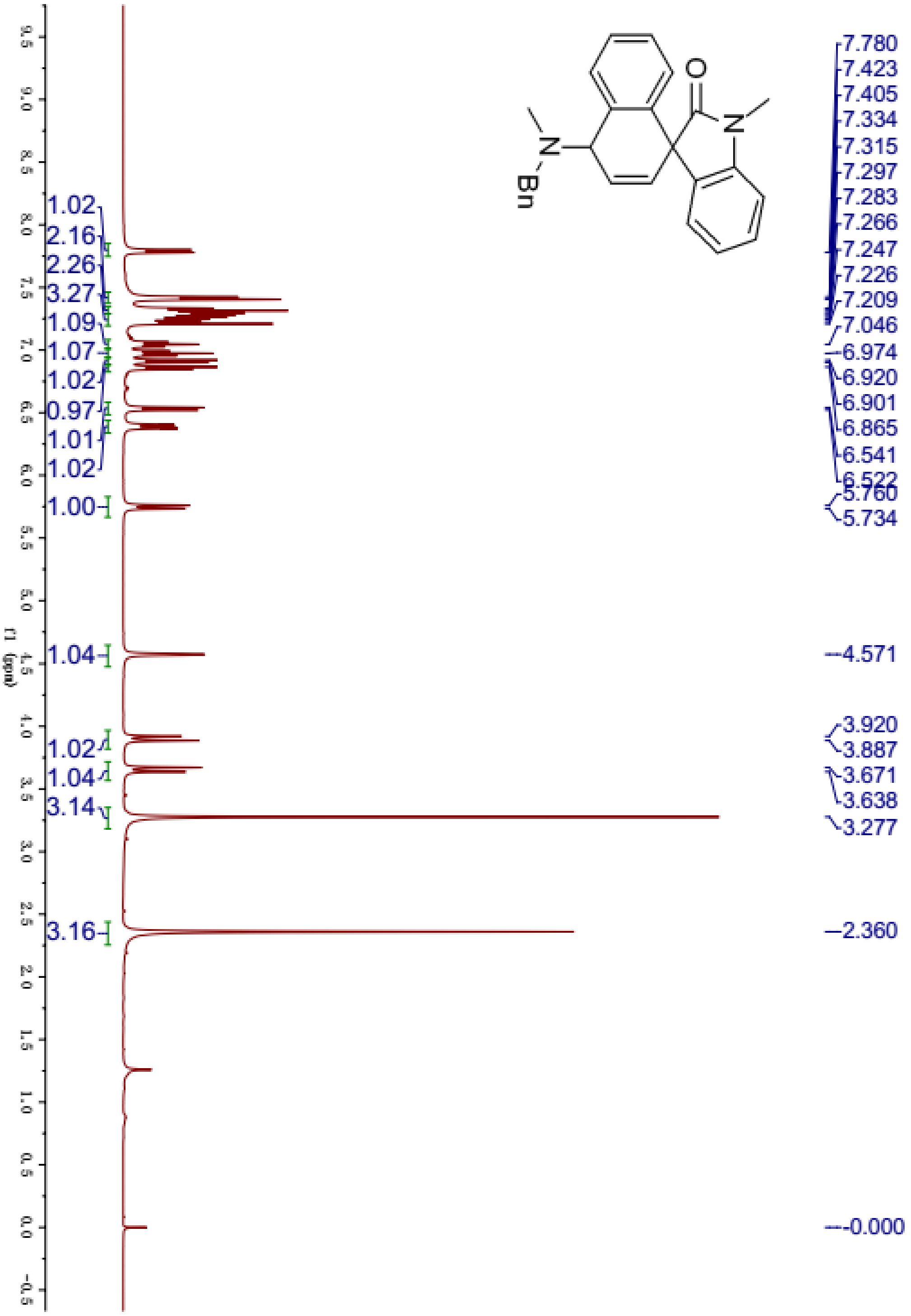
Bn
N
O
N
7.780
7.423
7.405
7.334
7.315
7.297
7.283
7.266
7.247
7.226
7.209
7.046
6.974
6.920
6.901
6.865
6.541
6.522
5.760
5.734
4.571
3.920
3.887
3.671
3.638
3.277
2.360
0.000
1.02
2.16
2.26
3.27
1.09
1.07
1.02
0.97
1.01
1.02
1.00
1.04
1.02
1.04
3.14
3.16
f1 (ppm)

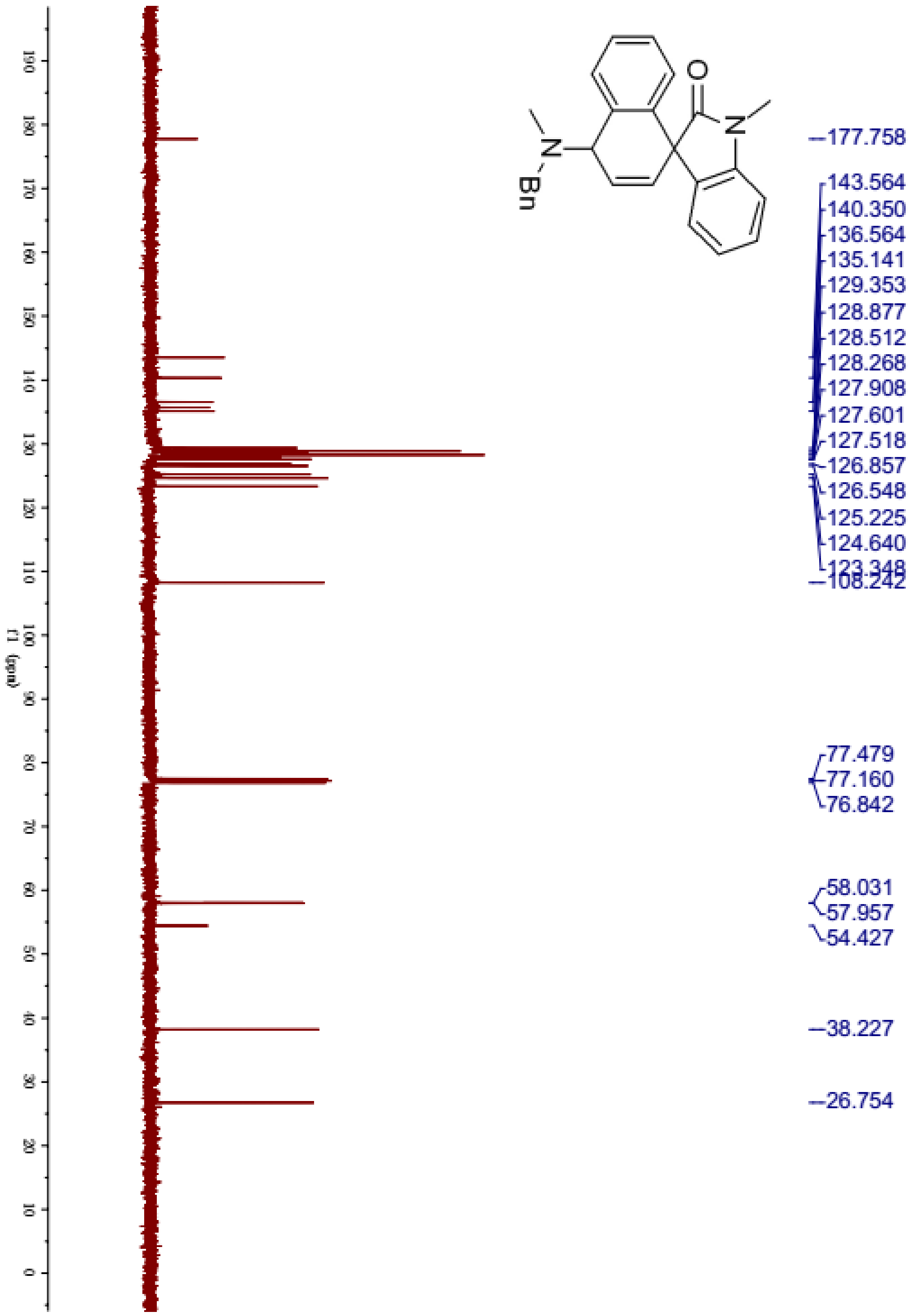
Bn
N
O
N
177.758
143.564
140.350
136.564
135.141
129.353
128.877
128.512
128.268
127.908
127.601
127.518
126.857
126.548
125.225
124.640
123.348
108.242
77.479
77.160
76.842
58.031
57.957
54.427
38.227
26.754
f1 (ppm)

6c

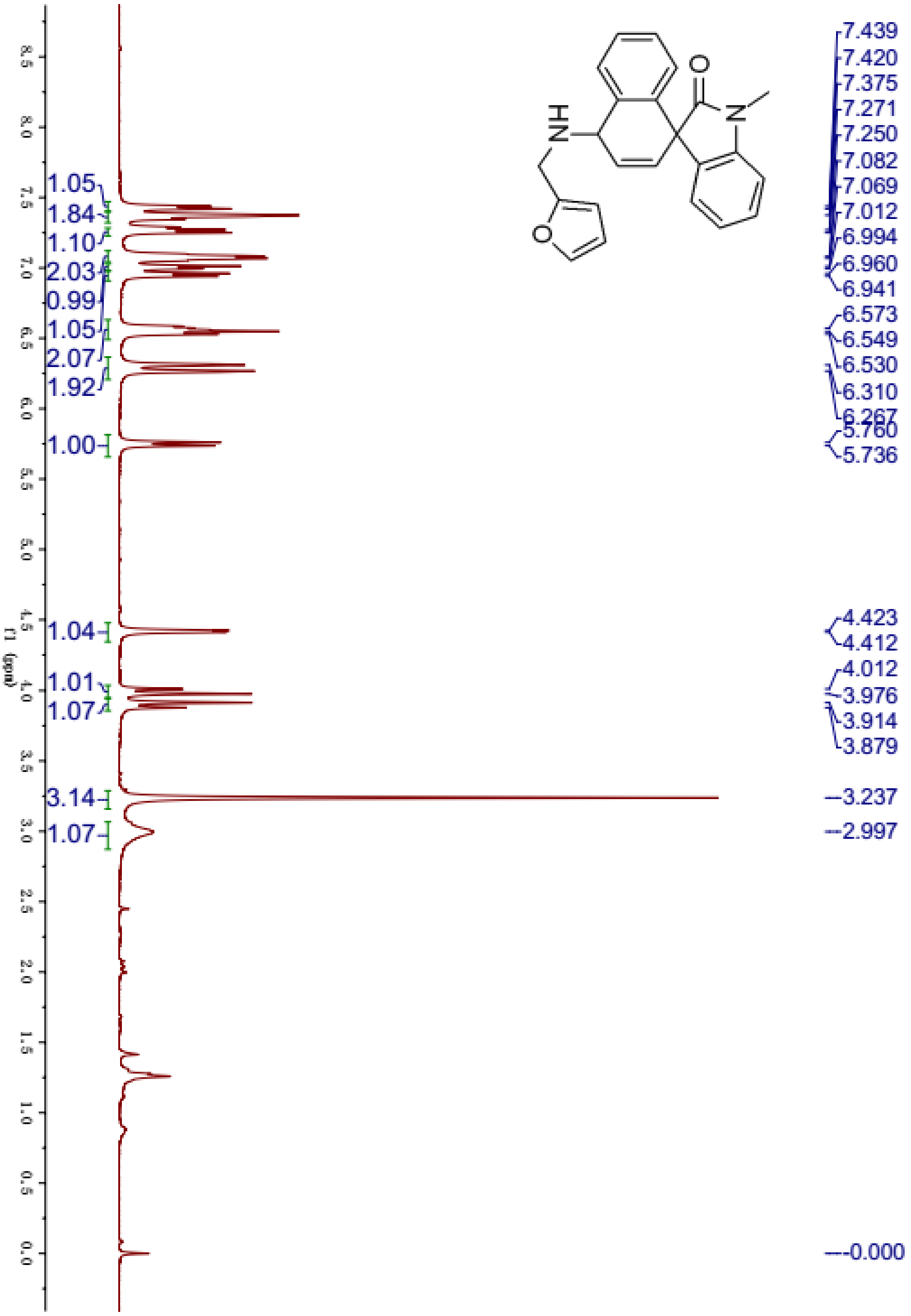
7.439
7.420
7.375
7.271
7.250
7.082
7.069
7.012
6.994
6.960
6.941
6.573
6.549
6.530
6.310
6.267
5.760
5.736
4.423
4.412
4.012
3.976
3.914
3.879
3.237
2.997
-0.000
1.05
1.84
1.10
2.03
0.99
1.05
2.07
1.92
1.00
1.04
1.01
1.07
3.14
1.07

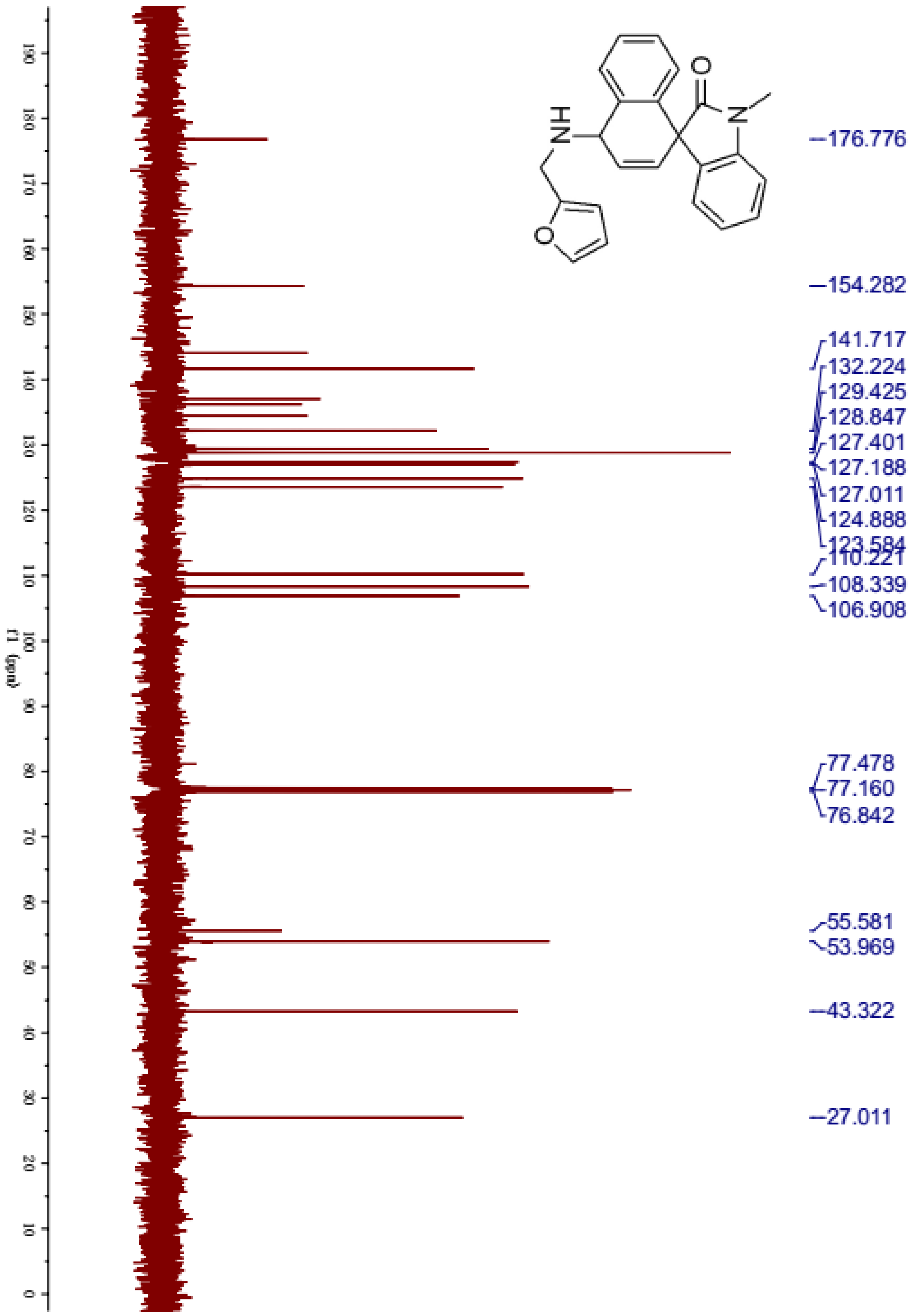
176.776
154.282
141.717
132.224
129.425
128.847
127.401
127.188
127.011
124.888
123.584
110.221
108.339
106.908
77.478
77.160
76.842
55.581
53.969
43.322
27.011
HN
O
N
O

6d

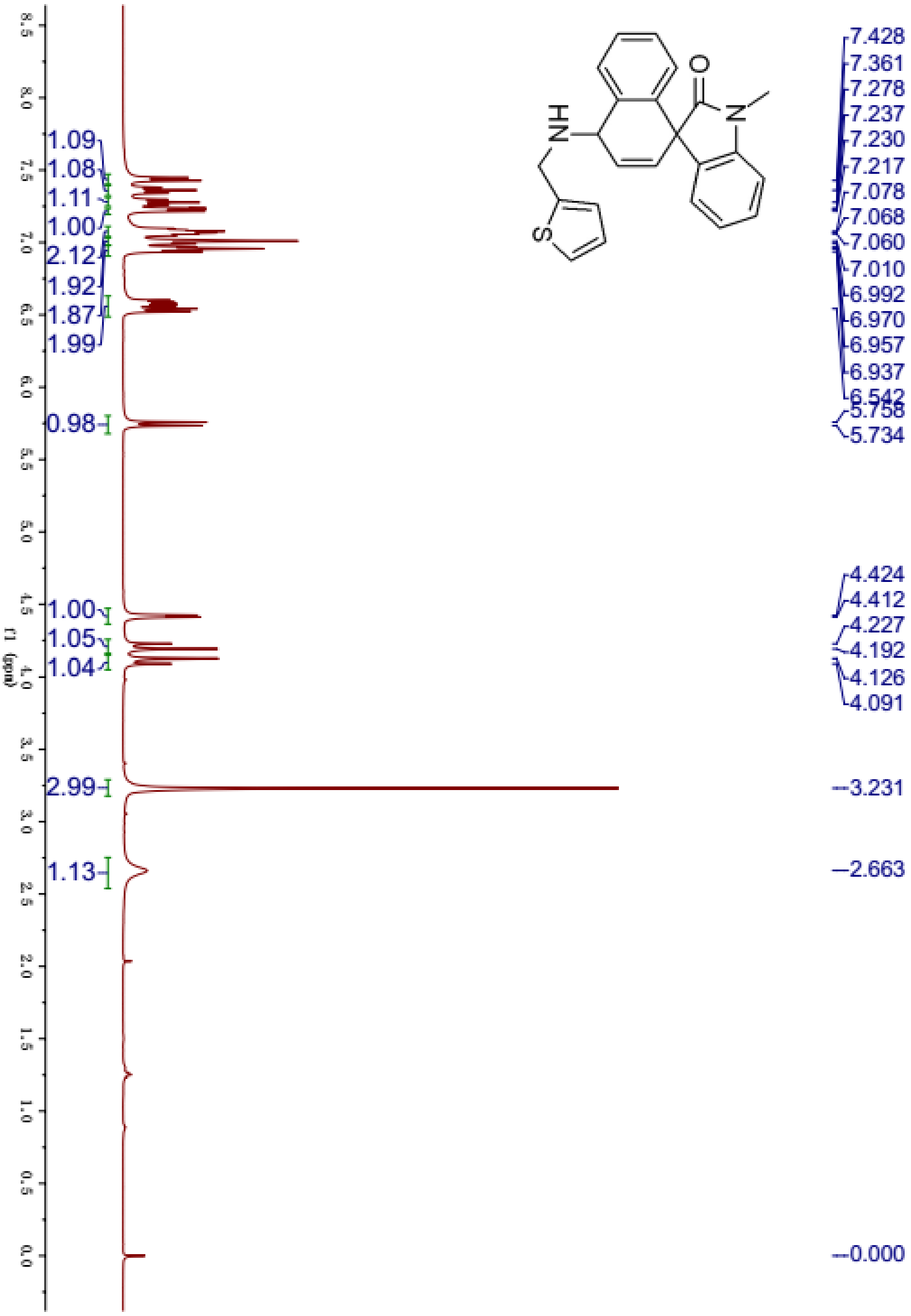
7.428
7.361
7.278
7.237
7.230
7.217
7.078
7.068
7.060
7.010
6.992
6.970
6.957
6.937
6.542
5.758
5.734
4.424
4.412
4.227
4.192
4.126
4.091
3.231
2.663
0.000
1.09
1.08
1.11
1.00
2.12
1.92
1.87
1.99
0.98
1.00
1.05
1.04
2.99
1.13
f1 (ppm)
HN
S
O
N

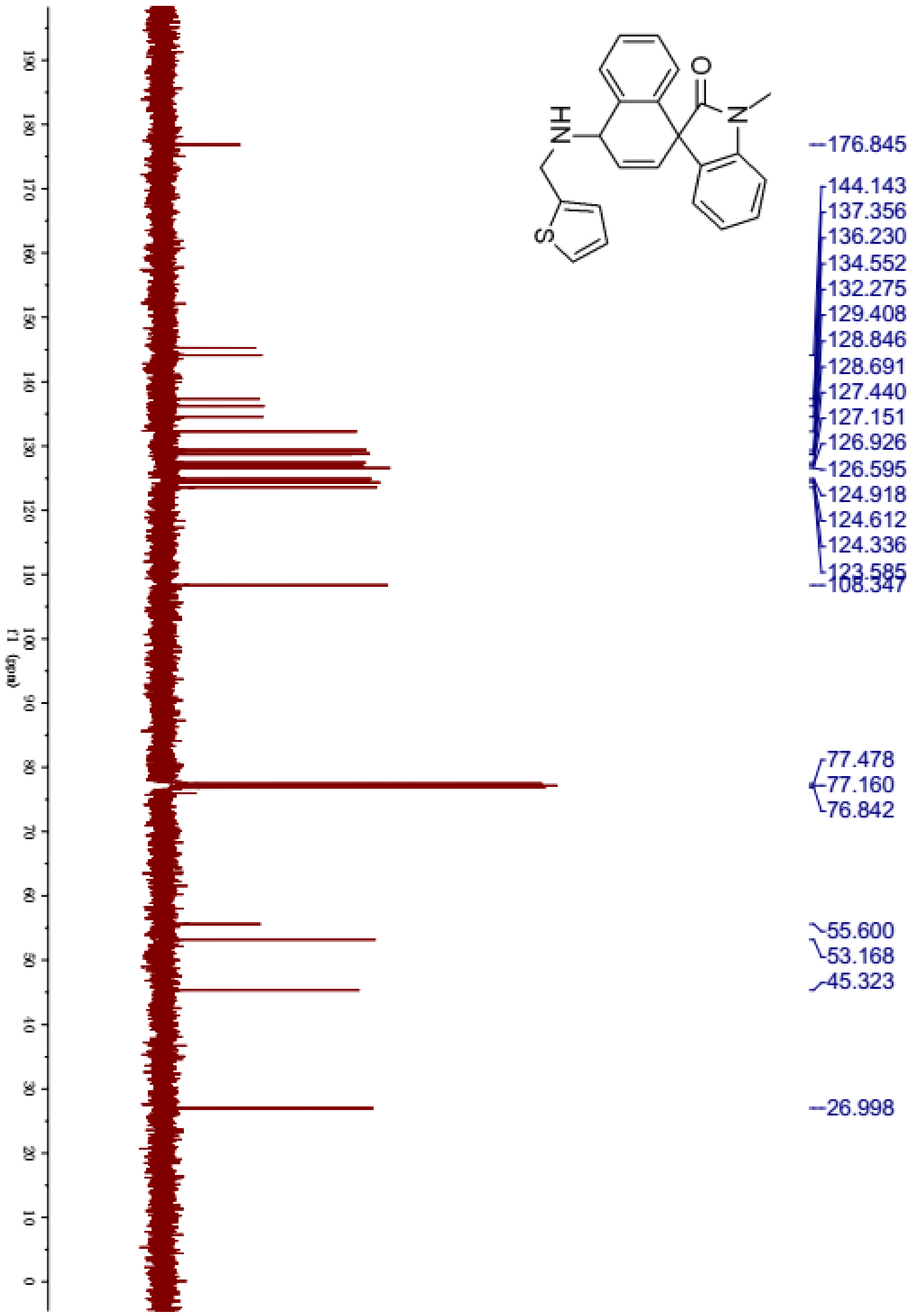

176.845
144.143
137.356
136.230
134.552
132.275
129.408
128.846
128.691
127.440
127.151
126.926
126.595
124.918
124.612
124.336
123.585
108.347
77.478
77.160
76.842
55.600
53.168
45.323
26.998
f1 (ppm)
HN
O
N
S

6e

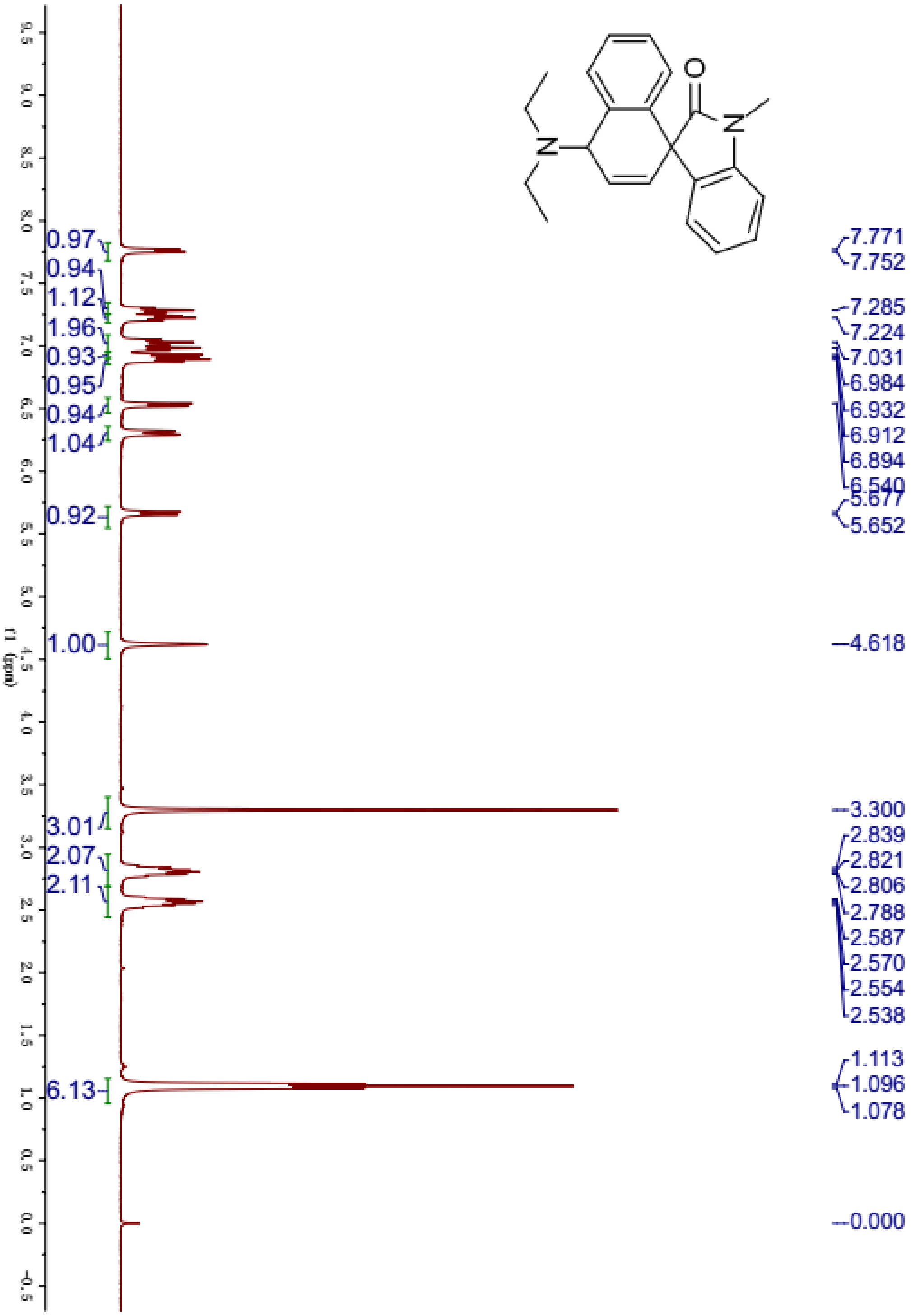
7.771
7.752
7.285
7.224
7.031
6.984
6.932
6.912
6.894
6.540
5.677
5.652
4.618
3.300
2.839
2.821
2.806
2.788
2.587
2.570
2.554
2.538
1.113
1.096
1.078
0.000
0.97
0.94
1.12
1.96
0.93
0.95
0.94
1.04
0.92
1.00
3.01
2.07
2.11
6.13
f1 (ppm)

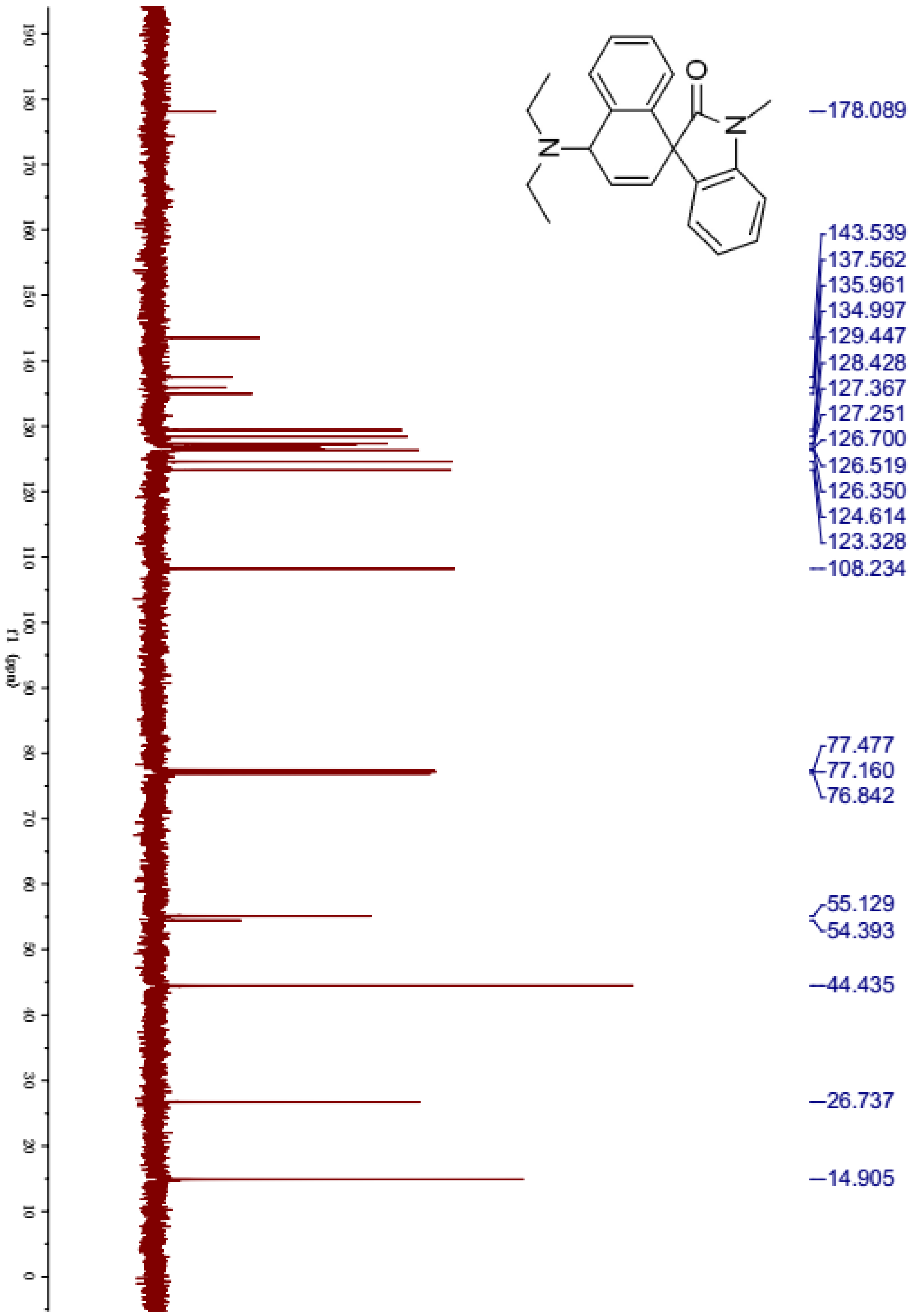
178.089
143.539
137.562
135.961
134.997
129.447
128.428
127.367
127.251
126.700
126.519
126.350
124.614
123.328
108.234
77.477
77.160
76.842
55.129
54.393
44.435
26.737
14.905
f1 (ppm)

6f

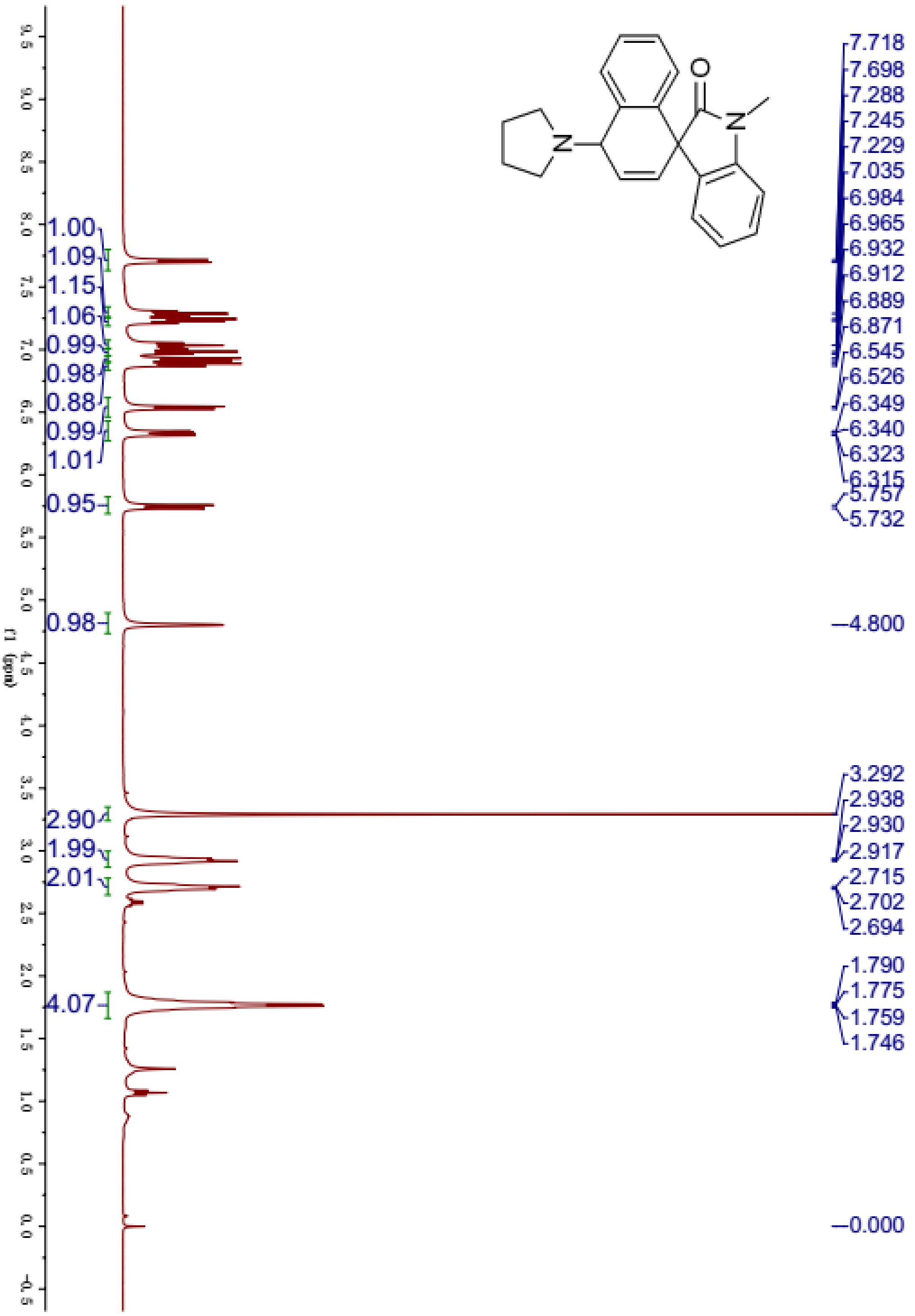
7.718
7.698
7.288
7.245
7.229
7.035
6.984
6.965
6.932
6.912
6.889
6.871
6.545
6.526
6.349
6.340
6.323
6.315
5.757
5.732
4.800
3.292
2.938
2.930
2.917
2.715
2.702
2.694
1.790
1.775
1.759
1.746
0.000
1.00
1.09
1.15
1.06
0.99
0.98
0.88
0.99
1.01
0.95
0.98
2.90
1.99
2.01
4.07
f1 (ppm)

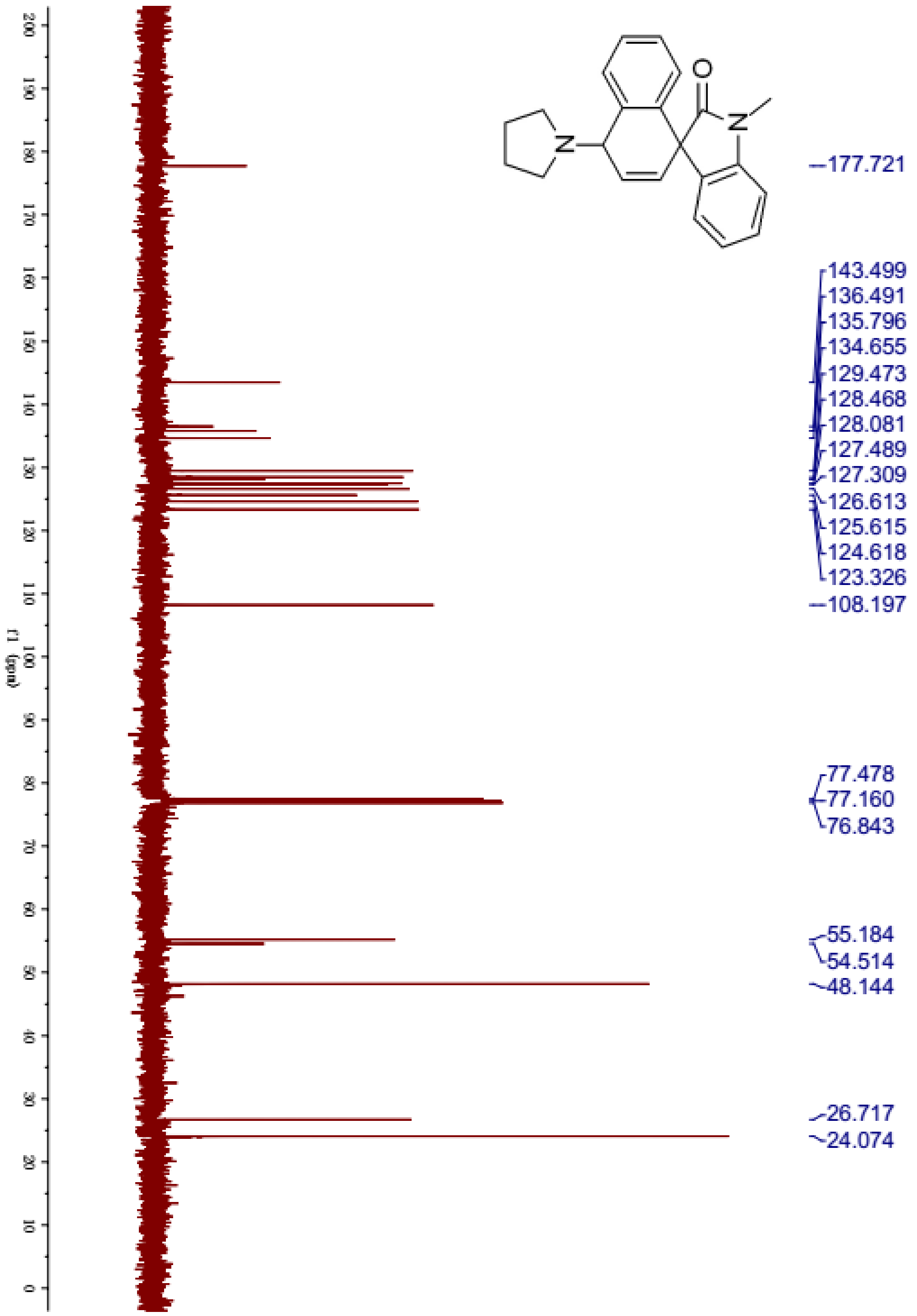
177.721
143.499
136.491
135.796
134.655
129.473
128.468
128.081
127.489
127.309
126.613
125.615
124.618
123.326
108.197
77.478
77.160
76.843
55.184
54.514
48.144
26.717
24.074
f1 (ppm)

6g

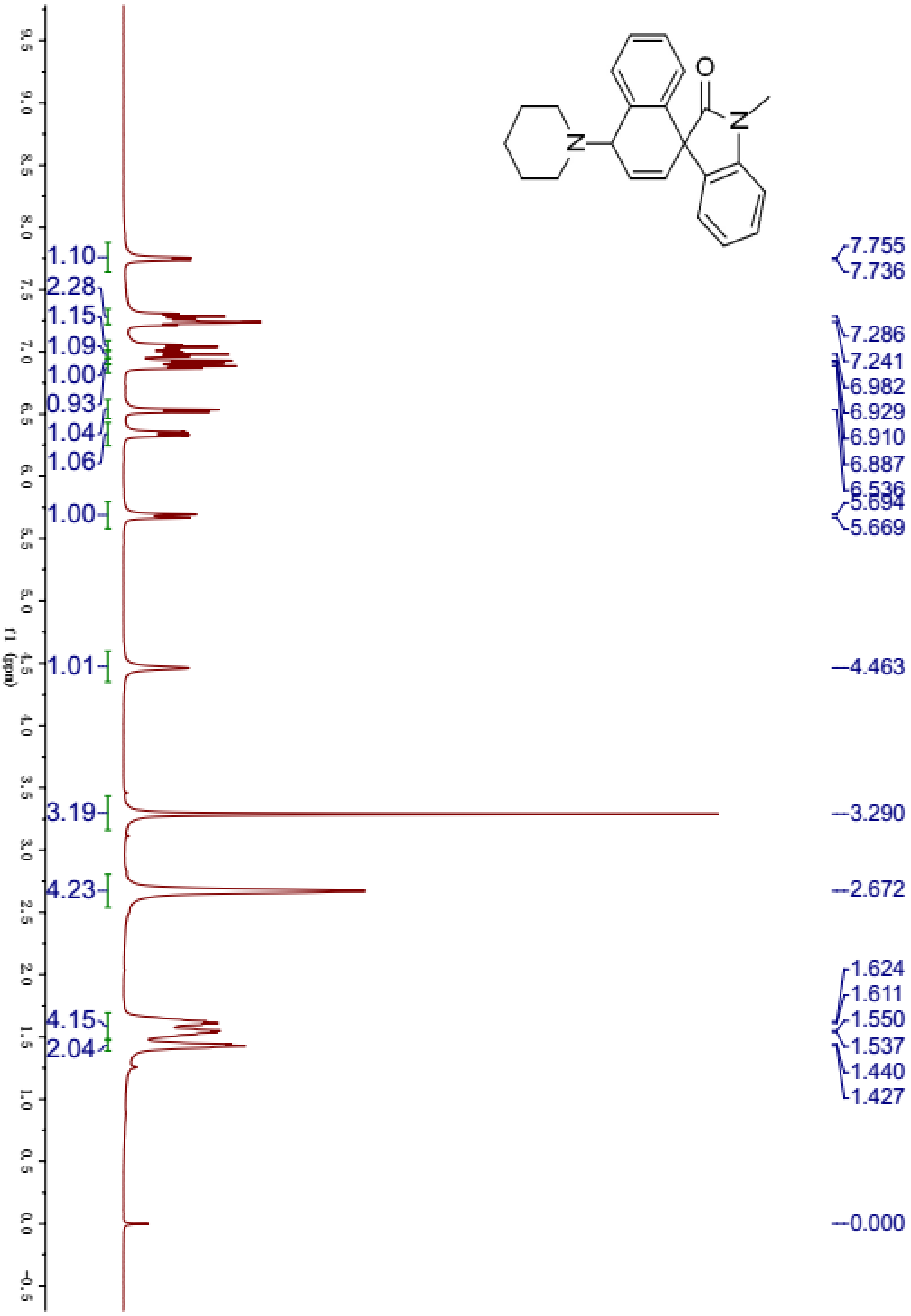
O
N
N
1.10
2.28
1.15
1.09
1.00
0.93
1.04
1.06
1.00
1.01
3.19
4.23
4.15
2.04
7.755
7.736
7.286
7.241
6.982
6.929
6.910
6.887
6.536
5.694
5.669
4.463
3.290
2.672
1.624
1.611
1.550
1.537
1.440
1.427
0.000
f1 (ppm)

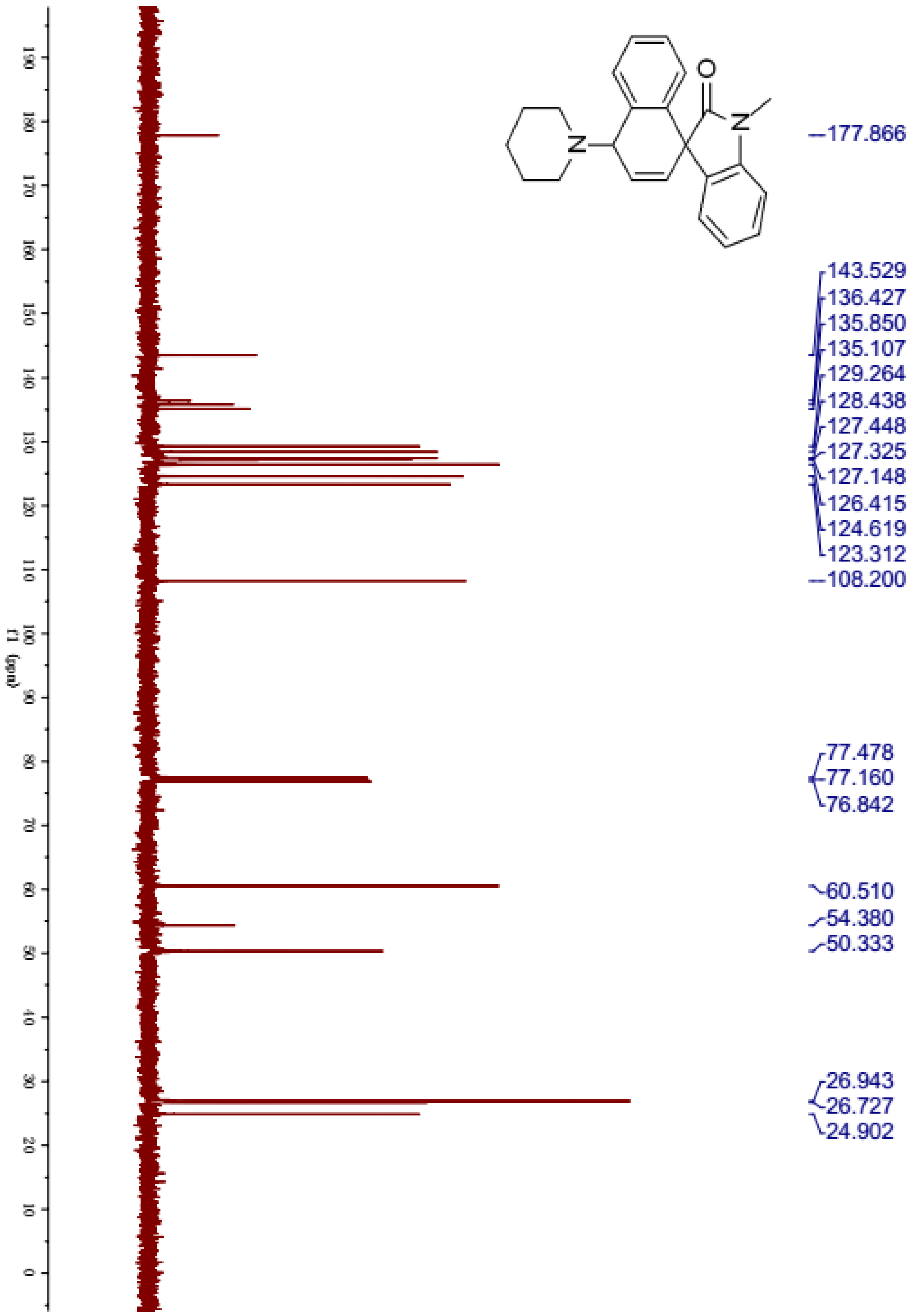

O
N
N
177.866
143.529
136.427
135.850
135.107
129.264
128.438
127.448
127.325
127.148
126.415
124.619
123.312
108.200
77.478
77.160
76.842
60.510
54.380
50.333
26.943
26.727
24.902
f1 (ppm)
190
180
170
160
150
140
130
120
110
100
90
80
70
60
50
40
30
20
10
0

6h

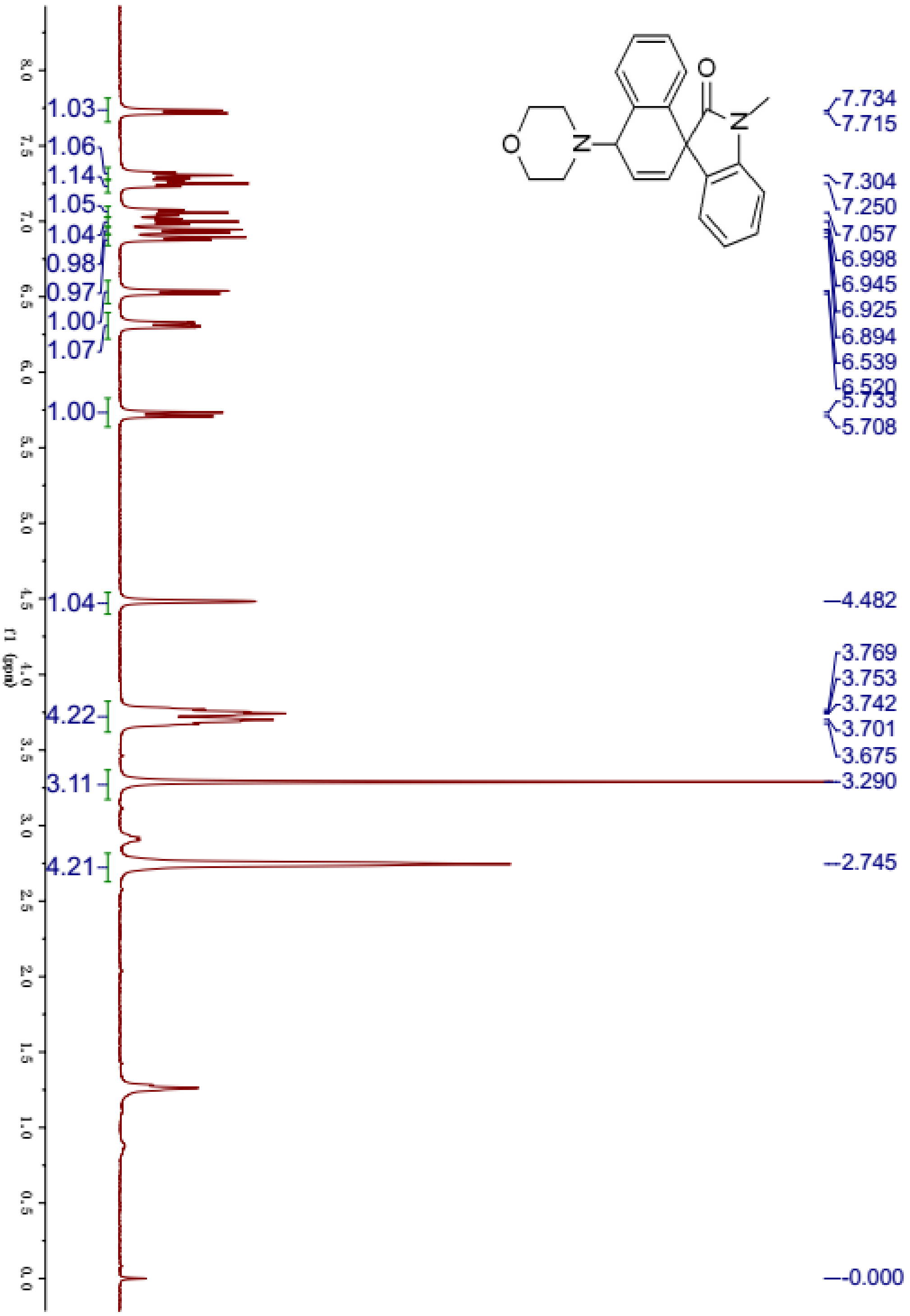
7.734
7.715
7.304
7.250
7.057
6.998
6.945
6.925
6.894
6.539
6.520
5.733
5.708
4.482
3.769
3.753
3.742
3.701
3.675
3.290
2.745
-0.000
1.03
1.06
1.14
1.05
1.04
0.98
0.97
1.00
1.07
1.00
1.04
4.22
3.11
4.21
f1 (ppm)

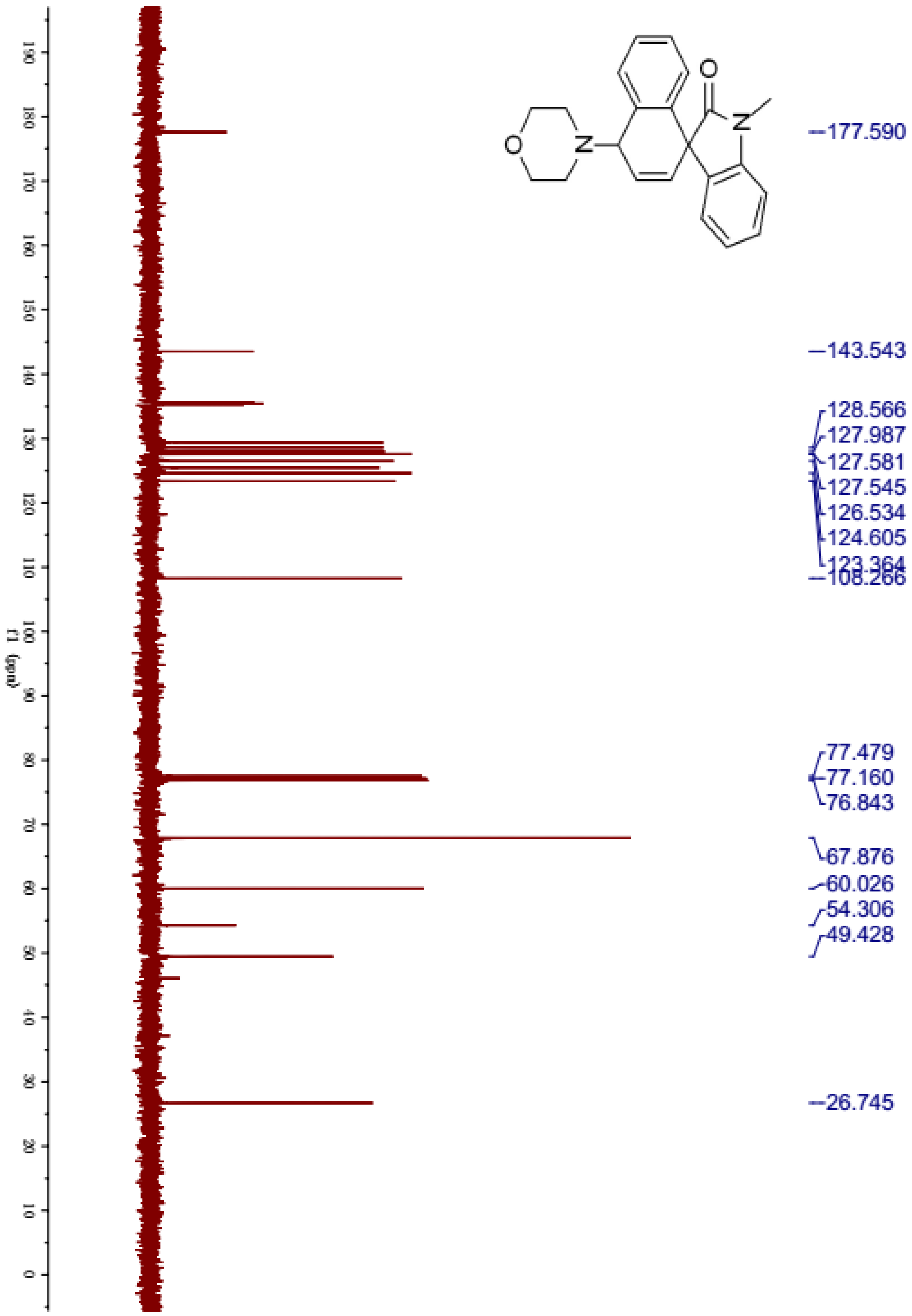
177.590
143.543
128.566
127.987
127.581
127.545
126.534
124.605
123.364
108.266
77.479
77.160
76.843
67.876
60.026
54.306
49.428
26.745
f1 (ppm)

6i

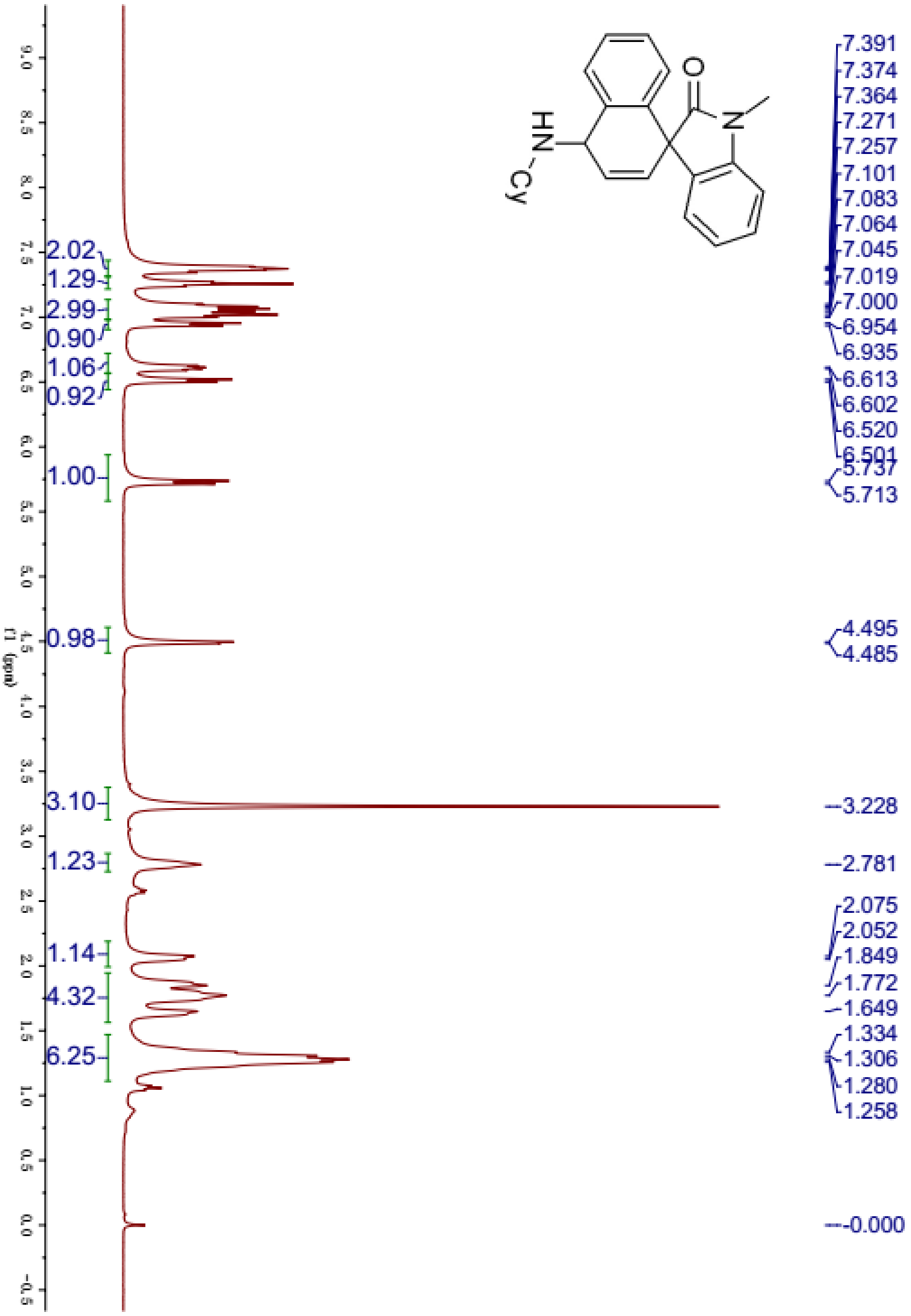
HN
Cy
O
N
7.391
7.374
7.364
7.271
7.257
7.101
7.083
7.064
7.045
7.019
7.000
6.954
6.935
6.613
6.602
6.520
6.501
5.737
5.713
4.495
4.485
3.228
2.781
2.075
2.052
1.849
1.772
1.649
1.334
1.306
1.280
1.258
-0.000
2.02
1.29
2.99
0.90
1.06
0.92
1.00
0.98
3.10
1.23
1.14
4.32
6.25
f1 (ppm)

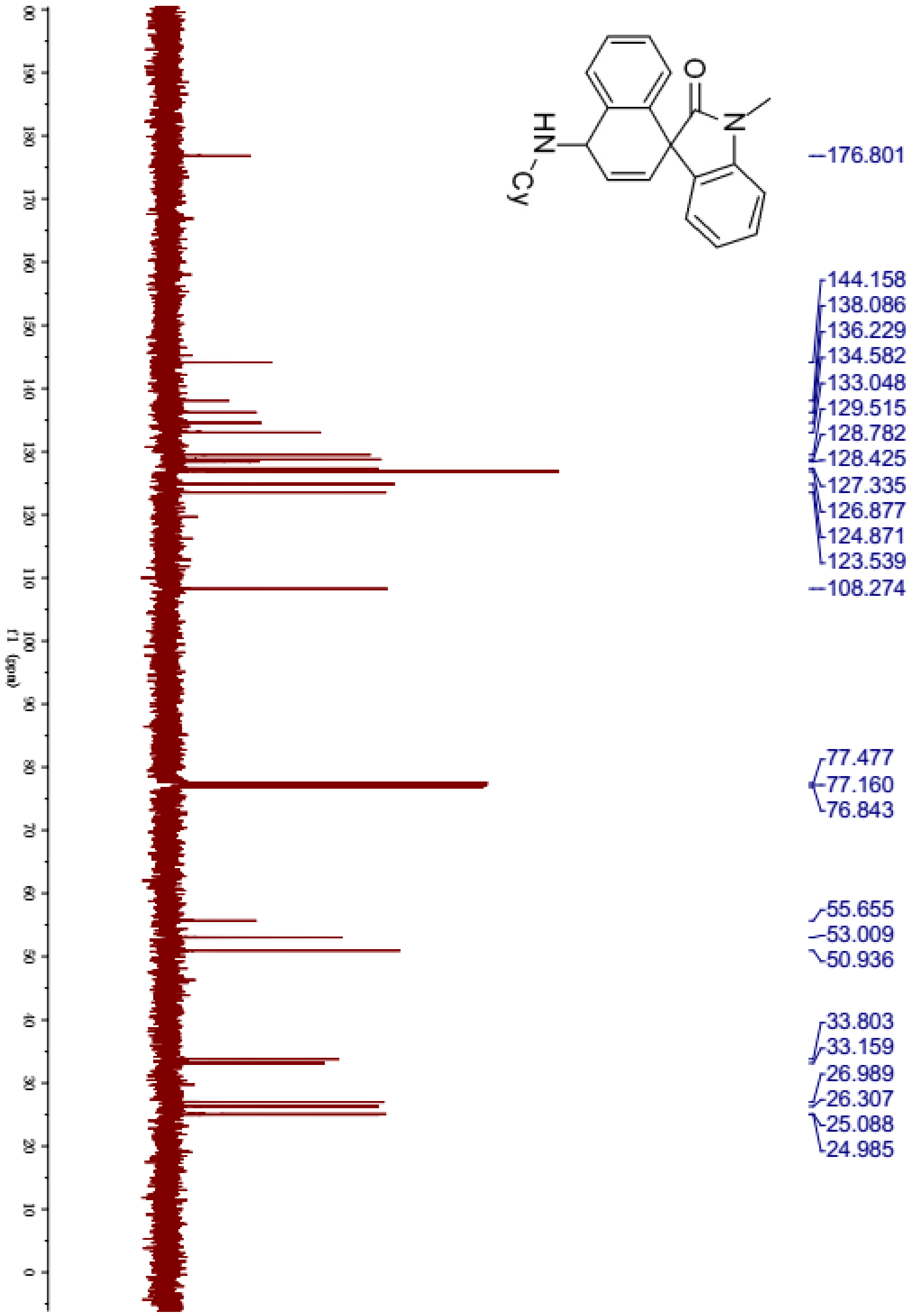
HN
Cy
O
N
176.801
144.158
138.086
136.229
134.582
133.048
129.515
128.782
128.425
127.335
126.877
124.871
123.539
108.274
77.477
77.160
76.843
55.655
53.009
50.936
33.803
33.159
26.989
26.307
25.088
24.985

6j

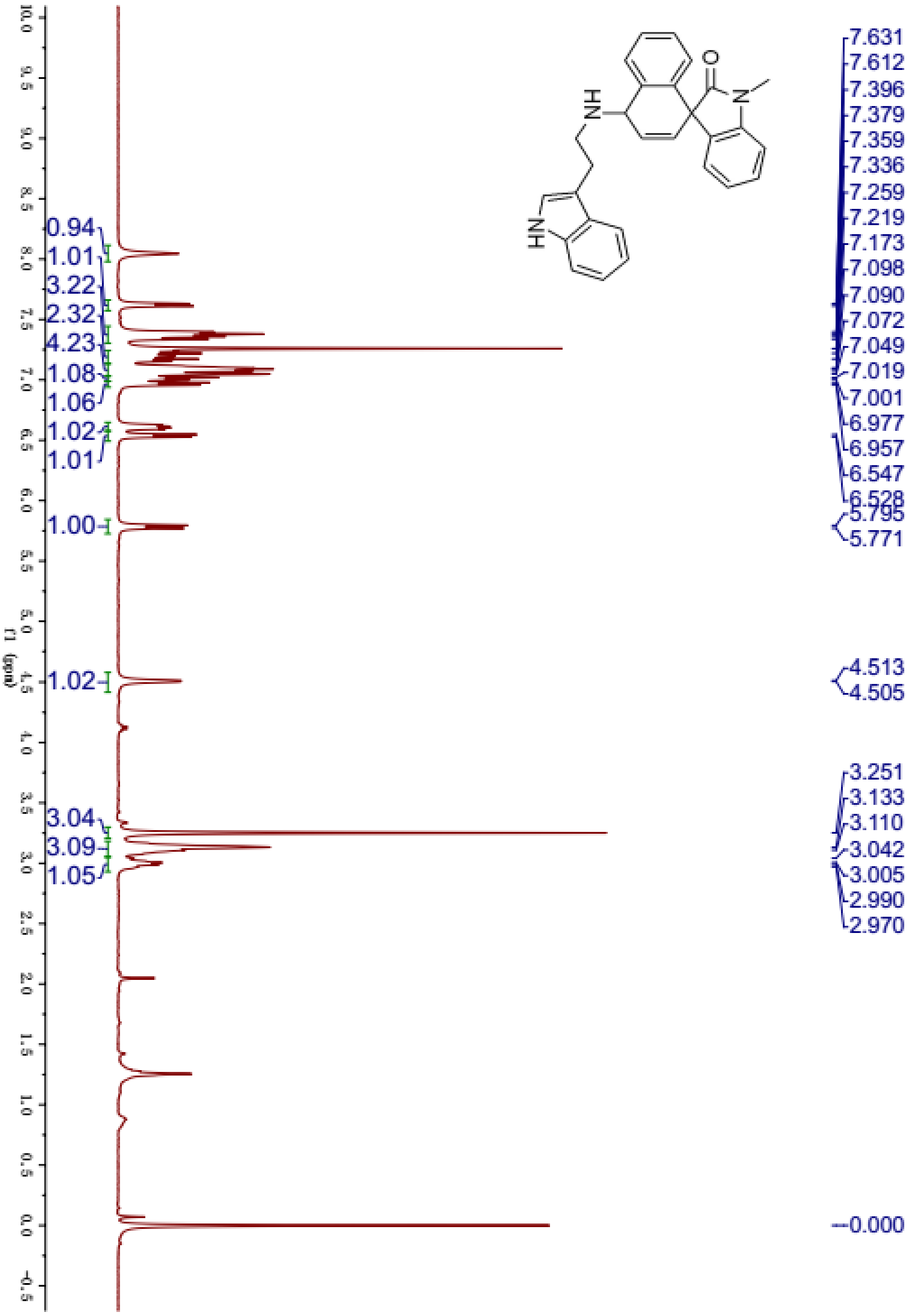
7.631
7.612
7.396
7.379
7.359
7.336
7.259
7.219
7.173
7.098
7.090
7.072
7.049
7.019
7.001
6.977
6.957
6.547
6.528
5.795
5.771
4.513
4.505
3.251
3.133
3.110
3.042
3.005
2.990
2.970
0.000
0.94
1.01
3.22
2.32
4.23
1.08
1.06
1.02
1.01
1.00
1.02
3.04
3.09
1.05
f1 (ppm)

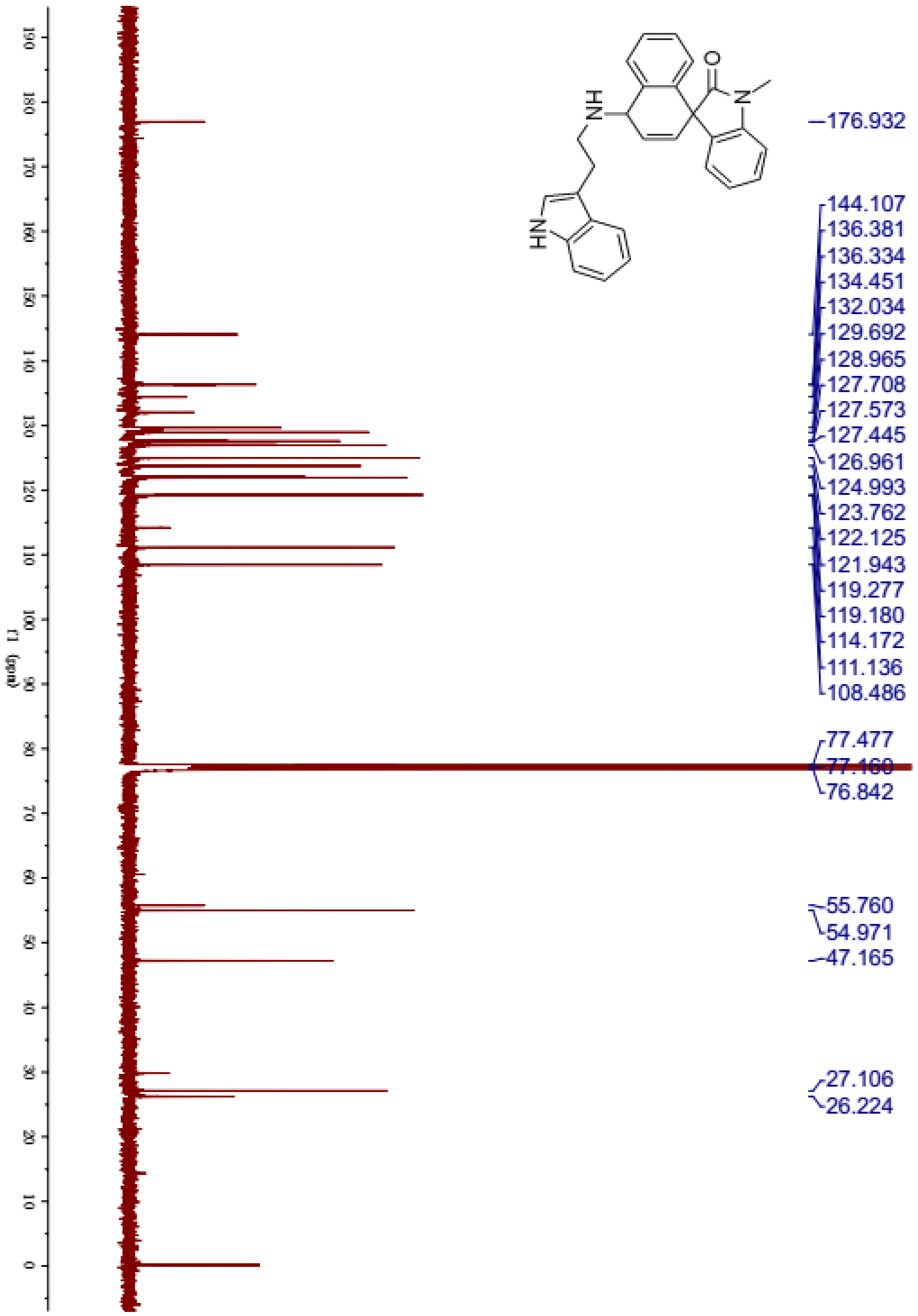
176.932
144.107
136.381
136.334
134.451
132.034
129.692
128.965
127.708
127.573
127.445
126.961
124.993
123.762
122.125
121.943
119.277
119.180
114.172
111.136
108.486
77.477
77.160
76.842
55.760
54.971
47.165
27.106
26.224
f1 (ppm)

6k

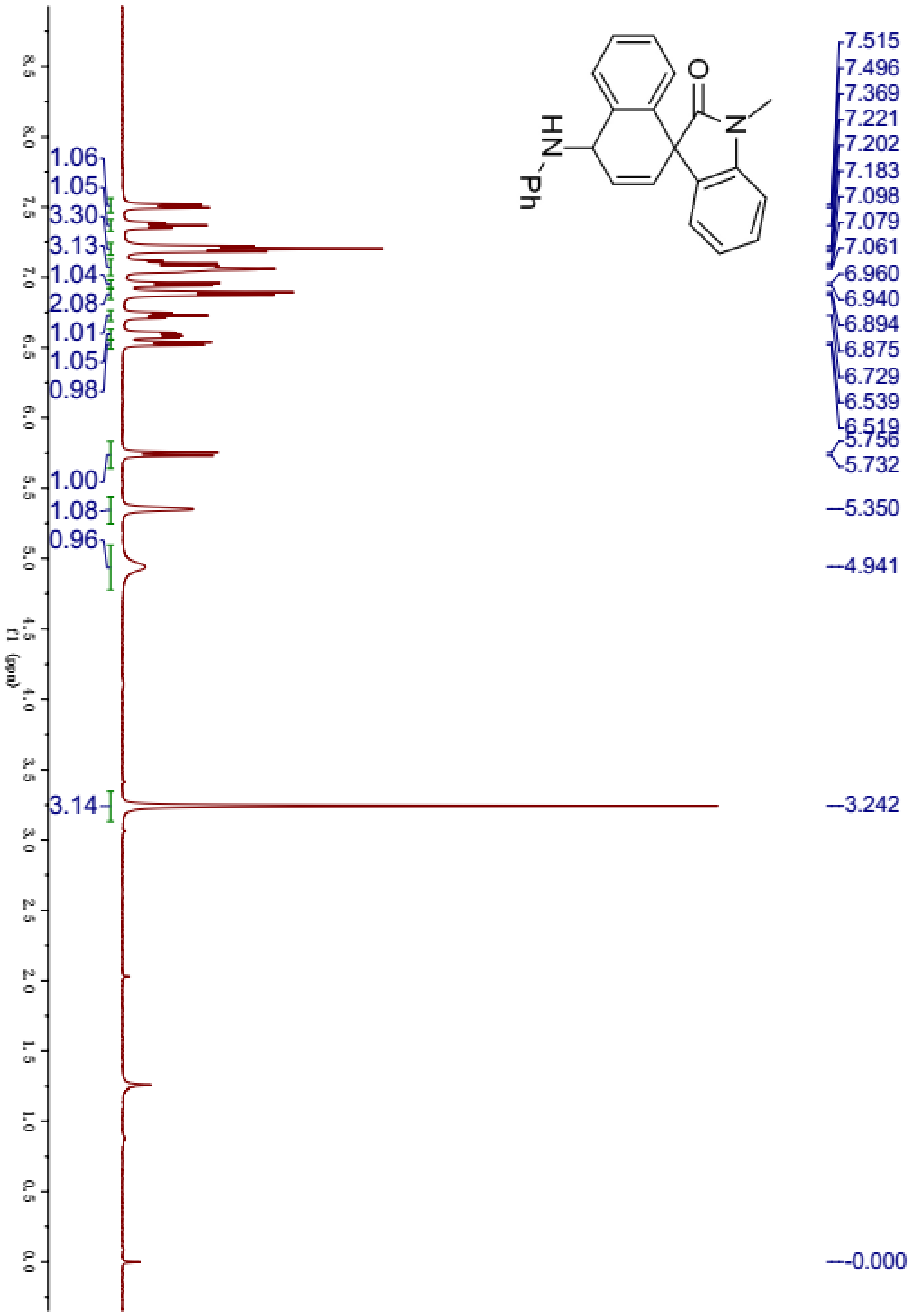

7.515
7.496
7.369
7.221
7.202
7.183
7.098
7.079
7.061
6.960
6.940
6.894
6.875
6.729
6.539
6.519
5.756
5.732
5.350
4.941
3.242
-0.000
1.06
1.05
3.30
3.13
1.04
2.08
1.01
1.05
0.98
1.00
1.08
0.96
3.14
f1 (ppm)
HN
Ph
O
N

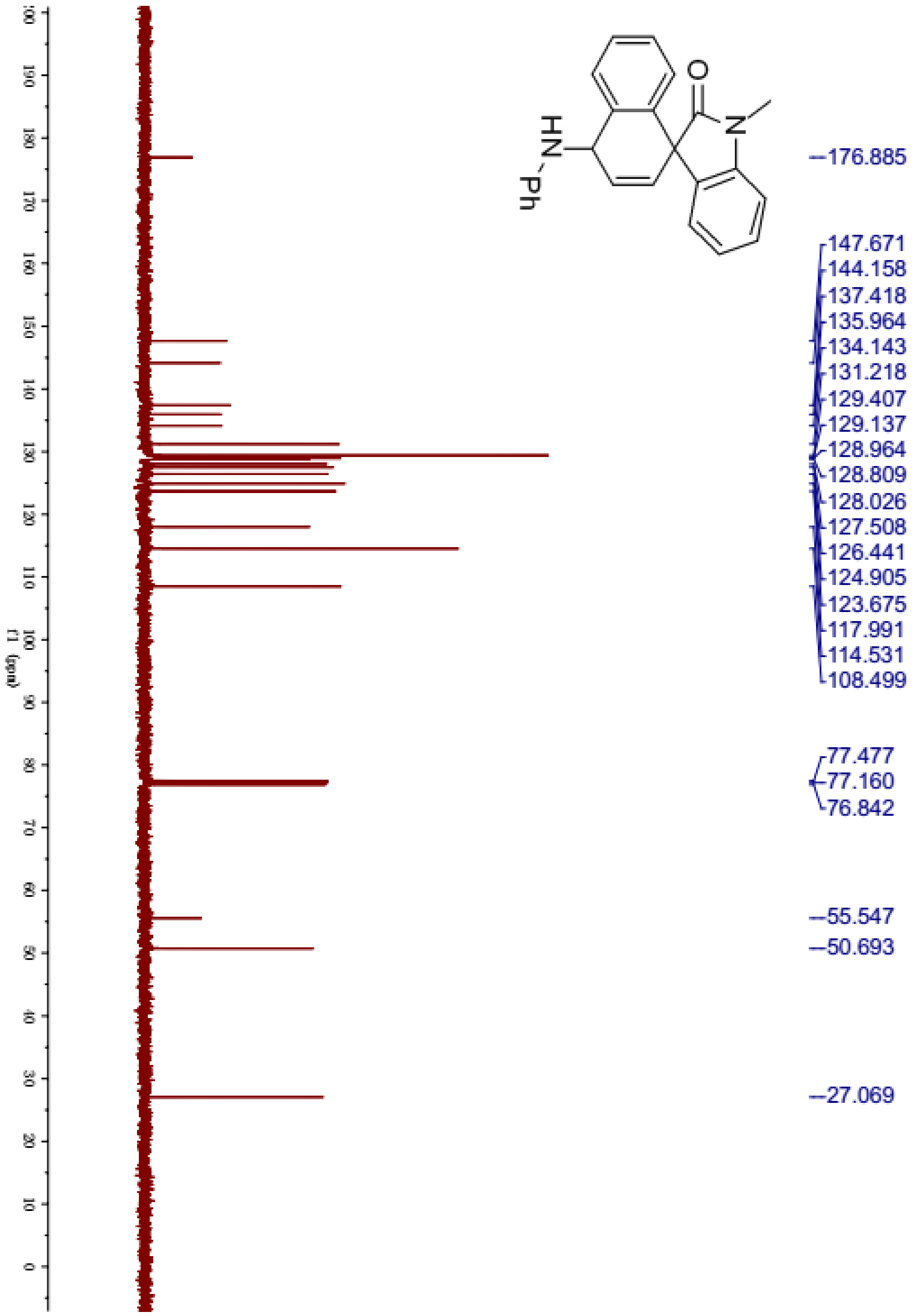
HN
Ph
O
N
176.885
147.671
144.158
137.418
135.964
134.143
131.218
129.407
129.137
128.964
128.809
128.026
127.508
126.441
124.905
123.675
117.991
114.531
108.499
77.477
77.160
76.842
55.547
50.693
27.069
f1 (ppm)

6l

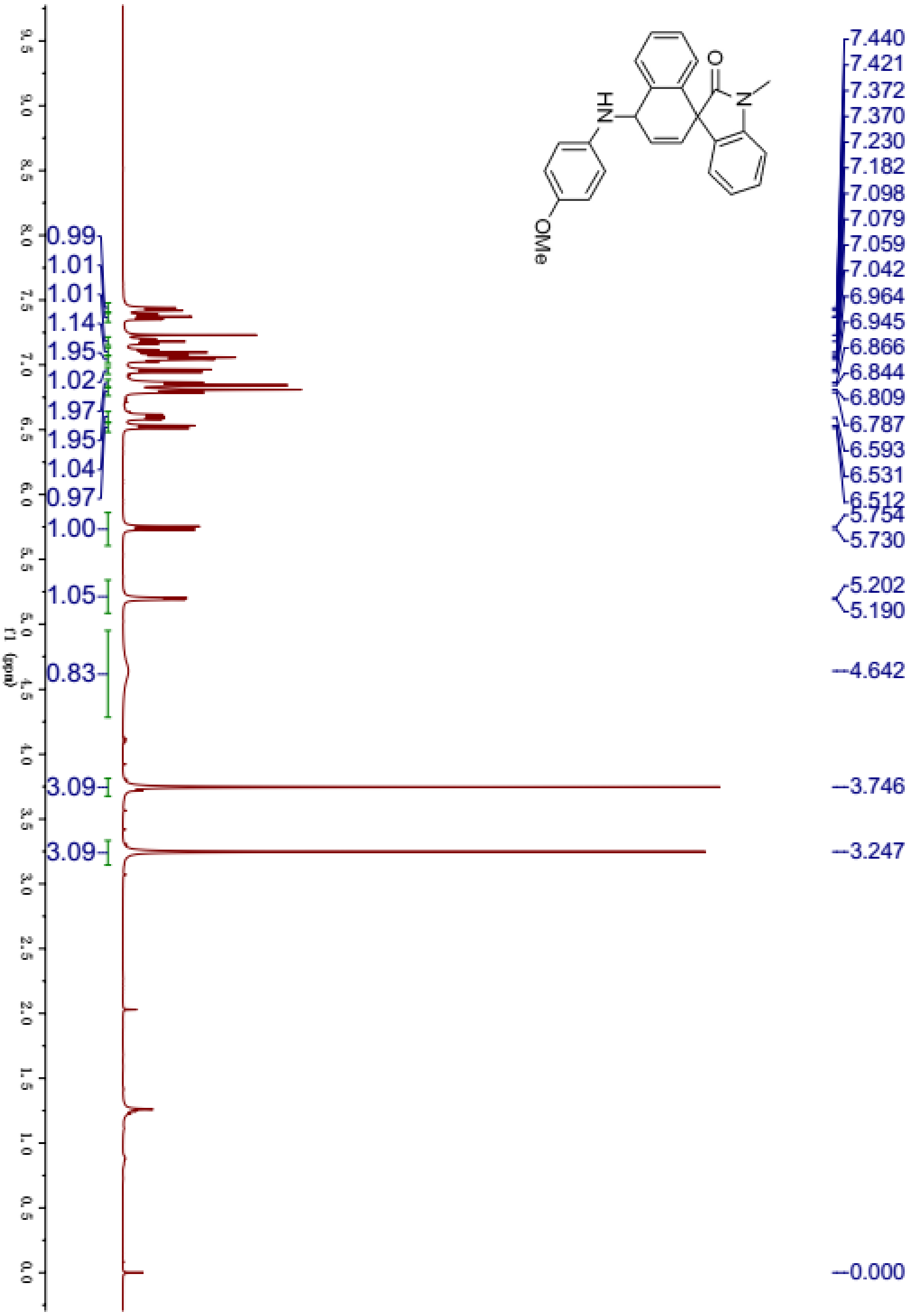

HN
O
N
OMe
7.440
7.421
7.372
7.370
7.230
7.182
7.098
7.079
7.059
7.042
6.964
6.945
6.866
6.844
6.809
6.787
6.593
6.531
6.512
5.754
5.730
5.202
5.190
4.642
3.746
3.247
0.000
0.99
1.01
1.01
1.14
1.95
1.02
1.97
1.95
1.04
0.97
1.00
1.05
0.83
3.09
3.09
f1 (ppm)

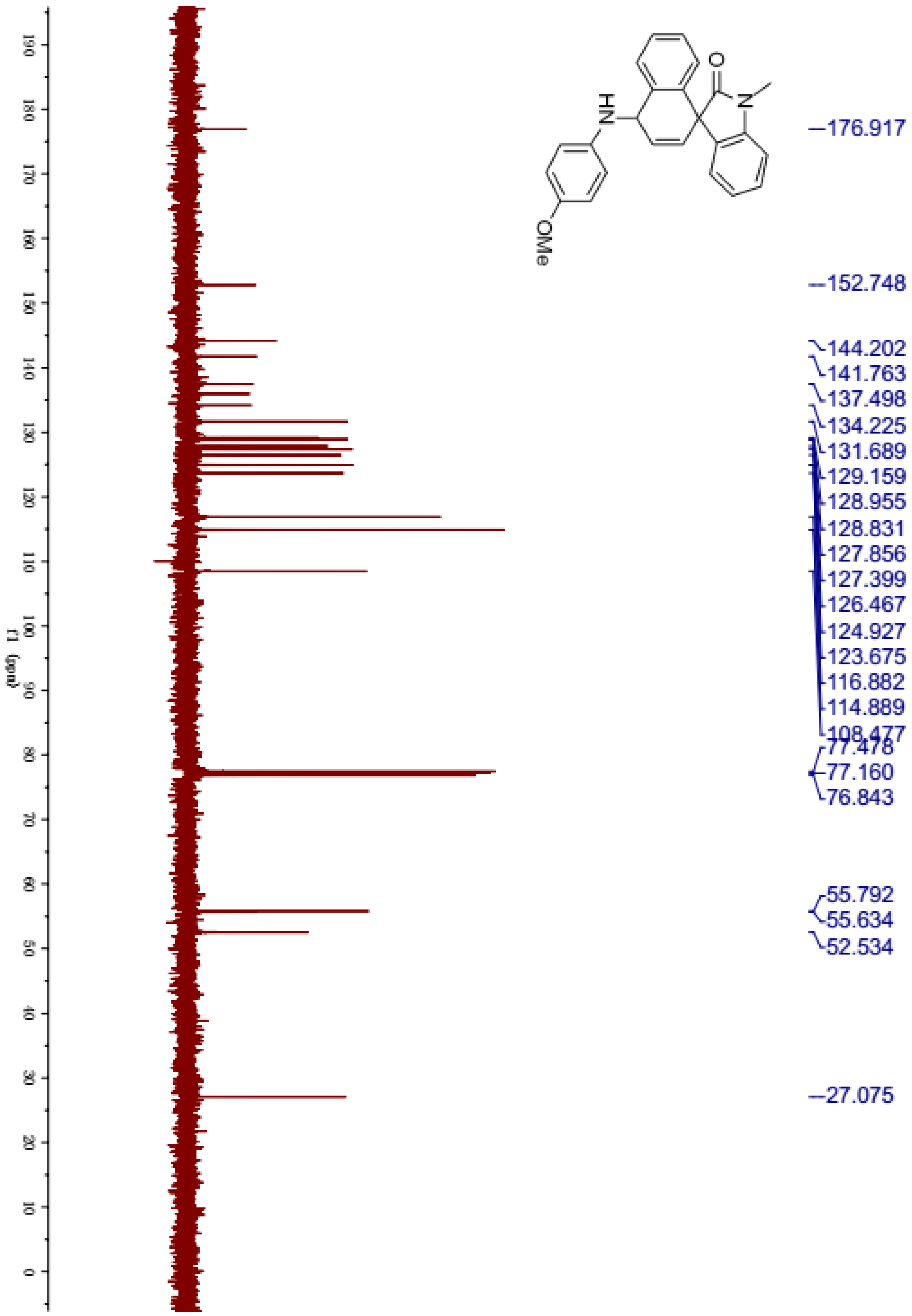

OMe
HN
O
N
176.917
152.748
144.202
141.763
137.498
134.225
131.689
129.159
128.955
128.831
127.856
127.399
126.467
124.927
123.675
116.882
114.889
108.477
77.478
77.160
76.843
55.792
55.634
52.534
27.075

6m

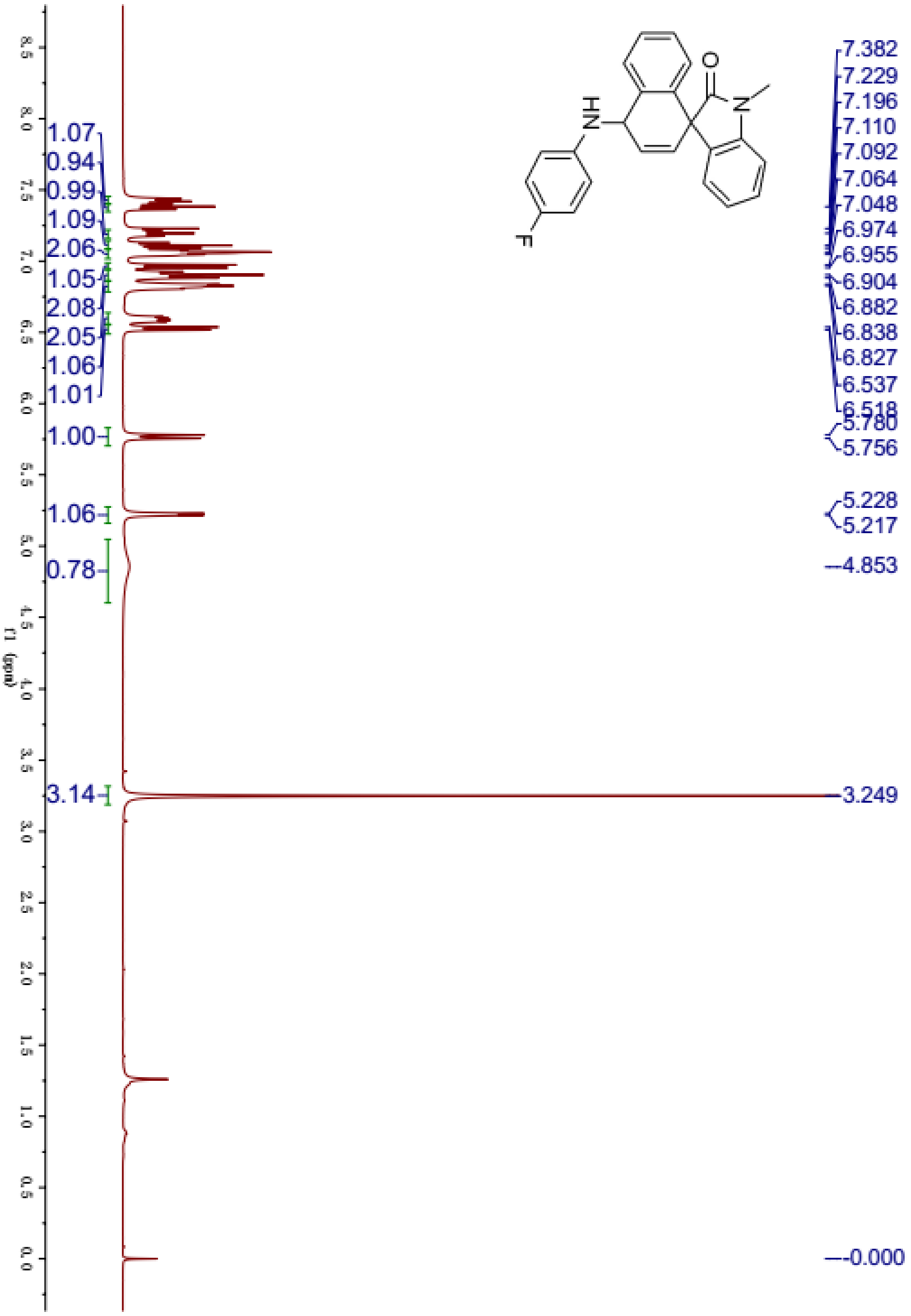
7.382
7.229
7.196
7.110
7.092
7.064
7.048
6.974
6.955
6.904
6.882
6.838
6.827
6.537
6.518
5.780
5.756
5.228
5.217
4.853
3.249
-0.000
1.07
0.94
0.99
1.09
2.06
1.05
2.08
2.05
1.06
1.01
1.00
1.06
0.78
3.14
f1 (ppm)
HN
F
O
N

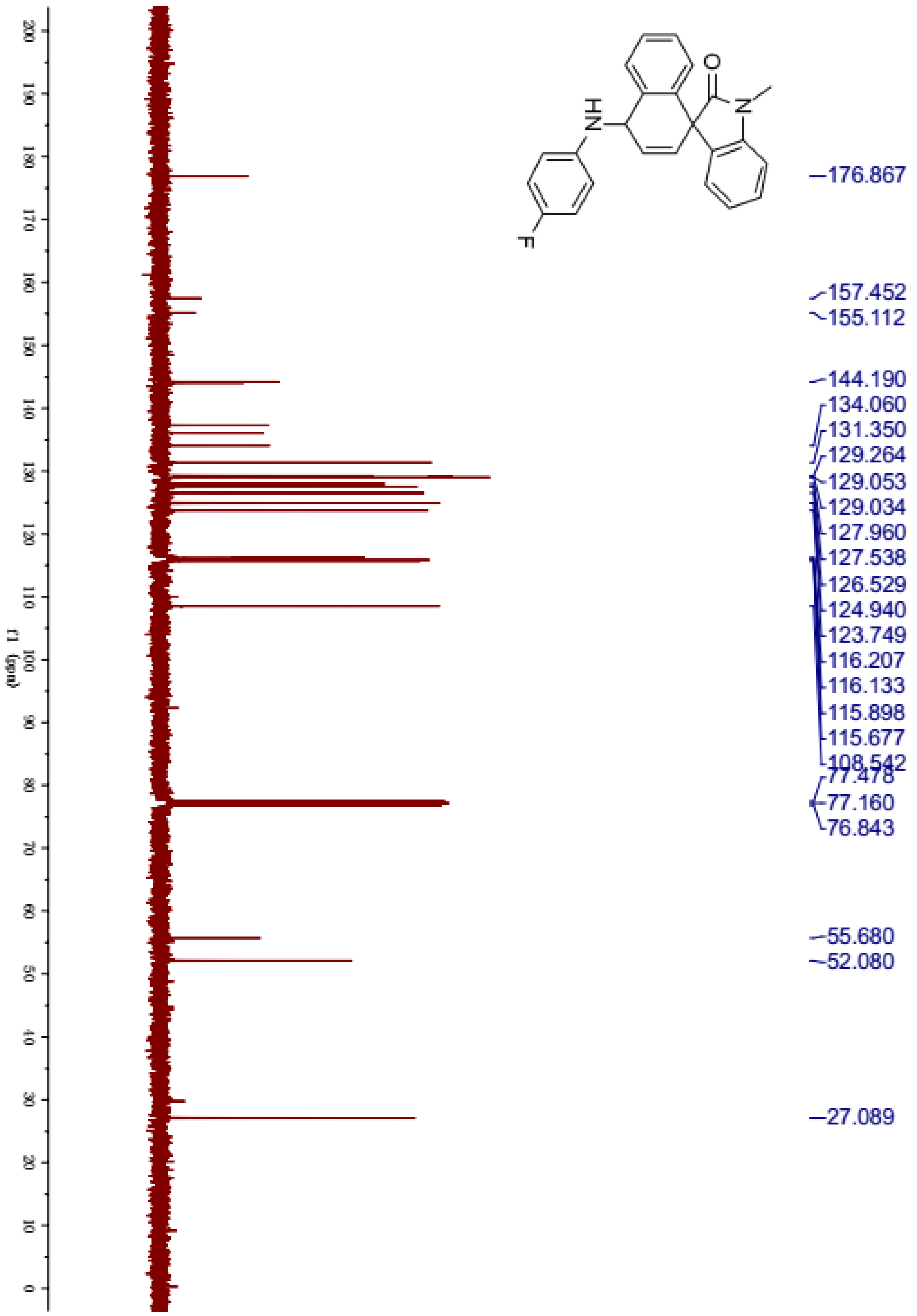
176.867
157.452
155.112
144.190
134.060
131.350
129.264
129.053
129.034
127.960
127.538
126.529
124.940
123.749
116.207
116.133
115.898
115.677
108.542
77.478
77.160
76.843
55.680
52.080
27.089
f1 (ppm)

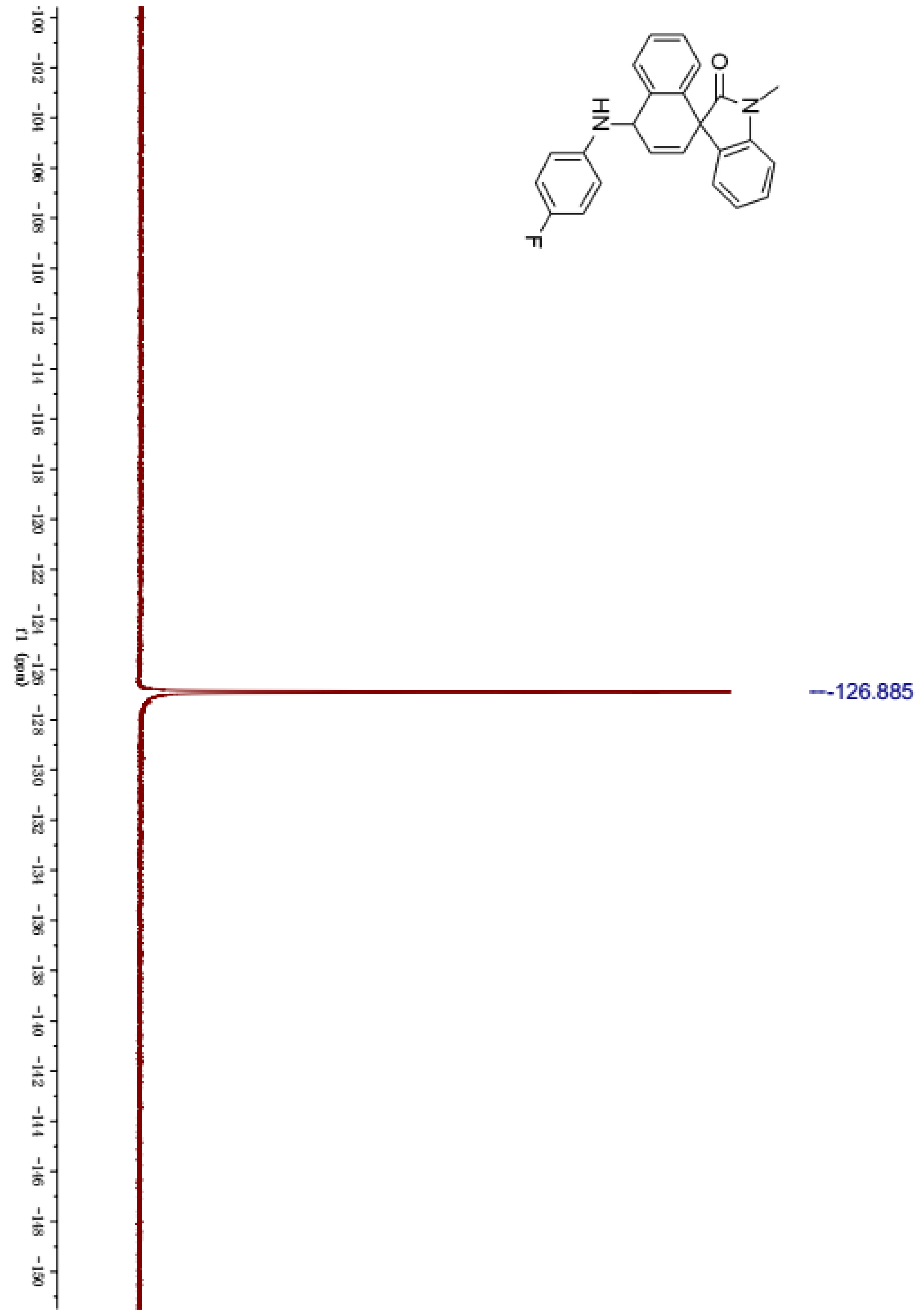

-126.885
f1 (ppm)
HN
F
O
N

6n

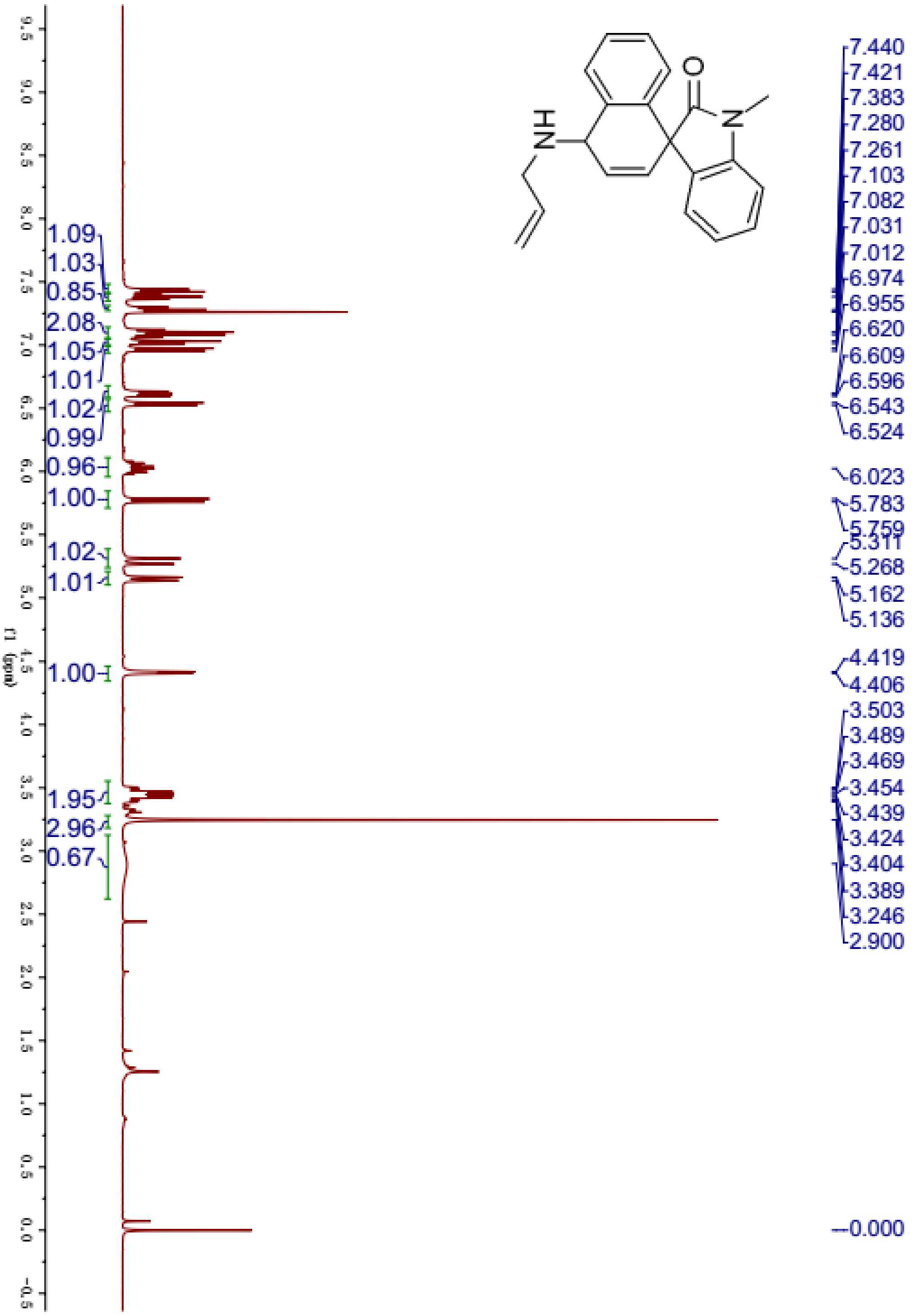
7.440
7.421
7.383
7.280
7.261
7.103
7.082
7.031
7.012
6.974
6.955
6.620
6.609
6.596
6.543
6.524
6.023
5.783
5.759
5.311
5.268
5.162
5.136
4.419
4.406
3.503
3.489
3.469
3.454
3.439
3.424
3.404
3.389
3.246
2.900
0.000
1.09
1.03
0.85
2.08
1.05
1.01
1.02
0.99
0.96
1.00
1.02
1.01
1.00
1.95
2.96
0.67
f1 (ppm)
O
N
HN

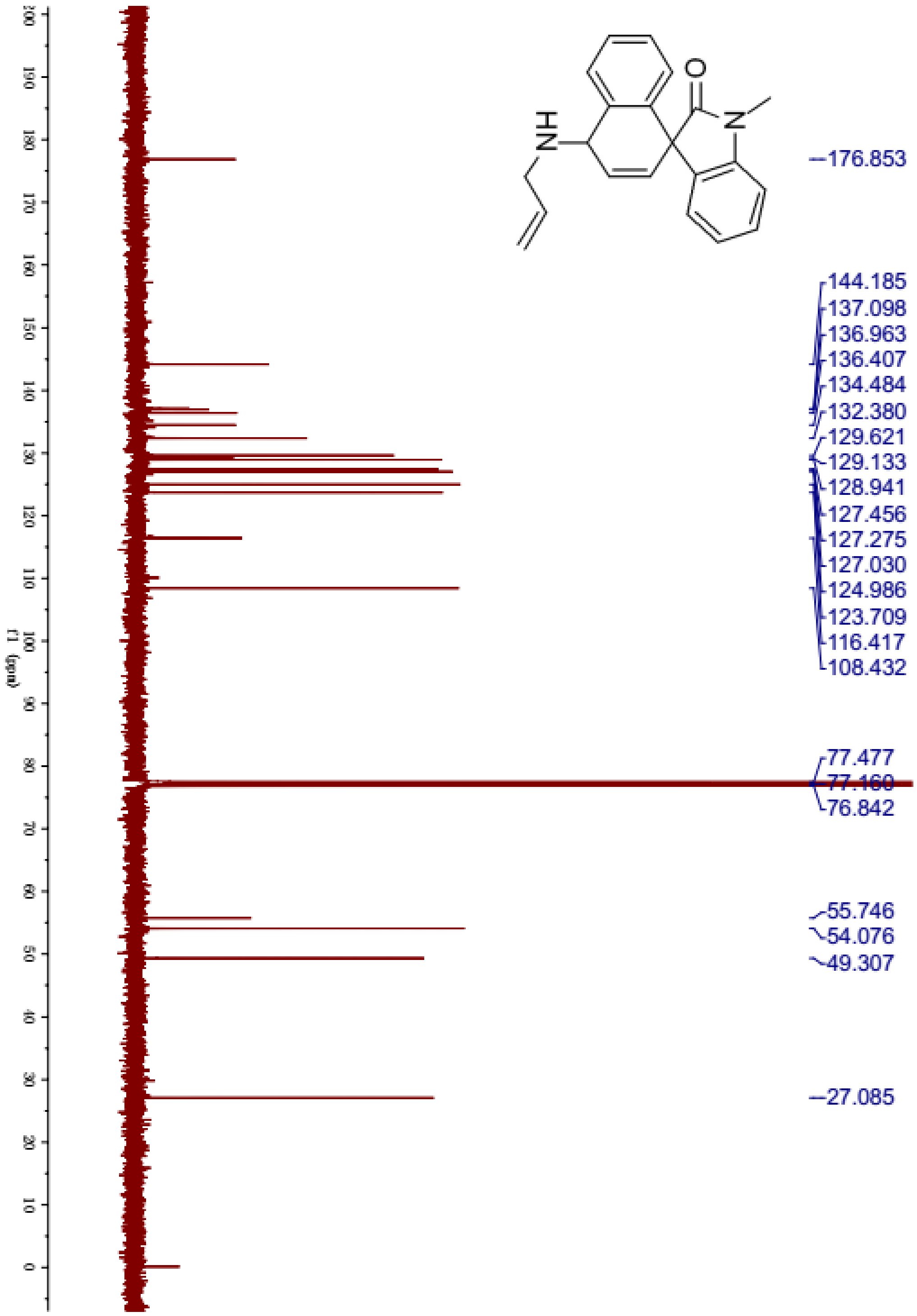
HN
O
N
176.853
144.185
137.098
136.963
136.407
134.484
132.380
129.621
129.133
128.941
127.456
127.275
127.030
124.986
123.709
116.417
108.432
77.477
77.160
76.842
55.746
54.076
49.307
27.085
f1 (ppm)

6o

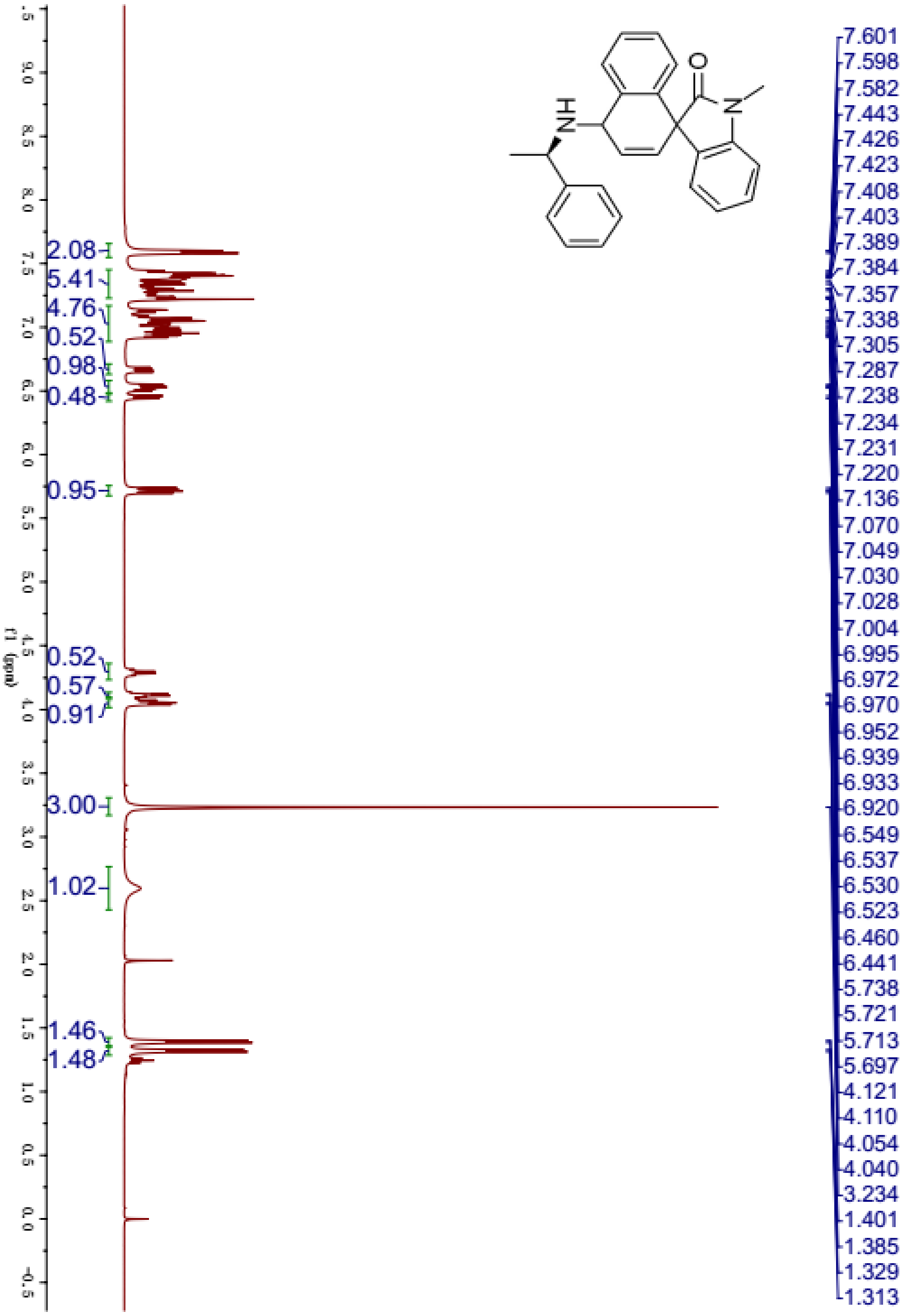
7.601
7.598
7.582
7.443
7.426
7.423
7.408
7.403
7.389
7.384
7.357
7.338
7.305
7.287
7.238
7.234
7.231
7.220
7.136
7.070
7.049
7.030
7.028
7.004
6.995
6.972
6.970
6.952
6.939
6.933
6.920
6.549
6.537
6.530
6.523
6.460
6.441
5.738
5.721
5.713
5.697
4.121
4.110
4.054
4.040
3.234
1.401
1.385
1.329
1.313
2.08
5.41
4.76
0.52
0.98
0.48
0.95
0.52
0.57
0.91
3.00
1.02
1.46
1.48
f1 (ppm)

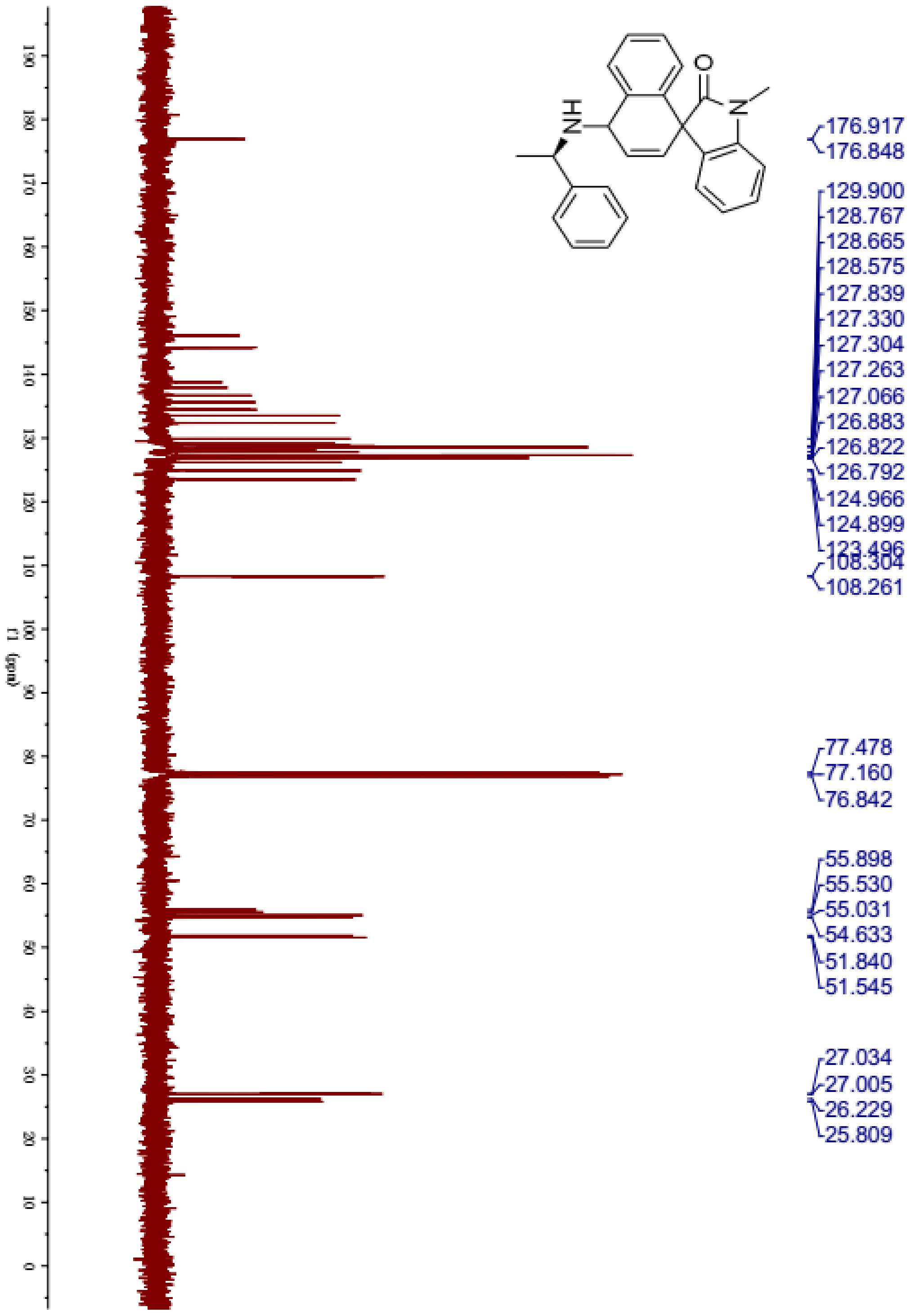
176.917
176.848
129.900
128.767
128.665
128.575
127.839
127.330
127.304
127.263
127.066
126.883
126.822
126.792
124.966
124.899
123.496
108.304
108.261
77.478
77.160
76.842
55.898
55.530
55.031
54.633
51.840
51.545
27.034
27.005
26.229
25.809
f1 (ppm)

7

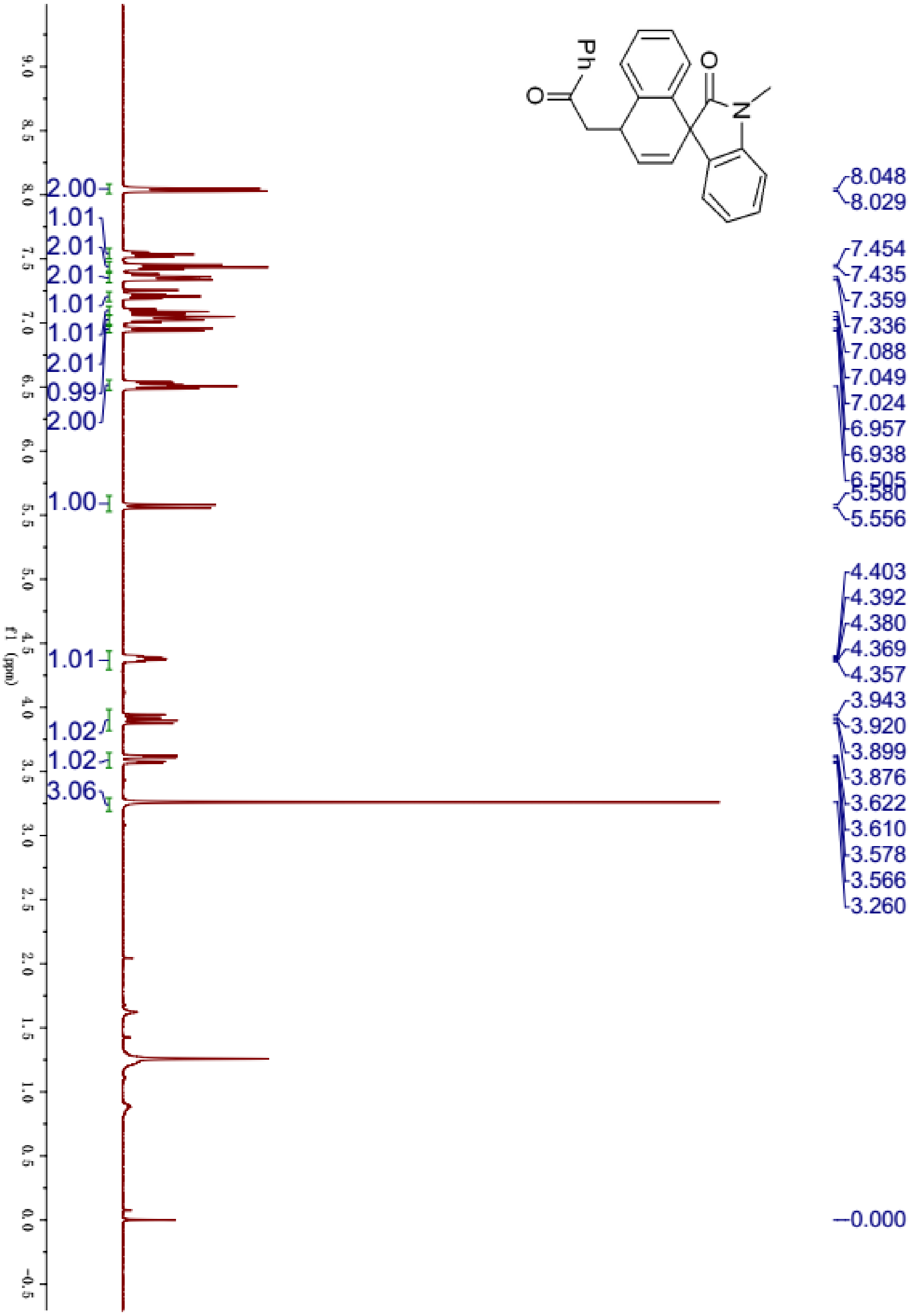
Ph
O
O
N
8.048
8.029
7.454
7.435
7.359
7.336
7.088
7.049
7.024
6.957
6.938
6.505
5.580
5.556
4.403
4.392
4.380
4.369
4.357
3.943
3.920
3.899
3.876
3.622
3.610
3.578
3.566
3.260
-0.000
2.00
1.01
2.01
2.01
1.01
1.01
2.01
0.99
2.00
1.00
1.01
1.02
1.02
3.06
f1 (ppm)

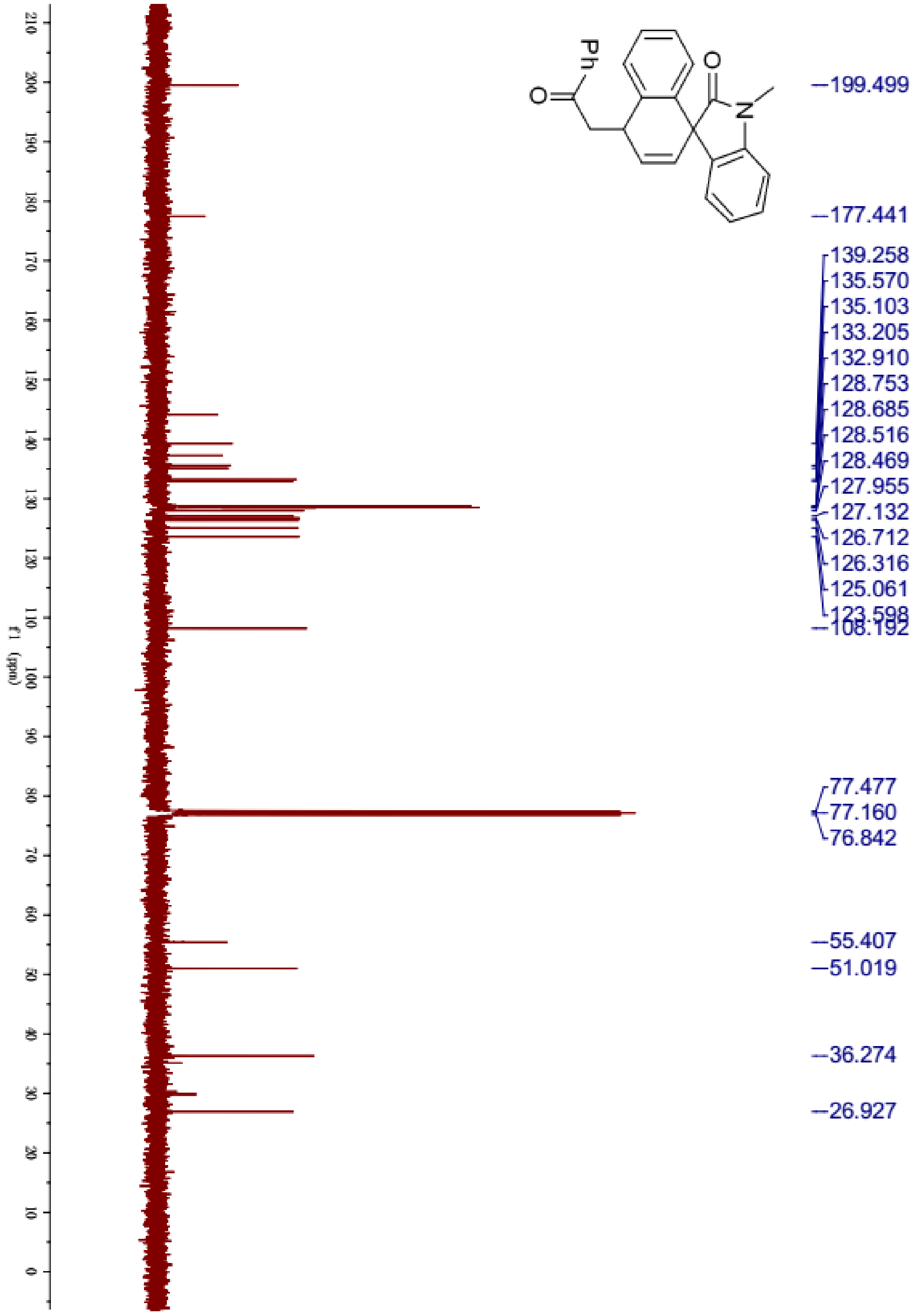

Ph
O
O
N
199.499
177.441
139.258
135.570
135.103
133.205
132.910
128.753
128.685
128.516
128.469
127.955
127.132
126.712
126.316
125.061
123.598
108.192
77.477
77.160
76.842
55.407
51.019
36.274
26.927
f1 (ppm)
210
200
190
180
170
160
150
140
130
120
110
100
90
80
70
60
50
40
30
20
10
0

8

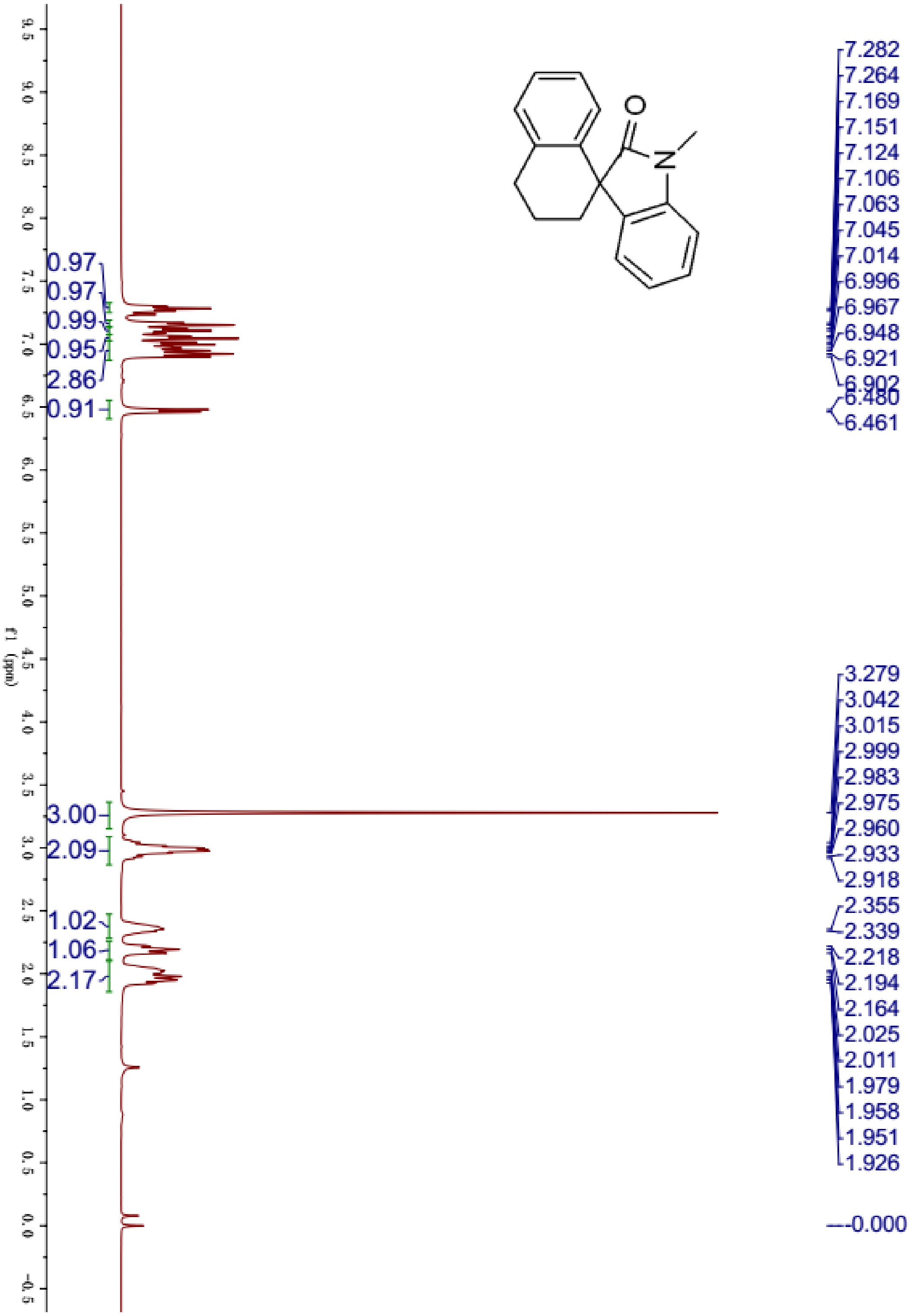
7.282
7.264
7.169
7.151
7.124
7.106
7.063
7.045
7.014
6.996
6.967
6.948
6.921
6.902
6.480
6.461
3.279
3.042
3.015
2.999
2.983
2.975
2.960
2.933
2.918
2.355
2.339
2.218
2.194
2.164
2.025
2.011
1.979
1.958
1.951
1.926
-0.000
0.97
0.97
0.99
0.95
2.86
0.91
3.00
2.09
1.02
1.06
2.17
f1 (ppm)

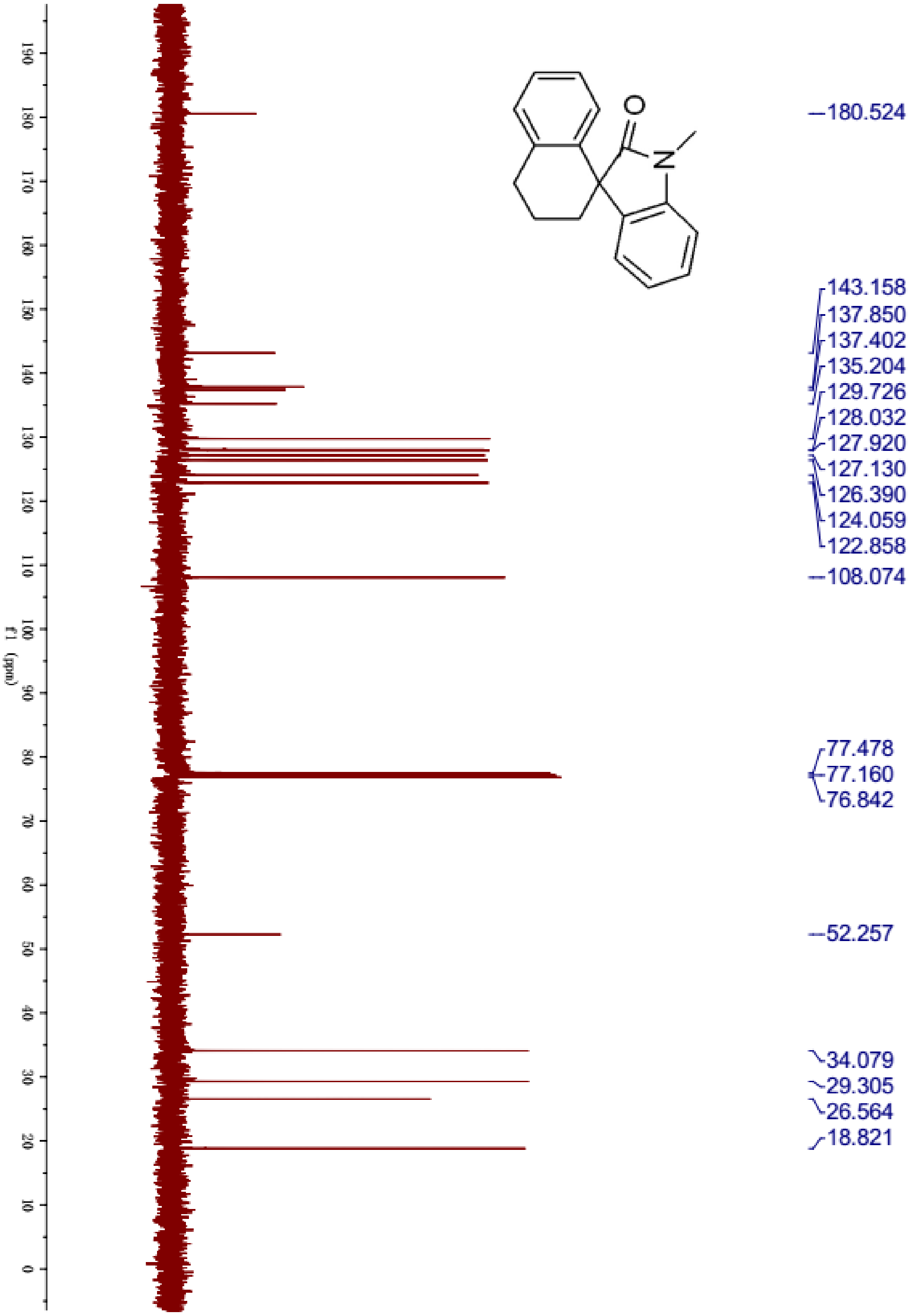
180.524
143.158
137.850
137.402
135.204
129.726
128.032
127.920
127.130
126.390
124.059
122.858
108.074
77.478
77.160
76.842
52.257
34.079
29.305
26.564
18.821

9

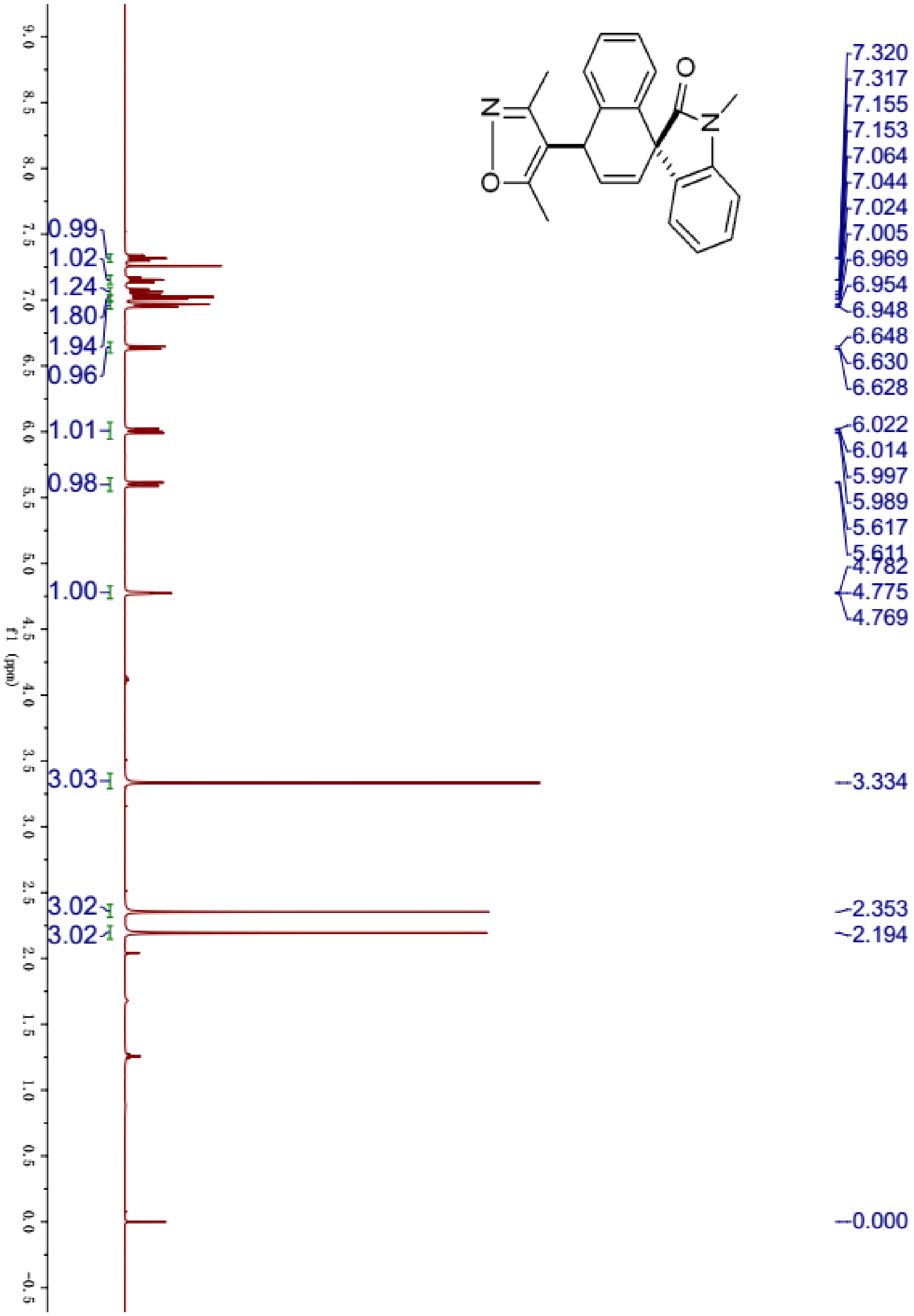
7.320
7.317
7.155
7.153
7.064
7.044
7.024
7.005
6.969
6.954
6.948
6.648
6.630
6.628
6.022
6.014
5.997
5.989
5.617
5.611
4.782
4.775
4.769
3.334
2.353
2.194
0.000
0.99
1.02
1.24
1.80
1.94
0.96
1.01
0.98
1.00
3.03
3.02
3.02
f1 (ppm)
O
N
N
O

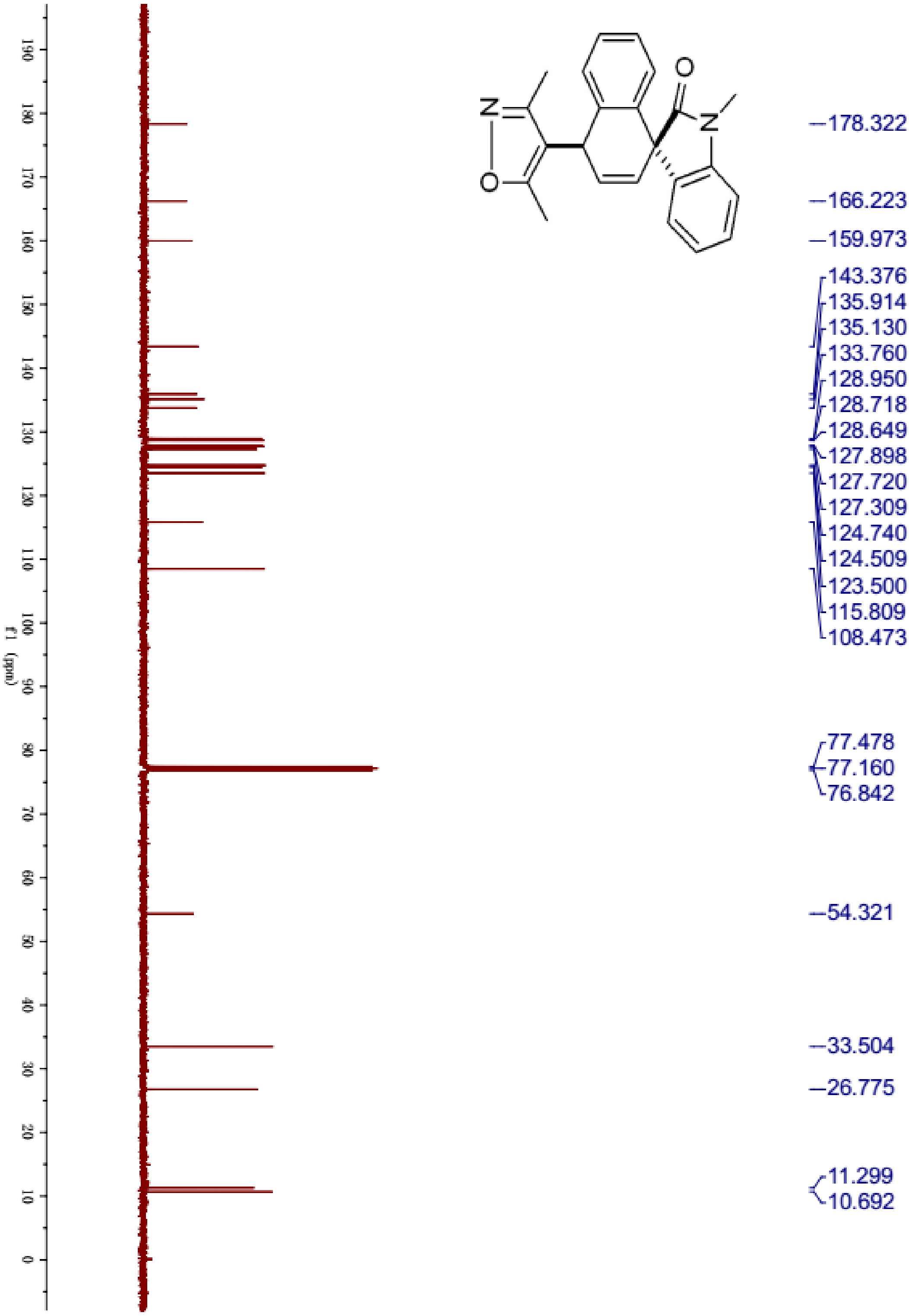

178.322
166.223
159.973
143.376
135.914
135.130
133.760
128.950
128.718
128.649
127.898
127.720
127.309
124.740
124.509
123.500
115.809
108.473
77.478
77.160
76.842
54.321
33.504
26.775
11.299
10.692
f1 (ppm)

10

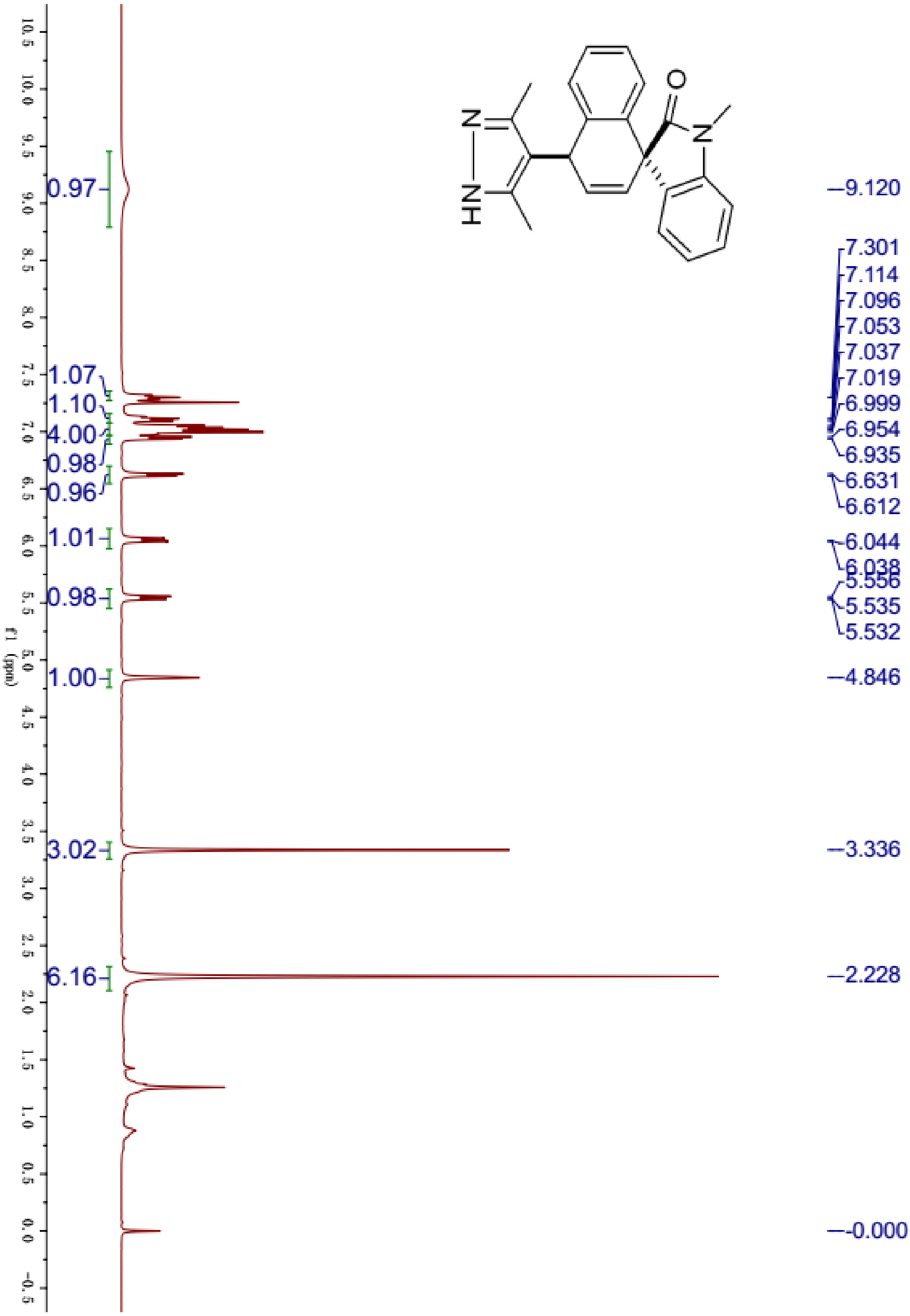
HN
N
N
O
0.97
1.07
1.10
4.00
0.98
0.96
1.01
0.98
1.00
3.02
6.16
9.120
7.301
7.114
7.096
7.053
7.037
7.019
6.999
6.954
6.935
6.631
6.612
6.044
6.038
5.556
5.535
5.532
4.846
3.336
2.228
-0.000
f1 (ppm)

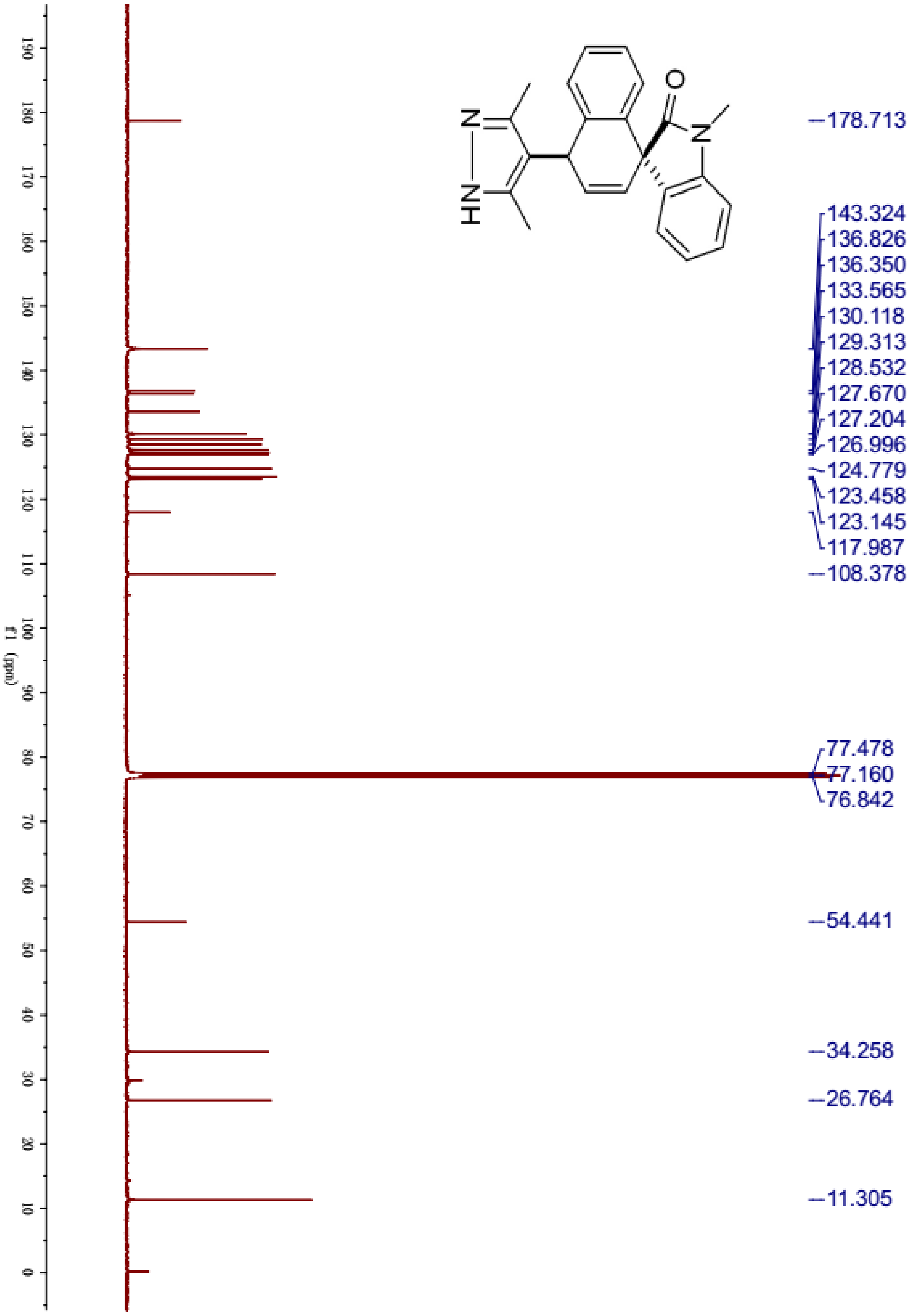
178.713
143.324
136.826
136.350
133.565
130.118
129.313
128.532
127.670
127.204
126.996
124.779
123.458
123.145
117.987
108.378
77.478
77.160
76.842
54.441
34.258
26.764
11.305
f1 (ppm)
